# Synthesis of Di-, Tri-, and Tetrasubstituted Oxetanes by Rhodium-Catalyzed O=H Insertion and C=C Bond-Forming Cyclization**

**DOI:** 10.1002/anie.201408928

**Published:** 2014-10-14

**Authors:** Owen A Davis, James A Bull

**Affiliations:** Department of Chemistry, Imperial College LondonSouth Kensington, London SW7 2AZ (UK)

**Keywords:** cyclization, diazo compounds, heterocycles, O=H insertion, oxetanes

## Abstract

Oxetanes offer exciting potential as structural motifs and intermediates in drug discovery and materials science. Here an efficient strategy for the synthesis of oxetane rings incorporating pendant functional groups is described. A wide variety of oxetane 2,2-dicarboxylates were accessed in high yields, including functionalized 3-/4-aryl- and alkyl-substituted oxetanes and fused oxetane bicycles. Enantioenriched alcohols provided enantioenriched oxetanes with complete retention of configuration. The oxetane products were further derivatized, while the ring was maintained intact, thus highlighting their potential as building blocks for medicinal chemistry.

Oxetanes are widely used compounds in numerous branches of science. As strained cyclic ethers, they find uses in polymer and materials science, as monomers for cationic ring-opening polymerizations,[1] and as crosslinkers.[2] Oxetanes are employed as intermediates in synthetic chemistry in processes featuring ring expansion,[3] ring opening,[4] or rearrangement processes.[5] The oxetane motif is also found in natural products and other biologically active compounds (Figure 1).[6]

**Figure 1:**
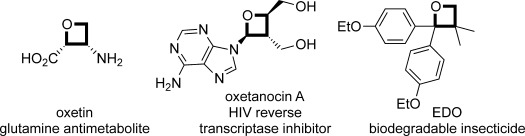
Biologically important oxetane-containing compounds.

Recently, oxetanes have received considerable attention in drug discovery and have been widely adopted in medicinal chemistry.[7,8] In this context, they are considered stable adjuncts to improve solubility, lipophilicity, and other physicochemical properties toward drug-like molecules. Pioneering studies by Carreira and co-workers demonstrated oxetane motifs to be effective polar replacements for *gem*-dimethyl groups and carbonyl derivatives.[7,9] Furthermore, compared to larger oxygen heterocycles, the small ring displays a metabolic robustness which is linked to its inherent lower lipophilicity.[10]

Classical oxetane synthesis includes intramolecular Williamson ether synthesis[11] and Paterno–Büchi [2+2] photo-cycloadditions (Scheme 1 a).[12] More recently, ring-opening/closing from epoxides using sulfoxonium ylides has been developed,[13] though this approach has not been extended to more functionalized derivatives. Current medicinal chemistry investigations are largely focused on 3-substituted oxetanes (Scheme 1 b). These are often derived from Carreira’s oxetan-3-one,[7,14] or from 3-iodooxetane by Suzuki cross-coupling[15] or other methods.[16,17] We recently reported a cyclization strategy for oxetane synthesis involving C=C bond formation in order to prepare 2-(arylsulfonyl)oxetanes as fragments for fragment-based drug discovery (Scheme 1 c).[18]

**Scheme 1:**
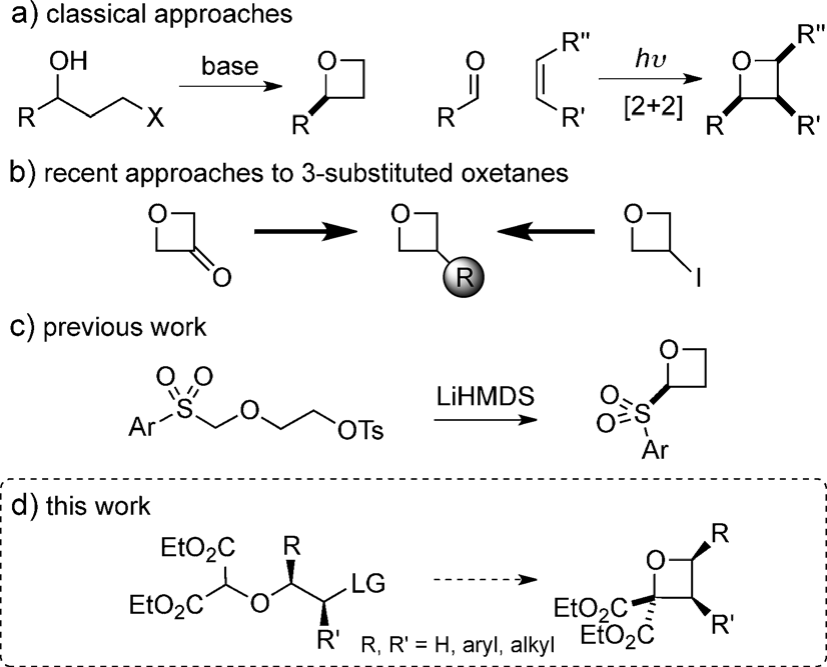
Approaches to oxetane derivatives. LG=leaving group, LiHMDS=1,1,1,3,3,3-hexamethyldisilazane, Ts=toluene-4-sulfonyl.

We considered that efficient methods to access more highly substituted and chiral oxetanes would afford interesting novel appendages or core scaffolds for drug discovery.[19] Oxetanes offer six possible vectors from which to develop a compound in three dimensions,[20] and such derivatives would constitute new chemical space for exploration in medicinal chemistry. However, the synthesis of diversely substituted oxetanes bearing functional groups remains a challenge. We proposed that applying a C=C bond-forming strategy would provide access to such oxetane derivatives. Here we report a mild and efficient O=H insertion and C=C bond-forming cyclization strategy for oxetane synthesis (Scheme 1 d). Functionalized di-, tri-, and tetrasubstituted oxetane derivatives are rapidly generated as building blocks and potential scaffolds of interest for medicinal chemistry. Further elaboration of these motifs is also reported.

A key aspect of our strategy was to develop a mild and widely applicable approach to the cyclization precursor. The activation of X=H bonds (X=N, O, S) through metal-catalyzed diazo decomposition and carbenoid insertion is a powerful approach to C=X bond construction.[21] We envisaged that insertion of a diazo compound that bears anion-stabilizing groups into the O=H bond of functionalized alcohols could rapidly deliver the required cyclization precursors. We targeted the synthesis of oxetane 2,2-dicarboxylates to facilitate the cyclization and install the ester functionality for further manipulation. A functional-group-tolerant O=H insertion would permit a convergent strategy with the required leaving group in place, as well as the incorporation of useful functionality to decorate the oxetane core.

We initially attempted an O=H insertion reaction using ethylene glycol and diazomalonate **1** (Scheme 5. 2). This transformation provided ether **2 a** in 77 % yield using catalytic [Rh_2_(OAc)_4_] and excess ethylene glycol.[22] Treatment with TsCl afforded **2 b** in high yield. Alternatively, tosylate **2 b** was accessed by O=H insertion on ethylene glycol monotosylate to obtain the cyclization precursor.

**Scheme 2:**
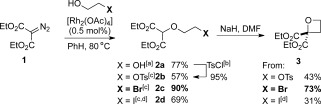
O=H insertion/cyclization strategy to diethyl 2,2-oxetane-dicarboxylate. See the Supporting Information for details of optimization for each substrate. [a] Using 1 as limiting reagent (10 equiv ethylene glycol). [b] TsCl, Et_3_N, Me_3_N⋅HCl. [c] Using alcohol as limiting reagent (1.5 equiv 1). [d] Using conditions optimized for bromide substrate. DMF=*N*,*N*-dimethylformamide.

To our delight, tosylate **2 b** cyclized successfully to oxetane **3** through the formation of the C=C bond by using NaH in DMF. Despite extensive examination of the reaction conditions, the yield of 43 % could not be improved.[22] Therefore, we examined alternative leaving groups, using functionalized ethanol derivatives. Following optimization, bromide **2 c** was obtained in 90 % yield by O=H insertion using 2-bromoethanol.[23] Iodoethanol was also successful but with reduced yields because of the instability of iodide **2 d**. The bromide leaving group was shown to be most effective for the cyclization and full optimization was conducted with bromide **2 c**. The developed conditions (NaH (1.2 equiv) in DMF (0.025 m) for 1 h at 0 °C) afforded oxetane **3** in 73 % yield on a 1.0 mmol scale.[22] This sequence constitutes an efficient two-step oxetane synthesis from readily available starting materials.

With these optimized conditions, the introduction of substituents onto bromoethanol was examined in order to form chiral oxetanes. Aryl substituents were examined using readily available β-bromohydrins to prepare 2,2,4-substituted oxetanes (Table 1).[22] For the phenyl-substituted bromohydrin **4 a**, the Rh-catalyzed O=H insertion gave a high yield under the conditions developed above. However, the cyclization required a longer reaction time (16 h) and higher temperature (25 °C) to achieve an excellent yield (Table 1, entry 1).[22] The sequence was then performed with enantioenriched β-bromohydrin (*S*)-**4 a**.[24a] As expected, the *ee* value was perfectly retained through both steps to access enantioenriched oxetane (*R*)-**6 a** (Table 1, entry 2). This reduces the requirements for enantioselective oxetane synthesis to enantioselective bromohydrin synthesis.[24]

**Table 1:** Synthesis of diethyl 4-aryl-2,2-oxetane dicarboxylates. 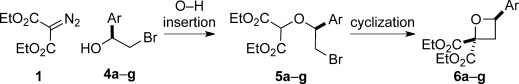

Entry	Ar		Yield5 [%][a]	Yield6 [%][b]
1 2[c]	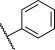	**a**	79 84 (85 % *ee*)	84 83 (85 % *ee*)
3	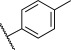	**b**	76	77
4	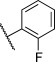	**c**	95	81
5 6	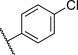	**d**	70 88[d]	76 77[e]
7	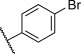	**e**	68	83
8	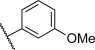	**f**	73	88
9	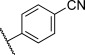	**g**	71	82

[a]  O=H insertion conditions: **4** (1.0–3.0 mmol), **1** (1.5 equiv), [Rh_2_(OAc)_4_] (0.5 mol %), PhH, 0.1 m, 80 °C.

[b] Cyclization conditions: **5** (0.5–1.0 mmol), NaH (1.2 equiv), DMF, 0.025 m, 25 °C, 16 h.

[c] Enantioenriched (*S*)-**4 a** (85 % *ee*).

[d] Reaction on 9.0 mmol scale.

[e] Reaction on 6.5 mmol scale.

The *p*-tolyl derivative gave high yields through both steps, as did aryl fluoride, chloride, and bromide derivatives (Table 1, entries 3–7). To demonstrate the scalability of the procedure, chlorophenyl derivative **6 d** was prepared on a larger scale, affording over 1.5 g of the oxetane. Electron-rich and electron-poor aromatic substituents gave similarly high yields (Table 1, entries 8 and 9).

Next, alkyl substituents were examined to generate trisubstituted 4-alkyloxetanes (Table 2). Under the conditions employed with the aryl substituents, both steps were effective. Benzyl and phenyl ethers were well tolerated (Table 2, entries 1 and 2).

**Table 2:** Synthesis of diethyl 4-alkyl-2,2-oxetane dicarboxylates. 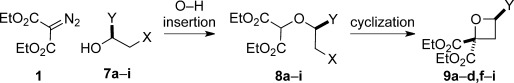

Entry	X	Y		Yield8 [%][a]	Yield9 [%][b]
1	Br	CH_2_OBn	**a**	67	89
2	Br	CH_2_OPh	**b**	92	65
3	Br	CH_2_Br	**c**	51[c]	81
4	Br	CH_2_Cl	**d**	80	45/7 (Y=Cl/Br, **9 d**/**9 c**)
5	Cl	CH_2_Cl	**e**	86	77 (**9 d**)
6	Cl	CH_2_O*i*Pr	**f**	97	75
7	Cl	CH_2_OTBS	**g**	65	71
8	Br	CF_3_	**h**	28[d]	43
9	Br	CH_3_[e]	**i**	98[f]	82[g]

[a] O=H insertion conditions: **7** (1.0–3.0 mmol), **1** (1.5 equiv), [Rh_2_(OAc)_4_] (0.5 mol %), PhH, 0.1 m, 80 °C.

[b] Cyclization conditions: **8** (0.4–1.0 mmol), NaH (1.2 equiv), DMF, 0.025 m, 25 °C, 16 h.

[c] Heated at 80 °C for 3 d.

[d] Yield over two steps from 3-bromo-1,1,1-trifluoroacetone.

[e] From technical grade 1-bromo-2-propanol (**7 i**) containing 20 wt % 2-bromo-1-propanol.

[f] Mixture of regioisomers (4:1).

[g] Mixture of regioisomers (4-Me/3-Me oxetanes 5.4:1). Bn=benzyl, TBS=*tert*-butyldimethylsilyl.

The O=H insertion reaction with 1,3-dibromo-2-propanol (**7 c**) occurred cleanly to generate dibromide **8 c**, but required a longer reaction time and gave a slightly reduced yield, presumably as a result of increased steric demands (Table 2, entry 3). Treatment of **8 c** with NaH afforded bromomethyloxetane **9 c** in a high yield, providing an alkyl bromide handle for further derivatization (see below).

In order to generate the corresponding chloromethyloxetane, bromide **8 d** was formed from 1-bromo-3-chloro-2-propanol (**7 d**). When the cyclization was attempted, both chloromethyloxetane **9 d** (major product) and bromomethyloxetane **9 c** were isolated (Table 2, entry 4). This result demonstrated that chlorides were also viable leaving groups for oxetane synthesis. Subsequently, 1,3-dichloro-2-propanol (**7 e**) was employed to generate dichloride **8 e** in high yield, which successfully underwent cyclization to chloromethyloxetane **9 d** (Table 2, entry 5). Similarly, a single chloride as leaving group also gave high yields in both O=H insertion and cyclization steps (Table 2, entries 6 and 7), including TBS ether **9 g**. 4-Trifluoromethyl-substituted oxetane **9 h** was successfully prepared from 3-bromo-1,1,1,-trifluoroacetone (Table 2, entry 8). We examined the installation of a methyl substituent starting from commercially available 1-bromo-2-propanol (**7 i**), consisting of a 4:1 mixture with 2-bromo-1-propanol (technical grade, Table 2, entry 9). The O=H insertion occurred in high yield and was equally effective for both isomers. Pleasingly, the cyclization occurred for both regioisomers to afford a mixture of 3-methyl- and 4-methyl-substituted oxetanes in a high yield without a significant change in ratio (5.4:1), indicating that the cyclization was not limited to primary halides.

The scope of substituted oxetanes was then expanded to a variety of sterically congested tetrasubstituted oxetanes using olefins as precursors (Scheme 5. 3). 2,2,4,4-Substituted derivative **12 a** was prepared from α-methylstyrene in three steps, including bromohydrin formation with NBS/H_2_O. We next investigated the 2,2,3,4-substituted derivatives from *trans*-stilbene; bromohydrin **10 b** was formed as a single diastereoisomer. This was converted to the corresponding 3,4-*anti*-substituted oxetane **12 b** through the same process, with stereospecific intramolecular displacement from benzylic bromide **11 b**. We then considered cyclic alkenes to access fused oxetane derivatives. Treating cyclohexene with NBS/H_2_O afforded the *anti*-substitution in bromohydrin **10 c**, as required for the proposed cyclization. Installation of the malonate group occurred effectively to provide **11 c**, and cyclization proceeded without incident to afford the oxabicyclo[4.2.0]octane derivative **12 c** in an excellent yield. From cyclopentene, the fused [3.2.0] ring system of bicycle **12 d** could be effectively obtained. Moreover, from 2,5-dihydrofuran, dioxabicyclo[3.2.0]heptane **12 e** was readily prepared in excellent yields. These oxetane-containing bicycles, readily accessible from simple alkenes in three steps, may provide interesting rigid motifs for medicinal chemistry.

**Scheme 3:**
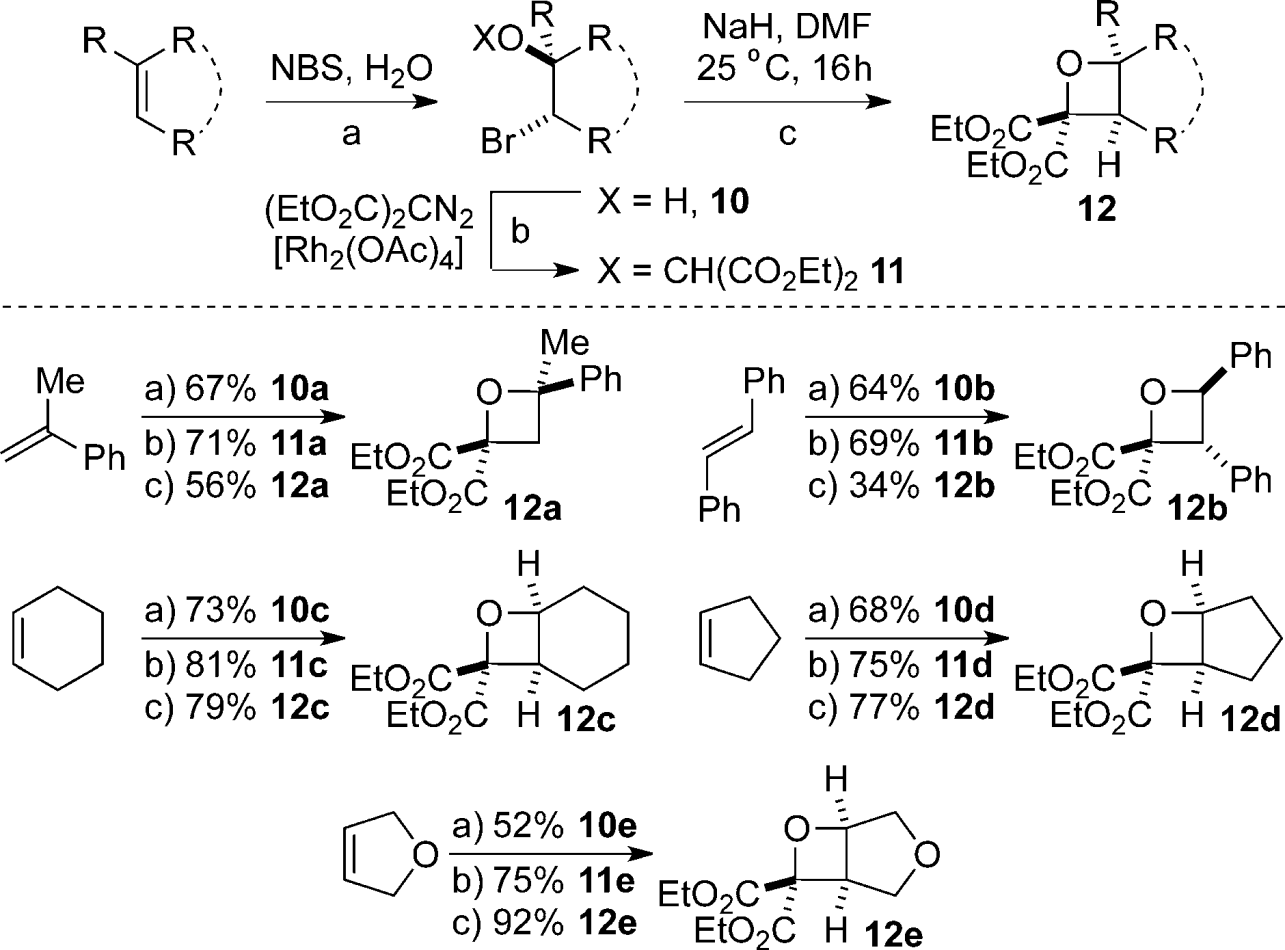
Synthesis of tetrasubstituted oxetanes. a) Bromohydrin formation. b) O=H insertion. c) Cyclization.

Elaboration of the oxetane products was then examined toward oxetane-containing fragments and building blocks. Initial investigations into the derivatization of the diester functionality were undertaken with oxetane **6 d** (Scheme 4). The diester **6 d** could be reduced using LiBH_4_, generated in situ, to give diol **13** in 91 % yield (Scheme 4). From this diol, monotosylation gave **14**, then treatment with NaH afforded the unusual bisoxetane spirocycle **15** through classical oxetane cyclization. Monohydrolysis of the diester moiety occurred quantitatively when treated with 1 m NaOH, affording the monocarboxylate sodium salt **16**. This compound successfully underwent amide coupling with both primary (**17**) and secondary (**18**) amines using HATU.[25,26] Krapcho decarboxylation using LiCl afforded *trans*- and *cis*-2,4-substituted oxetanes in a 1:1 ratio (**19 a**:**19 b**), which were readily separable.[22]

**Scheme 4:**
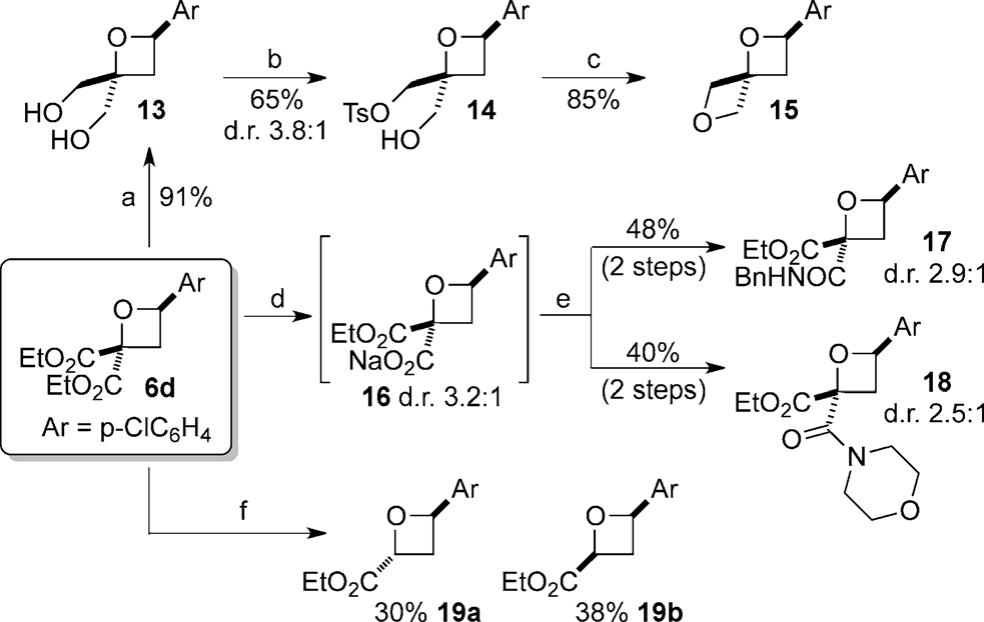
Derivatization of oxetanes 6 d. Conditions: a) NaBH_4_, LiCl, MeOH, THF, 0 °C→RT; b) *n*BuLi, THF, 0 °C, 1 h; then TsCl, THF, 30 °C; c) NaH, DMF, 0 °C→25 °C; d) NaOH, EtOH, 30 °C; e) amine (BnNH_2_ for 17; morpholine for 18), HATU, DMF, 40 °C; f) LiCl, DMSO, 150 °C. HATU=*N*-[(dimethylamino)-1*H*-1,2,3-triazolo[4,5-*b*]-pyridin-l-ylmethylene]-*N*-methylmethanaminium hexafluorophosphate *N*-oxide, DMSO=dimethyl sulfoxide.

Finally, bromomethyloxetane **9 c** was treated with nitrogen nucleophiles, resulting in the successful displacement of the bromide without affecting the oxetane ring (Scheme 5). Reaction with NaN_3_ afforded azide **20** in 92 % yield, which underwent cycloaddition with phenyl acetylene to afford triazole **21**. Treatment of **9 c** with imidazole and NaI under basic conditions displaced the primary bromide, thus affording **22** in a 62 % yield.

**Scheme 5:**
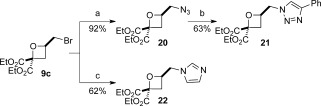
Bromide displacement from oxetane 9 c. a) NaN_3_, DMF, 60 °C; b) phenylacetylene, CuSO_4_⋅5 H_2_O (10 mol %), sodium ascorbate (30 mol %), H_2_O/CH_2_Cl_2_ (1:1), RT; c) imidazole, NaI, K_2_CO_3_, DMF, 80 °C.

In summary, we have developed an efficient protocol for the preparation of 2,2-disubstituted, 2,2,4-trisubstituted, and 2,2,3,4-tetrasubstitued oxetanes. These oxetane structures contain functionalities that can be further derivatized to an array of oxetane containing motifs. We anticipate that these will provide interesting new structural elements for synthesis, materials science, and medicinal chemistry programs in particular. Further advances of this strategy in the synthesis of small rings will be reported in due course.
