# Supplementary material for: Synthesis of Di-, Tri-, and Tetrasubstituted Oxetanes by Rhodium-Catalyzed O=H Insertion and C=C Bond-Forming Cyclization
Source: Angew Chem Int Ed Engl. 2014 Oct 14;53(51):14230–4. doi: 10.1002/anie.201408928 (PMC4502968; doi:10.1002/anie.201408928)
Supplement: Supplementary file 1 [file anie0053-14230-sd1.pdf]

Angewandte Chemie
Eine Zeitschrift der Gesellschaft Deutscher Chemiker

Supporting Information

© Wiley-VCH 2014

69451 Weinheim, Germany

Synthesis of Di-, Tri-, and Tetrasubstituted Oxetanes by Rhodium-Catalyzed O–H Insertion and C–C Bond-Forming Cyclization**

*Owen A. Davis and James A. Bull**

anie_201408928_sm_miscellaneous_information.pdf

Table of Contents

Table of Contents S1
References: Complete references 8a, 8c–8e, 9b, 10a, 14a–14b and 15 .... S2
General Experimental Considerations S3
Optimization of Rh-Catalyzed O–H Insertion using Ethylene Glycol S4
Optimization of Rh-Catalyzed O–H Insertion using Alcohols Incorporating a Leaving Group S5
Optimization of C–C Bond Forming Cyclization using Tosylate **2b** S6
Optimization of C–C Bond Forming Cyclization using Halides **2c*,* 2d** S7
Reoptimization of C–C Bond Forming Cyclization for 2,2,4-Trisubstituted Oxetanes S8
Synthesis of β-Halohydrins **4a–4g**, **7a–7g** S9
Synthesis of 2,2-Diethyl Oxetane-2,2-dicarboxylate **3** S17
Synthesis of Trisubstituted Oxetanes **6a–6h**, **9a–9i** S20
Synthesis of Tetrasubstituted Oxetanes **12a–12e** S38
Functionalization of Oxetanes **6d** and **9c** S45
^{1}H and ^{13}C NMR spectra of selected compounds S51
References S132

References: Complete references 8a, 8c–8e, 9b, 10a, 14a–14b and 15 are given below:

[8] a) J. Du, B.-K. Chun, R. T. Mosley, S. Bansal, H. Bao, C. Espiritu, A. M. Lam, E. Murakami, C. Niu, H. M. Micolochick Steuer, P. A. Furman, M. J. Sofia, *J. Med. Chem.* **2014**, *57*, 1826.

[8] c) T. H. M. Jonckers, K. Vandyck, L. Vandekerckhove, L. Hu, A. Tahri, S. Van Hoof, T.-I. Lin, L. Vijgen, J. M. Berke, S. Lachau-Durand, B. Stoops, L. Leclercq, G. Fanning, B. Samuelsson, M. Nilsson, Å. Rosenquist, K. Simmen, P. Raboisson, *J. Med. Chem.* **2014**, *57*, 1836.

[8] d) A. A. Estrada, B. K. Chan, C. Baker-Glenn, A. Beresford, D. J. Burdick, M. Chambers, H. Chen, S. L. Dominguez, J. Dotson, J. Drummond, M. Flagella, R. Fuji, A. Gill, J. Halladay, S. F. Harris, T. P. Heffron, T. Kleinheinz, D. W. Lee, C. E. Le Pichon, X. Liu, J. P. Lyssikatos, A. D. Medhurst, J. G. Moffat, K. Nash, K. Scearce-Levie, Z. Sheng, D. G. Shore, S. Wong, S. Zhang, X. Zhang, H. Zhu, Z. K. Sweeney, *J. Med. Chem.* **2014**, *57*, 921.

[8] e) J. E. Dowling, M. Alimzhanov, L. Bao, M. H. Block, C. Chuaqui, E. L. Cooke, C. R. Denz, A. Hird, S. Huang, N. A. Larsen, B. Peng, T. W. Pontz, C. Rivard-Costa, J. C. Saeh, K. Thakur, Q. Ye, T. Zhang, P. D. Lyne, *ACS Med. Chem. Lett.* **2013**, *4*, 800.

[9] b) G. Wuitschik, M. Rogers-Evans, A. Buckl, M. Bernasconi, M. Märki, T. Godel, H. Fischer, B. Wagner, I. Parrilla, F. Schuler, J. Schneider, A. Alker, W. B. Schweizer, K. Müller, E. M. Carreira, *Angew. Chem. Int. Ed.* **2008**, *47*, 4512; *Angew. Chem.* **2008**, *120*, 4588.

[10] a) A. F. Stepan, K. Karki, S. McDonald, P. H. Dorff, J. K. Dutra, K. J. DiRico, A. Won, C. Subramanyam, I. V. Efremov, C. J. O'Donnell, C. E. Nolan, S. L. Becker, L. R. Pustilnik, B. Sneed, H. Sun, Y. Lu, A. E. Robshaw, D. Riddell, T. J. O'Sullivan, E. Sibley, S. Capetta, K. Atchison, A. J. Hallgren, E. Miller, A. Wood, R. S. Obach, *J. Med. Chem.* **2011**, *54*, 7772.

[14] a) K. Kinoshita, T. Kobayashi, K. Asoh, N. Furuichi, T. Ito, H. Kawada, S. Hara, J. Ohwada, K. Hattori, T. Miyagi, W.-S. Hong, M.-J. Park, K. Takanashi, T. Tsukaguchi, H. Sakamoto, T. Tsukuda, N. Oikawa, *J. Med. Chem.* **2011**, *54*, 6286.

[14] b) A. A. Estrada, D. G. Shore, E. Blackwood, Y.-H. Chen, G. Deshmukh, X. Ding, A. G. DiPasquale, J. A. Epler, L. S. Friedman, M. F. T. Koehler, L. Liu, S. Malek, J. Nonomiya, D. F. Ortwine, Z. Pei, S. Sideris, F. St-Jean, L. Trinh, T. Truong, J. P. Lyssikatos, *J. Med. Chem.* **2013**, *56*, 3090.

[15] M. A. J. Duncton, M. A. Estiarte, D. Tan, C. Kaub, D. J. R. O'Mahony, R. J. Johnson, M. Cox, W. T. Edwards, M. Wan, J. Kincaid, M. G. Kelly, *Org. Lett.* **2008**, *10*, 3259.

General Experimental Considerations

All nonaqueous reactions were run under an inert atmosphere (argon) with flame-dried glassware using anhydrous solvents using standard techniques.

Anhydrous solvents were obtained by filtration through drying columns (THF, DMF, diethyl ether, CH_2Cl_2, toluene) or used as supplied (DMF, ethylene glycol).

Flash column chromatography was performed using 230-400 mesh silica with the indicated solvent system according to standard techniques. Analytical thin-layer chromatography (TLC) was performed on precoated, glass-backed silica gel plates. Visualization of the developed chromatogram was performed by UV absorbance (254 nm), aqueous potassium permanganate stain, PMA (phosphomolybdic acid) or vanillin.

Infrared spectra (ν_{max}, FTIR ATR) were recorded in reciprocal centimeters (cm^{-1}). Nuclear magnetic resonance spectra were recorded on either 400 MHz or 500 MHz spectrometers. Chemical shifts for ^1H NMR spectra are recorded in parts per million from tetramethylsilane with the solvent resonance as the internal standard (chloroform: δ = 7.27 ppm, DMSO: δ = 2.50 ppm). Data is reported as follows: chemical shift [multiplicity (s = singlet, d = doublet, t = triplet, m = multiplet and br = broad), coupling constant in Hz, integration, assignment]. ^{13}C NMR spectra were recorded with complete proton decoupling. Chemical shifts are reported in parts per million from tetramethylsilane with the solvent resonance as the internal standard ($^{13}CDCl_3$: 77.0 ppm, $(^{13}CD_3)_2SO$: 39.5 ppm). ^{19}F NMR spectra were recorded with complete proton decoupling. Chemical shifts are reported in parts per million referenced to the standard monofluorobenzene: −113.5 ppm. *J* values are reported in Hz. Assignments of ^1H/^{13}C spectra were made by the analysis of δ/*J* values, and COSY, DEPT-135, HSQC, and HMBC experiments as appropriate. Melting points are uncorrected.

Reagents: Commercial reagents were used as supplied or purified by standard techniques where necessary.

Optimization of Rh-Catalyzed O–H Insertion using Ethylene Glycol

Selected optimization of reaction conditions (**Table S1**).

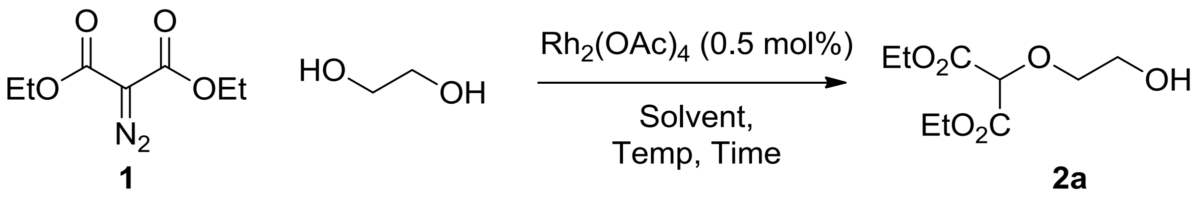

Entry[a]	Solvent (Concn, M)	Time (h)	Temp (°C)	Equiv of Ethylene Glycol	Yield **2a** (%)[b]
1[c]	Ethylene Glycol (0.1)	6 d	rt to 40	N/A	56
2	Ethylene Glycol (0.1)	2	80	N/A	52
3	Toluene (0.1)	2	80	10	65
4	Toluene (0.1)	2	80	20	63
**5**	**Benzene (0.1)**	**2**	**80**	**10**	**70 (77)**[d]

[a]1.0 mmol diazo malonate **1**. [b]Isolated yield. [c]Stirred at rt for 3 d followed by 40 °C for 3 d. [d]10 mmol diazo malonate **1**.

Initially, a 56% isolated yield was obtained for the Rh-catalyzed O–H insertion with ethylene glycol after being stirred at rt for 3 d followed by 40 °C for 3 d (entry 1). Increasing the temperature to 80 °C (entry 2) shortened the reaction time dramatically and a comparable yield was obtained after only 2 h. Switching the solvent to toluene (entry 3) increased the yield to 65%, however doubling the equivalents of ethylene glycol (entry 4) did not improve the yield. Finally, the yield was increased further by using benzene as the solvent (entry 5) and an isolated yield of 77% was obtained on a 10 mmol scale.

Optimization of Rh-Catalyzed O–H Insertion using Alcohols Incorporating a Leaving Group
Selected optimization of reaction conditions (**Table S2**).

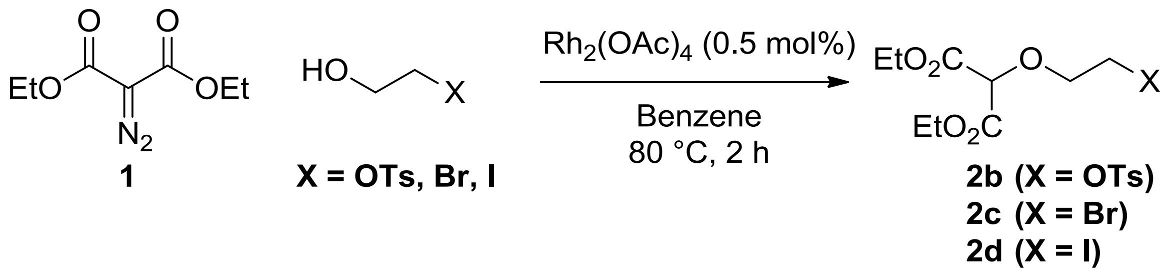

Entry[a]	X =	Equiv of Diazo Malonate **1**	Equiv of Alcohol	Yield (%)[b]
1	OTs	1.0	1.1	46
2	Br	1.0	1.1	64
3	I	1.0	1.1	54
4[c]	OTs	1.5	1.0	57
**5**	**Br**	**1.5**	**1.0**	**90**
6[c]	I	1.5	1.0	69

[a]1.0 mmol alcohol. [b]Isolated yield. [c]3.0 mmol alcohol.

The yield of the Rh-catalyzed O–H insertion using 2-[(4-Methylbenzenesulfonyl)oxy]ethan-1-ol, 2-bromoethanol and 2-iodoethanol initially gave low yields (entries 1–3), with 2-bromoethanol giving the highest yield of 64% (entry 3). The yields were increased when the alcohol was made the limiting reagent and the equiv of diazo malonate **1** was increased to 1.5 (entries 4–6). Once again 2-bromoethanol gave the highest yield of 90% (entry 5). It is important to note that while iodide **2d** can be isolated, it readily decomposes.

Optimization of C–C Bond Forming Cyclization using Tosylate 2b

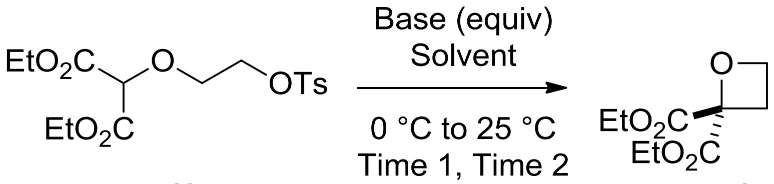

2b **3**

The following reaction variables were screened during optimization (**Table S3**).

Solvent	DMF; THF; Toluene; Acetonitrile; CH_2Cl_2
Base	NaH; NaOH; KOH; KOtBu; NaOtBu; LiOtBu; NaOEt; KOEt; KHMDS; DBU
Equiv of Base	2.0; 1.8; 1.5; 1.2
Concn (M)	0.025
Temp (°C)	0; 25
Time (h)	2 h 30 min; 3; 6; 19

Selected optimization of reaction conditions (**Table S4**).

Entry[a]	Solvent	Base (equiv)	Time 1 (h)	Time 2 (h)	Yield **3** (%)[b]	Remaining **2b** (%)[b]
**1**	**DMF**	**NaH (1.8)**	**1**	**90 min**	**65 (43)**	**0**
2	THF	NaH (1.8)	1	2	45	39
3	THF	NaH (1.8)	1	18	61	17
4	THF	KOtBu (1.8)	1	2	56	6
5	DMF	NaH (1.8)	1	2	63	0
**6**	**DMF**	**NaH (1.5)**	**1**	**2**	**65**	**6**
7	DMF	NaH (1.2)	1	2	48	14
8	DMF	NaH (1.5)	6	-	21	36
9	DMF	DBU (1.5)	-	6	26	42

[a]0.2 mmol tosylate **2b**. [b]Yield determined by ^{1}H NMR with respect to an internal standard (1,3,5-trimethoxybenzene). Isolated yield in parentheses.

The initial attempt at cyclisation (entry 1) afforded oxetane **3** with a promising isolated yield of 43%. However, none of tosylate **2b** was recovered. Conducting the reaction in THF (entry 2) lowered the yield but return of starting material was observed, which showed promise for optimization. Increasing the reaction time to 18 h (entry 3) gave a yield comparable to that with NaH in DMF (entry 1). However, when KOtBu was used as the base instead (entry 4), the reaction time was once again reduced. Extensive optimization using THF as the solvent was conducted, but unfortunately the yield could not be improved upon the initial result. Therefore investigations focused on optimizing the intial DMF/NaH conditions. Slightly lowering the equiv of NaH to 1.5 did not affect the yield (entry 6), but lowering it to 1.2 equiv reduced the yield significantly (entry 7). A slightly elevated temperature was required (entry 8) as a low yield was obtained when the reaction was left at 0 °C. Finally, DBU was used as the base for cyclization (entry 9).[1] However a disappointing yield of 26% was obtained. Despite all of the optimization conducted, none of the conditions tested improved upon the initial result.

Therefore alternative leaving groups for the C-C bond forming cyclization were examined.

Optimization of C–C Bond Forming Cyclization using Halides 2c, 2d

Selected optimization of reaction conditions (**Table S5**).

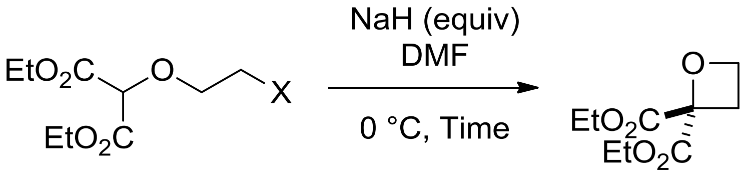

2c (X = Br)
2d (X=I)
3

Entry[a]	X =	Concn (M)	Equiv of NaH	Time (h)	Yield **3** (%)[b]
1	Br	0.025	1.5	1	79
2	Br	0.025	1.5	2	72
3	Br	0.025	1.8	1	80
**4**	**Br**	**0.025**	**1.2**	**1**	**90 (79)**
5	Br	0.025	1.2	20 min	73
6	Br	0.025	1.2	40 min	80
7	Br	0.05	1.2	1	79
8	Br	0.0125	1.2	1	83
**9**[c]	**Br**	**0.025**	**1.2**	**1**	**(73)**
10[c]	I	0.025	1.2	1	(31)

[a]0.2 mmol halide **2c**. [b]Yield determined by ^{1}H NMR with respect to an internal standard (1,3,5-trimethoxybenzene). Isolated yield in parentheses. [c]1.0 mmol halide **2c, 2d**.

Bromide **2c** was subjected to the best cyclization conditions from the previous optimization (entry 1). Pleasingly the desired oxetane **3** was obtained in a higher yield (79%) than tosylate **2b** (65%, **Table S4**, entry 6). Doubling the reaction time did not improve the yield (entry 2). Increasing the equiv of NaH did not affect the formation of oxetane **3** (entry 3), but lowering the equiv (entry 4) gave a yield of 90% with an isolated yield of 79%. Shortening the reaction time (entries 5 and 6) showed less of oxetane **3** being formed. Both increasing and decreasing the concentration of the reaction (entries 7 and 8) slightly lowered the yield. The reaction was conducted on a 1.0 mmol scale (entry 9) and a desirable 73% isolated yield was obtained. Finally, iodide **2d** was subjected to the optimized cyclization conditions and a disappointing 31% isolated yield was obtained. This is most likely due to degradation of iodide **2d** during the reaction.

Reoptimization of C–C Bond Forming Cyclization for 2,2,4-Trisubstituted Oxetanes
Reoptimization was conducted using bromide **5b** (**Table S6**).

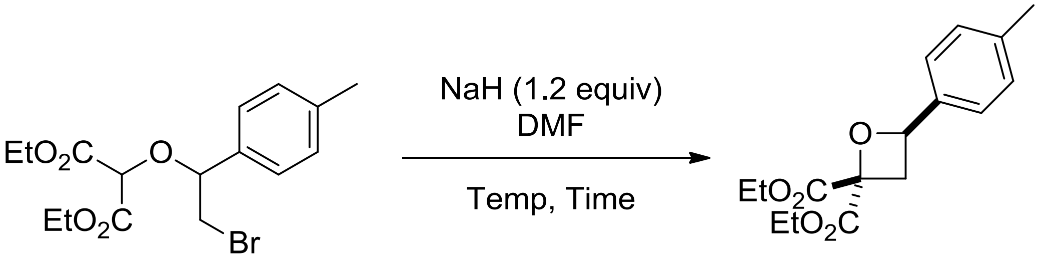

5b **6b**

Entry[a]	Temp (°C)	Time (h)	Yield (%)
1	0	1	10[b]
2	25	8	74
**3**	**25**	**16**	**77**
4	25	24	62

[a]0.5 mmol bromide **5b**. [b]Conversion determined by ^{1}H NMR.

Attempting the cyclization of bromide **5b** with the optimized conditions for oxetane **6b** (See **Table S6**), a disappointing 10% conversion was observed (entry 1). Increasing the temperature of the reaction to 25 °C and increasing the reaction time to 8 h (entry 2) afforded oxetane **6b** on a comparable yield to that previously optimized. Allowing the reaction to stir overnight for 16 h (entry 3) increased the yield very marginally, but a lower yield was observed when left for any longer (entry 4). As a result, the optimum conditions for the synthesis of 2,2,4-trisubstituted oxetanes was to stir the reaction at 25 °C for 16 h (entry 3).

Synthesis of β-Halohydrins 4a–4g, 7a–7g

Synthesis of β-bromohydrins **4a, 4b, 4d and 4e** from styrene derivatives (**Table S7**).

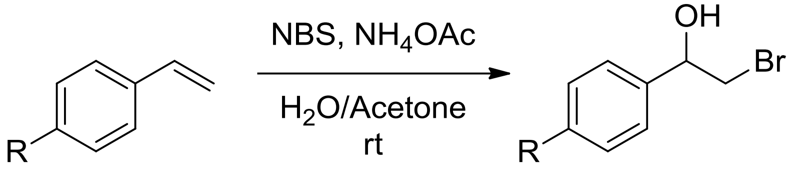

4a, 4b, 4d, 4e

Entry	R	Yield (%)
1	H	47 (**4a**)
2	Me	54 (**4b**)
3	Cl	46 (**4d**)
4	Br	47 (**4e**)

(±)-2-Bromo-1-phenylethan-1-ol (4a)

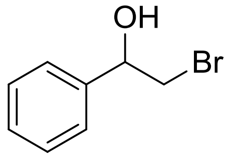

Ammonium acetate (237 mg, 3.1 mmol) was added to a solution of *N*-bromosuccinimide (4.26 g, 36.1 mmol) in acetone (15 mL) and water (15 mL). Styrene (1.72 mL, 15.0 mmol) was then added. The reaction mixture was stirred at rt for 24 h. Water (15 mL) was then added, followed by CH_2Cl_2 (30 mL). The layers were separated and the aqueous layer was extracted with CH_2Cl_2 (3 x 30 mL). The organic extracts were combined, dried (Na_2SO_4) and concentrated *in vacuo*. Purification by flash chromatography (10% EtOAc in hexanes) afforded alcohol **4a** as a pale yellow oil (1.41 g, 47%); R_f = 0.25 (20% EtOAc in hexanes); IR (film) / cm^{-1} 3379 (br O-H), 3031, 2963, 1493, 1454, 1420, 1217, 1198, 1060, 989, 764, 700, 593; ^{1}H NMR (400 MHz, $CDCl_3$) δ 7.44–7.29 (m, 5 H, 5 x Ph-H), 4.93 (ddd, *J* = 9.0, 3.4, 3.4 Hz, 1 H, C*H*(OH)), 3.65 (dd, *J* = 10.5, 3.4 Hz, 1 H, C*H*HBr), 3.56 (dd, *J* = 10.5, 9.0 Hz, 1 H, CH*H*Br), 2.74 (d, *J* = 3.4 Hz, 1 H, OH); ^{13}C NMR (101 MHz, $CDCl_3$) δ 140.2 (Ph-C_q), 128.6 (2 x Ph-CH), 128.4 (Ph-CH), 125.9 (2 x Ph-CH), 73.8 (CH(OH)), 40.2 (CH_2Br).

Observed data (IR, ^{1}H, ^{13}C) was consistent with that previously reported.[2,3]

(±)-2-Bromo-1-(4-methylphenyl)ethan-1-ol (4b)

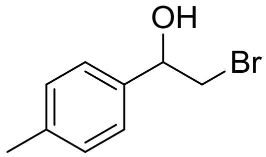

Ammonium acetate (192 mg, 2.5 mmol) was added to a solution of *N*-bromosuccinimide (3.52 g, 30.0 mmol) in acetone (25 mL) and water (25 mL). 4-Methylstyrene (3.29 mL, 25.0 mmol) was then added, after which, the solution turned from orange to cloudy white. The reaction mixture was stirred at rt for 27 h. Water (25 mL) was then added, followed by CH_2Cl_2 (25 mL). The layers were separated and the aqueous layer was extracted with CH_2Cl_2 (2 x 25 mL). The organic extracts were combined, dried (Na_2SO_4) and concentrated *in vacuo*. Purification by flash chromatography (10% EtOAc in hexanes) afforded alcohol **4b** as a colourless oil which solidified to a white solid upon storage at –20°C (2.89 g, 54%); R_f = 0.41 (20% EtOAc in hexanes); mp = 33–35 °C; IR (film) / cm^{-1} 3391 (br O-H), 3025, 2958, 2921, 1614, 1515, 1421, 1218, 1198, 1067, 993, 818, 766, 723, 643, 574, 559; ^{1}H NMR (400 MHz, $CDCl_3$) δ 7.28 (d, *J* = 7.9 Hz, 2 H,

2 x Tol-H), 7.20 (d, *J* = 7.9 Hz, 2 H, 2 x Tol-H), 4.91 (ddd, *J* = 8.9, 3.4, 3.4 Hz, 1 H, C*H*(OH)), 3.63 (dd, *J* = 10.4, 3.4 Hz, 1 H, C*H*HBr), 3.55 (dd, *J* = 10.4, 8.9 Hz, 1 H, CH*H*Br), 2.62 (d, *J* = 3.2 Hz, 1 H, OH), 2.37 (s, 3 H, Tol-CH_3); ^{13}C NMR (101 MHz, $CDCl_3$) δ 138.3 (Tol-C_q-CH(OH)), 137.3 (Tol-C_q-Me), 129.3 (2 x Tol-CH), 125.9 (2 x Tol-CH), 73.7 (CH(OH)), 40.3 (CH_2Br), 21.2 (Ar-CH_3).

Observed data (IR, ^{1}H, ^{13}C) was consistent with that previously reported.[4,5]

(±)-2-Bromo-1-(4-chlorophenyl)ethan-1-ol (4d)

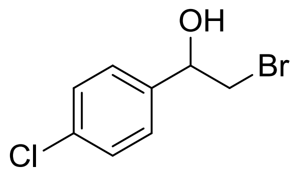

Ammonium acetate (472 mg, 6.0 mmol) was added to a solution of *N*-bromosuccinimide (8.50 g, 72.0 mmol) in acetone (30 mL) and water (30 mL). 4-Chlorostyrene (3.60 mL, 30.0 mmol) was then added. The reaction mixture was stirred at rt for 22 h. Water (30 mL) was then added, followed by CH_2Cl_2 (60 mL). The layers were separated and the aqueous layer was extracted with CH_2Cl_2 (3 x 60 mL). The organic extracts were combined, dried (Na_2SO_4) and concentrated *in vacuo*. Purification by flash chromatography (10% EtOAc in hexanes) afforded alcohol **4d** as a white solid (3.26 g, 46%); R_f = 0.15 (10% EtOAc in hexanes); mp = 60–61 °C (lit.[6] mp = 61–62 °C); IR (film) / cm^{-1} 3401 (br O-H), 2961, 2895, 1596, 1491, 1409, 1217, 1194, 1091, 1067, 1014, 991, 857, 831, 727, 688, 623, 546; ^{1}H NMR (400 MHz, $CDCl_3$) δ 7.37–7.34 (m, 2 H, 2 x Ar-H), 7.34–7.30 (m, 2 H, 2 x Ar-H), 4.90 (ddd, *J* = 8.7, 3.4, 3.4 Hz, 1 H, C*H*(OH)), 3.61 (dd, *J* = 10.5, 3.4 Hz, 1 H, C*H*HBr), 3.50 (dd, *J* = 10.5, 8.7 Hz, 1 H, CH*H*Br), 2.77 (d, *J* = 3.4 Hz, 1 H, OH); ^{13}C NMR (101 MHz, $CDCl_3$) δ 138.7 (Ar-C_q-CH(OH), 134.2 (Ar-C_q-Cl), 128.8 (2 x Ar-CH), 127.3 (2 x Ar-CH), 73.0 (CH(OH)), 39.8 (CH_2Br).

Observed data (IR, ^{1}H, ^{13}C) was consistent with that previously reported.[6,7,8]

(±)-2-Bromo-1-(4-bromophenyl)ethan-1-ol (4e)

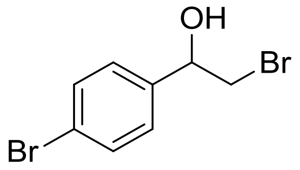

Ammonium acetate (227 mg, 3.0 mmol) was added to a solution of *N*-bromosuccinimide (4.28 g, 36.3 mmol) in acetone (15 mL) and water (15 mL). 4-Bromostyrene (1.96 mL, 15.0 mmol) was then added. The reaction mixture was stirred at rt for 24 h. Water (15 mL) was then added, followed by CH_2Cl_2 (30 mL). The layers were separated and the aqueous layer was extracted with CH_2Cl_2 (3 x 30 mL). The organic extracts were combined, dried (Na_2SO_4) and concentrated *in vacuo*. Purification by flash chromatography (10%–20% EtOAc in hexanes) afforded alcohol **4e** as a white crystalline solid (1.96 g, 47%); R_f = 0.29 (20% EtOAc in hexanes); mp = 64–66 °C; IR (film) / cm^{-1} 3387 (br O-H), 2960, 2890, 1593, 1488, 1421, 1403, 1217, 1194, 1071, 1011, 992, 855, 828, 722, 680, 615, 541; ^{1}H NMR (400 MHz, $CDCl_3$) δ 7.54–7.50 (m, 2 H, 2 x Ar-H), 7.30–7.26 (m, 2 H, 2 x Ar-H), 4.91 (ddd, *J* = 8.8, 3.4, 3.4 Hz, 1 H, C*H*(OH)), 3.63 (dd, *J* = 10.5, 3.4 Hz, 1 H, C*H*HBr), 3.51 (dd, *J* = 10.5, 8.8 Hz, 1 H, CH*H*Br), 2.64 (d, *J* = 3.4 Hz, 1 H, OH); ^{13}C NMR (101 MHz, $CDCl_3$) δ 139.2 (Ar-C_q-CH(OH)), 131.8 (2 x Ar-CH), 127.7 (2 x Ar-CH), 122.4 (Ar-C_q-Br), 73.1 (CH(OH)), 39.9 (CH_2Br).

Observed data (IR, ^{1}H, ^{13}C) was consistent with that previously reported.[3,9]

Synthesis of β-bromohydrins **4c**, **4f** and **4g** from Ketones (**Table S8**).

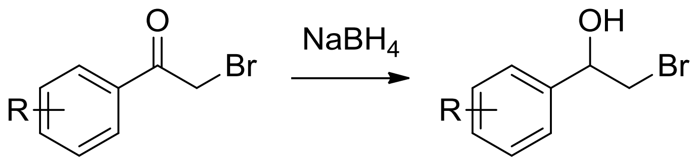

4c, 4f, 4g

Entry	R	Yield (%)
1	2-F	65 (**4c**)
2	3-OMe	65 (**4f**)
3	4-CN	70 (**4g**)

(±)-2-Bromo-1-(2-fluorophenyl)ethan-1-ol (4c)

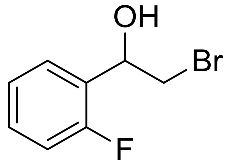

Sodium borohydride (205 mg, 5.4 mmol) was added portionwise to a solution of 2-bromo-2′-fluoroacetophenone (0.83 mL, 6.0 mmol) in MeOH (30 mL) at 0 °C. The reaction mixture was warmed to rt and stirred for 3 h. The reaction mixture was concentrated *in vacuo*, and water (20 mL) was added. The aqueous mixture was extracted with Et_2O (3 x 20 mL). The organic extracts were combined, washed with saturated aq. NH_4Cl (10 mL), brine (10 mL), dried (Na_2SO_4) and concentrated *in vacuo*. Purificaiton by flash chromatography (10% EtOAc in hexanes) afforded alcohol **4c** as a white solid (863 mg, 65%); R_f = 0.57 (30% EtOAc in hexanes); mp = 51–53 °C (lit.[10] mp = 52–53 °C); IR (film) / cm^{-1} 3400 (br O-H), 3068, 2965, 1617, 1587, 1487, 1455, 1421, 1230, 1181, 1066, 1032, 827, 795, 757, 659, 595; ^{1}H NMR (400 MHz, $CDCl_3$) δ 7.59–7.51 (m, 1 H, Ar-H), 7.36–7.28 (m, 1 H, Ar-H), 7.23–7.16 (m, 1 H, Ar-H), 7.10–7.02 (m, 1 H, Ar-H), 5.24 (ddd, *J* = 8.6, 4.2, 3.2 Hz, 1 H, C*H*(OH)), 3.75 (dd, *J* = 10.4, 3.2 Hz, 1 H, C*H*HBr), 3.56 (dd, *J* = 10.4, 8.6 Hz, 1 H, CH*H*Br), 2.73 (d, *J* = 4.2 Hz, 1 H, OH); ^{13}C NMR (101 MHz, $CDCl_3$) δ 159.6 (d, J_{CF} = 246 Hz, Ar-C_q-F), 129.8 (d, J_{CF} = 8 Hz, Ar-CH), 127.4 (d, J_{CF} = 4 Hz, Ar-CH), 127.3 (d, J_{CF} = 13 Hz, Ar-C_q-CH(OH)), 124.4 (d, J_{CF} = 3 Hz, Ar-CH), 115.4 (d, J_{CF} = 22 Hz, Ar-CH), 67.9 (d, J_{CF} = 2 Hz, CH(OH)), 39.0 (CH_2Br); ^{19}F NMR (376 MHz, $CDCl_3$) δ –119.0.

Observed data (^{1}H, ^{13}C) was consistent with that previously reported.[11]

(±)-2-Bromo-1-(3-methoxyphenyl)ethan-1-ol (4f)[12]

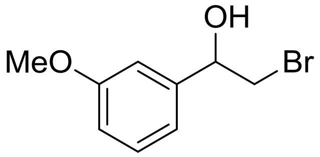

Sodium borohydride (157 mg, 4.2 mmol) in water (1.8 mL) was added to a solution of 2-bromo-3'-methoxyacetophenone (1.38 g, 6.0 mmol) in dioxane (6.0 mL). The mixture was stirred at rt for 3 h 30 min. The reaction mixture was neutralised with 1 M H_2SO_4, diluted with water (10 mL) and the aqueous mixture extracted with Et_2O (4 x 15 mL). The organic extracts were combined, dried (Na_2SO_4) and concentrated *in vacuo*. Purification by flash chromatography (20% EtOAc in hexanes) afforded alcohol **4f** as a colourless oil (901 mg, 65%); R_f = 0.27 (20% EtOAc in hexanes); IR (film) / cm^{-1} 3403 (br O-H), 2961, 2836, 1600, 1586, 1488, 1455, 1434, 1319, 1255, 1216, 1159, 1141, 1065, 1039, 994, 870, 785, 753, 700, 689, 570, 551; ^{1}H NMR (400 MHz, $CDCl_3$) δ 7.31–7.25 (m, 1 H, Ar-H), 6.97–6.91 (m, 2 H, 2 x Ar-H),

6.89–6.83 (m, 1 H, Ar-H), 4.88 (d, *J* = 7.8 Hz, 1 H, C*H*(OH)), 3.81 (s, 3 H, OCH_3), 3.62 (dd, *J* = 10.4, 3.4 Hz, 1 H, C*H*HBr), 3.52 (dd, *J* = 10.4, 9.0 Hz, 1 H, CH*H*Br), 2.85 (br s, 1 H, OH); ^{13}C NMR (101 MHz, $CDCl_3$) δ 159.7 (Ar-C_q-OMe), 141.9 (Ar-C_q-CH(OH)), 129.6 (Ar-CH), 118.2 (Ar-CH), 113.9 (Ar-CH), 111.4 (Ar-CH), 73.6 (CH(OH)), 55.2 (OCH_3), 40.0 (CH_2Br).

Observed data (^1H) was consistent with that previously reported.[13]

(±)-4-(2-Bromo-1-hydroxyethyl)benzonitrile (4g)[14]

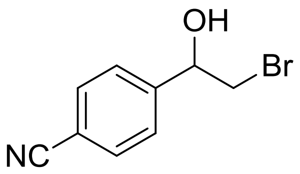

Sodium borohydride (160 mg, 4.2 mmol) was added portionwise to a solution of 2-bromo-4′-cyanoacetophenone (1.34 g, 6.0 mmol) in MeOH at 0 °C. The reaction mixture was warmed to rt and stirred for 90 min. The reaction mixture was concentrated *in vacuo*, and water (20 mL) was added. The aqueous mixture was extracted with Et_2O (3 x 20 mL). The organic extracts were combined, washed with saturated aq. NH_4Cl (10 mL), brine (10 mL), dried (Na_2SO_4) and concentrated *in vacuo*. Purificaiton by flash chromatography (20%–30% EtOAc in hexanes) afforded alcohol **4g** as a colourless oil (950 mg, 70%); R_f = 0.23 (30% EtOAc in hexanes); IR (film) / cm^{-1} 3448 (br O-H), 2962, 2229 (C≡N), 1609, 1503, 1410, 1219, 1197, 1068, 1018, 992, 838, 757, 681, 636, 556, 531; ^1H NMR (400 MHz, $CDCl_3$) δ 7.67 (d, *J* = 8.2 Hz, 2 H, 2 x Ar-H), 7.53 (d, *J* = 8.2 Hz, 2 H, 2 x Ar-H), 5.00 (ddd, *J* = 8.4, 3.6, 3.5 Hz, 1 H, C*H*(OH)), 3.65 (dd, *J* = 10.6, 3.5 Hz, 1 H, C*H*HBr), 3.52 (dd, *J* = 10.6, 8.4 Hz, 1 H, CH*H*Br), 2.90 (d, *J* = 3.6 Hz, 1 H, OH); ^{13}C NMR (101 MHz, $CDCl_3$) δ 145.5 (Ar-C_q-CH(OH)), 132.5 (2 x Ar-CH), 126.8 (2 x Ar-CH), 118.5 (C≡N), 112.2 (Ar-C_q-CN), 72.81 (CH(OH)), 39.4 (CH_2Br).

Observed data (^1H, ^{13}C) was consistent with that previously reported.[14]

Synthesis of Enantioenriched β-bromohydrin **(*S*)-4a**

(1*S*)-2-bromo-1-phenylethan-1-ol ((*S*)-4a)[15]

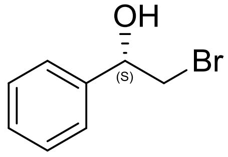

A solution of BH_3.THF (1 M in THF, 1.71 mL, 1.71 mmol) was added slowly to a cooled, stirring solution of L-valine (134 mg, 1.15 mmol) in THF (1.71 mL) at 0 °C. The mixture was stirred at 70 °C for 17 h. The mixture was further diluted with THF (4.0 mL). The mixture was cooled to 60 °C and $B(OMe)_3$ (56 mL, 0.50 mmol) was added and the mixture stirred at 60 °C for 1 h. The mixture was cooled to rt and $BH_3.SMe_2$ (0.46 mL, 4.85 mmol) was added over 1 h *via* syringe pump. After this time, 2-bromoacetophenone (797 mg, 4.00 mmol) in THF (3.56 mL) was added to the mixture over 1 h *via* syringe pump. The reaction was stirred at rt for a further 30 min, then cooled to 0 °C. Methanol (3 mL) and water (10 mL) were added and the mixture concentrated *in vacuo*. The remaining aqueous layer was extracted with CH_2Cl_2 (3 x 20 mL). The organic extracts were combined, dried (Na_2SO_4) and concentrated *in vacuo*. Purification by flash chromatography (20% EtOAc in hexanes) afforded alcohol **(*S*)-4a** as a colourless oil (717 mg, 85% ee, 89%); $[\alpha]_D^{21}$ +44.6° (c. 3.14, $CHCl_3$) (lit.[16] $[\alpha]_D^{20}$ +30.0° (c. 1.61, $CHCl_3$)); **ee = 85%** by HPLC, Chiralpak IB-3 column, 98:2 *n*hexane/IPA, flow 1.0 $mLmin^{-1}$, UV detection at 224 nm, 12.7 min (**(*S*)-4a**), 16.1 min (**(*R*)-4a**).

Observed data was identical to the racemic alcohol (±)-**4a**.

(1*S*)-2-Bromo-1-phenylethan-1-ol ((*S*)-4a)

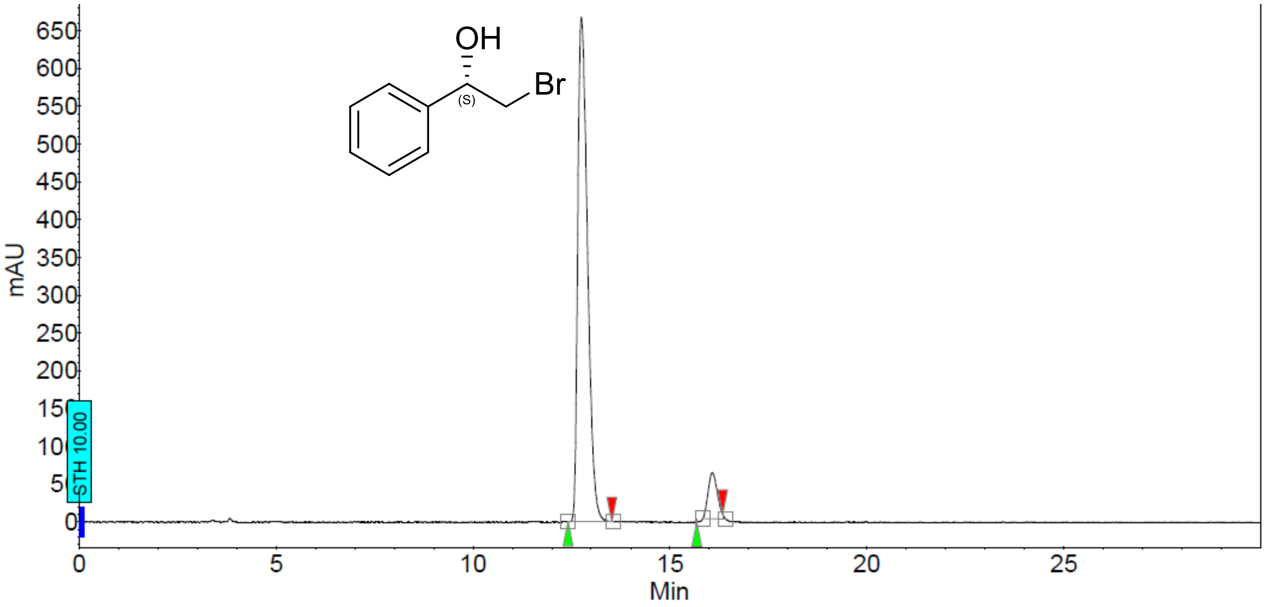

Chiralpak IB-3 column, 98:2 *n*hexane/IPA, flow 1.0 mLmin^{-1}, UV detection at 224 nm

Index	Name	Time [Min]	Quantity [% Area]	Height [mAU]	Area [mAU.Min]	Area % [%]
1	UNKNOWN	12.746	92.30	667.6	191.1	92.301
2	UNKNOWN	16.079	7.70	60.6	15.9	7.699
Total			100.00	728.2	207.1	100.000

ee = 85%

(±)-2-Bromo-1-phenylethan-1-ol ((±)-4a)

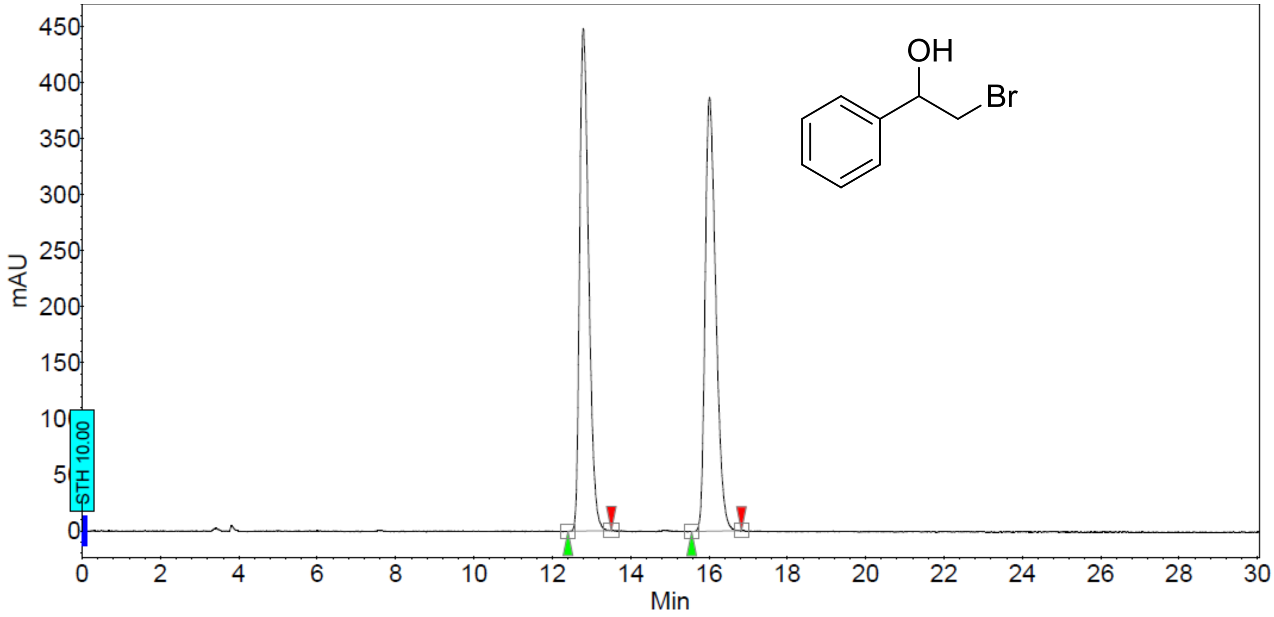

Chiralpak IB-3 column, 98:2 *n*hexane/IPA, flow 1.0 mLmin^{-1}, UV detection at 224 nm

Index	Name	Time [Min]	Quantity [% Area]	Height [mAU]	Area [mAU.Min]	Area % [%]
1	UNKNOWN	12.786	49.67	448.3	120.5	49.668
2	UNKNOWN	16.012	50.33	387.1	122.1	50.332
Total			100.00	835.4	242.5	100.000

Synthesis of β-halohydrins **7a–7f** from Epoxides (**Table S9**).

*Synthesis of β-halohydrins **7a–7f** was performed using 230-400 mesh silica.*

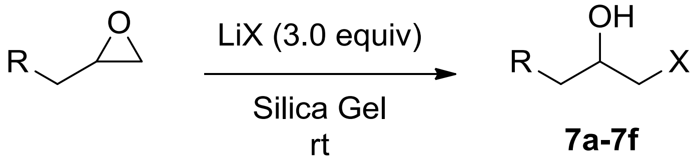

7a-7f

Entry	R	X	Yield (%)
1	OBn	Br	72 (**7a**)
2	OPh	Br	76 (**7b**)
3	Br	Br	75 (**7c**)
4	Cl	Br	73 (**7d**)
5[a]	Cl	Cl	71 (**7e**)
6[a]	OiPr	Cl	74 (**7f**)

[a]3.0 equiv H_2O added.

(±)-1-(Benzyloxy)-3-bromopropan-2-ol (7a)

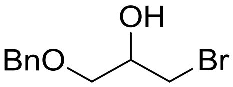

Benzyl glycidyl ether (0.91 mL, 6 mmol) was added to a slurry of lithium bromide (1.57 g, 18 mmol) and silica gel (1.71 g) in CH_2Cl_2 (10 mL). The solvent was removed under reduced pressure and the mixture was stirred at rt for 2 d. The solid was filtered, washed with CH_2Cl_2 (50 mL) and the filtrate was concentrated *in vacuo* to afford alcohol **7a** as a pale yellow oil (1.05 g, 72%); IR (film) / cm^{-1} 3336 (br O-H), 2863, 1454, 1362, 1216, 1101, 1028, 909, 836, 738, 699, 609, 574; ^{1}H NMR (400 MHz, $CDCl_3$) δ 7.40–7.30 (m, 5 H, 5 x Ph-H), 4.58 (s, 2 H, OCH_2Ph), 4.04–3.97 (m, 1 H, C*H*(OH)), 3.62 (dd *J* = 9.6, 5.3 Hz, 1 H, C*H*HOBn), 3.60 (dd, *J* = 9.6, 4.9 Hz, 1 H, CH*H*OBn), 3.55 (dd, *J* = 10.3, 5.3 Hz, 1 H, C*H*HBr), 3.49 (dd, *J* = 10.3, 5.7 Hz, 1 H, CH*H*Br), 2.64 (d, *J* = 5.7 Hz, 1 H, OH); ^{13}C NMR (101 MHz, $CDCl_3$) δ 137.5 (C_q-Ph), 128.5 (2 x Ph-CH), 127.9 (Ph-CH), 127.7 (2 x Ph-CH), 73.5 (O*C*H_2Ph), 71.4 (*C*H_2OBn), 69.9 (CH(OH)), 35.1 (CH_2Br).

Observed data (IR, ^{1}H, ^{13}C) was consistent with that previously reported.[17]

(±)-2-Bromo-1-phenylethan-1-ol (7b)

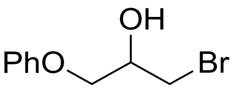

Phenyl glycidyl ether (2.0 mL, 15 mmol) was added to a slurry of lithium bromide (3.93 g, 45 mmol) and silica gel (4.15 g) in CH_2Cl_2 (20 mL). The solvent was removed under reduced pressure and the mixture was stirred at rt for 18 h. The mixture was loaded directly onto a silica column and purification by flash chromatography (20% Et_2O in hexanes) afforded alcohol **7b** as a pale yellow oil (2.57 g, 76%); R_f = 0.30 (20% EtOAc in hexanes); IR (film) / cm^{-1} 3404 (br O-H), 2930, 2878, 1598, 1588, 1494, 1457, 1423, 1291, 1239, 1172, 1108, 1078, 1042, 811, 751, 690, 527; ^{1}H NMR (400 MHz, $CDCl_3$) δ 7.34–7.29 (m, 2 H, 2 x Ph-H), 7.02–6.98 (m, 1 H, Ph-H), 6.95–6.92 (m, 2 H, 2 x Ph-H), 4.24–4.19 (m, 1 H, C*H*(OH)), 4.15–4.07 (m, 2 H, CH_2OPh), 3.68 (dd, *J* = 10.4, 5.2 Hz, 1 H, C*H*HBr), 3.61 (dd, *J* = 10.4, 5.4 Hz, 1 H, CH*H*Br), 2.51 (br s, 1 H, OH); ^{13}C NMR (101 MHz, $CDCl_3$) δ 156.0 (C_q-Ph), 129.4 (2 x Ph-CH), 121.3 (Ph-CH), 114.4 (2 x Ph-CH), 69.3 (CH(OH)), 69.0 (CH_2OPh), 34.8 (CH_2Br).

Observed data (IR, ^{1}H, ^{13}C) was consistent with that previously reported.[18,19]

(±)-1,3-Dibromopropan-2-ol (7c)

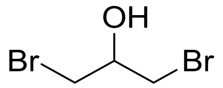

Epibromohydrin (1.28 mL, 15 mmol) was added to a slurry of lithium bromide (3.92 g, 45 mmol) and silica gel (4.44 g) in CH_2Cl_2 (20 mL). The solvent was removed under reduced pressure and the mixture was stirred at rt for 18 h. The solid was filtered, washed with CH_2Cl_2 (70 mL) and the filtrate and concentrated *in vacuo* to afford alcohol **7d** as a colourless oil (2.44 g, 75%); ^{1}H NMR (400 MHz, $CDCl_3$) δ 4.05–3.98 (m, 1 H, C*H*(OH)), 3.62 (dd, *J* = 10.5, 5.4 Hz, 2 H, 2 x C*H*HBr), 3.58 (dd, *J* = 10.5, 5.4 Hz, 2 H, CH*H*Br), 2.45 (d, *J* = 6.8 Hz, 1 H, OH).

Observed data (^{1}H) was consistent with that previously reported[20] and the compound is commercially available.[21]

(±)-1-Bromo-3-chloropropan-2-ol (7d)[22]

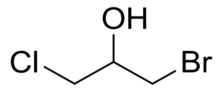

Epichlorohydrin (1.17 mL, 15 mmol) was added to a slurry of lithium bromide (3.92 g, 45 mmol) and silica gel (4.90 g) in CH_2Cl_2 (20 mL). The solvent was removed under reduced pressure and the mixture was stirred at rt for 24 h. The solid was filtered, washed with CH_2Cl_2 (70 mL) and the filtrate concentrated *in vacuo* to afford alcohol **7c** as a colourless oil (1.91 g, 73%); ^{1}H NMR (400 MHz, $CDCl_3$) δ 4.09–4.00 (m, 1 H, C*H*(OH)), 3.74 (dd, *J* = 11.3, 4.8 Hz, 1 H, C*H*HCl), 3.70 (dd, *J* = 11.3, 5.3 Hz, 1 H, CH*H*Cl) 3.61–3.58 (m, 2 H, CH_2Br), 2.46 (d, *J* = 7.0 Hz, 1 H, OH).

Observed data (^{1}H) was consistent with that previously reported[22] and the compound is commercially available.[23]

(±)-1,3-Dichloropropan-2-ol (7e)[22]

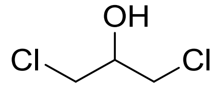

Epichlorohydrin (1.17 mL, 15 mmol) was added to a slurry of lithium chloride (1.92 g, 45 mmol) and silica gel (4.76 g) in CH_2Cl_2 (20 mL). The solvent was removed under reduced pressure and water (0.81 mL, 45 mmol) was added. The mixture was stirred at rt for 5 d. The solid was filtered, washed with CH_2Cl_2 (70 mL) and the filtrate concentrated *in vacuo* to afford alcohol **7e** as a colourless oil (1.37 g, 71%); ^{1}H NMR (400 MHz, $CDCl_3$) δ 4.14–4.00 (m, 1 H, C*H*(OH)), 3.70 (d, *J* = 5.3 Hz, 4 H, 2 x CH_2Cl), 2.68 (br s, 1 H, OH).

Observed data (^{1}H) was consistent with that previously reported[22] and the compound is commerically available.[24]

(±)-1-Chloro-3-(propan-2-yloxy)propan-2-ol (7f)

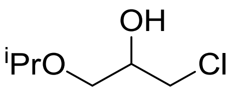

Glycidyl isopropyl ether (1.9 mL, 15 mmol) was added to a slurry of lithium chloride (1.92 g, 45 mmol) and silica gel (4.32 g) in CH_2Cl_2 (20 mL). The solvent was removed under reduced pressure and water (0.81 mL, 45 mmol) was added. The mixture was stirred at rt for 4 d. The solid was filtered, washed with CH_2Cl_2 (70 mL) and the filtrate was concentrated *in vacuo* to afford alcohol **7f** as a colourless oil (1.70 g, 74%); IR (film) / cm^{-1} 3419 (br O-H), 2973, 2928, 2873, 1470, 1430, 1370, 1336, 1175, 1127, 1088, 1066, 976, 927, 848, 823, 743, 707, 618; ^{1}H NMR (400 MHz, $CDCl_3$) δ 4.00–3.90 (m, 1 H, C*H*(OH)), 3.69–3.56 (m, 3 H, OC*H*$(CH_3)_2$ and CH_2O^iPr), 3.54 (d, *J* = 5.1 Hz, 2 H, CH_2Cl), 2.54 (d, *J* = 5.7 Hz, 1 H, OH), 1.18 (d, *J* = 6.1

Hz, 6 H, CH(C*H*$_3$)$_2$); ^{13}C NMR (101 MHz, $CDCl_3$) δ 72.3 (O*C*H(CH$_3$)$_2$), 70.4 (CH(OH)), 68.5 (CH$_2$O^{i}Pr), 45.9 (CH$_2$Cl), 22.0 (CH$_3$), 21.9 (CH$_3$).

Observed data (^{1}H, ^{13}C) was consistent with that previously reported.[25]

Synthesis of β-chlorohydrin **7g** from (±)-3-Chloro-1,2-propanediol.

(±)-1-[(*tert*-Butyldimethylsilyl)oxy]-3-chloropropan-2-ol (7g)[26]

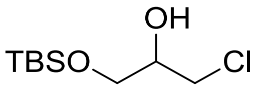

Imidazole (680 mg, 10.0 mmol) and 4-dimethylaminopyridine (301 mg, 2.5 mmol) was added to a solution of (±)-3-chloro-1,2-propanediol (0.84 mL, 10.0 mmol) in DMF (10 mL) and then cooled to 0 °C. *tert*-Butyldimethylsilyl chloride (1.51 g, 10.0 mmol) was added portionwise and the reaction mixture was stirred at rt for 15 h. The reaction mixture was diluted with H_2O (100 mL) and the aqueous mixture was extracted with CH_2Cl_2 (3 x 20 mL). The organic extracts were combined, washed with H_2O (10 mL), brine (10 mL), dried (Na_2SO_4) and concentrated *in vacuo*. Purification by flash chromatography (15% EtOAc in hexanes) afforded alcohol **7g** as a colourless oil (1.96 g, 87%); R_f = 0.48 (20% EtOAc in hexanes); IR (film) / cm^{-1} 3429 (br O-H), 2956, 2930, 2858, 1474, 1429, 1362, 1253, 1115 (C-O), 1048 (C-O), 1006, 969, 938, 834, 776, 739 (C-Cl), 667; ^{1}H NMR (400 MHz, $CDCl_3$) δ 3.89–3.81 (m, 1 H, C*H*(OH)), 3.74 (dd, *J* = 10.1, 4.6 Hz, 1 H, C*H*HOTBS), 3.69 (dd, *J* = 10.1, 5.0 Hz, 1 H, CH*H*OTBS), 3.62 (dd, *J* = 11.0, 5.9 Hz, 1 H, C*H*HCl), 3.58 (dd, *J* = 11.0, 5.6 Hz, 1 H, CH*H*Cl), 2.57 (d, *J* = 6.3 Hz, 1 H, OH), 0.91 (s, 9 H, C(C*H*$_3$)$_3$), 0.09 (s, 6 H, OSi(C*H*$_3$)$_2$(*t*Bu)). ^{13}C NMR (101 MHz, $CDCl_3$) δ 71.4 (CH(OH)), 63.4 (CH$_2$OTBS), 45.3 (CH$_2$Cl), 25.8 (C(*C*H$_3$)$_3$), 18.2 (*C*$_q$(CH$_3$)$_3$), –5.5 (OSi(*C*H$_3$)$_2$(*t*Bu).

Observed data (^{1}H) was consistent with that previously reported.[27]

Synthesis of 2,2-Diethyl Oxetane-2,2-dicarboxylate 3

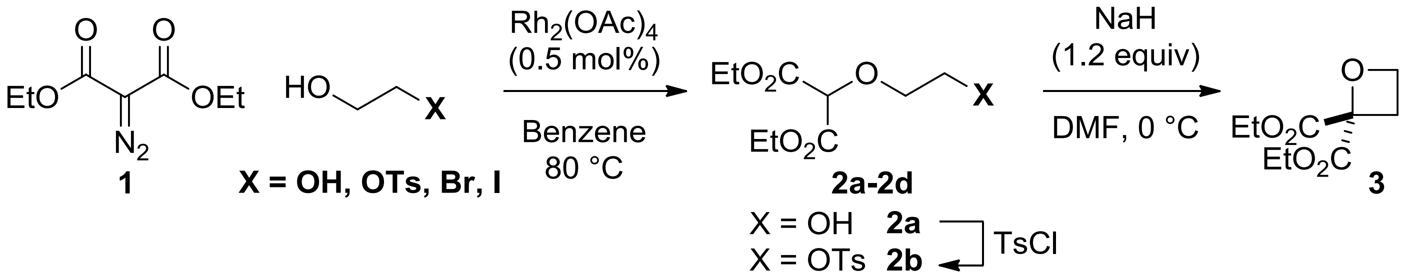

1,3-Diethyl 2-diazopropanedioate (1)[28]

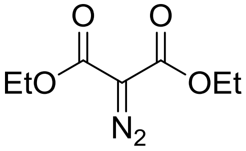

Sodium azide (2.05 g, 31.5 mmol) and 4-toluenesulfonyl chloride (6.00 g, 31.5 mmol) were dissolved in EtOH (63 mL) and H_2O (27 mL). The mixture was stirred at rt for 4 h. Diethyl malonate (4.55 mL, 30.0 mmol) and triethylamine (4.39 mL, 31.5 mmol) were then added. The mixture turned a yellow/orange colour after the triethylamine was added. The mixture was stirred at rt for 70 h. Water (300 mL) was added, and the aqueous layer was extracted with Et_2O (3 x 150 mL). The organic extracts were combined, washed with brine (150 mL), dried (Na_2SO_4) and concentrated *in vacuo*. Purification by flash chromatography (20% Et_2O in hexanes) afforded diazo malonate **1** as an orange liquid (5.14 g, 92%); R_f = 0.23 (25% Et_2O in hexanes); IR (film) / cm^{-1} 2984, 2133 (C=N=N out-of-phase),[29] 1757 (C=O), 1732 (C=O), 1687, 1395, 1371 (C=N=N in-phase),[29] 1266, 1072, 759; ^1H NMR (400 MHz, $CDCl_3$) δ 4.31 (q, *J* = 7.1 Hz, 4 H, 2 x $CH_3C\mathit{H_2}CO_2$), 1.32 (t, *J* = 7.1 Hz, 6 H, 2 x $C\mathit{H_3}CH_2CO_2$); ^{13}C NMR (101 MHz, $CDCl_3$) δ 161.1 (2 x CO_2), 61.6 (2 x $CO_2\mathit{C}H_2CH_3$), 14.3 (2 x $CO_2CH_2\mathit{C}H_3$); HRMS (ESI-TOF) *m/z* Calcd for $C_7H_{11}N_2O_4^+$ $[M+H]^+$: 187.0713, Found: 187.0711.

Observed data (IR, ^1H, ^{13}C) was consistent with that previously reported.[30]

1,3-Diethyl 2-(2-hydroxyethoxy)propanedioate (2a)

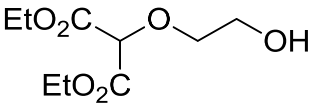

A mixture of diazo malonate **1** (1.86 g, 10.0 mmol), ethylene glycol (5.56 mL, 100 mmol) and dirhodium(II) tetraacetate (22 mg, 0.05 mmol) in benzene (100 mL) was heated under reflux (85 °C) for 2 h. The reaction mixture was allowed to cool to rt. Water (100 mL) was added, and the aqueous mixture was extracted with $CHCl_3$ (2 x 100 mL). The organic extracts were combined, dried (Na_2SO_4) and concentrated *in vacuo*. Purification by flash chromatography (50% EtOAc in hexanes) afforded alcohol **2a** as a colourless oil (1.70 g, 77%); R_f = 0.21 (50% EtOAc in hexanes); IR (film) / cm^{-1} 3476 (br O-H), 2984, 2940, 1736 (C=O), 1448, 1371, 1228, 1181, 1138, 1075, 1023, 888, 851, 600; ^1H NMR (400 MHz, $CDCl_3$) δ 4.53 (s, 1 H, $CH(CO_2Et)_2$), 4.33–4.21 (m, 4 H, 2 x $CH_3C\mathit{H_2}CO_2$), 3.80–3.72 (m, 4 H, $OC\mathit{H_2}C\mathit{H_2}OH$), 2.67 (br s, 1 H, OH), 1.29 (t, *J* = 7.1 Hz, 6 H, 2 x CH_3); ^{13}C NMR (101 MHz, $CDCl_3$) δ 166.6 (2 x CO_2), 79.2 ($(EtO_2C)_2\mathit{C}HO$), 73.1 (CH_2OH), 62.2 (2 x $CO_2\mathit{C}H_2CH_3$), 61.4 ($O\mathit{C}H_2CH_2OH$), 14.0 (2 x $CO_2CH_2\mathit{C}H_3$); HRMS (ESI-TOF) *m/z* Calcd for $C_9H_{16}NaO_6^+$ $[M+Na]^+$: 243.0845, Found: 243.0856.

1,3-Diethyl 2-{2-[(4-methylbenzenesulfonyl)oxy]ethoxy}propanedioate (2b)

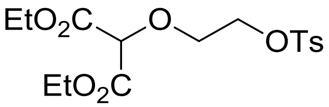

Method A – From alcohol **2a**:

Triethylamine (2.85 mL, 20.4 mmol) and trimethylamine hydrogen chloride (62.2 mg, 0.65 mmol) were added to a solution of alcohol **2a** (1.50 g, 6.8 mmol) in toluene (30 mL). The mixture was stirred at 0 °C for 10 min. 4-Toluenesulfonyl chloride (2.60 g, 13.6 mmol) was added in one portion and the resulting mixture was stirred at 0 °C for 15 min and then was warmed to rt and stirred for a further 2 h. Water (60 mL) was added followed by EtOAc (30 mL). The layers were separated and the aqueous layer was extracted with EtOAc (2 x 60 mL). The organic extracts were combined, washed with brine (60 mL), dried (Na_2SO_4) and concentrated *in vacuo*. Purification by flash chromatography (20%–40% EtOAc in hexanes) afforded tosylate **2b** as a colourless oil (2.42 g, 95%); R_f = 0.09 (20% EtOAc in hexanes); IR (film) / cm^{-1} 2985, 1740 (C=O), 1598, 1448, 1358, 1232, 1175, 1096, 1019, 916, 816, 773, 731, 662, 577, 553; ^1H NMR (400 MHz, $CDCl_3$) δ 7.84–7.79 (m, 2 H, 2 x Ts-H), 7.37–7.33 (m, 2 H, 2 x Ts-H), 4.52 (s, 1 H, $CH(CO_2Et)_2$), 4.32–4.20 (m, 6 H, 2 x $CO_2CH_2CH_3$ and CH_2OTs), 3.91–3.86 (m, 2 H, OCH_2CH_2OTs), 2.46 (s, 3 H, Ts-CH_3), 1.30 (t, *J* = 7.1 Hz, 6 H, 2 x $CO_2CH_3CH_3$); ^{13}C NMR (101 MHz, $CDCl_3$) δ 166.0 (2 x CO_2), 144.9 (Ts-C_q-SO_2), 132.7 (Ts-C_q-Me), 129.8 (2 x Ts-CH), 128.0 (2 x Ts-CH), 79.2 ($(EtO_2C)_2CHO$), 68.6 (CH_2OTs), 68.4 (OCH_2CH_2OTs), 62.1 (2 x $CO_2CH_2CH_3$), 21.6 (Ts-CH_3), 14.0 (2 x $CO_2CH_2CH_3$); HRMS (ESI-TOF) *m/z* Calcd for $C_{16}H_{23}O_8S^+$ $[M+H]^+$: 375.1108, Found: 375.1115.

Method B – From diazo malonate **1**:

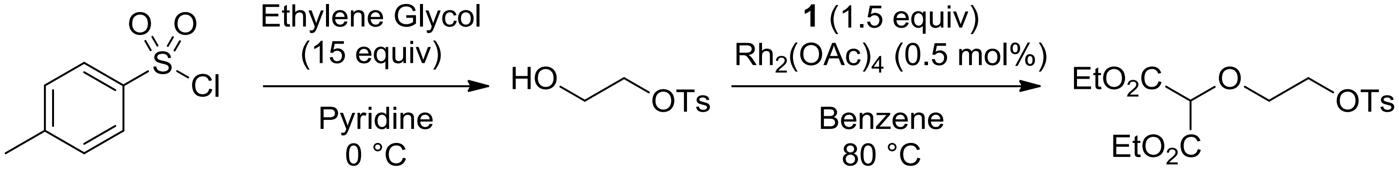

2-[(4-Methylbenzenesulfonyl)oxy]ethan-1-ol was prepared according to the procedure of Bradshaw and co-workers.[31] 4-Toluenesulfonyl chloride (1.90 g, 10.0 mmol) in pyridine (9.53 mL) was added dropwise to ethylene glycol (8.36 mL, 150.0 mmol) at 0 °C over 18 min. The reaction mixture was stirred at 0 °C for 30 min. Saturated aq. NH_4Cl (30 mL) was added followed by $CHCl_3$ (30 mL). The layers were separated and the aqueous layer was extracted with $CHCl_3$ (2 x 30 mL). The organic extracts were combined, dried (Na_2SO_4) and concentrated *in vacuo*. Purification by flash chromatography (50% EtOAc in hexanes) afforded 2-[(4-Methylbenzenesulfonyl)oxy]ethan-1-ol as a pale yellow oil (1.56 g, 72%); R_f = 0.37 (50% EtOAc in hexanes); IR (film) / cm^{-1} 3418 (br O-H), 2954, 2881, 1598, 1452, 1349, 1171, 1095, 997, 909, 814, 771, 661, 578, 551; ^1H NMR (400 MHz, $CDCl_3$) δ 7.86–7.80 (m, 2 H, 2 x Ts-H), 7.37 (d, *J* = 8.2 Hz, 2 H, 2 x Ts-H), 4.18–4.13 (m, 2 H, CH_2OTs), 3.86–3.80 (m, 2 H, CH_2OH), 2.47 (s, 3 H, Ts-CH_3), 1.93 (t, *J* = 6.5 Hz, 1 H, OH); ^{13}C NMR (101 MHz, $CDCl_3$) δ 145.1 (Ts-C_q-SO_2), 132.6 (Ts-C_q-Me), 129.9 (2 x Ts-CH), 127.9 (2 x Ts-CH), 71.6 (CH_2OTs), 60.6 (CH_2OH), 21.6 (Ts-CH_3).

Observed data (IR, ^1H) was consistent with that previously reported.[32]

A mixture of diazo malonate **1** (839 mg, 4.5 mmol), 2-[(4-Methylbenzenesulfonyl)oxy]ethan-1-ol (648 mg, 3.0 mmol) and dirhodium(II) tetraacetate (6.5 mg, 0.015 mmol) in benzene (30 mL) was heated at 80 °C for 4 h. The reaction mixture was allowed to cool to rt. Water (30 mL) was added, and the aqueous mixture was extracted with $CHCl_3$ (3 x 30 mL). The organic extracts were combined, dried (Na_2SO_4) and concentrated *in vacuo*. Purification by flash chromatography (20%–30% EtOAc in hexanes) afforded tosylate **2b** as a colourless oil (640 mg, 57%).

1,3-Diethyl 2-(2-bromoethoxy)propanedioate (2c)

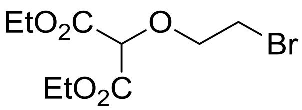

A mixture of diazo malonate **1** (279 mg, 1.5 mmol), 2-bromoethanol (71 μL, 1.0 mmol) and dirhodium(II) tetraacetate (2.1 mg, 0.005 mmol) in benzene (10 mL) was heated at 80 °C for 90 min. The reaction mixture was allowed to cool to rt. Water (10 mL) was added, and the aqueous mixture was extracted with $CHCl_3$ (2 x 20 mL). The organic extracts were combined, dried (Na_2SO_4) and concentrated *in vacuo*. Purification by flash chromatography (10% EtOAc in hexanes) afforded bromide **2c** as a colourless oil (256 mg, 90%); R_f = 0.56 (40% EtOAc in hexanes); IR (film) / cm^{-1} 2983, 2940, 2905, 1739 (C=O), 1447, 1392, 1370, 1281, 1229, 1180, 1132, 1026, 860, 601, 569; ^1H NMR (400 MHz, $CDCl_3$) δ 4.58 (s, 1 H, $CH(CO_2Et)_2$), 4.33–4.25 (m, 4 H, 2 x $CH_3CH_2CO_2$), 3.97 (t, *J* = 6.7 Hz, 2 H, OCH_2CH_2Br), 3.54 (t, *J* = 6.7 Hz, 2 H, OCH_2CH_2Br), 1.32 (t, *J* = 7.1 Hz, 6 H, 2 x CH_3); ^{13}C NMR (101 MHz, $CDCl_3$) δ 166.1 (2 x CO_2), 79.2 ($(EtO_2C)_2CHO$), 70.9 (OCH_2CH_2Br), 62.1 (2 x $CO_2CH_2CH_3$), 29.0 (OCH_2CH_2Br), 14.0 (2 x $CO_2CH_2CH_3$); HRMS (ESI-TOF) *m/z* Calcd for $C_9H_{16}BrO_5^+$ $[M+H]^+$: 283.0176, Found: 283.0186.

1,3-Diethyl 2-(2-iodoethoxy)propanedioate (2d)

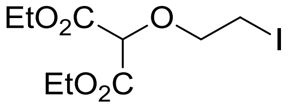

A mixture of diazo malonate **1** (840 mg, 4.5 mmol), 2-iodoethanol (235 μL, 3.0 mmol) and dirhodium(II) tetraacetate (6.7 mg, 0.015 mmol) in benzene (30 mL) was heated at 80 °C for 2 h. The reaction mixture was allowed to cool to rt. Water (30 mL) was added and the layers were separated. The aqueous layer was extracted with $CHCl_3$ (3 x 30 mL). The organic extracts were combined, dried (Na_2SO_4) and concentrated *in vacuo*. Purification by flash chromatography (10% EtOAc in hexanes) afforded iodide **2d** as a colourless oil (683 mg, 69%); R_f = 0.51 (30% EtOAc in hexanes); IR (film) / cm^{-1} 2982, 2939, 2905, 1738 (C=O), 1466, 1446, 1369, 1230, 1177, 1115, 1024, 858, 745, 706, 598; ^1H NMR (400 MHz, $CDCl_3$) δ 4.54 (s, 1 H, $CH(CO_2Et)_2$), 4.33–4.22 (m, 4 H, 2 x $CO_2CH_2CH_3$), 3.92–3.85 (m, 2 H, OCH_2CH_2I), 3.34–3.27 (m, 2 H, OCH_2CH_2I), 1.32–1.27 (m, 6 H, 2 x CH_3); ^{13}C NMR (101 MHz, $CDCl_3$) δ 166.1 (2 x CO_2), 78.8 ($(EtO_2C)_2CHO$), 71.7 (OCH_2CH_2I), 62.1 (2 x $CO_2CH_2CH_3$), 14.0 (2 x $CO_2CH_2CH_3$), 0.9 (OCH_2CH_2I); Calcd for $C_9H_{15}O_5INa^+$ $[M+Na]^+$: 352.9856, Found: 352.9850.

2,2-Diethyl Oxetane-2,2-dicarboxylate (3)

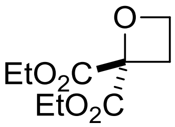

DMF (32.5 mL) was added to a flask containing sodium hydride (60% w/v, 48 mg, 1.2 mmol) which had been cooled to 0 °C. Bromide **2c** (383 mg, 1.0 mmol) in DMF (7.5 mL) was added dropwise to the stirred suspension of sodium hydride in DMF at 0 °C over 10 min. The reaction mixture was stirred at 0 °C for 1 h. Saturated aq. NH_4Cl (40 mL) was added followed by EtOAc (40 mL). The layers were separated and the aqueous layer was extracted with EtOAc (3 x 40 mL). The organic extracts were combined, dried (Na_2SO_4) and concentrated *in vacuo*. Purification by flash chromatography (20% EtOAc in hexanes) afforded oxetane **3** as a colourless oil (148 mg, 73%); R_f = 0.25 (20% EtOAc in hexanes); IR (film) / cm^{-1} 2983, 2904, 1740 (C=O), 1448, 1369, 1277, 1258, 1191, 1108, 1065, 1014, 971, 946, 857, 766, 686; ^1H NMR (400 MHz, $CDCl_3$) δ 4.65 (t, *J* = 7.7 Hz, 2 H, OCH_2), 4.294 (q, *J* = 7.1, 2 H, $CO_2CH_2CH_3$), 4.291 (q, *J* = 7.1, 2 H, $CO_2CH_2CH_3$), 3.08 (t, *J* = 7.7 Hz, 2 H, CH_2), 1.30 (t, *J* = 7.1 Hz, 6 H, 2 x $CO_2CH_2CH_3$); ^{13}C NMR (101 MHz, $CDCl_3$) δ 168.6 (2 x CO_2), 84.4 ($C_q(CO_2Et)_2$), 67.9 (OCH_2), 62.1 (2 x $CO_2CH_2CH_3$), 28.4 (CH_2), 13.9 (2 x $CO_2CH_2CH_3$); HRMS (ESI-TOF) *m/z* Calcd for $C_9H_{15}O_5^+$ $[M+H]^+$: 203.0914, Found: 203.0908.

Observed data (IR, ^{1}H, ^{13}C) was consistent with that previously reported.[1]

Synthesis of Trisubstituted Oxetanes 6a–6g, 9a–9i

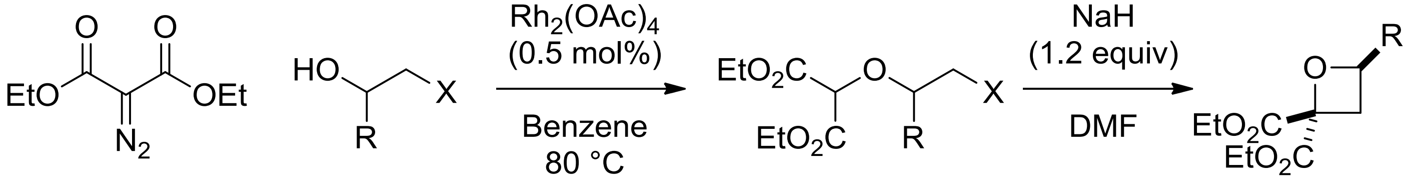

(±)-1,3-Diethyl 2-(2-bromo-1-phenylethoxy)propanedioate (5a)

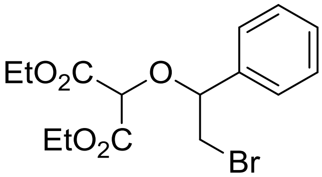

A mixture of diazo malonate **1** (836 mg, 4.5 mmol), alcohol **4a** (605 mg, 3.0 mmol) and dirhodium(II) tetraacetate (6.9 mg, 0.016 mmol) in benzene (30 mL) was heated at 80 °C for 2 h. The reaction mixture was allowed to cool to rt. Water (30 mL) was added and the layers were separated. The aqueous layer was extracted with $CHCl_3$ (3 x 30 mL). The organic extracts were combined, dried (Na_2SO_4) and concentrated *in vacuo*. Purification by flash chromatography (10% EtOAc in hexanes) afforded bromide **5a** as a colourless oil (568 mg, 79%); R_f = 0.27 (20% EtOAc in hexanes); IR (film) / cm^{-1} 2982, 1738 (C=O), 1494, 1455, 1370, 1284, 1226, 1204, 1178, 1157, 1114, 1026, 859, 758, 700, 668, 602, 547; ^{1}H NMR (400 MHz, $CDCl_3$) δ 7.40–7.35 (m, 5 H, 5 x Ph-H), 4.74 (dd, *J* = 7.5, 5.3 Hz, 1 H, OCH(Ph)), 4.41 (s, 1 H, $CH(CO_2Et)_2$), 4.35–4.23 (m, 2 H, $CH_3CH_2CO_2$), 4.20–4.10 (m, 2 H, $CH_3CH_2CO_2$), 3.74 (dd, *J* = 10.7, 7.5 Hz, 1 H, C*H*HBr), 3.53 (dd, *J* = 10.7, 5.3 Hz, 1 H, CH*H*Br), 1.31 (t, *J* = 7.1 Hz, 3 H, $CH_3CH_2CO_2$), 1.21 (t, *J* = 7.1 Hz, 3 H, $CH_3CH_2CO_2$); ^{13}C NMR (101 MHz, $CDCl_3$) δ 166.5 (CO_2), 165.8 (CO_2), 137.3 (Ph-C_q), 129.1 (Ph-CH), 128.8 (2 x Ph-H), 127.3 (2 x Ph-H), 82.4 (OCH(Ph)), 77.2 ($(EtO_2C)_2CHO$), 62.0 ($CO_2CH_2CH_3$), 61.9 ($CO_2CH_2CH_3$), 35.0 (CH_2Br), 14.1 ($CO_2CH_2CH_3$), 13.9 ($CO_2CH_2CH_3$); HRMS (CI) *m/z* Calcd for $C_{15}H_{23}BrNO_5^+$ $[M+NH_4]^+$: 376.0754, Found: 376.0769.

1,3-Diethyl 2-[(1*S*)-2-bromo-1-phenylethoxy]propanedioate ((*S*)-5a)

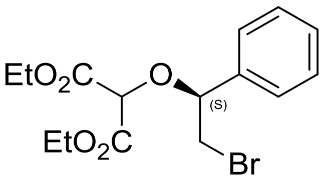

Following the procedure for the synthesis of (±)-**5a** using enantioenriched **(*S*)-4a** (402 mg, 2.0 mmol, 85% ee) afforded bromide **(*S*)-5a** as a colourless oil (607 mg, 85% ee, 84%); $[\alpha]_D^{22}$ +56.7° (c. 2.61, $CHCl_3$); **ee = 85%** by HPLC, Chiralpak IC-3 column, 90:10 *n*hexane/IPA, flow 1.0 mLmin^{-1}, UV detection at 228 nm, 9.3 min (**(*S*)-5a**), 13.2 min (**(*R*)-5a**).

Observed data was identical to the racemic bromide (±)-**5a**.

1,3-Diethyl 2-[(1*S*)-2-bromo-1-phenylethoxy]propanedioate ((*S*)-5a)

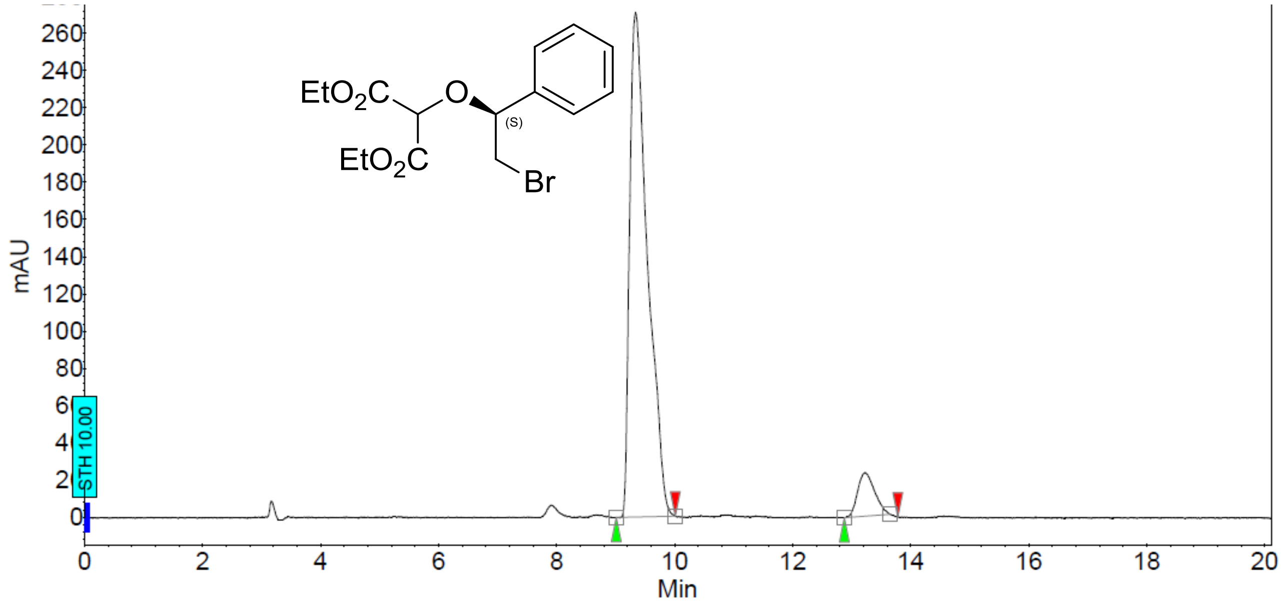

Chiralpak IC-3 column, 90:10 *n*hexane/IPA, flow 1.0 mLmin^{-1}, UV detection at 228 nm

Index	Name	Time [Min]	Quantity [% Area]	Height [mAU]	Area [mAU.Min]	Area % [%]
1	UNKNOWN	9.333	92.47	271.0	94.8	92.474
2	UNKNOWN	13.226	7.53	23.4	7.7	7.526
Total			100.00	294.4	102.5	100.000

ee = 85%

(±)-1,3-Diethyl 2-(2-bromo-1-phenylethoxy)propanedioate (5a)

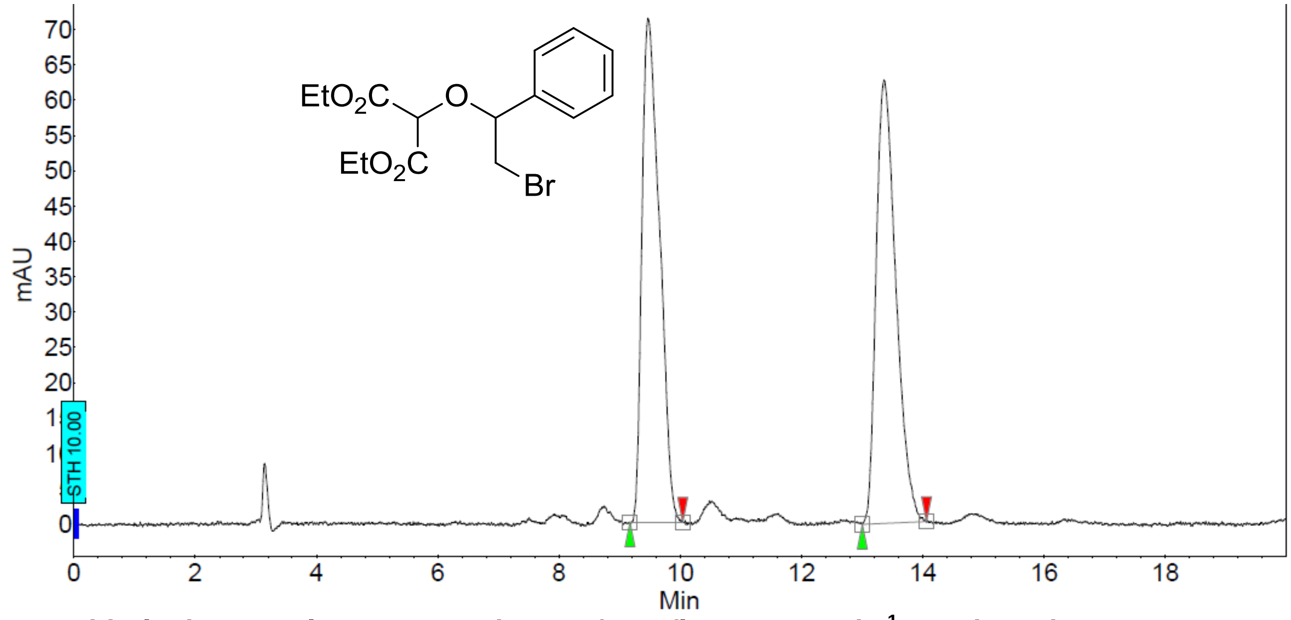

Chiralpak IC-3 column, 90:10 *n*hexane/IPA, flow 1.0 mLmin^{-1}, UV detection at 228 nm

Index	Name	Time [Min]	Quantity [% Area]	Height [mAU]	Area [mAU.Min]	Area % [%]
1	UNKNOWN	9.479	50.16	71.1	24.0	50.160
2	UNKNOWN	13.359	49.84	62.8	23.8	49.840
Total			100.00	133.8	47.8	100.000

(±)-2,2-Diethyl 4-phenyloxetane-2,2-dicarboxylate (6a)

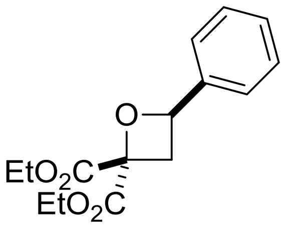

DMF (32.5 mL) was added to a flask containing sodium hydride (60% w/v, 49 mg, 1.2 mmol) which had been cooled to 0 °C. Bromide **5a** (359 mg, 1.0 mmol) in DMF (7.5 mL) was added dropwise to the stirred suspension of sodium hydride in DMF at 0 °C over 10 min. The reaction mixture was stirred at 30 °C for 16 h. Saturated aq. NH_4Cl (40 mL) was added followed by EtOAc (40 mL). The layers were separated and the aqueous layer was extracted with EtOAc (3 x 40 mL). The organic extracts were combined, dried (Na_2SO_4) and concentrated *in vacuo*. Purification by flash chromatography (20% EtOAc in hexanes) afforded oxetane **6a** as a colourless oil (234 mg, 84%); R_f = 0.29 (20% EtOAc in hexanes); IR (film) / cm^{-1} 2982, 2938, 2938, 1739 (C=O), 1447, 1369, 1260, 1228, 1127, 1055, 1012, 961, 856, 757, 698, 593, 532; ^1H NMR (400 MHz, $CDCl_3$) δ 7.50–7.45 (m, 2 H, 2 x Ph-H), 7.42–7.36 (m, 2 H, 2 x Ph-H), 7.36–7.31 (m, 1 H, Ph-H), 5.80 (t, *J* = 7.6 Hz, 1 H, OCH(Ph)), 4.41–4.30 (m, 4 H, 2 x $CO_2CH_2CH_3$), 3.39 (dd, *J* = 12.0, 7.7 Hz, 1 H, C*H*H), 3.12 (dd, *J* = 12.0, 7.5 Hz, 1 H, CH*H*), 1.35 (t, *J* = 7.1 Hz, 3 H, $CO_2CH_2CH_3$), 1.33 (t, *J* = 7.1 Hz, 3 H, $CO_2CH_2CH_3$); ^{13}C NMR (101 MHz, $CDCl_3$) δ 169.0 (CO_2), 168.4 (CO_2), 140.9 (Ph-C_q), 128.5 (3 x Ph-CH), 125.8 (2 x Ph-CH), 80.8 ($C_q(CO_2Et)_2$), 78.9 (OCH(Ph)), 62.3 ($CO_2CH_2CH_3$), 62.1 ($CO_2CH_2CH_3$), 36.7 (CH_2), 14.00 ($CO_2CH_2CH_3$), 13.98 ($CO_2CH_2CH_3$); HRMS (ESI-TOF) *m/z* Calcd for $C_{15}H_{19}O_5^+$ $[M+H]^+$: 279.1227, Found: 279.1238.

2,2-Diethyl (4*R*)-4-phenyloxetane4-phenyloxetane-2,2-dicarboxylate ((*R*)-6a)

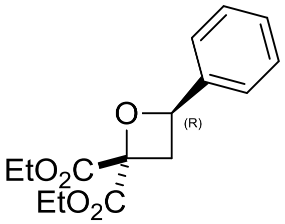

Following the procedure for the synthesis of (±)-**6a** using enantioenriched **(*S*)-5a** (360 mg, 1.0 mmol, 85% ee) afforded oxetane **(*R*)-6a** as a colourless oil (232 mg, 85% ee, 83%); $[\alpha]_D^{22}$ +60.0° (c. 2.67, $CHCl_3$); **ee = 85%** by HPLC, Chiralpak IC-3 column, 90:10 *n*hexane/IPA, flow 1.0 $mLmin^{-1}$, UV detection at 228 nm, 22.3 min (**(*R*)-6a**), 29.1 min (**(*S*)-6a**).

Observed data was identical to the racemic oxetane (±)-**6a**.

2,2-Diethyl (4*R*)-4-phenyloxetane4-phenyloxetane-2,2-dicarboxylate ((*R*)-6a)

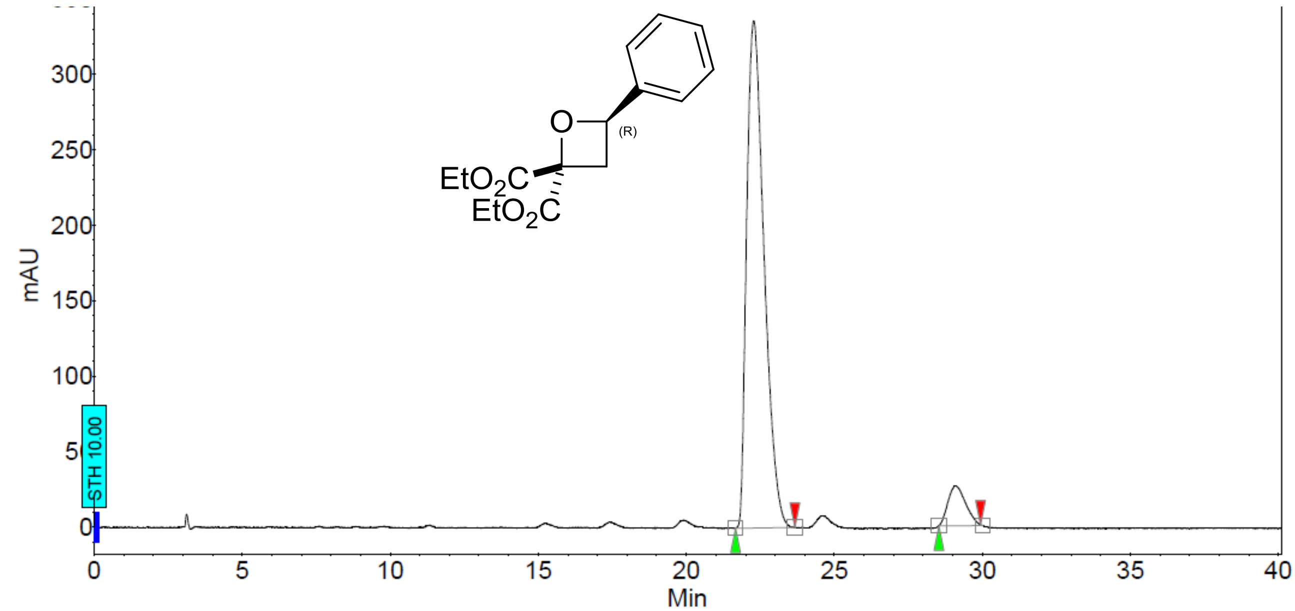

Chiralpak IC-3 column, 90:10 *n*hexane/IPA, flow 1.0 mLmin^{-1}, UV detection at 228 nm

Index	Name	Time [Min]	Quantity [% Area]	Height [mAU]	Area [mAU.Min]	Area % [%]
1	UNKNOWN	22.278	92.55	336.2	229.1	92.550
2	UNKNOWN	29.091	7.45	26.3	18.4	7.450
Total			100.00	362.5	247.5	100.000

ee = 85%

(±)-2,2-Diethyl 4-phenyloxetane-2,2-dicarboxylate (6a)

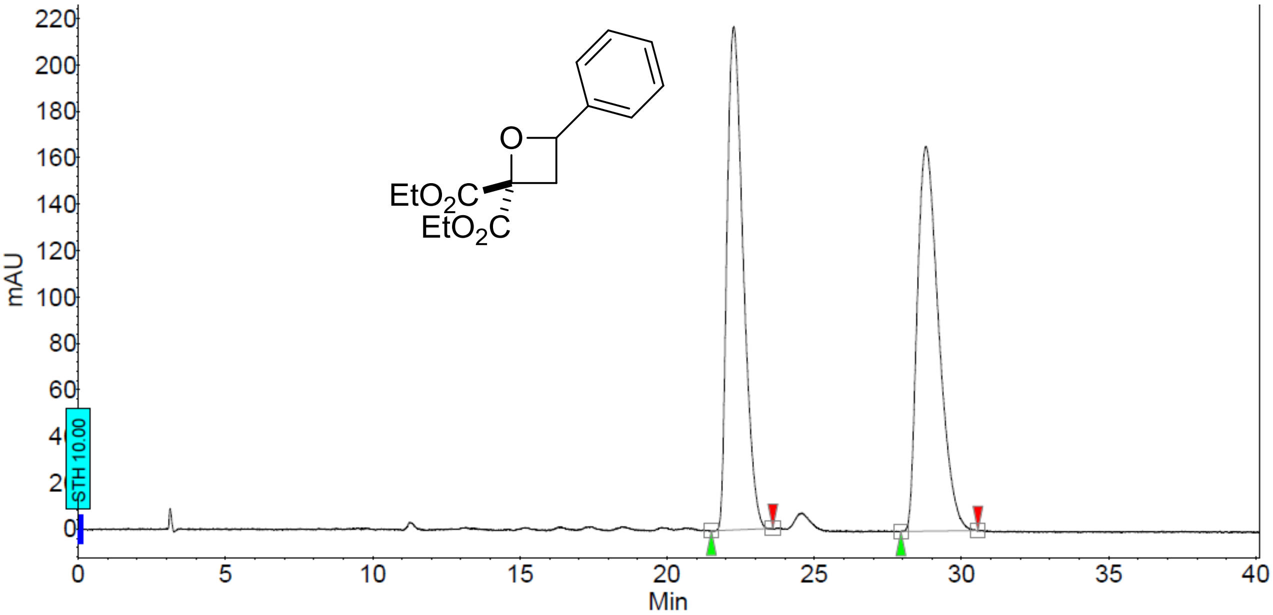

Chiralpak IC-3 column, 90:10 *n*hexane/IPA, flow 1.0 mLmin^{-1}, UV detection at 228 nm

Index	Name	Time [Min]	Quantity [% Area]	Height [mAU]	Area [mAU.Min]	Area % [%]
1	UNKNOWN	22.265	49.69	216.8	138.0	49.689
2	UNKNOWN	28.785	50.31	165.4	139.7	50.311
Total			100.00	382.2	277.8	100.000

(±)-1,3-Diethyl 2-[2-bromo-1-(4-methylphenyl)ethoxy]propanedioate (5b)

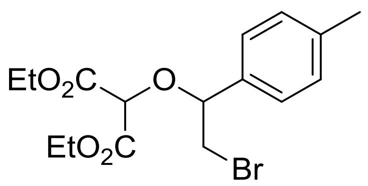

A mixture of diazo malonate **1** (280 mg, 1.5 mmol), alcohol **4b** (215 mg, 1.0 mmol) and dirhodium(II) tetraacetate (2.4 mg, 0.005 mmol) in benzene (10 mL) was heated at 80 °C for 1 h. The reaction mixture was allowed to cool to rt. Water (10 mL) was added and the layers were separated. The aqueous layer was extracted with $CHCl_3$ (3 x 10 mL). The organic extracts were combined, dried (Na_2SO_4) and concentrated *in vacuo*. Purification by flash chromatography (5% EtOAc in hexanes) afforded bromide **5b** as a pale yellow oil (285 mg, 76%); R_f = 0.33 (20% EtOAc in hexanes); IR (film) / cm^{-1} 2982, 2939, 2905, 1764 (C=O), 1739 (C=O), 1615, 1514, 1446, 1370, 1226, 1179, 1112, 1027, 818, 724, 647, 600, 564; ^1H NMR (400 MHz, $CDCl_3$) δ 7.26 (d, *J* = 8.0 Hz, 2 H, 2 x Tol-H), 7.19 (d, *J* = 8.0 Hz, 2 H, 2 x Tol-H), 4.70 (dd, *J* = 7.4, 5.5 Hz, 1 H, OCH(Tol)), 4.40 (s, 1 H, $CH(CO_2Et)_2$), 4.35–4.23 (m, 2 H, $CO_2CH_2CH_3$), 4.21–4.11 (m, 2 H, $CO_2CH_2CH_3$), 3.73 (dd, *J* = 10.6, 7.4 Hz, 1 H, C*H*HBr), 3.51 (dd, *J* = 10.6, 5.4 Hz, 1 H, CH*H*Br), 2.36 (s, 3 H, Tol-CH_3), 1.32 (t, *J* = 7.1 Hz, 3 H, $CO_2CH_2CH_3$), 1.22 (t, *J* = 7.1 Hz, 3 H, $CO_2CH_2CH_3$); ^{13}C NMR (101 MHz, $CDCl_3$) δ 166.6 (CO_2), 165.9 (CO_2), 139.1 (Tol-C_q-Me), 134.2 (Tol-C_q-CH(O), 129.5 (2 x Tol-CH), 127.3 (2 x Tol-CH), 82.3 (OCH(Tol)), 77.3 ($(EtO_2C)_2CHO$), 62.0 ($CO_2CH_2CH_3$), 61.9 ($CO_2CH_2CH_3$), 35.1 (CH_2Br), 21.2 (Tol-CH_3), 14.1 ($CO_2CH_2CH_3$), 13.9 ($CO_2CH_2CH_3$); HRMS (ESI-TOF) *m/z* Calcd for $C_{16}H_{21}BrNaO_5^+$ $[M+Na]^+$: 395.0470, Found: 395.0487.

(±)-2,2-Diethyl 4-(4-methylphenyl)oxetane-2,2-dicarboxylate (6b)

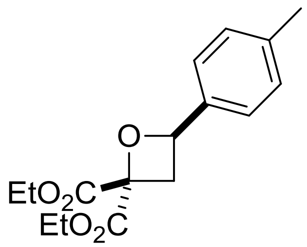

DMF (16.3 mL) was added to a flask containing sodium hydride (60% w/v, 24 mg, 0.6 mmol) which had been cooled to 0 °C. Bromide **5b** (187 mg, 0.5 mmol) in DMF (3.7 mL) was added dropwise to the stirred suspension of sodium hydride in DMF at 0 °C over 10 min. The reaction mixture was stirred at 25 °C for 16 h. Saturated aq. NH_4Cl (20 mL) was added followed by EtOAc (20 mL). The layers were separated and the aqueous layer was extracted with EtOAc (4 x 20 mL). The organic extracts were combined, dried (Na_2SO_4) and concentrated *in vacuo*. Purification by flash chromatography (20% EtOAc in hexanes) afforded oxetane **6b** as a pale yellow oil (113 mg, 77%); R_f = 0.34 (20% EtOAc in hexanes); IR (film) / cm^{-1} 2982, 2939, 1739 (C=O), 1615, 1447, 1369, 1261, 1227, 1128, 1060, 954, 928, 856, 815, 765, 679; ^1H NMR (400 MHz, $CDCl_3$) δ 7.36 (d, *J* = 8.0 Hz, 2 H, 2 x Tol-H), 7.20 (d, *J* = 8.0 Hz, 2 H, 2 x Tol-H), 5.76 (t, *J* = 7.6 Hz, 1 H, OCH(Tol)), 4.36 (q, *J* = 7.2 Hz, 2 H, $CO_2CH_2CH_3$), 4.34 (q, *J* = 7.1 Hz, 2 H, $CO_2CH_2CH_3$), 3.36 (dd, *J* = 12.0, 7.6 Hz, 1 H, C*H*H), 3.12 (dd, *J* = 12.0, 7.6 Hz, 1 H, CH*H*), 2.36 (s, 3 H, Tol-CH_3), 1.35 (t, *J* = 7.1 Hz, 3 H, $CO_2CH_2CH_3$), 1.33 (t, *J* = 7.2 Hz, 3 H, $CO_2CH_2CH_3$); ^{13}C NMR (101 MHz, $CDCl_3$) δ 169.2 (CO_2), 168.5 (CO_2), 138.4 (Tol-C_q-Me), 137.9 (Tol-C_q-CH(O)), 129.2 (2 x Tol-CH), 126.0 (2 x Tol-CH), 80.7 ($C_q(CO_2Et)_2$), 78.9 (OCH(Tol)), 62.2 ($CO_2CH_2CH_3$), 62.1 ($CO_2CH_2CH_3$), 36.8 (CH_2), 21.2 (Tol-CH_3), 14.03 ($CO_2CH_2CH_3$), 14.00 ($CO_2CH_2CH_3$); HRMS (ESI-TOF) *m/z* Calcd for $C_{16}H_{21}O_5^+$ $[M+H]^+$: 293.1384, Found: 293.1385.

(±)-1,3-Diethyl 2-[2-bromo-1-(2-fluorophenyl)ethoxy]propanedioate (5c)

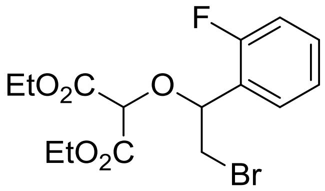

A mixture of diazo malonate **1** (557 mg, 3.0 mmol), alcohol **4c** (439 mg, 2.0 mmol) and dirhodium(II) tetraacetate (4.8 mg, 0.01 mmol) in benzene (20 mL) was heated at 80 °C for 90 min. The reaction mixture was allowed to cool to rt. Water (20 mL) was added and the layers were separated. The aqueous layer was extracted with $CHCl_3$ (2 x 20 mL). The organic extracts were combined, dried (Na_2SO_4) and concentrated *in vacuo*. Purification by flash chromatography (10% EtOAc in hexanes) afforded bromide **5c** as a colourless oil (717 mg, 95%); R_f = 0.53 (30% EtOAc in hexanes); IR (film) / cm^{-1} 2984, 1740 (C=O), 1616, 1588, 1489, 1457, 1369, 1230, 1179, 1118, 1027, 859, 826, 760, 661, 607, 566; ^1H NMR (400 MHz, $CDCl_3$) δ 7.59–7.49 (m, 1 H, Ar-H), 7.38–7.30 (m, 1 H, Ar-H), 7.22–7.17 (m, 1 H, Ar-H), 7.10–7.03 (m, 1 H, Ar-H), 5.12 (d, J = 7.1, 5.1 Hz, 1 H, OCH(Ar)), 4.47 (s, 1 H, $CH(CO_2Et)_2$), 4.34–4.24 (m, 2 H, $CO_2CH_2CH_3$), 4.21–4.11 (m, 2 H, $CO_2CH_2CH_3$), 3.71 (dd, J = 10.8, 7.1 Hz, 1 H, C*H*HBr), 3.63 (dd, J = 10.8, 5.1 Hz, 1 H, CH*H*Br), 1.31 (t, J = 7.1 Hz, 3 H, $CO_2CH_2CH_3$), 1.21 (t, J = 7.1 Hz, 3 H, C $CO_2H_2CH_3$); ^{13}C NMR (101 MHz, $CDCl_3$) δ 166.2 (CO_2), 165.7 (CO_2), 160.5 (d, J_{CF} = 247 Hz, Ar-C_q-F), 130.5 (d, J_{CF} = 8 Hz, Ar-CH), 128.2 (d, J_{CF} = 3 Hz, Ar-CH), 124.7 (d, J_{CF} = 13 Hz, Ar-C_q-CH(O)), 124.6 (d, J_{CF} = 3 Hz, Ar-CH), 115.5 (d, J_{CF} = 22 Hz, Ar-CH), 77.7 ($(EtO_2C)_2CHO$), 75.6 (d, J_{CF} = 2 Hz, OCH(Ar)), 62.1 ($CO_2CH_2CH_3$), 62.0 ($CO_2CH_2CH_3$), 34.0 (CH_2Br), 14.0 ($CO_2CH_2CH_3$), 13.8 ($CO_2CH_2CH_3$); ^{19}F NMR (376 MHz, $CDCl_3$) δ –118.4; HRMS (ESI-TOF) *m/z* Calcd for $C_{15}H_{18}BrO_5FNa^+$ $[M+Na]^+$: 399.0219, Found: 399.0231.

(±)-2,2-Diethyl 4-(2-fluorophenyl)oxetane-2,2-dicarboxylate (6c)

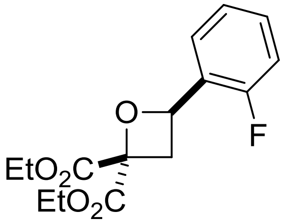

DMF (32.5 mL) was added to a flask containing sodium hydride (60% w/v, 48 mg, 1.2 mmol) which had been cooled to 0 °C. Bromide **5c** (377 mg, 1.0 mmol) in DMF (7.5 mL) was added dropwise to the stirred suspension of sodium hydride in DMF at 0 °C over 10 min. The reaction mixture was stirred at 25 °C for 16 h. Saturated aq. NH_4Cl (40 mL) was added followed by EtOAc (40 mL). The layers were separated and the aqueous layer was extracted with EtOAc (3 x 40 mL). The organic extracts were combined, dried (Na_2SO_4) and concentrated *in vacuo*. Purification by flash chromatography (20% EtOAc in hexanes) afforded oxetane **6c** as a colourless oil (240 mg, 81%); R_f = 0.38 (20% EtOAc in hexanes); IR (film) / cm^{-1} 2984, 1741 (C=O), 1618, 1588, 1491, 1457, 1369, 1260, 1232, 1128, 1061, 1013, 979, 857, 818, 759, 598; ^1H NMR (400 MHz, $CDCl_3$) δ 7.75–7.68 (m, 1 H, Ar-H), 7.34–7.25 (m, 1 H, Ar-H), 7.23–7.16 (m, 1 H, Ar-H), 7.05–6.96 (m, 1 H, Ar-H), 6.04 (t, J = 7.6 Hz, 1 H, OCH(Ar)), 4.39–4.26 (m, 4 H, 2 x $CO_2CH_2CH_3$), 3.46 (dd, J = 12.0, 7.9 Hz, 1 H, C*H*H), 3.08 (dd, J = 12.0, 7.3 Hz, 1 H, CH*H*), 1.34 (t, J = 7.1 Hz, 3 H, $CO_2CH_2CH_3$), 1.30 (t, J = 7.1 Hz, 3 H, $CO_2CH_2CH_3$); ^{13}C NMR (101 MHz, $CDCl_3$) δ 168.7 (CO_2), 168.3 (CO_2), 159.2 (d, J_{CF} = 247 Hz, Ar-C_q-F), 129.8 (d, J_{CF} = 8 Hz, Ar-CH), 128.3 (d, J_{CF} = 13 Hz, Ar-C_q-CH(O)), 127.3 (d, J_{CF} = 4 Hz, Ar-CH), 124.3 (d, J_{CF} = 3 Hz, Ar-CH), 115.1 (d, J_{CF} = 21 Hz, Ar-CH), 81.3 ($C_q(CO_2Et)_2$), 73.5 (d, J_{CF} = 4 Hz, OCH(Ar)), 62.4 ($CO_2CH_2CH_3$), 62.1 ($CO_2CH_2CH_3$), 36.0 (CH_2), 14.0 ($CO_2CH_2CH_3$), 13.9 ($CO_2CH_2CH_3$); ^{19}F NMR (376 MHz, $CDCl_3$) δ –119.2; HRMS (ESI-TOF) *m/z* Calcd for $C_{15}H_{18}O_5F^+$ $[M+H]^+$: 297.1138 Found: 297.1142.

(±)-1,3-Diethyl 2-[2-bromo-1-(4-chlorophenyl)ethoxy]propanedioate (5d)

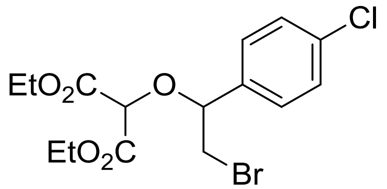

A mixture of diazo malonate **1** (837 mg, 4.5 mmol), alcohol **4d** (707 mg, 3.0 mmol) and dirhodium(II) tetraacetate (6.9 mg, 0.016 mmol) in benzene (30 mL) was heated at 80 °C for 2 h. The reaction mixture was allowed to cool to rt. Water (30 mL) was added and the layers were separated. The aqueous layer was extracted with $CHCl_3$ (3 x 30 mL). The organic extracts were combined, dried (Na_2SO_4) and concentrated *in vacuo*. Purification by flash chromatography (10% EtOAc in hexanes) afforded bromide **5d** as a colourless oil (830 mg, 70%); R_f = 0.61 (30% EtOAc in hexanes); IR (film) / cm^{-1} 2982, 1738 (C=O), 1598, 1491, 1370, 1297, 1227, 1203, 1178, 1116, 1090, 1027, 1014, 828, 729, 633, 596, 551, 530; ^{1}H NMR (400 MHz, $CDCl_3$) δ 7.41–7.32 (m, 2 H, 2 x Ar-H), 7.36–7.28 (m, 2 H, 2 x Ar-H), 4.72 (dd, *J* = 6.9, 5.8 Hz, 1 H, OCH(Ar)), 4.39 (s, 1 H, $CH(CO_2Et)_2$), 4.36–4.20 (m, 2 H, $CO_2C\mathit{H_2}CH_3$), 4.22–4.09 (m, 2 H, $CO_2C\mathit{H_2}CH_3$), 3.71 (dd, *J* = 10.6, 6.9 Hz, 1 H, C*H*HBr), 3.49 (dd, *J* = 10.6, 5.8 Hz, 1 H, CH*H*Br), 1.31 (t, *J* = 7.2 Hz, 3 H, $CO_2CH_2C\mathit{H_3}$), 1.22 (t, *J* = 7.1 Hz, 3 H, $CO_2CH_2C\mathit{H_3}$); ^{13}C NMR (101 MHz, $CDCl_3$) δ 166.3 (CO_2), 165.7 (CO_2), 135.9 (Ar-C_q-CH(O)), 135.0 (Ar-C_q-Cl), 129.0 (2 x Ar-CH), 128.7 (2 x Ar-CH), 81.6 (OCH(Ar)), 77.2 ($(EtO_2C)_2\mathit{C}HO$), 62.1 ($CO_2\mathit{C}H_2CH_3$), 62.0 ($CO_2\mathit{C}H_2CH_3$), 34.7 (CH_2Br), 14.0 ($CO_2CH_2\mathit{C}H_3$), 13.9 ($CO_2CH_2CH_3$); HRMS (ESI-TOF) *m/z* Calcd for $C_{15}H_{18}BrClO_5Na^+$ $[M+Na]^+$: 414.9924, Found: 414.9924.

Reaction performed on a large scale (9.0 mmol alcohol **4d**) afforded an improved isolated yield of bromide **5d** (3.11 g, 88%).

(±)-2,2-Diethyl 4-(4-chlorophenyl)oxetane-2,2-dicarboxylate (6d)

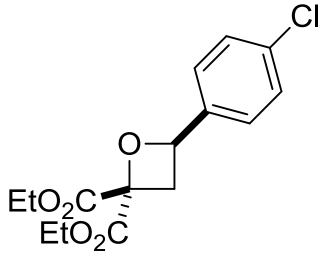

DMF (32.5 mL) was added to a flask containing sodium hydride (60% w/v, 48 mg, 1.2 mmol) which had been cooled to 0 °C. Bromide **5d** (393 mg, 1.0 mmol) in DMF (7.5 mL) was added dropwise to the stirred suspension of sodium hydride in DMF at 0 °C over 10 min. The reaction mixture was stirred at 25 °C for 16 h. Saturated aq. NH_4Cl (40 mL) was added followed by EtOAc (40 mL). The layers were separated and the aqueous layer was extracted with EtOAc (3 x 40 mL). The organic extracts were combined, dried (Na_2SO_4) and concentrated *in vacuo*. Purification by flash chromatography (20% EtOAc in hexanes) afforded oxetane **6d** as a colourless oil (238 mg, 76%); R_f = 0.29 (20% EtOAc in hexanes); IR (film) / cm^{-1} 2984, 2939, 2909, 1739 (C=O), 1599, 1493, 1446, 1369, 1261, 1128, 1090, 1060, 1014, 958, 882, 856, 824, 775, 744, 690, 539; ^{1}H NMR (400 MHz, $CDCl_3$) δ 7.45–7.37 (m, 2 H, 2 x Ar-H), 7.37–7.33 (m, 2 H, 2 x Ar-H), 5.75 (t, *J* = 7.6 Hz, 1 H, OCH(Ar)), 4.39–4.29 (m, 4 H, 2 x $CO_2C\mathit{H_2}CH_3$), 3.39 (dd, *J* = 12.0, 7.6 Hz, 1 H, C*H*H), 3.04 (dd, *J* = 12.0, 7.6 Hz, 1 H, CH*H*), 1.34 (t, *J* = 7.2 Hz, 3 H, $CO_2CH_2C\mathit{H_3}$), 1.31 (t, *J* = 7.2 Hz, 3 H, $CO_2CH_2C\mathit{H_3}$); ^{13}C NMR (101 MHz, $CDCl_3$) δ 168.8 (CO_2), 168.3 (CO_2), 139.4 (Ar-C_q-CH(O)), 134.3 (Ar-C_q-Cl), 128.8 (2 x Ar-CH), 127.2 (2 x Ar-CH), 80.8 ($\mathit{C_q}(CO_2Et)_2$), 78.2 (OCH(Ar)), 62.4 ($CO_2\mathit{C}H_2CH_3$), 62.2 ($CO_2\mathit{C}H_2CH_3$), 36.7 (CH_2), 14.00 ($CO_2CH_2\mathit{C}H_3$), 13.98 ($CO_2CH_2\mathit{C}H_3$); HRMS (ESI-TOF) *m/z* Calcd for $C_{15}H_{18}ClO_5^+$ $[M+H]^+$: 313.0837, Found: 313.0833.

Reaction performed on a large scale (6.5 mmol bromide **5d**) afforded an isolated yield of oxetane **6d** (1.58 g, 77%).

(±)-1,3-Diethyl 2-[2-bromo-1-(4-bromophenyl)ethoxy]propanedioate (5e)

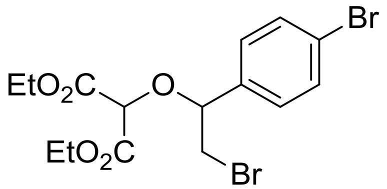

A mixture of diazo malonate **1** (838 mg, 4.5 mmol), alcohol **4e** (839 mg, 3.0 mmol) and dirhodium(II) tetraacetate (6.2 mg, 0.014 mmol) in benzene (30 mL) was heated at 80 °C for 2 h. The reaction mixture was allowed to cool to rt. Water (30 mL) was added and the layers were separated. The aqueous layer was extracted with $CHCl_3$ (3 x 30 mL). The organic extracts were combined, dried (Na_2SO_4) and concentrated *in vacuo*. Purification by flash chromatography (10% EtOAc in hexanes) afforded bromide **5e** as a colourless oil (890 mg, 68%); R_f = 0.34 (20% EtOAc in hexanes); IR (film) / cm^{-1} 2983, 1740 (C=O), 1593, 1487, 1446, 1407, 1370, 1298, 1227, 1203, 1120, 1072, 1028, 1010, 825, 725, 622, 552; ^{1}H NMR (400 MHz, $CDCl_3$) δ 7.55–7.50 (m, 2 H, 2 x Ar-H), 7.29–7.25 (m, 2 H, 2 x Ar-H), 4.71 (dd, *J* = 6.9, 5.9 Hz, 1 H, OCH(Ar)), 4.39 (s, 1 H, CH(CO_2Et)$_2$), 4.35–4.23 (m, 2 H, $CO_2CH_2CH_3$), 4.22–4.11 (m, 2 H, $CO_2CH_2CH_3$), 3.71 (dd, *J* = 10.6, 6.9 Hz, 1 H, C*H*HBr), 3.49 (dd, *J* = 10.6, 5.9 Hz, 1 H, CH*H*Br), 1.31 (t, *J* = 7.1 Hz, 3 H, $CO_2CH_2CH_3$), 1.23 (t, *J* = 7.1 Hz, 3 H, $CO_2CH_2CH_3$); ^{13}C NMR (101 MHz, $CDCl_3$) δ 166.3 (CO_2), 165.7 (CO_2), 136.4 (Ar-C_q-CH(O)), 131.9 (2 x Ar-CH), 129.0 (2 x Ar-CH), 123.2 (Ar-C_q-Br), 81.7 (OCH(Ar)), 77.2 ((EtO_2C)$_2$*C*HO), 62.13 ($CO_2CH_2CH_3$), 62.06 ($CO_2CH_2CH_3$), 34.7 (CH_2Br), 14.1 ($CO_2CH_2CH_3$), 13.9 ($CO_2CH_2CH_3$); HRMS (ESI-TOF) *m/z* Calcd for $C_{15}H_{20}Br_2O_6$ [M+H_2O]: 453.9627, Found: 453.9644.

(±)-2,2-Diethyl 4-(4-bromophenyl)oxetane-2,2-dicarboxylate (6e)

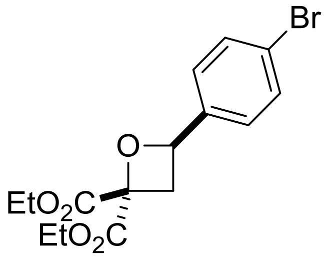

DMF (32.5 mL) was added to a flask containing sodium hydride (60% w/v, 49 mg, 1.2 mmol) which had been cooled to 0 °C. Bromide **5e** (440 mg, 1.0 mmol) in DMF (7.5 mL) was added dropwise to the stirred suspension of sodium hydride in DMF at 0 °C over 10 min. The reaction mixture was stirred at 25 °C for 16 h. Saturated aq. NH_4Cl (40 mL) was added followed by EtOAc (40 mL). The layers were separated and the aqueous layer was extracted with EtOAc (3 x 40 mL). The organic extracts were combined, dried (Na_2SO_4) and concentrated *in vacuo*. Purification by flash chromatography (20% EtOAc in hexanes) afforded oxetane **6e** as a colourless oil (299 mg, 83%); R_f = 0.34 (20% EtOAc in hexanes); IR (film) / cm^{-1} 2983, 1740 (C=O), 1595, 1489, 1446, 1369, 1264, 1229, 1128, 1064, 1011, 962, 882, 821, 773, 691, 537, 530; ^{1}H NMR (400 MHz, $CDCl_3$) δ 7.54–7.49 (m, 2 H, 2 x Ar-H), 7.38–7.33 (m, 2 H, 2 x Ar-H), 5.74 (dd, *J* = 7.6, 7.6 Hz, 1 H, OCH(Ar)), 4.40–4.29 (m, 4 H, 2 x $CO_2CH_2CH_3$), 3.40 (dd, *J* = 12.0, 7.6 Hz, 1 H, C*H*H), 3.04 (dd, *J* = 12.0, 7.6 Hz, 1 H, CH*H*), 1.34 (t, *J* = 7.1 Hz, 3 H, $CO_2CH_2CH_3$), 1.31 (t, *J* = 7.2 Hz, 3 H, $CO_2CH_2CH_3$); ^{13}C NMR (101 MHz, $CDCl_3$) δ 168.8 (CO_2), 168.3 (CO_2), 140.0 (Ar-C_q-CH(O)), 131.7 (2 x Ar-CH), 127.5 (2 x Ar-CH), 122.5 (Ar-C_q-Br), 80.8 ((C_q(CO_2Et)$_2$), 78.2 (OCH(Ar)), 62.4 ($CO_2CH_2CH_3$), 62.3 ($CO_2CH_2CH_3$), 36.6 (CH_2), 14.02 ($CO_2CH_2CH_3$), 14.00 ($CO_2CH_2CH_3$); HRMS (ESI-TOF) *m/z* Calcd for $C_{15}H_{18}BrO_5^+$ [M+H]$^+$: 357.0332, Found: 357.0342.

(±)-1,3-Diethyl 2-[2-bromo-1-(3-methoxyphenyl)ethoxy]propanedioate (5f)

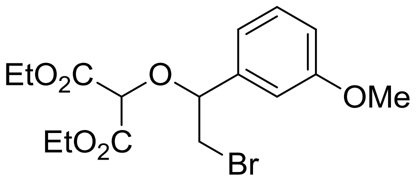

A mixture of diazo malonate **1** (558 mg, 3.0 mmol), alcohol **4f** (462 mg, 2.0 mmol) and dirhodium(II) tetraacetate (4.6 mg, 0.01 mmol) in benzene (20 mL) was heated at 80 °C for 90 min. The reaction mixture was allowed to cool to rt. Water (20 mL) was added and the layers were separated. The aqueous layer was extracted with $CHCl_3$ (2 x 20 mL). The organic extracts were combined, dried (Na_2SO_4) and concentrated *in vacuo*. Purification by flash chromatography (10% EtOAc in hexanes) afforded bromide **5f** as a colourless oil (566 mg, 73%); R_f = 0.43 (30% EtOAc in hexanes); IR (film) / cm^{-1} 2982, 2938, 2839, 1738 (C=O), 1601, 1587, 1489, 1466, 1370, 1257, 1115, 1027, 860, 788, 703, 671, 571; ^{1}H NMR (400 MHz, $CDCl_3$) δ 7.33–7.25 (m, 1 H, Ar-H), 6.97–6.91 (m, 2 H, 2 x Ar-H), 6.91–6.86 (m, 1 H, Ar-H), 4.72 (dd, *J* = 7.4, 5.2 Hz, 1 H, OCH(Ar)), 4.42 (s, 1 H, $CH(CO_2Et)_2$), 4.34–4.24 (m, 2 H, $CO_2CH_2CH_3$), 4.22–4.12 (m, 2 H, $CO_2CH_2CH_3$), 3.81 (s, 3 H, OCH_3), 3.71 (dd, *J* = 10.6, 7.4 Hz, 1 H, C*H*HBr), 3.52 (dd, *J* = 10.6, 5.2 Hz, 1 H, CH*H*Br), 1.31 (t, *J* = 7.1 Hz, 3 H, $CO_2CH_2CH_3$), 1.22 (t, *J* = 7.1 Hz, 3 H, $CO_2CH_2CH_3$); ^{13}C NMR (101 MHz, $CDCl_3$) δ 166.5 (CO_2), 165.9 (CO_2), 159.9 (Ar-C_q-OMe), 138.9 (Ar-C_q-CH(O), 129.8 (Ar-CH), 119.6 (Ar-CH), 114.8 (Ar-CH), 112.4 (Ar-CH), 82.4 (OCH(Ar)), 77.2 ($(EtO_2C)_2CHO$), 62.0 ($CO_2CH_2CH_3$), 61.9 ($CO_2CH_2CH_3$), 55.3 (OCH_3), 35.0 (CH_2Br), 14.1 ($CO_2CH_2CH_3$), 13.9 ($CO_2CH_2CH_3$); HRMS (ESI-TOF) *m/z* Calcd for $C_{16}H_{21}BrNaO_6^+$ $[M+Na]^+$: 411.0419, Found: 411.0428.

(±)-2,2-Diethyl 4-(3-methoxyphenyl)oxetane-2,2-dicarboxylate (6f)

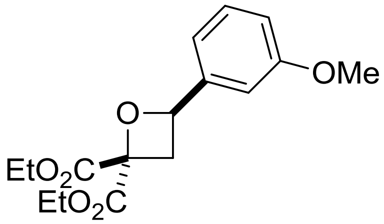

DMF (32.5 mL) was added to a flask containing sodium hydride (60% w/v, 48 mg, 1.2 mmol) which had been cooled to 0 °C. Bromide **5f** (390 mg, 1.0 mmol) in DMF (7.5 mL) was added dropwise to the stirred suspension of sodium hydride in DMF at 0 °C over 8 min. The reaction mixture was stirred at 25 °C for 16 h. Saturated aq. NH_4Cl (40 mL) was added followed by EtOAc (40 mL). The layers were separated and the aqueous layer was extracted with EtOAc (3 x 20 mL). The organic extracts were combined, dried (Na_2SO_4) and concentrated *in vacuo*. Purification by flash chromatography (20% EtOAc in hexanes) afforded oxetane **6f** as a colourless oil (273 mg, 88%); R_f = 0.26 (20% EtOAc in hexanes); IR (film) / cm^{-1} 2982, 2938, 2837, 1739 (C=O), 1604, 1587, 1488, 1466, 1369, 1259, 1126, 1063, 1043, 1013, 977, 850, 785, 695; ^{1}H NMR (400 MHz, $CDCl_3$) δ 7.32–7.25 (m, 1 H, Ar-H), 7.11–7.07 (m, 1H, Ar-H), 7.00 (d, *J* = 7.6 Hz, 1 H, Ar-H), 6.87 (dd, *J* = 8.2, 2.1 Hz, 1 H, Ar-H), 5.77 (t, *J* = 7.5 Hz, 1 H, OCH(Ar)), 4.40–4.29 (m, 4 H, 2 x $CO_2CH_2CH_3$), 3.83 (s, 3 H, OCH_3), 3.40 (dd, *J* = 11.9, 7.7 Hz, 1 H, C*H*H), 3.08 (dd, *J* = 11.9, 7.5 Hz, 1 H, CH*H*), 1.35 (t, *J* = 7.1 Hz, 3 H, $CO_2CH_2CH_3$), 1.32 (t, *J* = 7.2 Hz, 3 H, $CO_2CH_2CH_3$); ^{13}C NMR (101 MHz, $CDCl_3$) δ 167.0 (CO_2), 168.4 (CO_2), 159.8 (Ar-C_q-OMe), 142.6 (Ar-C_q-CH(O)), 129.6 (Ar-CH), 117.7 (Ar-CH), 114.2 (Ar-CH), 110.9 (Ar-CH), 80.9 ($C_q(CO_2Et)_2$), 78.7 (OCH(Ar)), 62.3 ($CO_2CH_2CH_3$), 62.1 ($CO_2CH_2CH_3$), 55.2 (OCH_3), 36.7 (CH_2), 14.01 ($CO_2CH_2CH_3$), 13.99 ($CO_2CH_2CH_3$); HRMS (ESI-TOF) *m/z* Calcd for $C_{16}H_{20}O_6Na^+$ $[M+Na]^+$: 331.1158, Found: 331.1161.

(±)-1,3-Diethyl 2-[2-bromo-1-(4-cyanophenyl)ethoxy]propanedioate (5g)

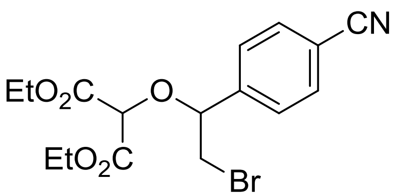

A mixture of diazo malonate **1** (565 mg, 3.0 mmol), alcohol **4g** (452 mg, 2.0 mmol) and dirhodium(II) tetraacetate (4.5 mg, 0.01 mmol) in benzene (20 mL) was heated at 80 °C for 90 min. The reaction mixture was allowed to cool to rt. Water (20 mL) was added and the layers were separated. The aqueous layer was extracted with $CHCl_3$ (2 x 20 mL). The organic extracts were combined, dried (Na_2SO_4) and concentrated *in vacuo*. Purification by flash chromatography (10% EtOAc in hexanes) afforded bromide **5g** as a colourless oil (541 mg, 71%); R_f = 0.38 (30% EtOAc in hexanes); IR (film) / cm^{-1} 2906, 2230 (C≡N), 1738 (C=O), 1610, 1505, 1466, 1446, 1370, 1280, 1230, 1205, 1180, 1116, 1021, 838, 642, 567; ^{1}H NMR (400 MHz, $CDCl_3$) δ 7.70 (d, *J* = 8.4 Hz, 2 H, 2 x Ar-H), 7.53 (d, *J* = 8.4 Hz, 2 H, 2 x Ar-H), 4.81 (t, *J* = 6.2 Hz, 1 H, OCH(Ar)), 4.43 (s, 1 H, $CH(CO_2Et)_2$), 4.37–4.24 (m, 2 H, $CO_2CH_2CH_3$), 4.23–4.13 (m, 2 H, $CO_2CH_2CH_3$), 3.71 (dd, *J* = 10.6, 6.6 Hz, 1 H, C*H*HBr), 3.52 (dd, *J* = 10.6, 5.8 Hz, 1 H, CH*H*Br), 1.30 (t, *J* = 7.1 Hz, 3 H, $CO_2CH_2CH_3$), 1.23 (t, *J* = 7.1 Hz, 3 H, C $CO_2H_2CH_3$); ^{13}C NMR (101 MHz, $CDCl_3$) δ 166.0 (CO_2), 165.1 (CO_2), 142.9 (Ar-C_q-CH(O)), 132.5 (2 x Ar-CH), 128.0 (2 x Ar-CH), 118.3 (C≡N), 112.9 (Ar-C_q-CN), 81.3 (OCH(Ar)), 77.6 ($(EtO_2C)_2CHO$), 62.25 ($CO_2CH_2CH_3$), 62.19 ($CO_2CH_2CH_3$), 34.3 (CH_2Br), 14.0 ($CO_2CH_2CH_3$), 13.9 ($CO_2CH_2CH_3$); HRMS (CI) *m/z* Calcd for $C_{16}H_{22}BrN_2O_6^+$ $[M+NH_4]^+$: 401.0712, Found: 401.0707.

(±)-2,2-Diethyl 4-(4-cyanophenyl)oxetane-2,2-dicarboxylate (6g)

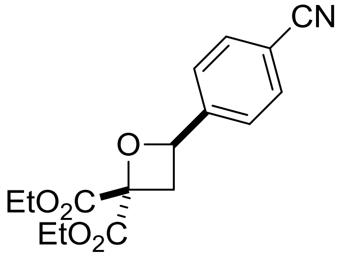

DMF (32.5 mL) was added to a flask containing sodium hydride (60% w/v, 48 mg, 1.2 mmol) which had been cooled to 0 °C. Bromide **5g** (387 mg, 1.0 mmol) in DMF (7.5 mL) was added dropwise to the stirred suspension of sodium hydride in DMF at 0 °C over 6 min. The reaction mixture was stirred at 25 °C for 16 h. Saturated aq. NH_4Cl (40 mL) was added followed by EtOAc (40 mL). The layers were separated and the aqueous layer was extracted with EtOAc (3 x 40 mL). The organic extracts were combined, dried (Na_2SO_4) and concentrated *in vacuo*. Purification by flash chromatography (30% EtOAc in hexanes) afforded oxetane **6g** as a colourless oil which crystallised to a white crystalline solid upon storage at –20 °C (251 mg, 82%); R_f = 0.39 (40% EtOAc in hexanes); mp = 34–36 °C; IR (film) / cm^{-1} 2983, 2229 (C≡N), 1739 (C=O), 1611, 1506, 1447, 1369, 1263, 1175, 1128, 1061, 1013, 977, 835, 694, 564; ^{1}H NMR (400 MHz, $CDCl_3$) δ 7.68 (d, *J* = 8.3 Hz, 2 H, 2 x Ar-H), 7.57 (d, *J* = 8.3 Hz, 2H, 2 x Ar-H), 5.82 (t, *J* = 7.6 Hz, 1 H, OCH(Ar)), 4.40–4.26 (m, 4 H, 2 x $CO_2CH_2CH_3$), 3.47 (dd, *J* = 12.0, 7.9 Hz, 1 H, C*H*H), 2.99 (dd, *J* = 12.0, 7.2 Hz, 1 H, CH*H*), 1.34 (t, *J* = 7.1 Hz, 3 H, $CO_2CH_2CH_3$), 1.30 (t, *J* = 7.1 Hz, 3 H, $CO_2CH_2CH_3$); ^{13}C NMR (101 MHz, $CDCl_3$) δ 168.4 (CO_2), 168.0 (CO_2), 146.2 (Ar-C_q-CH(O)), 132.4 (2 x Ar-CH), 126.0 (2 x Ar-CH), 118.5 (C≡N), 112.1 (Ar-C_q-CN), 81.0 ($C_q(CO_2Et)_2$), 77.3 (OCH(Ar)), 62.5 ($CO_2CH_2CH_3$), 62.3 ($CO_2CH_2CH_3$), 36.4 (CH_2), 14.0 ($CO_2CH_2CH_3$), 13.9 ($CO_2CH_2CH_3$); HRMS (ESI-TOF) *m/z* Calcd for $C_{16}H_{18}NO_5^+$ $[M+H]^+$: 304.1185, Found: 304.1188.

(±)-1,3-Diethyl 2-{[1-(benzyloxy)-3-bromopropan-2-yl]oxy}propanedioate (8a)

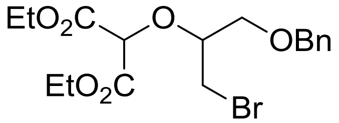

A mixture of diazo malonate **1** (279 mg, 1.5 mmol), alcohol **7a** (242 mg, 1.0 mmol) and dirhodium(II) tetraacetate (2.2 mg, 0.005 mmol) in benzene (10 mL) was heated at 80 °C for 90 min. The reaction mixture was allowed to cool to rt. Water (10 mL) was added and the layers were separated. The aqueous layer was extracted with $CHCl_3$ (3 x 10 mL). The organic extracts were combined, dried (Na_2SO_4) and concentrated *in vacuo*. Purification by flash chromatography (10% EtOAc in hexanes) afforded bromide **8a** as a colourless oil (268 g, 67%); R_f = 0.29 (20% EtOAc in hexanes); IR (film) / cm^{-1} 2982, 2937, 1761 (C=O), 1740 (C=O), 1454, 1369, 1228, 1178, 1115, 1026, 860, 738, 698, 671, 598; ^{1}H NMR (400 MHz, $CDCl_3$) δ 7.37–7.27 (m, 5 H, 5 x Ph-H), 4.85 (s, 1 H, $CH(CO_2Et)_2$), 4.54 (s, 2 H, OCH_2Ph) 4.28–4.19 (m, 4 H, 2 x $CO_2CH_2CH_3$), 3.98–3.93 (m, 1 H, $BnOCH_2CH(O)$)), 3.75 (dd, *J* = 10.2, 4.9 Hz, 1 H, BnOC*H*H), 3.72 (dd, *J* = 10.2, 5.6 Hz, 1 H, BnOCH*H*), 3.57 (dd, *J* = 11.3, 5.4 Hz, 1 H, C*H*HBr), 3.55 (dd, *J* = 11.3, 5.8 Hz, 1 H, CH*H*Br), 1.32–1.24 (m, 6 H, 2 x $CO_2CH_2CH_3$); ^{13}C NMR (101 MHz, $CDCl_3$) δ 166.4 (2 x CO_2), 137.6 (Ph-C_q), 128.3 (2 x Ph-CH), 127.7 (Ph-CH), 127.6 (2 x Ph-CH), 79.5 (($BnOCH_2$)*C*H(O)), 79.0 (($EtO_2C)_2$*C*HO), 73.4 (O*C*H_2Ph), 70.9 (BnO*C*H_2), 62.0 ($CO_2CH_2CH_3$), 61.9 ($CO_2CH_2CH_3$), 31.2 (CH_2Br), 14.0 ($CO_2CH_2CH_3$), 13.9 ($CO_2CH_2CH_3$); HRMS (ESI-TOF) *m/z* Calcd for $C_{17}H_{23}BrNaO_6^+$ $[M+Na]^+$: 425.0570, Found: 425.0576.

(±)-2,2-Diethyl 4-[(benzyloxy)methyl]oxetane-2,2-dicarboxylate (9a)

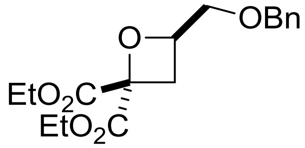

DMF (12.4 mL) was added to a flask containing sodium hydride (60% w/v, 19 mg, 0.48 mmol) which had been cooled to 0 °C. Bromide **8a** (152 mg, 0.38 mmol) in DMF (2.8 mL) was added dropwise to the stirred suspension of sodium hydride in DMF at 0 °C over 8 min. The reaction mixture was stirred at 25 °C for 16 h. Saturated aq. NH_4Cl (15 mL) was added followed by EtOAc (15 mL). The layers were separated and the aqueous layer was extracted with EtOAc (3 x 15 mL). The organic extracts were combined, dried (Na_2SO_4) and concentrated *in vacuo*. Purification by flash chromatography (30% EtOAc in hexanes) afforded oxetane **9a** as a colourless oil (108 mg, 89%); R_f = 0.28 (30% EtOAc in hexanes); IR (film) / cm^{-1} 2982, 2934, 1741 (C=O), 1452, 1368, 1258, 1138, 1092, 1076, 1026, 877, 856, 795, 737, 698, 607, 585; ^{1}H NMR (400 MHz, $CDCl_3$) δ 7.36–7.26 (m, 5 H, 5 x Ph-H), 4.96–4.91 (m, 1 H, OC*H*(CH_2OBn)), 4.64 (d, *J* = 12.1 Hz, 1 H, OC*H*HPh), 4.59 (d, *J* = 12.1 Hz, 1 H, OCH*H*Ph), 4.30 (q, *J* = 7.1 Hz, 2 H, $CO_2CH_2CH_3$), 4.26–4.18 (m, 2 H, $CO_2CH_2CH_3$), 3.69 (dd, *J* = 11.5, 3.5 Hz, 1 H, BnOC*H*H), 3.64 (dd, *J* = 11.5, 4.3 Hz, 1 H, BnOCH*H*), 3.07 (dd, *J* = 11.9, 6.9 Hz, 1 H, C*H*H), 3.03 (dd, *J* = 11.9, 7.7 Hz, 1 H, CH*H*), 1.31 (t, *J* = 7.1 Hz, 3 H, $CO_2CH_2CH_3$), 1.25 (t, *J* = 7.1 Hz, 3 H, $CO_2CH_2CH_3$); ^{13}C NMR (101 MHz, $CDCl_3$) δ 168.9 (CO_2), 168.5 (CO_2), 138.0 (Ph-C_q), 128.3 (2 x Ph-CH), 127.6 (3 x Ph-CH), 81.5 ($C_q(CO_2Et)_2$), 77.3 (O*C*H(CH_2OBn)), 73.5 (O*C*H_2Ph), 71.8 (*C*H_2OBn), 62.2 ($CO_2CH_2CH_3$), 61.9 ($CO_2CH_2CH_3$), 30.1 (CH_2), 14.0 ($CO_2CH_2CH_3$), 13.9 ($CO_2CH_2CH_3$); HRMS (ESI-TOF) *m/z* Calcd for $C_{17}H_{22}O_6Na^+$ $[M+Na]^+$: 345.1314, Found: 345.1324.

(±)-1,3-Diethyl 2-[(1-bromo-3-phenoxypropan-2-yl)oxy]propanedioate (8b)

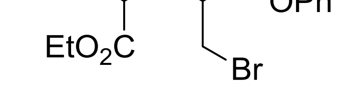

A mixture of diazo malonate **1** (838 mg, 4.5 mmol), alcohol **7b** (694 mg, 3.0 mmol) and dirhodium(II) tetraacetate (7.1 mg, 0.016 mmol) in benzene (30 mL) was heated at 80 °C for 90 min. The reaction mixture was allowed to cool to rt. Water (30 mL) was added and the layers were separated. The aqueous layer was extracted with $CHCl_3$ (3 x 30 mL). The organic extracts were combined, dried (Na_2SO_4) and

concentrated *in vacuo*. Purification by flash chromatography (10% EtOAc in hexanes) afforded bromide **8b** as a colourless oil (1.08 g, 92%); R_f = 0.31 (20% EtOAc in hexanes); IR (film) / cm^{-1} 2983, 1740 (C=O), 1600, 1588, 1497, 1456, 1370, 1292, 1240, 1174, 1130, 1027, 860, 814, 755, 692, 600; ^1H NMR (400 MHz, $CDCl_3$) δ 7.32–7.27 (m, 2 H, 2 x Ph-H), 7.00–6.96 (m, 1 H, Ph-H), 6.92–6.89 (m, 2 H, 2 x Ph-H), 4.93 (s, 1 H, $CH(CO_2Et)_2$), 4.33–4.14 (m, 7 H, 2 x $CO_2CH_2CH_3$ and $PhOCH_2$ and $PhOCH_2CH(O)$), 3.68–3.66 (m, 2 H, CH_2Br), 1.32–1.23 (m, 6 H, 2 x $CO_2CH_2CH_3$); ^{13}C NMR (101 MHz, $CDCl_3$) δ 166.4 (CO_2), 166.3 (CO_2), 158.0 (Ph-C_q), 129.5 (2 x Ph-CH), 121.3 (Ph-CH), 114.4 (2 x Ph-CH), 79.3 ($(EtO_2C)_2CHO$), 78.9 ($PhOCH_2CH(O)$), 68.6 ($PhOCH_2$), 62.2 ($CO_2CH_2CH_3$), 62.1 ($CO_2CH_2CH_3$), 30.9 (CH_2Br), 14.0 ($CO_2CH_2CH_3$), 13.9 ($CO_2CH_2CH_3$); HRMS (ESI-TOF) *m/z* Calcd for $C_{16}H_{21}BrNaO_6^+$ $[M+Na]^+$: 411.0414, Found: 411.0416.

(±)-2,2-Diethyl 4-(phenoxymethyl)oxetane-2,2-dicarboxylate (9b)

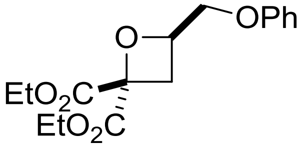

DMF (32.5 mL) was added to a flask containing sodium hydride (60% w/v, 49 mg, 1.2 mmol) which had been cooled to 0 °C. Bromide **8b** (390 mg, 1.0 mmol) in DMF (7.5 mL) was added dropwise to the stirred suspension of sodium hydride in DMF at 0 °C over 10 min. The reaction mixture was stirred at 25 °C for 16 h. Saturated aq. NH_4Cl (40 mL) was added followed by EtOAc (40 mL). The layers were separated and the aqueous layer was extracted with EtOAc (3 x 40 mL). The organic extracts were combined, dried (Na_2SO_4) and concentrated *in vacuo*. Purification by flash chromatography (20% EtOAc in hexanes) afforded oxetane **9b** as a colourless oil (201 mg, 65%); R_f = 0.25 (20% EtOAc in hexanes); IR (film) / cm^{-1} 2983, 2934, 1741 (C=O), 1599, 1587, 1496, 1448, 1369, 1289, 1246, 1134, 1077, 1028, 1016, 889, 856, 754, 692; ^1H NMR (400 MHz, $CDCl_3$) δ 7.32–7.25 (m, 2 H, 2 x Ph-H), 7.00–6.94 (m, 1 H, Ph-H), 6.94–6.89 (m, 2 H, 2 x Ph-H), 5.11 (dddd, J = 7.9, 6.6, 4.2, 3.6 Hz, 1 H, $OCH(CH_2OPh)$), 4.38–4.23 (m, 4 H, 2 x $CO_2CH_2CH_3$), 4.20 (dd, J = 10.9, 3.6 Hz, 1 H, PhOC*H*H), 4.12 (dd, J = 10.9, 4.2 Hz, 1 H, PhOCH*H*), 3.20 (dd, J = 12.0, 6.6 Hz, 1 H, C*H*H), 3.14 (dd, J = 12.0, 7.9 Hz, 1 H, CH*H*), 1.33 (t, J = 7.1 Hz, 3 H, $CO_2CH_2CH_3$), 1.29 (t, J = 7.1 Hz, 3 H, $CO_2CH_2CH_3$); ^{13}C NMR (101 MHz, $CDCl_3$) δ 168.8 (CO_2), 168.4 (CO_2), 158.5 (Ph-C_q), 129.4 (2 x Ph-CH), 121.1 (Ph-CH), 114.6 (2 x Ph-CH), 81.7 ($C_q(CO_2Et)_2$), 76.2 ($OCH(CH_2OPh)$), 69.4 (CH_2OPh), 62.3 ($CO_2CH_2CH_3$), 62.1 ($CO_2CH_2CH_3$), 30.1 (CH_2), 14.0 (2 x $CO_2CH_2CH_3$); HRMS (ESI-TOF) *m/z* Calcd for $C_{16}H_{20}NaO_6^+$ $[M+Na]^+$: 331.1152, Found: 331.1145.

(±)-1,3-Diethyl 2-[(1,3-dibromopropan-2-yl)oxy]propanedioate (8c)

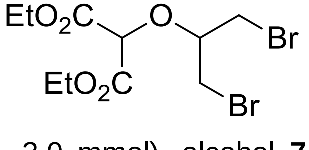

A mixture of diazo malonate **1** (559 mg, 3.0 mmol), alcohol **7c** (434 mg, 2.0 mmol) and dirhodium(II) tetraacetate (4.5 mg, 0.01 mmol) in benzene (20 mL) was heated at 80 °C for 3 d. The reaction mixture was allowed to cool to rt. Water (20 mL) was added and the layers were separated. The aqueous layer was extracted with $CHCl_3$ (3 x 20 mL). The organic extracts were combined, dried (Na_2SO_4) and concentrated *in vacuo*. Purification by flash chromatography (10% EtOAc in hexanes) afforded bromide **8c** as a colourless oil (380 mg, 51%); R_f = 0.38 (20% EtOAc in hexanes); IR (film) / cm^{-1} 2983, 1740 (C=O), 1421, 1370, 1232, 1179, 1126, 1026, 857, 668, 604, 553, 546; ^1H NMR (400 MHz, $CDCl_3$) δ 4.75 (s, 1 H, $CH(CO_2Et)_2$), 4.35–4.23 (m, 4 H, 2 x $CO_2CH_2CH_3$), 3.99–3.93 (m, 1 H, $CH(CH_2Br)_2$), 3.68 (dd, J = 10.9, 4.9 Hz, 2 H, 2 x C*H*HBr), 3.66 (dd, J = 10.9, 5.8 Hz, 2 H, 2 x CH*H*Br), 1.31 (t, J = 7.1 Hz, 6 H, 2 x $CO_2CH_2CH_3$); ^{13}C NMR (101 MHz, $CDCl_3$) δ 166.0 (2 x CO_2), 79.4 ($OCH(CH_2Br)_2$), 79.0 ($(EtO_2C)_2CHO$), 62.3 (2 x $CO_2CH_2CH_3$), 32.0 (2 x CH_2Br), 14.0 (2 x $CO_2CH_2CH_3$); HRMS (ESI-TOF) *m/z* Calcd for $C_{12}H_{19}Br_2NNaO_5$ $[M+Na+MeCN]^+$: 437.9522, Found: 437.9534.

(±)-2,2-Diethyl 4-(bromomethyl)oxetane-2,2-dicarboxylate (9c)

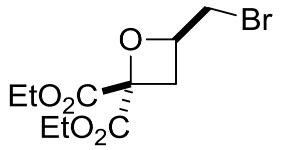

DMF (16.3 mL) was added to a flask containing sodium hydride (60% w/v, 27 mg, 0.7 mmol) which had been cooled to 0 °C. Bromide **8c** (189 mg, 0.5 mmol) in DMF (3.7 mL) was added dropwise to the stirred suspension of sodium hydride in DMF at 0 °C over 8 min. The reaction mixture was stirred at 25 °C for 16 h. Saturated aq. NH_4Cl (20 mL) was added followed by EtOAc (20 mL). The layers were separated and the aqueous layer was extracted with EtOAc (3 x 20 mL). The organic extracts were combined, dried (Na_2SO_4) and concentrated *in vacuo*. Purification by flash chromatography (20% EtOAc in hexanes) afforded oxetane **9c** as a colourless oil (120 mg, 81%); R_f = 0.25 (20% EtOAc in hexanes); IR (film) / cm^{-1} 2983, 1740 (C=O), 1446, 1369, 1260, 1130, 1094, 1057, 969, 856, 799, 758, 647; ^1H NMR (400 MHz, $CDCl_3$) δ 4.93–4.87 (m, 1 H, OC*H*(CH_2Br)), 4.31 (q, *J* = 7.1 Hz, 2 H, $CO_2CH_2CH_3$), 4.30 (q, *J* = 7.1 Hz, 2 H, $CO_2CH_2CH_3$), 3.61 (dd, *J* = 10.3, 5.6 Hz, 1 H, C*H*HBr), 3.55 (dd, *J* = 10.3, 7.6 Hz, 1 H, CH*H*Br), 3.15 (dd, *J* = 12.4, 7.6 Hz, 1 H, C*H*H), 2.89 (dd, *J* = 12.4, 6.4 Hz, 1 H, CH*H*), 1.310 (t, *J* = 7.1 Hz, 3 H, $CO_2CH_2CH_3$), 1.307 (t, *J* = 7.1 Hz, 3 H, $CO_2CH_2CH_3$); ^{13}C NMR (101 MHz, $CDCl_3$) δ 168.4 (CO_2), 168.2 (CO_2), 80.6 ($C_q(CO_2Et)_2$), 76.1 (O*C*H(CH_2Br)), 62.4 (2 x $CO_2CH_2CH_3$), 34.3 (CH_2Br), 33.0 (CH_2), 14.0 (2 x $CO_2CH_2CH_3$); HRMS (ESI-TOF) *m/z* Calcd for $C_{10}H_{15}BrO_5^+$ $[M+H]^+$: 295.0176, Found: 295.0190.

(±)-1,3-Diethyl 2-[(1-bromo-3-chloropropan-2-yl)oxy]propanedioate (8d)

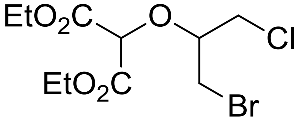

A mixture of diazo malonate **1** (839 mg, 4.5 mmol), alcohol **7d** (534 mg, 3.0 mmol) and dirhodium(II) tetraacetate (7.0 mg, 0.016 mmol) in benzene (30 mL) was heated at 80 °C for 90 min. The reaction mixture was allowed to cool to rt. Water (30 mL) was added and the layers were separated. The aqueous layer was extracted with $CHCl_3$ (3 x 30 mL). The organic extracts were combined, dried (Na_2SO_4) and concentrated *in vacuo*. Purification by flash chromatography (10% EtOAc in hexanes) afforded bromide **8d** as a colourless oil (814 mg, 80%); R_f = 0.32 (20% EtOAc in hexanes); IR (film) / cm^{-1} 2983, 1740 (C=O), 1467, 1445, 1370, 1233, 1179, 1123, 1027, 858, 756, 670, 601; ^1H NMR (400 MHz, $CDCl_3$) δ 4.76 (s, 1 H, $CH(CO_2Et)_2$), 4.35–4.23 (m, 4 H, 2 x $CO_2CH_2CH_3$), 3.99–3.94 (m, 1 H, C*H*(CH_2Cl)), 3.82 (dd, *J* = 11.7, 5.0 Hz, 1 H, C*H*HCl), 3.80 (dd, *J* = 11.7, 5.7 Hz, 1 H, CH*H*Cl) 3.68–3.60 (m, 2 H, CH_2Br), 1.31 (t, *J* = 7.1 Hz 6 H, 2 x $CO_2CH_2CH_3$); ^{13}C NMR (101 MHz, $CDCl_3$) δ 166.03 (CO_2), 166.00 (CO_2), 79.8 (OC*H*(CH_2Cl)), 79.1 ($(EtO_2C)_2$*C*HO), 62.3 (2 x $CO_2CH_2CH_3$), 44.1 (CH_2Cl), 31.1 (CH_2Br), 14.0 (2 x $CO_2CH_2CH_3$); HRMS (ESI-TOF) *m/z* Calcd for $C_{12}H_{19}NO_5ClBrNa^+$ $[M+CH_3CN+Na]^+$: 394.0033, Found: 394.0046.

(±)-1,3-Diethyl 2-[(1,3-dichloropropan-2-yl)oxy]propanedioate (8e)

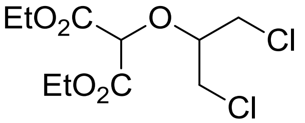

A mixture of diazo malonate **1** (565 mg, 3.0 mmol), alcohol **7e** (258 mg, 2.0 mmol) and dirhodium(II) tetraacetate (4.8 mg, 0.011 mmol) in benzene (20 mL) was heated at 80 °C for 2 h. The reaction mixture was allowed to cool to rt. Water (20 mL) was added and the layers were separated. The aqueous layer was extracted with $CHCl_3$ (3 x 20 mL). The organic extracts were combined, dried (Na_2SO_4) and concentrated *in vacuo*. Purification by flash chromatography (10% EtOAc in hexanes) afforded bromide **8e** as a colourless oil (488 mg, 86%); R_f = 0.33 (20% EtOAc in hexanes); IR (film) / cm^{-1} 2984, 2942, 1739 (C=O), 1467, 1445, 1369, 1234, 1179, 1123, 1023, 870, 758, 697, 605; ^1H NMR (400 MHz, $CDCl_3$) δ 4.76 (s, 1 H, $CH(CO_2Et)_2$),

4.36–4.21 (m, 4 H, 2 x $CO_2CH_2CH_3$),4.00–3.92 (m, 1 H, OC*H*(CH_2Cl)$_2$), 3.81–3.77 (m, 4 H, 2 x CH_2Cl), 1.31 (t, *J* = 7.1 Hz, 6 H, 2 x $CO_2CH_2CH_3$); ^{13}C NMR (101 MHz, $CDCl_3$) δ 166.0 (2 x CO_2), 80.1 (O*C*H(CH_2Cl)$_2$), 79.1 ((EtO_2C)$_2$*C*HO), 62.3 (2 x $CO_2CH_2CH_3$), 43.2 (2 x CH_2Cl), 14.0 (2 x $CO_2CH_2CH_3$); HRMS (CI) *m/z* Calcd for $C_{10}H_{20}Cl_2NO_5^+$ [M+Na+MeCN]$^+$: 304.0719, Found: 304.0726.

(±)-2,2-Diethyl 4-(chloromethyl)oxetane-2,2-dicarboxylate (9d)

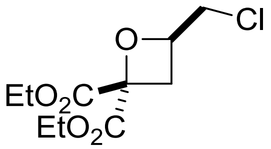

Method A – From Bromide **8d**:

DMF (32.5 mL) was added to a flask containing sodium hydride (60% w/v, 49 mg, 1.2 mmol) which had been cooled to 0 °C. Bromide **8d** (332 mg, 1.0 mmol) in DMF (7.5 mL) was added dropwise to the stirred suspension of sodium hydride in DMF at 0 °C over 10 min. The reaction mixture was stirred at 25 °C for 16 h. Saturated aq. NH_4Cl (40 mL) was added followed by EtOAc (40 mL). The layers were separated and the aqueous layer was extracted with EtOAc (3 x 40 mL). The organic extracts were combined, dried (Na_2SO_4) and concentrated *in vacuo*. Purification by flash chromatography (30% EtOAc in hexanes) afforded oxetane **9c (Br)** as a colourless oil (19 mg, 7%) followed by oxetane **9d (Cl)** as a colourless oil (113 mg, 45%); R_f = 0.19 (20% EtOAc in hexanes); IR (film) / cm^{-1} 2984, 1741 (C=O), 1447, 1370, 1258, 1133, 1098, 1061, 1048, 1013, 987, 856, 801, 731, 668, 553; ^{1}H NMR (400 MHz, $CDCl_3$) δ 5.11 (ddt, *J* = 7.6, 6.5, 5.6 Hz, 1 H, OC*H*(CH_2Cl)), 4.31 (q, *J* = 7.1 Hz, 2 H, $CO_2CH_2CH_3$), 4.30 (q, *J* = 7.1 Hz, 2 H, $CO_2CH_2CH_3$), 3.72 (d, *J* = 5.6 Hz, 2 H, CH_2Cl), 3.13 (dd, *J* = 12.4, 7.6 Hz, 1 H, C*H*H), 2.97 (dd, *J* = 12.4, 6.5 Hz, 1 H, CH*H*), 1.31 (t, *J* = 7.1 Hz, 3 H, $CO_2CH_2CH_3$), 1.30 (t, *J* = 7.1 Hz, 3 H, $CO_2CH_2CH_3$); ^{13}C NMR (101 MHz, $CDCl_3$) δ 168.4 (CO_2), 168.1 (CO_2), 81.0 (C_q(CO_2Et)$_2$), 76.4 (O*C*H(CH_2Cl)), 62.3 (2 x $CO_2CH_2CH_3$), 46.2 (CH_2Cl), 31.7 (CH_2), 14.0 (2 x $CO_2CH_2CH_3$); HRMS (ESI-TOF) *m/z* Calcd for $C_{10}H_{19}ClNO_5^+$ [M+NH_4]$^+$: 268.0946, Found: 268.0954.

Method B – From Chloride **8e**:

DMF (32.5 mL) was added to a flask containing sodium hydride (60% w/v, 49 mg, 1.2 mmol) which had been cooled to 0 °C. Chloride **8e** (287 mg, 1.0 mmol) in DMF (7.5 mL) was added dropwise to the stirred suspension of sodium hydride in DMF at 0 °C over 10 min. The reaction mixture was stirred at 25 °C for 16 h. Saturated aq. NH_4Cl (40 mL) was added followed by EtOAc (40 mL). The layers were separated and the aqueous layer was extracted with EtOAc (3 x 40 mL). The organic extracts were combined, dried (Na_2SO_4) and concentrated *in vacuo*. Purification by flash chromatography (20% EtOAc in hexanes) afforded oxetane **9d** as a colourless oil (193 mg, 77%).

(±)-1,3-Diethyl 2-{[1-chloro-3-(propan-2-yloxy)propan-2-yl]oxy}propanedioate (8f)

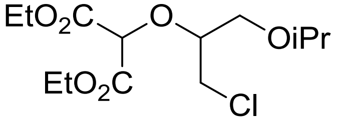

A mixture of diazo malonate **1** (555 mg, 3.0 mmol), alcohol **7f** (305 mg, 2.0 mmol) and dirhodium(II) tetraacetate (4.5 mg, 0.010 mmol) in benzene (20 mL) was heated at 80 °C for 90 min. The reaction mixture was allowed to cool to rt. Water (20 mL) was added and the layers were separated. The aqueous layer was extracted with $CHCl_3$ (2 x 20 mL). The organic extracts were combined, dried (Na_2SO_4) and concentrated *in vacuo*. Purification by flash chromatography (10% EtOAc in hexanes) afforded chloride **8f** as a colourless oil (600 mg, 97%); R_f = 0.56 (30% EtOAc in hexanes); IR (film) / cm^{-1} 2976, 2939, 2874, 1742 (C=O), 1468, 1370, 1229, 1176, 1125, 1095, 1026, 956, 857, 825, 750, 694, 591; ^{1}H NMR (400 MHz, $CDCl_3$) δ 4.89 (s, 1 H, C*H*(CO_2Et)$_2$), 4.33–4.21 (m, 4 H, 2 x $CO_2CH_2CH_3$), 3.92–3.84 (m, 1 H, OC*H*(CH_2Cl)),

3.70 (d, *J* = 5.5 Hz, 2 H, CH_2Cl), 3.67–3.55 (m, 3 H, OC*H*(CH_3)$_2$ and CH_2O^iPr), 1.31 (t, *J* = 7.1 Hz, 6 H, 2 x $CO_2CH_2CH_3$), 1.14 (d, *J* = 6.1 Hz, 6 H, CH(C*H*$_3$)$_2$); ^{13}C NMR (101 MHz, $CDCl_3$) δ 166.6 (CO_2), 166.5 (CO_2), 79.9 (OCH(CH_2Cl)), 79.1 ((EtO_2C)$_2$CHO), 72.3 (OCH(CH_3)$_2$), 68.3 (CH_2O^iPr), 61.91 ($CO_2CH_2CH_3$), 61.85 ($CO_2CH_2CH_3$), 43.7 (CH_2Cl), 21.9 (CH_3), 21.8 (CH_3), 14.0 (2 x $CO_2CH_2CH_3$); HRMS (ESI-TOF) *m/z* Calcd for $C_{13}H_{23}ClO_6Na^+$ $[M+Na]^+$: 333.1081, Found: 333.1085.

(±)-2,2-Diethyl 4-[(propan-2-yloxy)methyl]oxetane-2,2-dicarboxylate (9f)

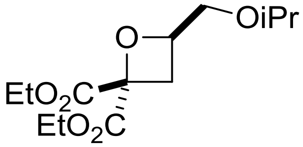

DMF (32.5 mL) was added to a flask containing sodium hydride (60% w/v, 48 mg, 1.2 mmol) which had been cooled to 0 °C. Chloride **8f** (311 mg, 1.0 mmol) in DMF (7.5 mL) was added dropwise to the stirred suspension of sodium hydride in DMF at 0 °C over 8 min. The reaction mixture was stirred at 25 °C for 16 h. Saturated aq. NH_4Cl (40 mL) was added followed by EtOAc (40 mL). The layers were separated and the aqueous layer was extracted with EtOAc (3 x 40 mL). The organic extracts were combined, dried (Na_2SO_4) and concentrated *in vacuo*. Purification by flash chromatography (25% EtOAc in hexanes) afforded oxetane **9f** as a colourless oil (205 mg, 75%); R_f = 0.28 (25% EtOAc in hexanes); IR (film) / cm^{-1} 2975, 2934, 1743 (C=O), 1467, 1448, 1369, 1276, 1257, 1132, 1075, 1019, 918, 879, 857, 801, 757, 687; ^{1}H NMR (400 MHz, $CDCl_3$) δ 4.90–4.82 (m, 1 H, OC*H*(CH_2O^iPr)), 4.33–4.23 (m, 4 H, 2 x $CO_2CH_2CH_3$), 3.69–3.62 (m, 1 H, OC*H*(CH_3)$_2$), 3.61–3.54 (m, 2 H, CH_2O^iPr), 3.02 (dd, *J* = 11.9, 7.6 Hz, 1 H, C*H*H), 2.99 (dd, *J* = 11.9, 6.9 Hz, 1 H, CH*H*), 1.30 (t, *J* = 7.1 Hz, 3 H, $CO_2CH_2CH_3$), 1.29 (t, *J* = 7.1 Hz, 3 H, $CO_2CH_2CH_3$), 1.15 (d, *J* = 6.1 Hz, 3 H, CH_3), 1.14 (d, *J* = 6.1 Hz, 3 H, CH_3); ^{13}C NMR (101 MHz, $CDCl_3$) δ 169.0 (CO_2), 168.6 (CO_2), 81.5 (C_q(CO_2Et)$_2$), 77.3 (OCH(CH_2O^iPr)), 72.2 (OCH(CH_3)$_2$), 69.9 (CH_2O^iPr), 62.1 ($CO_2CH_2CH_3$), 61.9 ($CO_2CH_2CH_3$), 30.2 (CH_2), 21.93 (CH_3), 21.89 (CH_3), 14.0 (2 x $CO_2CH_2CH_3$); HRMS (ESI-TOF) *m/z* Calcd for $C_{13}H_{22}O_6Na^+$ $[M+Na]^+$: 297.1314, Found: 297.1319.

(±)-1,3-Diethyl 2-({1-[(tert-butyldimethylsilyl)oxy]-3-chloropropan-2-yl}oxy)propanedioate (8g)

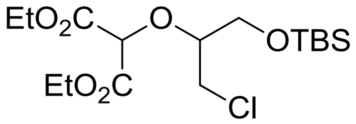

A mixture of diazo malonate **1** (582 mg, 3.2 mmol), alcohol **7g** (450 mg, 2.0 mmol) and dirhodium(II) tetraacetate (4.3 mg, 0.01 mmol) in benzene (20 mL) was heated at 80 °C for 90 min. The reaction mixture was allowed to cool to rt. Water (20 mL) was added and the layers were separated. The aqueous layer was extracted with CH_2Cl_2 (3 x 20 mL). The organic extracts were combined, dried (Na_2SO_4) and concentrated *in vacuo*. Purification by flash chromatography (5% EtOAc in hexanes) afforded chloride **8g** as a colourless oil (500 mg, 65%); R_f = 0.50 (20% EtOAc in hexanes); IR (film) / cm^{-1} 2956, 2931, 2858, 1764 (C=O), 1744 (C=O), 1472, 1447, 1370, 1253, 1231, 1180, 1125 (C-O), 1097 (C-O), 1029 (C-O), 939, 835, 777, 736 (C-Cl), 668; ^{1}H NMR (400 MHz, $CDCl_3$) δ 4.87 (s, 1 H, CH(CO_2Et)$_2$), 4.35–4.20 (m, 4 H, 2 x $CO_2CH_2CH_3$), 3.89–3.76 (m, 3 H, OC*H*(CH_2Cl) and CH_2OTBS), 3.75–3.66 (m, 2 H, CH_2Cl), 1.31 (t, *J* = 7.1 Hz, 3 H, $CO_2CH_2CH_3$), 1.30 (t, *J* = 7.1 Hz, 3 H, $CO_2CH_2CH_3$), 0.89 (s, 9 H, C(C*H*$_3$)$_3$), 0.08 (s, 3 H, OSi(C*H*$_3$)(C'H$_3$)(*t*Bu)), 0.07 (s, 3 H, OSi(CH_3)(C'*H*$_3$)(*t*Bu)); ^{13}C NMR (101 MHz, $CDCl_3$) δ 166.6 (CO_2), 166.4 (CO_2), 80.9 (OCH(CH_2Cl)), 79.1 ((EtO_2C)$_2$CHO), 63.2 (CH_2OTBS), 62.01 ($CO_2CH_2CH_3$), 61.97 ($CO_2CH_2CH_3$), 43.4 (CH_2Cl), 25.8 (C(CH$_3$)$_3$), 18.2 (C_q(CH_3)$_3$), 14.0 (2 x $CO_2CH_2CH_3$), –5.5 (OSi(CH$_3$)(C'H$_3$)(*t*Bu), –5.6 (OSi(CH_3)(C'H$_3$)(*t*Bu); HRMS (ESI-TOF) *m/z* Calcd for $C_{16}H_{31}ClO_6SiNa^+$ $[M+Na]^+$: 405.1476, Found: 405.1475.

(±)-2,2-Diethyl 4-{[(tert-butyldimethylsilyl)oxy]methyl}oxetane-2,2-dicarboxylate (9g)

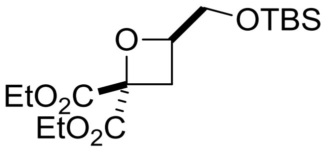

DMF (32.5 mL) was added to a flask containing sodium hydride (60% w/v, 48 mg, 1.2 mmol) which had been cooled to 0 °C. Chloride **8g** (383 mg, 1.0 mmol) in DMF (7.5 mL) was added dropwise to the stirred suspension of sodium hydride in DMF at 0 °C over 9 min. The reaction mixture was stirred at 25 °C for 16 h. Saturated aq. NH_4Cl (40 mL) was added followed by EtOAc (40 mL). The layers were separated and the aqueous layer was extracted with EtOAc (3 x 40 mL). The organic extracts were combined, dried (Na_2SO_4) and concentrated *in vacuo*. Purification by flash chromatography (10% EtOAc in hexanes) afforded oxetane **9g** as a colourless oil (246 mg, 71%); R_f = 0.40 (20% EtOAc in hexanes); IR (film) / cm^{-1} 2954, 2931, 2858, 1745 (C=O), 1473, 1448, 1390, 1369, 1253, 1141, 1098, 1078, 1018, 939, 909, 878, 834, 777, 669; ^1H NMR (400 MHz, $CDCl_3$) δ 4.85–4.78 (m, 1 H, OC*H*(CH_2OTBS)), 4.32–4.24 (m, 4 H, 2 x $CO_2CH_2CH_3$), 3.80 (dd, *J* = 11.8, 3.6 Hz, 1 H, C*H*HOTBS), 3.69 (dd, *J* = 11.8, 3.9 Hz, 1 H, CH*H*OTBS), 3.08 (dd, *J* = 11.8, 6.9 Hz, 1 H, C*H*H), 2.91 (dd, *J* = 11.8, 6.9 Hz, 1 H, CH*H*), 1.29 (t, *J* = 7.1 Hz, 3 H, $CO_2CH_2CH_3$), 1.28 (t, *J* = 7.1 Hz, 3 H, $CO_2CH_2CH_3$), 0.89 (s, 9 H, C(CH_3)$_3$), 0.07 (s, 6 H, OSi(CH_3)$_2$(*t*Bu)); ^{13}C NMR (101 MHz, $CDCl_3$) δ 169.1 (CO_2), 168.4 (CO_2), 81.3 (C_q(CO_2Et)$_2$), 78.2 (O*C*H(CH_2OTBS)), 64.9 (CH_2OTBS), 62.1 ($CO_2CH_2CH_3$), 61.8 ($CO_2CH_2CH_3$), 29.4 (CH_2), 25.8 (C(CH_3)$_3$), 18.4 (C_q(CH_3)$_3$), 13.98 ($CO_2CH_2CH_3$), 13.95 ($CO_2CH_2CH_3$), –5.4 (OSi(CH_3)(C'H_3)(*t*Bu), –5.5 (OSi(CH_3)(*C'*H_3)(*t*Bu); HRMS (ESI-TOF) *m/z* Calcd for $C_{16}H_{31}O_6Si^+$ $[M+H]^+$: 347.1890, Found: 347.1882.

(±)-1,3-Diethyl 2-[(3-bromo-1,1,1-trifluoropropan-2-yl)oxy]propanedioate (8h)

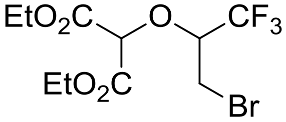

A solution of 3-bromo-1,1,1-trifluoropropan-2-one (0.62 mL, 6.0 mmol) in Et_2O (5 mL) was added dropwise to a mixture of $LiAlH_4$ (252 mg, 6.6 mmol) in Et_2O (5 mL) at rt over 6 min. The reaction mixture was stirred at rt for 90 min. Water (20 mL) was added followed by 1 M HCl (10 mL) and the mixture was extracted with Et_2O (3 x 20 mL). The organic extracts were combined, dried (Na_2SO_4), filtered through a short pad of silica and concentrated *in vacuo* to afford 3-bromo-1,1,1-trifluoropropan-2-ol which was used without further purification. *Note: Care must be taken when removing solvent as the product is also volatile.*

A mixture of diazo malonate **1** (1.67 g, 9.0 mmol), 3-bromo-1,1,1-trifluoropropan-2-ol (6.0 mmol) and dirhodium(II) tetraacetate (13.2 mg, 0.03 mmol) in benzene (60 mL) was heated at 80 °C for 2 h. The reaction mixture was allowed to cool to rt. Water (60 mL) was added and the layers were separated. The aqueous layer was extracted with CH_2Cl_2 (2 x 60 mL). The organic extracts were combined, dried (Na_2SO_4) and concentrated *in vacuo*. Purification by flash chromatography (5%–10% EtOAc in hexanes) afforded bromide **8h** as a colourless oil (581 mg, 28% over 2 steps); R_f = 0.60 (30% EtOAc in hexanes); IR (film) / cm^{-1} 2987, 1742 (C=O), 1467, 1448, 1391, 1371, 1274, 1247, 1168, 1126, 1024, 915, 859, 724, 648, 603, 557; ^1H NMR (400 MHz, $CDCl_3$) δ 4.78 (s, 1 H, CH(CO_2Et)$_2$), 4.33–4.23 (m, 5 H, OCH(CF_3) and 2 x $CO_2CH_2CH_3$), 3.59 (d, *J* = 5.9 Hz, 2 H, CH_2Br), 1.34–1.27 (m, 6 H, 2 x $CO_2CH_2CH_3$); ^{13}C NMR (101 MHz, $CDCl_3$) δ 165.3 (CO_2), 164.9 (CO_2), 123.2 (q, J_{CF} = 282 Hz, CF_3), 80.3 ((EtO_2C)$_2$*C*HO), 79.0 (q, J_{CF} = 31 Hz, O*C*H(CF_3)), 62.43 ($CO_2CH_2CH_3$), 62.35 ($CO_2CH_2CH_3$), 26.2 (CH_2Br), 14.0 ($CO_2CH_2CH_3$), 13.9 ($CO_2CH_2CH_3$); ^{19}F NMR (376 MHz, $CDCl_3$) δ –75.6; HRMS (ESI-TOF) *m/z* Calcd for $C_{10}H_{15}BrO_5F_3^+$ $[M+H]^+$: 351.0055, Found: 351.0060.

(±)-2,2-Diethyl 4-(trifluoromethyl)oxetane-2,2-dicarboxylate (9h)

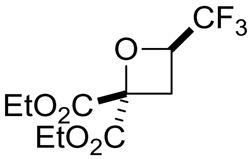

DMF (32.5 mL) was added to a flask containing sodium hydride (60% w/v, 49 mg, 1.2 mmol) which had been cooled to 0 °C. Bromide **8h** (351 mg, 1.0 mmol) in DMF (7.5 mL) was added dropwise to the stirred suspension of sodium hydride in DMF at 0 °C over 8 min. The reaction mixture was stirred at 25 °C for 16 h. Saturated aq. NH_4Cl (40 mL) was added followed by EtOAc (40 mL). The layers were separated and the aqueous layer was extracted with EtOAc (3 x 40 mL). The organic extracts were combined, dried (Na_2SO_4) and concentrated *in vacuo*. Purification by flash chromatography (10% EtOAc in hexanes) afforded oxetane **9h** as a colourless oil (117 mg, 43%); R_f = 0.38 (20% EtOAc in hexanes); IR (film) / cm^{-1} 2988, 1746 (C=O), 1450, 1398, 1371, 1283, 1160, 1129, 1054, 1013, 911, 885, 856, 785, 762, 655; ^{1}H NMR (400 MHz, $CDCl_3$) δ 4.98–4.87 (m, 1 H, OCH(CF_3)), 4.36–4.28 (m, 4 H, 2 x $CO_2CH_2CH_3$), 3.22 (dd, *J* = 12.0, 7.6 Hz, 1 H, C*H*H), 3.18 (dd, *J* = 12.0, 6.9 Hz, 1 H, CH*H*), 1.31 (t, *J* = 7.1 Hz, 6 H, 2 x $CO_2CH_2CH_3$); ^{13}C NMR (101 MHz, $CDCl_3$) δ 167.4 (CO_2), 166.8 (CO_2), 123.3 (q, J_{CF} = 280 Hz, CF_3), 82.1 ($C_q(CO_2Et)_2$), 73.0 (q, J_{CF} = 37 Hz, OCH(CF_3)), 62.8 ($CO_2CH_2CH_3$), 62.5 ($CO_2CH_2CH_3$), 28.2 (CH_2), 13.9 ($CO_2CH_2CH_3$); ^{19}F NMR (376 MHz, $CDCl_3$) δ –81.7; HRMS (ESI-TOF) *m/z* Calcd for $C_{10}H_{14}O_5F_3^+$ $[M+H]^+$: 271.0793, Found: 271.0795.

(±)-1,3-Diethyl 2-[(1-bromopropan-2-yl)oxy]propanedioate and (±)-1,3-Diethyl 2-(2-bromopropoxy)propanedioate (8i)

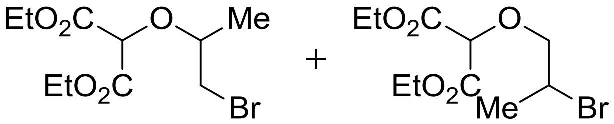

A mixture of diazo malonate **1** (558 mg, 3.0 mmol), 1-bromo-2-propanol (180 μL, 2.0 mmol, technical grade, 4:1 mixture of regioisomers) and dirhodium(II) tetraacetate (4.5 mg, 0.01 mmol) in benzene (20 mL) was heated at 80 °C for 90 min. The reaction mixture was allowed to cool to rt. Water (20 mL) was added and the layers were separated. The aqueous layer was extracted with $CHCl_3$ (3 x 20 mL). The organic extracts were combined, dried (Na_2SO_4) and concentrated *in vacuo*. Purification by flash chromatography (10% EtOAc in hexanes) afforded bromide **8i** as a colourless oil (576 mg, 98%, 4:1 mixture of regioisomers); R_f = 0.47 (30% EtOAc in hexanes); IR (film) / cm^{-1} 2983, 1739 (C=O), 1447, 1370, 1230, 1178, 1119, 1064, 1023, 934, 859, 664, 598; **Major Product (CH_2Br):** ^{1}H NMR (400 MHz, $CDCl_3$) δ 4.64 (s, 1 H, CH($CO_2Et)_2$), 4.33–4.19 (m, 4 H, 2 x $CO_2CH_2CH_3$), 3.91–3.81 (m, 1 H, OCH(Me)), 3.52 (dd, *J* = 10.5, 5.3 Hz, 1 H, C*H*HBr), 3.36 (dd, *J* = 10.5, 6.2 Hz, 1 H, CH*H*Br), 1.39 (d, *J* = 6.2 Hz, 3 H, CH_3), 1.30 (t, *J* = 7.1 Hz, 6 H, 2 x $CO_2CH_2CH_3$); ^{13}C NMR (101 MHz, $CDCl_3$) δ 166.7 (CO_2), 166.4 (CO_2), 78.1 (($EtO_2C)_2$CHO), 77.0 (OCH(Me)), 62.1 (2 x $CO_2CH_2CH_3$), 35.2 (CH_2Br), 19.0 (CH_3) 14.0 (2 x $CO_2CH_2CH_3$); **Minor Product (CH(Me)Br):** ^{1}H NMR (400 MHz, $CDCl_3$) δ 4.57 (s, 1 H, CH($CO_2Et)_2$), 4.33–4.19 (m, 4 H, 2 x $CO_2CH_2CH_3$), 3.91–3.81 (m, 1 H, OC*H*H), 3.76 (dd, *J* = 9.9, 7.4 Hz, 1 H, OCH*H*), 3.48–3.46 (m, 1 H, C*H*(Me)Br), 1.74 (d, *J* = 6.6 Hz, 3 H, CH_3), 1.30 (t, *J* = 7.1 Hz, 6 H, 2 x $CO_2CH_2CH_3$); ^{13}C NMR (101 MHz, $CDCl_3$) δ 166.7 (CO_2), 166.4 (CO_2), 79.2 (($EtO_2C)_2$CHO), 66.9 (OCH_2), 61.9 (2 x $CO_2CH_2CH_3$), 45.4 (CH(Me)Br), 22.5 (CH_3) 14.0 (2 x $CO_2CH_2CH_3$); HRMS (ESI-TOF) *m/z* Calcd for $C_{10}H_{17}BrO_5Na^+$ $[M+Na]^+$: 319.0157, Found: 319.0143.

(±)-2,2-Diethyl 4-methyloxetane-2,2-dicarboxylate and (±)-2,2-Diethyl 3-methyloxetane-2,2-dicarboxylate (9i)

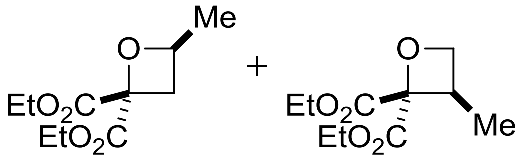

DMF (25 mL) was added to a flask containing sodium hydride (60% w/v, 33 mg, 0.8 mmol) which had been cooled to 0 °C. Bromide **8i** (202 mg, 0.7 mmol) in DMF (5 mL) was added dropwise to the stirred suspension of sodium hydride in DMF at 0 °C over 11 min. The reaction mixture was stirred at 25 °C for 16 h. Saturated aq. NH_4Cl (30 mL) was added followed by EtOAc (30 mL). The layers were separated and the aqueous layer was extracted with EtOAc (3 x 40 mL). The organic extracts were combined, dried (Na_2SO_4) and concentrated *in vacuo*. Purification by flash chromatography (10% EtOAc in hexanes) afforded oxetanes **9i** as a colourless oil (120 mg, 82%, 5.4:1 mixture of regioisomers); R_f = 0.31 (20% EtOAc in hexanes); IR (film) / cm^{-1} 2981, 1741 (C=O), 1447, 1382, 1369, 1259, 1231, 1179, 1124, 1079, 1031, 963, 873, 858, 790, 758, 685; **Major Product (4-Me):** ^1H NMR (400 MHz, $CDCl_3$) δ 5.01–4.89 (m, 1 H, OCH(Me)), 4.35–4.22 (m, 4 H, 2 x $CO_2CH_2CH_3$), 3.08 (dd, *J* = 11.7, 7.4 Hz, 1 H, C*H*H), 2.72 (dd, *J* = 11.7, 7.0 Hz, 1 H, CH*H*), 1.45 (d, *J* = 6.3 Hz, 3 H, CH_3), 1.29 (t, *J* = 7.1 Hz, 6 H, 2 x $CO_2CH_2CH_3$); ^{13}C NMR (101 MHz, $CDCl_3$) δ 169.2 (CO_2), 168.9 (CO_2), 80.7 ($C_q(CO_2Et)_2$), 75.5 (O*C*H(Me)), 62.04 ($CO_2CH_2CH_3$), 62.01 ($CO_2CH_2CH_3$), 35.0 (CH_2), 23.2 (CH_3), 14.0 ($CO_2CH_2CH_3$), 13.9 ($CO_2CH_2CH_3$); **Minor Product (3-Me):** ^1H NMR (400 MHz, $CDCl_3$) δ 4.69 (dd, 1 H, OC*H*H), 4.36–4.15 (m, 5 H, 2 x $CO_2CH_2CH_3$ and OCH*H*), 3.79–3.59 (m, 1 H, CH(Me)), 1.29 (t, *J* = 7.1 Hz, 6 H, 2 x $CO_2CH_2CH_3$), 1.20 (d, *J* = 7.1 Hz, 3 H, CH_3); ^{13}C NMR (101 MHz, $CDCl_3$) δ 167.6 (CO_2), 166.6 (CO_2), 79.1 ($C_q(CO_2Et)_2$), 74.9 (OCH_2), 62.0 ($CO_2CH_2CH_3$), 61.8 ($CO_2CH_2CH_3$), 35.5 (CH(Me)), 14.6 ($CO_2CH_2CH_3$), 14.2 ($CO_2CH_2CH_3$), 14.0 (CH_3); HRMS (ESI-TOF) *m/z* Calcd for $C_{12}H_{19}NO_5Na^+$ $[M+CH_3CN+Na]^+$: 280.1161, Found: 280.1175.

Synthesis of Tetrasubstituted Oxetanes 12a–12e

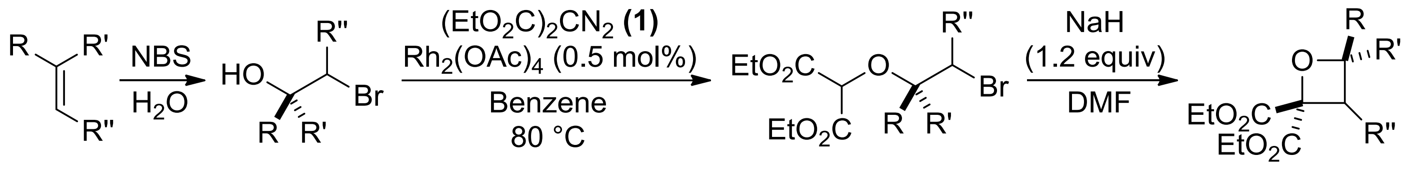

10a-10e **11a-11e** **12a-12e**

(±)-1-Bromo-2-phenylpropan-2-ol (10a)

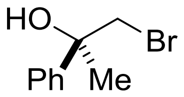

α-Methylstyrene (2.60 mL, 20 mmol) was added to a mixture of *N*-bromosuccinimide (2.60 g, 22 mmol) in water (20 mL). The reaction mixture was stirred at rt for 3 d. The reaction mixture was extracted with Et_2O (2 x 20 mL), the organic extracts were combined, washed with $Na_2S_2O_3$ (10 mL, 10% w/v), dried (Na_2SO_4) and concentrated *in vacuo*. Purification by flash chromatography (10% EtOAc in hexanes) afforded alcohol **10a** as a colourless oil (2.88 g, 67%); R_f = 0.52 (20% EtOAc in hexanes); IR (film) / cm^{-1} 3448 (br O-H), 3062, 3028, 2980, 1493, 1447, 1418, 1375, 1327, 1270, 1230, 1180, 1067, 1027, 941, 914, 870, 846, 764, 698, 663, 587, 570; ^{1}H NMR (400 MHz, $CDCl_3$) δ 7.53–7.45 (m, 2 H, 2 x Ph-H), 7.44–7.36 (m, 2 H, 2 x Ph-H), 7.36–7.28 (m, 1 H, Ph-H), 3.78 (d, *J* = 10.5 Hz, 1 H, C*H*HBr), 3.72 (d, *J* = 10.5 Hz, 1 H, CH*H*Br), 2.63 (br s, 1 H, OH), 1.70 (s, 3 H, CH_3); ^{13}C NMR (101 MHz, $CDCl_3$) δ 144.1 (Ph-C_q), 128.4 (2 x Ph-CH), 127.5 (Ph-CH), 124.8 (2 x Ph-CH), 73.1 (C_q(OH)), 46.2 (CH_2Br), 28.0 (CH_3).

Observed data (^{1}H, ^{13}C) was consistent with that previously reported.[5]

(±)-1,3-Diethyl 2-[(1-bromo-2-phenylpropan-2-yl)oxy]propanedioate (11a)

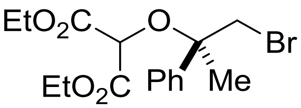

A mixture of diazo malonate **1** (556 mg, 3.0 mmol), alcohol **10a** (429 mg, 2.0 mmol) and dirhodium(II) tetraacetate (4.3 mg, 0.01 mmol) in benzene (20 mL) was heated at 80 °C for 2 h. The reaction mixture was allowed to cool to rt. Water (20 mL) was added and the layers were separated. The aqueous layer was extracted with $CHCl_3$ (2 x 20 mL). The organic extracts were combined, dried (Na_2SO_4) and concentrated *in vacuo*. Purification by flash chromatography (10% EtOAc in hexanes) afforded bromide **11a** as a colourless oil (529 mg, 71%); R_f = 0.40 (20% EtOAc in hexanes); IR (film) / cm^{-1} 2983, 2934, 2905, 1766 (C=O), 1741 (C=O), 1495, 1447, 1370, 1346, 1278, 1229, 1206, 1177, 1157, 1106, 1027, 920, 862, 846, 768, 701, 658, 620, 584, 527; ^{1}H NMR (400 MHz, $CDCl_3$) δ 7.46–7.41 (m, 2 H, 2 x Ph-H), 7.34–7.27 (m, 3 H, 3 x Ph-H), 4.26 (s, 1 H, $CH(CO_2Et)_2$), 4.24–4.15 (m, 2 H, $CO_2CH_2CH_3$), 4.15– 4.02 (m, 2 H, $CO_2CH_2CH_3$), 3.71 (d, *J* = 10.5 Hz, 1 H, C*H*HBr), 3.54 (d, *J* = 10.5 Hz, 1 H, CH*H*Br), 1.72 (s, 3 H, CH_3), 1.23 (t, *J* = 7.1 Hz, 3 H, $CO_2CH_2CH_3$), 1.17 (t, *J* = 7.1 Hz, 3 H, $CO_2CH_2CH_3$); ^{13}C NMR (101 MHz, $CDCl_3$) δ 167.7 (CO_2), 166.5 (CO_2), 140.0 (Ph-C_q), 128.6 (Ph-CH), 128.4 (2 x Ph-CH), 126.9 (2 x Ph-CH), 81.5 (OC_q(Ph)(Me)), 74.1 (($EtO_2C)_2$*C*HO), 61.8 (2 x $CO_2CH_2CH_3$), 41.8 (CH_2Br), 22.0 (CH_3), 13.9 ($CO_2CH_2CH_3$), 13.8 ($CO_2CH_2CH_3$); HRMS (ESI-TOF) *m/z* Calcd for $C_{16}H_{21}BrNaO_5^+$ $[M+Na]^+$: 395.0470, Found: 395.0484.

(±)-2,2-Diethyl 4-methyl-4-phenyloxetane-2,2-dicarboxylate (12a)

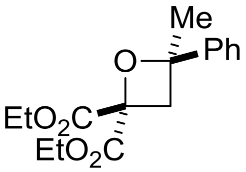

DMF (25 mL) was added to a flask containing sodium hydride (60% w/v, 36.5 mg, 0.91 mmol) which had been cooled to 0 °C. Bromide **11a** (280 mg, 0.75 mmol) in DMF (5 mL) was added dropwise to the stirred suspension of sodium hydride in DMF at 0 °C over 8 min. The reaction mixture was stirred at 25 °C for 24 h. Saturated aq. NH_4Cl (30 mL) was added followed by EtOAc (30 mL). The layers were separated and the aqueous layer was extracted with EtOAc (3 x 30 mL). The organic extracts were combined, dried (Na_2SO_4) and concentrated *in vacuo*. Purification by flash chromatography (20% EtOAc in hexanes) afforded oxetane **12a** as a colourless oil (123 mg, 56%); R_f = 0.34 (20% EtOAc in hexanes); IR (film) / cm^{-1} 2981, 1741 (C=O), 1495, 1446, 1369, 1275, 1258, 1201, 1175, 1141, 1105, 1074, 1046, 1026, 970, 882, 854, 764, 700, 555; ^1H NMR (400 MHz, $CDCl_3$) δ 7.44–7.33 (m, 4 H, 4 x Ph-H), 7.29–7.24 (m, 1 H, Ph-H), 4.36 (q, *J* = 7.1 Hz, 2 H, $CO_2CH_2CH_3$), 4.26–4.14 (m, 2 H, $CO_2CH_2CH_3$), 3.24 (d, *J* = 11.7 Hz, 1 H, C*H*H), 3.16 (d, *J* = 11.7 Hz, 1 H, CH*H*), 1.79 (s, 3 H, CH_3), 1.35 (t, *J* = 7.1 Hz, 3 H, $CO_2CH_2CH_3$), 1.21 (t, *J* = 7.1 Hz, 3 H, $CO_2CH_2CH_3$); ^{13}C NMR (101 MHz, $CDCl_3$) δ 169.3 (CO_2), 168.7 (CO_2), 146.1 (Ph-C_q), 128.2 (2 x Ph-CH), 127.1 (Ph-CH), 123.6 (2 x Ph-CH), 83.8 ($C_q(CO_2Et)_2$), 78.5 (OC_q(Me)(Ph)), 62.3 ($CO_2CH_2CH_3$), 62.0 ($CO_2CH_2CH_3$), 41.4 (CH_2), 30.5 (CH_3), 14.0 ($CO_2CH_2CH_3$), 13.9 ($CO_2CH_2CH_3$); HRMS (ESI-TOF) *m/z* Calcd for $C_{16}H_{21}O_5^+$ $[M+H]^+$: 293.1389, Found: 293.1389.

(±)-*trans*-2-Bromo-1,2-diphenylethan-1-ol (10b)

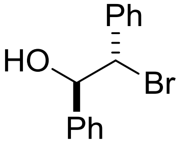

N-Bromosuccinimide (781 mg, 6.6 mmol) was added to a solution of *trans*-stilbene (1.08 g, 6.0 mmol) in acetone (24 mL) and water (6 mL). The reaction mixture was stirred at rt for 3 d. Water (6 mL) was added and the aqueous mixture was extracted with CH_2Cl_2 (3 x 30 mL). The organic extracts were combined, dried (Na_2SO_4) and concentrated *in vacuo*. Purification by flash chromatography (10% EtOAc in hexanes) afforded alcohol **10b** as a white solid (1.06 g, 64%); R_f = 0.36 (20% EtOAc in hexanes); mp = 82-84 °C (lit.[33] mp = 83 °C); IR (film) / cm^{-1} 3386 (br O-H), 3064, 3030, 1586, 1495, 1453, 1269, 1227, 1204, 1157, 1075, 1030, 994, 913, 859, 818, 758, 697, 638, 579; ^1H NMR (400 MHz, $CDCl_3$) δ 7.43–7.38 (m, 2 H, 2 x Ph-H), 7.37–7.28 (m, 8 H, 8 x Ph-H), 5.22 (d, *J* = 6.7 Hz, 1 H, C*H*(OH)), 5.12 (d, *J* = 6.7 Hz, 1 H, CHBr), 2.45 (br s, 1 H, OH); ^{13}C NMR (101 MHz, $CDCl_3$) δ 139.7 (Ph-C_q), 137.6 (Ph-C_q), 128.9 (2 x Ph-CH), 128.7 (Ph-CH), 128.4 (2 x Ph-CH), 128.3 (Ph-CH), 128.2 (2 x Ph-CH), 127.0 (2 x Ph-CH), 78.1 (CH(OH)), 58.9 (CHBr).

Observed data (IR, ^1H, ^{13}C) was consistent with that previously reported.[34]

(±)-*trans*-1,3-Diethyl 2-(2-bromo-1,2-diphenylethoxy)propanedioate (11b)

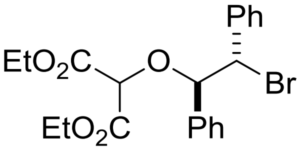

A mixture of diazo malonate **1** (554 mg, 3.0 mmol), alcohol **10b** (555 mg, 2.0 mmol) and dirhodium(II) tetraacetate (5.0 mg, 0.01 mmol) in benzene (20 mL) was heated at 80 °C for 90 min. The reaction mixture was allowed to cool to rt. Water (20 mL) was added and the layers were separated. The aqueous layer was extracted with $CHCl_3$ (2 x 20 mL). The organic extracts were combined, dried (Na_2SO_4) and concentrated *in vacuo*. Purification by flash chromatography (10% EtOAc in hexanes) afforded bromide **11b** as a pale yellow

oil (606 mg, 69%); R_f = 0.35 (20% EtOAc in hexanes); IR (film) / cm^{-1} 3032, 2983, 1758 (C=O), 1739 (C=O), 1495, 1455, 1370, 1285, 1232, 1178, 1156, 1113, 1027, 921, 858, 820, 763, 696, 600, 580; ^{1}H NMR (400 MHz, $CDCl_3$) δ 7.46–7.41 (m, 2 H, 2 x Ph-H), 7.34–7.22 (m, 8 H, 8 x Ph-H), 5.15 (d, *J* = 6.6 Hz, 1 H, CH(Ph)Br), 5.04 (d, *J* = 6.6 Hz, 1 H, OCH(Ph)), 4.34 (s, 1 H, $CH(CO_2Et)_2$), 4.22–4.14 (m, 2 H, $CO_2CH_2CH_3$), 4.13– 4.03 (m, 2 H, $CO_2CH_2CH_3$), 1.24 (t, *J* = 7.1 Hz, 3 H, $CO_2CH_2CH_3$), 1.15 (t, *J* = 7.2 Hz, 3 H, $CO_2CH_2CH_3$); ^{13}C NMR (101 MHz, $CDCl_3$) δ 166.2 (CO_2), 165.8 (CO_2), 137.9 (Ph-C_q), 136.7 (Ph-C_q), 129.1 (2 x Ph-CH), 128.9 (Ph-CH), 128.3 (Ph-CH), 128.2 (4 x Ph-CH), 128.0 (2 x Ph-CH), 85.8 (OCH(Ph)), 77.5 ($(EtO_2C)_2CHO$), 61.8 ($CO_2CH_2CH_3$), 61.7 ($CO_2CH_2CH_3$), 56.2 (CH(Ph)Br), 14.0 ($CO_2CH_2CH_3$), 13.8 ($CO_2CH_2CH_3$); HRMS (ESI-TOF) *m/z* Calcd for $C_{21}H_{23}BrO_6$ [M+H_2O]: 452.0834, Found: 452.0854.

(±)-*trans*-2,2-Diethyl 3,4-diphenyloxetane-2,2-dicarboxylate (12b)

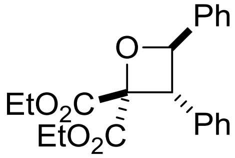

DMF (16 mL) was added to a flask containing sodium hydride (60% w/v, 25 mg, 0.6 mmol) which had been cooled to 0 °C. Bromide **11b** (216 mg, 0.5 mmol) in DMF (4 mL) was added dropwise to the stirred suspension of sodium hydride in DMF at 0 °C over 8 min. The reaction mixture was stirred at 25 °C for 48 h. Saturated aq. NH_4Cl (20 mL) was added followed by EtOAc (20 mL). The layers were separated and the aqueous layer was extracted with EtOAc (3 x 20 mL). The organic extracts were combined, dried (Na_2SO_4) and concentrated *in vacuo*. Purification by flash chromatography (10% EtOAc in hexanes) afforded oxetane **12b** as a colourless oil (61 mg, 34%); R_f = 0.18 (10% EtOAc in hexanes); IR (film) / cm^{-1} 2983, 1739 (C=O), 1498, 1454, 1390, 1368, 1275, 1206, 1174, 1116, 1062, 1044, 953, 907, 853, 756, 697, 591, 535; ^{1}H NMR (400 MHz, $CDCl_3$) δ 7.48–7.27 (m, 10 H, 10 x Ph-H), 6.21 (d, *J* = 8.2 Hz, 1 H, OCH(Ph)), 4.82 (d, *J* = 8.2 Hz, 1 H, CH(Ph)), 4.47–4.32 (m, 2 H, $CO_2CH_2CH_3$), 4.00–3.82 (m, 2 H, $CO_2CH_2CH_3$), 1.35 (t, *J* = 7.1 Hz, 3 H, $CO_2CH_2CH_3$), 0.85 (t, *J* = 7.1 Hz, 3 H, $CO_2CH_2CH_3$); ^{13}C NMR (101 MHz, $CDCl_3$) δ 168.0 (CO_2), 167.3 (CO_2), 140.1 (Ph-C_q), 134.2 (Ph-C_q), 128.6 (Ph-CH), 128.54 (2 x Ph-CH), 128.47 (2 x Ph-CH), 128.1 (2 x Ph-CH), 128.0 (Ph-CH), 125.7 (2 x Ph-CH), 86.3 ($C_q(CO_2Et)_2$), 82.6 (OCH(Ph)), 62.3 ($CO_2CH_2CH_3$), 61.7 ($CO_2CH_2CH_3$), 54.1 (CH(Ph)), 14.0 ($CO_2CH_2CH_3$), 13.5 ($CO_2CH_2CH_3$); HRMS (ESI-TOF) *m/z* Calcd for $C_{21}H_{23}O_5^+$ $[M+H]^+$: 355.1545, Found: 355.1561.

(±)-*trans*-2-Bromocyclohexan-1-ol (10c)

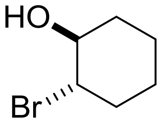

Cyclohexene (4.05 mL, 40 mmol) was added to a mixture of *N*-bromosuccinimide (4.95 g, 42 mmol) in water (40 mL). The mixture was stirred at rt for 21 h. After this time, the product was extracted with Et_2O (2 x 40 mL). The organic extracts were combined, washed with $Na_2S_2O_3$ (20 mL, 10% w/v), dried (Na_2SO_4) and concentrated *in vacuo* using a rotavap. Removal of remaining starting material and solvent by heating at 70 °C for 2 h afforded alcohol **10c** as a colourless oil (5.21 g, 73%); R_f = 0.16 (20% Et_2O in hexanes); IR (film) / cm^{-1} 3393 (br O-H), 2934, 2860, 1449, 1360, 1253, 1229, 1185, 1121, 1068, 1034, 1007, 955, 862, 841, 791, 687, 555; ^{1}H NMR (400 MHz, $CDCl_3$) δ 3.94–3.88 (m, 1 H, C*H*(OH)), 3.64–3.58 (m, 1 H, CHBr), 2.51 (br s, 1 H, OH), 2.39–2.33 (m, 1 H, Cy-CH), 2.18–2.12 (m, 1 H, Cy-CH), 1.90–1.79 (m, 2 H, 2 x Cy-CH), 1.73–1.68 (m, 1 H, Cy-CH), 1.44–1.24 (m, 3 H, 3 x Cy-CH); ^{13}C NMR (101 MHz, $CDCl_3$) δ 75.3 (CH(OH)), 61.9 (CHBr), 36.2 (Cy-CH_2), 33.5 (Cy-CH_2), 26.7 (Cy-CH_2), 24.1 (Cy-CH_2).

Observed data (IR, ^{1}H, ^{13}C) was consistent with that previously reported.[35]

(±)-*trans*-1,3-Diethyl 2-[(2-bromocyclohexyl)oxy]propanedioate (11c)

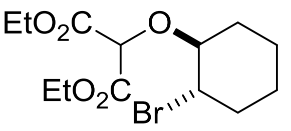

A mixture of diazo malonate **1** (558 mg, 3.0 mmol), alcohol **10c** (360 mg, 2.0 mmol) and dirhodium(II) tetraacetate (5.0 mg, 0.011 mmol) in benzene (20 mL) was heated at 80 °C for 90 min. The reaction mixture was allowed to cool to rt. Water (20 mL) was added and the layers were separated. The aqueous layer was extracted with $CHCl_3$ (3 x 20 mL). The organic extracts were combined, dried (Na_2SO_4) and concentrated *in vacuo*. Purification by flash chromatography (10% Et_2O in hexanes) afforded bromide **11c** as a colourless oil (552 g, 81%); R_f = 0.38 (20% EtOAc in hexanes); IR (film) / cm^{-1} 2939, 2864, 1764 (C=O), 1740 (C=O), 1448, 1369, 1226, 1188, 1135, 1104, 1026, 951, 858, 800, 671, 600, 585, 528; ^1H NMR (400 MHz, $CDCl_3$) δ 4.78 (s, 1 H, $CH(CO_2Et)_2$), 4.34–4.20 (m 4 H, 2 x $CO_2CH_2CH_3$), 4.13–4.06 (m, 1 H, CHBr), 3.57–3.52 (m, 1 H, CH(O)), 2.36–2.30 (m, 1 H, Cy-CH), 2.23–2.17 (m, 1 H, Cy-CH), 1.85–1.73 (m, 2 H, 2 x Cy-CH), 1.72–1.65 (m, 1 H, Cy-CH), 1.58–1.49 (m, 1 H, Cy-CH), 1.39–1.27 (m, 8 H, 2 x $CO_2CH_2CH_3$ and 2 x Cy-CH); ^{13}C NMR (101 MHz, $CDCl_3$) δ 167.2 (CO_2), 166.3 (CO_2), 83.6 (CH(O)), 79.2 ($(EtO_2C)_2CHO$), 61.9 (2 x $CO_2CH_2CH_3$), 54.7 (CHBr), 35.3 (Cy-CH_2), 31.1 (Cy-CH_2), 24.9 (Cy-CH_2), 23.1 (Cy-CH_2), 14.03 ($CO_2CH_2CH_3$), 14.01 ($CO_2CH_2CH_3$); HRMS (ESI-TOF) *m/z* Calcd for $C_{13}H_{21}BrNaO_5^+$ $[M+Na]^+$: 359.0465, Found: 359.0465.

(±)-*cis*-8,8-Diethyl 7-oxabicyclo[4.2.0]octane-8,8-dicarboxylate (12c)

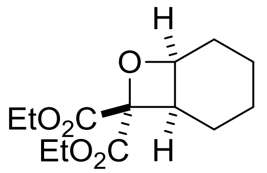

DMF (32.5 mL) was added to a flask containing sodium hydride (60% w/v, 48 mg, 1.2 mmol) which had been cooled to 0 °C. Bromide **11c** (337 mg, 1.0 mmol) in DMF (7.5 mL) was added dropwise to the stirred suspension of sodium hydride in DMF at 0 °C over 10 min. The reaction mixture was stirred at 25 °C for 16 h. Saturated aq. NH_4Cl (40 mL) was added followed by EtOAc (40 mL). The layers were separated and the aqueous layer was extracted with EtOAc (3 x 40 mL). The organic extracts were combined, dried (Na_2SO_4) and concentrated *in vacuo*. Purification by flash chromatography (20% EtOAc in hexanes) afforded oxetane **12c** as a colourless oil (202 mg, 79%); R_f = 0.25 (20% EtOAc in hexanes); IR (film) / cm^{-1} 2939, 2871, 1740 (C=O), 1449, 1368, 1267, 1210, 1174, 1136, 1083, 1049, 1024, 947, 857, 770, 667; ^1H NMR (400 MHz, $CDCl_3$) δ 5.00–4.96 (m, 1 H, OCH), 4.34–4.22 (m, 4 H, 2 x $CO_2CH_2CH_3$), 3.44 (dt, J = 8.0, 6.9 Hz, 1 H, CH), 1.98–1.88 (m, 1 H, Cy-CH), 1.84–1.75 (m, 1 H, Cy-CH), 1.74–1.57 (m, 4 H, 4 x Cy-CH), 1.46–1.36 (m, 1 H, Cy-CH), 1.32–1.20 (m, 7 H, 2 x $CO_2CH_2CH_3$ and Cy-CH); ^{13}C NMR (101 MHz, $CDCl_3$) δ 168.7 (CO_2), 167.9 (CO_2), 86.3 ($C_q(CO_2Et)_2$), 76.8 (OCH), 62.1 ($CO_2CH_2CH_3$), 61.6 ($CO_2CH_2CH_3$), 38.3 (CH), 27.7 (Cy-CH_2), 21.9 (Cy-CH_2), 19.9 (Cy-CH_2), 18.4 (Cy-CH_2), 14.2 ($CO_2CH_2CH_3$), 13.9 ($CO_2CH_2CH_3$); HRMS (ESI-TOF) *m/z* Calcd for $C_{13}H_{21}O_5^+$ $[M+H]^+$: 257.1389, Found: 257.1393.

(±)-*trans*-2-Bromocyclopentan-1-ol (10d)[36]

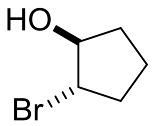

Cyclopentene (3.53 mL, 40 mmol) was added to a mixture of *N*-bromosuccinimide (4.96 g, 42 mmol) in water (40 mL). The mixture was stirred at rt for 21 h. After this time, the product was extracted with Et_2O (3 x 40 mL). The organic extracts were combined, washed with $Na_2S_2O_3$ (30 mL, 10% w/v), dried (Na_2SO_4) and concentrated *in vacuo* using a rotavap. Removal of remaining starting material and solvent by heating at 70 °C for 2 h afforded alcohol **10d** as a colourless oil (4.48 g, 68%); R_f = 0.55 (40% EtOAc in hexanes);

IR (film) / cm^{-1} 3341 (br O-H), 2971, 2877, 1436, 1335, 1302, 1197, 1130, 1101, 1070, 1033, 983, 896, 842, 605, 550; ^{1}H NMR (400 MHz, $CDCl_3$) δ 4.40–4.33 (m, 1 H, C*H*(OH)), 4.11–4.02 (m, 1 H, CHBr), 2.43–2.31 (m, 1 H, C*H*H), 2.24–2.09 (m, 2 H, CH*H* and OH), 2.07–1.95 (m, 1 H, CH*H*), 1.93–1.75 (m, 2 H, 2 x C*H*H), 1.65–1.63 (m, 1 H, CH*H*); ^{13}C NMR (101 MHz, $CDCl_3$) δ 80.5 (CH(OH)), 57.0 (CHBr), 33.7 (CH_2), 31.1 (CH_2), 21.0 (CH_2).

Observed data (IR, ^{1}H) was consistent with that previously reported.[37,38]

(±)-*trans*-1,3-Diethyl 2-[(2-bromocyclopentyl)oxy]propanedioate (11d)

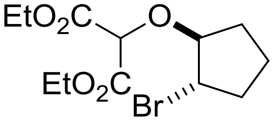

A mixture of diazo malonate **1** (588 mg, 3.2 mmol), alcohol **10d** (330 mg, 2.0 mmol) and dirhodium(II) tetraacetate (4.4 mg, 0.010 mmol) in benzene (20 mL) was heated at 80 °C for 90 min. The reaction mixture was allowed to cool to rt. Water (20 mL) was added and the layers were separated. The aqueous layer was extracted with $CHCl_3$ (3 x 20 mL). The organic extracts were combined, dried (Na_2SO_4) and concentrated *in vacuo*. Purification by flash chromatography (10% EtOAc in hexanes) afforded bromide **11d** as a colourless oil (486 mg, 75%); R_f = 0.36 (20% EtOAc in hexanes); IR (film) / cm^{-1} 2981, 2876, 1763 (C=O), 1740 (C=O), 1466, 1446, 1369, 1229, 1178, 1117, 1070, 1026, 972, 859, 710, 604; ^{1}H NMR (400 MHz, $CDCl_3$) δ 4.61 (s, 1 H, CH(CO_2Et)$_2$), 4.34–4.22 (m 6 H, 2 x $CO_2CH_2CH_3$ and CHBr and CH(O)), 2.44–2.31 (m, 1 H, C*H*H), 2.28–2.15 (m, 1 H, C*H*H), 2.07–1.96 (m, 1 H, C*H*H), 1.92–1.77 (m, 3 H, 3 x CH*H*), 1.31 (t, *J* = 7.2 Hz, 3 H, $CO_2CH_2CH_3$), 1.30 (t, *J* = 7.2 Hz, 3 H, $CO_2CH_2CH_3$); ^{13}C NMR (101 MHz, $CDCl_3$) δ 166.6 (CO_2), 166.5 (CO_2), 89.4 (CH(O)), 78.2 (($EtO_2C)_2$CHO), 62.10 ($CO_2CH_2CH_3$), 62.05 ($CO_2CH_2CH_3$), 53.6 (CHBr), 34.7 (CH_2), 29.9 (CH_2), 21.6 (CH_2), 14.0 (2 x $CO_2CH_2CH_3$); HRMS (ESI-TOF) *m/z* Calcd for $C_{12}H_{19}BrNaO_5^+$ [M+Na]$^+$: 345.0314, Found: 345.0331.

(±)-*cis*-7,7-Diethyl 6-oxabicyclo[3.2.0]heptane-7,7-dicarboxylate (12d)

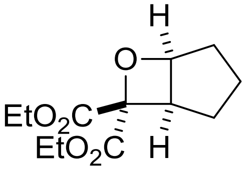

DMF (32.5 mL) was added to a flask containing sodium hydride (60% w/v, 48 mg, 1.2 mmol) which had been cooled to 0 °C. Bromide **11d** (324 mg, 1.0 mmol) in DMF (7.5 mL) was added dropwise to the stirred suspension of sodium hydride in DMF at 0 °C over 9 min. The reaction mixture was stirred at 25 °C for 16 h. Saturated aq. NH_4Cl (40 mL) was added followed by EtOAc (40 mL). The layers were separated and the aqueous layer was extracted with EtOAc (3 x 40 mL). The organic extracts were combined, dried (Na_2SO_4) and concentrated *in vacuo*. Purification by flash chromatography (20% EtOAc in hexanes) afforded oxetane **12d** as a colourless oil (188 mg, 77%); R_f = 0.28 (20% EtOAc in hexanes); IR (film) / cm^{-1} 2961, 2877, 1741 (C=O), 1449, 1369, 1295, 1272, 1236, 1185, 1189, 1110, 1069, 1021, 954, 918, 877, 772, 685; ^{1}H NMR (400 MHz, $CDCl_3$) δ 5.31–5.25 (m, 1 H, OCH), 4.35–4.22 (m, 4 H, 2 x $CO_2CH_2CH_3$), 3.65–3.57 (m, 1 H, CH), 2.12–1.94 (m, 3 H, 3 x C*H*H), 1.91–1.79 (m, 1 H, CH*H*), 1.66–1.50 (m, 1 H, CH*H*), 1.43–1.33 (m, 1 H, CH*H*), 1.30 (t, *J* = 7.2 Hz, 3 H, $CO_2CH_2CH_3$), 1.29 (t, *J* = 7.2 Hz, 3 H, $CO_2CH_2CH_3$); ^{13}C NMR (101 MHz, $CDCl_3$) δ 168.9 (CO_2), 167.3 (CO_2), 85.5 (OCH), 85.0 (C_q(CO_2Et)$_2$), 62.2 ($CO_2CH_2CH_3$), 61.5 ($CO_2CH_2CH_3$), 45.1 (CH), 33.8 (CH_2), 27.5 (CH_2), 24.2 (CH_2), 14.2 ($CO_2CH_2CH_3$), 13.9 ($CO_2CH_2CH_3$); HRMS (FTICR) *m/z* Calcd for $C_{12}H_{19}O_5^+$ [M+H]$^+$: 243.1227, Found: 243.1234.

(±)-*trans*-4-Bromooxolan-3-ol (10e)[39]

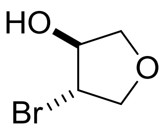

2,5-Dihydrofuran (3.0 mL, 40 mmol) was added to a mixture of *N*-bromosuccinimide (4.95 g, 42 mmol) in water (40 mL). The mixture was stirred at rt for 24 h. After this time, the product was extracted with Et_2O (3 x 40 mL). The organic extracts were combined, washed with $Na_2S_2O_3$ (50 mL, 10% w/v), dried (Na_2SO_4) and concentrated *in vacuo* using a rotavap. Removal of remaining starting material and solvent by heating at 70 °C for 1 h afforded alcohol **10e** as a yellow oil (3.48 g, 52%); IR (film) / cm^{-1} 3387 (br O-H), 2949, 2872, 1467, 1415, 1364, 1315, 1266, 1208, 1055, 956, 890, 784, 725, 634; ^1H NMR (400 MHz, $CDCl_3$) δ 4.64–4.55 (m, 1 H, C*H*(OH)), 4.43 (dd, *J* = 10.6, 4.7 Hz, 1 H, OC*H*H), 4.27 (dd, *J* = 10.0, 4.3 Hz, 1 H, OC'*H*H), 4.23–4.18 (m, 1 H, CHBr), 4.09 (dd, *J* = 10.6, 2.2 Hz, 1 H, OCH*H*), 3.86–3.78 (m, 1 H, OC'H*H*), 2.14–2.03 (m, 1 H, OH); ^{13}C NMR (101 MHz, $CDCl_3$) δ 78.6 (CH(OH)), 74.2 (OCH_2), 73.2 ($OC'H_2$) 51.4 (CHBr).

Observed data (^1H, ^{13}C) was consistent with that previously reported.[39]

(±)-*trans*-1,3-Diethyl 2-[(4-bromooxolan-3-yl)oxy]propanedioate (11e)

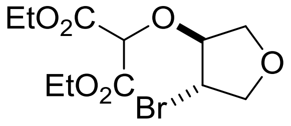

A mixture of diazo malonate **1** (560 mg, 3.0 mmol), alcohol **10d** (335 mg, 2.0 mmol) and dirhodium(II) tetraacetate (4.7 mg, 0.011 mmol) in benzene (20 mL) was heated at 80 °C for 90 min. The reaction mixture was allowed to cool to rt. Water (20 mL) was added and the layers were separated. The aqueous layer was extracted with $CHCl_3$ (2 x 20 mL). The organic extracts were combined, dried (Na_2SO_4) and concentrated *in vacuo*. Purification by flash chromatography (20% EtOAc in hexanes) afforded bromide **11e** as a colourless oil (491 mg, 75%); R_f = 0.26 (20% EtOAc in hexanes); IR (film) / cm^{-1} 2984, 1738 (C=O), 1467, 1370, 1232, 1179, 1123, 1093, 1024, 924, 903, 855, 832, 731, 635; ^1H NMR (400 MHz, $CDCl_3$) δ 4.64 (s, 1 H, $CH(CO_2Et)_2$), 4.48–4.44 (m, 1 H, CH(O)), 4.41–4.37 (m, 1 H, CHBr), 4.36–4.26 (m, 6 H, 2 x $CO_2CH_2CH_3$ and OC*H*H and OC'*H*H), 4.05 (dd, *J* = 10.6, 2.0 Hz, 1 H, OCH*H*), 3.96 (dd, *J* = 10.4, 1.8 Hz, 1 H, OC'H*H*), 1.31 (t, *J* = 7.1 Hz, 6 H, 2 x $CO_2CH_2CH_3$); ^{13}C NMR (101 MHz, $CDCl_3$) δ 166.1 (CO_2), 166.0 (CO_2), 87.3 (CH(O)), 78.1 ($(EtO_2C)_2CHO$), 74.4 (OCH_2), 71.8 ($OC'H_2$), 62.33 ($CO_2CH_2CH_3$), 62.32 ($CO_2CH_2CH_3$), 48.8 (CHBr), 14.0 (2 x $CO_2CH_2CH_3$); HRMS (ESI-TOF) *m/z* Calcd for $C_{11}H_{18}BrO_6^+$ $[M+H]^+$: 325.0287, Found: 325.0295.

(±)-*cis*-7,7-Diethyl 3,6-dioxabicyclo[3.2.0]heptane-7,7-dicarboxylate (12e)

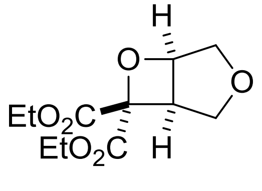

DMF (32.5 mL) was added to a flask containing sodium hydride (60% w/v, 48 mg, 1.2 mmol) which had been cooled to 0 °C. Bromide **11e** (326 mg, 1.0 mmol) in DMF (7.5 mL) was added dropwise to the stirred suspension of sodium hydride in DMF at 0 °C over 8 min. The reaction mixture was stirred at 25 °C for 16 h. Saturated aq. NH_4Cl (40 mL) was added followed by EtOAc (40 mL). The layers were separated and the aqueous layer was extracted with EtOAc (3 x 40 mL). The organic extracts were combined, dried (Na_2SO_4) and concentrated *in vacuo*. Purification by flash chromatography (40% EtOAc in hexanes) afforded oxetane **12e** as a colourless oil (206 mg, 92%); R_f = 0.29 (50% EtOAc in hexanes); IR (film) / cm^{-1} 2983, 1740 (C=O), 1467, 1447, 1369, 1270, 1228, 1175, 1124, 1079, 1061, 1040, 1016, 915, 846, 769, 719, 678; ^1H NMR (400

MHz, $CDCl_3$) δ 5.31 (dd, *J* = 5.7, 2.5 Hz, 1 H, OCH), 4.37–4.20 (m, 6 H, 2 x $CO_2CH_2CH_3$ and OC*H*H and OC'*H*H), 3.94–3.87 (m, 1 H, CH), 3.46 (dd, *J* = 10.9, 4.9 Hz, 1 H, OCH*H*), 3.35 (dd, *J* = 11.5, 2.5 Hz, OC'H*H*), 1.35–1.28 (m, 6 H, 2 x $CO_2CH_2CH_3$); ^{13}C NMR (101 MHz, $CDCl_3$) δ 168.1 (CO_2), 166.8 (CO_2), 84.1 ($C_q(CO_2Et)_2$), 82.6 (OCH), 73.4 (OC'H_2), 68.2 (OCH_2), 62.5 ($CO_2CH_2CH_3$), 62.0 ($CO_2CH_2CH_3$), 45.9 (CH), 14.1 ($CO_2CH_2CH_3$), 13.9 ($CO_2CH_2CH_3$); HRMS (ESI-TOF) *m/z* Calcd for $C_{11}H_{16}O_6Na^+$ $[M+Na]^+$: 267.0845, Found: 267.0851.

Functionalization of Oxetanes 6d and 9c

(±)-[4-(4-Chlorophenyl)-2-(hydroxymethyl)oxetan-2-yl]methanol (13)

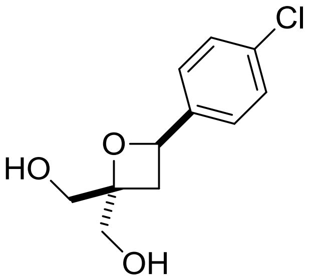

Sodium borohydride (37 mg, 0.97 mmol) was added in one portion to a mixture of oxetane **6d** (100 mg, 0.32 mmol) and LiCl (40 mg, 0.95 mmol) in THF (0.6 mL) and MeOH (1.2 mL) at 0 °C. The reaction mixture was stirred at 0 °C for a further 10 min and allowed to warm to rt and stirred for 17 h. Saturated aq. Rochelle salt (4 mL) was added and the resulting mixture was stirred for 20 min and the aqueous mixture was extracted with EtOAc (6 x 10 mL). The organic extracts were combined, dried (Na_2SO_4) and concentrated *in vacuo*. Purification by flash chromatography (2% MeOH in EtOAc) afforded oxetane **13** as a white crystalline solid (67 mg, 91%); R_f = 0.30 (EtOAc); mp = 125-126 °C; IR (film) / cm^{-1} 3348 (br O-H), 2917, 1412, 1389, 1269, 1229, 1089, 1048, 967, 869, 823, 718; ^{1}H NMR (400 MHz, $CDCl_3$) δ 7.36 (s, 4 H, 4 x Ar-H), 5.67–5.60 (m, 1 H, OCH(Ar)), 3.91–3.80 (m, 2 H, CH_2OH), 3.70 (dd, *J* = 12.3, 5.5 Hz, 1 H, C*H*HOH), 3.64 (dd, *J* = 12.3, 7.1 Hz, 1 H, CH*H*OH), 2.85 (dd, *J* = 11.2, 8.1 Hz, 1 H, C*H*H), 2.61 (dd, *J* = 11.2, 7.4 Hz, 1 H, CH*H*), 2.57–2.44 (m, 1 H, OH), 2.28–2.14 (m, 1 H, OH); ^{13}C NMR (101 MHz, $CDCl_3$) δ 140.9 (Ar-C_q-CH(O)), 133.7 (Ar-C_q-Cl), 128.8 (2 x Ar-CH), 126.6 (2 x Ar-CH), 84.4 (C_q(CH_2OH)$_2$), 76.1 (OCH(Ar)), 65.9 (CH_2OH), 65.6 (CH_2OH), 32.0 (CH_2). HRMS (APCI) *m/z* Calcd for $C_{11}H_{17}ClNO_3^+$ $[M+NH_4]^+$: 246.0889, Found: 246.0891.

(±)-[4-(4-chlorophenyl)-2-(hydroxymethyl)oxetan-2-yl]methyl 4-methylbenzene-1-sulfonate (14)

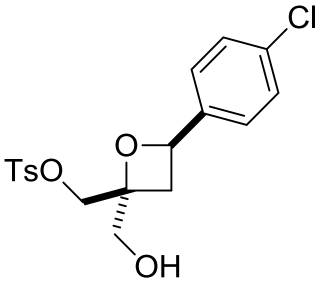

Major

n-Butyllithium (2.11 M in hexanes, 0.11 mL, 0.23 mmol) was added dropwise to a stirring solution of oxetane **13** (50 mg, 0.22 mmol) in THF (0.7 mL) at 0 °C. The reaction mixture was stirred at 0 °C for 1 h. A solution of 4-toluenesulfonyl chloride (44 mg, 0.23 mmol) in THF (0.5 mL) was added dropwise over 2 min. The reaction mixture was stirred at 30 °C for 22 h. Water (5 mL) was added and the resulting mixture was extracted with EtOAc (6 x 10 mL). The organic extracts were combined, dried (Na_2SO_4) and concentrated *in vacuo*. Purification by flash chromatography (50% EtOAc in hexanes) afforded minor oxetane **14a** as a pale yellow oil followed by major oxetane **14b** as a white crystalline solid (55 mg, 65%, d.r. 3.8:1).

Minor Product 14a: R_f = 0.48 (50% EtOAc in hexanes); IR (film) / cm^{-1} 3428 (br O-H), 1598, 1493, 1449, 1359, 1295, 1190, 1175, 1094, 1046, 977, 877, 823, 667, 556; ^{1}H NMR (400 MHz, $CDCl_3$) δ 7.91–7.82 (m, 2 H, 2 x Ts-H), 7.41–7.37 (m, 2 H, 2 x Ts-H), 7.36–7.33 (m, 2 H, 2 x Ar-H), 7.33–7.28 (m, 2 H, Ar-H), 5.61 (t, *J* = 7.7 Hz, 1 H, OCH(Ar)), 4.32 (d, *J* = 10.5 Hz, 1 H, C*H*HOTs), 4.23 (d, *J* = 10.5 Hz, 1 H, CH*H*OTs), 3.67 (d, *J* = 12.3 Hz, 1 H, C*H*HOH), 3.59 (d, *J* = 12.3 Hz, 1 H CH*H*OH), 2.90 (dd, *J* = 11.5, 7.9 Hz, 1 H, C*H*H), 2.60 (dd, *J* = 11.5, 7.6 Hz, 1 H, CH*H*), 2.47 (s, 3 H, Ts-CH_3), 1.83 (br s, 1 H, OH); ^{13}C NMR (101 MHz, $CDCl_3$) δ 145.2 (Ts-C_q-SO_2), 140.4 (Ar-C_q-CH(O)), 133.8 (Ar-C_q-Cl), 132.5 (Ts-C_q-Me), 130.0 (2 x Ts-CH),

128.8 (2 x Ar-CH), 128.0 (2 x Ts-CH), 126.6 (2 x Ar-CH), 81.7 (C_q(CH_2OH)(CH_2OTs)), 76.2 (OCH(Ar)), 70.9 (CH_2OTs), 65.4 (CH_2OH), 32.4 (CH_2), 21.7 (Ts-CH_3); HRMS (ESI-TOF) *m/z* Calcd for $C_{20}H_{22}ClNO_5SNa^+$ [M+CH_3CN+Na]$^+$: 446.0805, Found: 446.0827.

Major Product 14b: R_f = 0.22 (50% EtOAc in hexanes); mp = 103-105 °C; IR (film) / cm^{-1} 3382 (br O-H), 2925, 1597, 1490, 1452, 1402, 1365, 1294, 1232, 1189, 1177, 1082, 1014, 979, 959, 941, 871, 822, 810, 795, 770, 693, 663, 545; ^{1}H NMR (400 MHz, $CDCl_3$) δ 7.79–7.73 (m, 2 H, 2 x Ts-H), 7.38–7.31 (m, 2 H, 2 x Ts-H), 7.30–7.26 (m, 2 H, 2 x Ar-H), 7.25–7.21 (m, 2 H, Ar-H), 5.67 (t, *J* = 7.7 Hz, 1 H, OCH(Ar)), 4.14 (d, *J* = 10.8 Hz, 1 H, C*H*HOTs), 4.11 (d, *J* = 10.8 Hz, 1 H, CH*H*OTs), 3.86 (dd, *J* = 12.2, 6.0 Hz, 1 H, C*H*HOH), 3.77 (dd, *J* = 12.2, 5.6 Hz, 1 H CH*H*OH), 2.88 (dd, *J* = 11.6, 8.2 Hz, 1 H, C*H*H), 2.49 (dd, *J* = 11.6, 7.2 Hz, 1 H, CH*H*), 2.47 (s, 3 H, Ts-CH_3), 2.32–2.24 (m, 1 H, OH); ^{13}C NMR (101 MHz, $CDCl_3$) δ 145.2 (Ts-C_q-SO_2), 140.5 (Ar-C_q-CH(O)), 133.7 (Ar-C_q-Cl), 132.4 (Ts-C_q-Me), 129.9 (2 x Ts-CH), 128.6 (2 x Ar-CH), 128.0 (2 x Ts-CH), 126.5 (2 x Ar-CH), 81.5 (C_q(CH_2OH)(CH_2OTs)), 76.3 (OCH(Ar)), 70.7 (CH_2OTs), 64.9 (CH_2OH), 32.9 (CH_2), 21.7 (Ts-CH_3); HRMS (ESI-TOF) *m/z* Calcd for $C_{20}H_{22}ClNO_5SNa^+$ [M+CH_3CN+Na]$^+$: 446.0805, Found: 446.0819.

(±)-2-(4-Chlorophenyl)-1,6-dioxaspiro[3.3]heptane (15)

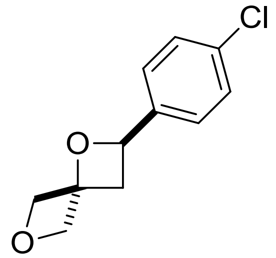

Sodium hydride (60% w/v, 5.2 mg, 0.13 mmol) was added to a stirring solution of oxetane **14** (41 mg, 0.11 mmol) in DMF (4.3 mL) at 0 °C. The reaction mixture was stirred at 0 °C for 10 min then at 25 °C for 20 h. Saturated aq. NH_4Cl (5 mL) was added followed by EtOAc (10 mL). The layers were separated and the aqueous layer was extracted with EtOAc (3 x 10 mL). The organic extracts were combined, dried (Na_2SO_4) and concentrated *in vacuo*. Purification by flash chromatography (40% EtOAc in hexanes) afforded oxetane **15** as a colourless oil (19 mg, 85%); R_f = 0.33 (40% EtOAc in hexanes); IR (film) / cm^{-1} 2937, 2865, 1598, 1492, 1447, 1441, 1339, 1298, 1246, 1164, 1126, 1089, 1015, 971, 878, 820, 765; ^{1}H NMR (400 MHz, $CDCl_3$) δ 7.38–7.32 (m, 2 H, 2 x Ar-H), 7.29–7.23 (m, 2 H, 2 x Ar-H), 5.53 (t, *J* = 7.1 Hz, 1 H, OCH(Ar)), 5.00 (dd, *J* = 7.7, 1.0 Hz, 1 H, OC*H*H), 4.97 (dd, *J* = 7.9, 0.9 Hz, 1 H, OC'*H*H), 4.86 (d, *J* = 7.7 Hz, 1 H, OCH*H*), 4.72 (d, *J* = 7.9 Hz, 1 H, OC'H*H*), 3.28 (dd, *J* = 12.0, 7.9 Hz, 1 H, C*H*H), 2.71 (dd, *J* = 12.0, 6.4 Hz, 1 H, CH*H*); ^{13}C NMR (101 MHz, $CDCl_3$) δ 140.9 (Ar-C_q-CH(O)), 133.7 (Ar-C_q-Cl), 128.8 (2 x Ar-CH), 126.3 (2 x Ar-CH), 85.0 (OC'H_2), 84.8 (OCH_2), 83.0 (C_q), 76.3 (OCH(Ar)), 39.5 (CH_2). HRMS (CI) *m/z* Calcd for $C_{11}H_{12}ClO_2^+$ [M+H]$^+$: 211.0526, Found: 211.0517.

(±)-Ethyl 2-(benzylcarbamoyl)-4-(4-chlorophenyl)oxetane-2-carboxylate (17)

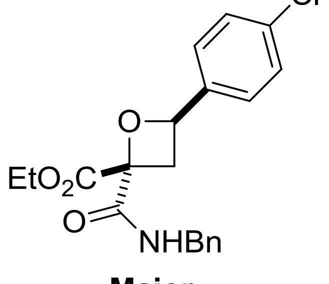

Major

A microwave vial (0.5–2.0 mL volume) was charged with oxetane **6d** (51 mg, 0.16 mmol). The reaction vial was flushed with argon, sealed with a cap and then further flushed with argon. Anhydrous ethanol (0.8 mL)

was added followed by 1N NaOH (176 μL, 0.18 mmol). The stirred mixture was stirred in an oil bath at 30 °C for 5 d. The reaction mixture was concentrated *in vacuo* to afford (±)-4-(4-chlorophenyl)-2-(ethoxycarbonyl)oxetane-2-carboxylate sodium salt **16** as a white solid (d.r. 3.1:1) which was used without further purification; **Major Product:** ^{1}H NMR (400 MHz, DMSO-d_6) 7.45–7.39 (m, 2 H, 2 x Ar-H), 7.39–7.35 (m, 2 H, 2 x Ar-H), 5.52 (t, *J* = 7.4 Hz, 1 H, OCH(Ar)), 4.12–4.05 (m, 2 H, $CO_2CH_2CH_3$), 3.07 (dd, *J* = 11.1, 7.9 Hz, 1 H, C*H*H), 2.62 (dd, *J* = 11.1, 7.0 Hz, 1 H, CH*H*), 1.17 (t, *J* = 7.1 Hz, 3 H, $CO_2CH_2CH_3$); **Minor Product:** ^{1}H NMR (400 MHz, DMSO-d_6) 7.73–7.68 (m, 2 H, 2 x Ar-H), 7.45–7.39 (m, 2 H, 2 x Ar-H), 5.41 (t, *J* = 7.6 Hz, 1 H, OCH(Ar)), 4.12–4.05 (m, 2 H, $CO_2CH_2CH_3$), 3.10 (dd, *J* = 11.3, 7.8 Hz, 1 H, C*H*H), 2.69 (dd, *J* = 11.3, 7.6 Hz, 1 H, CH*H*), 1.20 (t, *J* = 7.1 Hz, 3 H, $CO_2CH_2CH_3$).

HATU (70 mg, 0.18 mmol) was added to a flask containing (±)-4-(4-chlorophenyl)-2-(ethoxycarbonyl)oxetane-2-carboxylate sodium salt **16** (0.16 mmol) in DMF (2.5 mL). The reaction mixture was stirred at 30 °C for 3 h. Benzyl amine (28 μL, 0.25 mmol) was added in one portion and the reaction mixture was stirred at 40 °C for 24 h. The reaction mixture was concentrated *in vacuo*. Purification by flash chromatography (30% EtOAc in hexanes) afforded oxetane **17** as a pale yellow oil (29 mg, 48% over 2 steps, d.r. 2.9:1); R_f = 0.49 (50% EtOAc in hexanes); IR (film) / cm^{-1} 3337 (N-H), 2981, 2934, 1741 (C=O ester), 1669 (C=O amide), 1600, 1525, 1493, 1454, 1366, 1275, 1226, 1157, 1135, 1090, 1069, 1014, 951, 919, 855, 823, 729, 698, 599; **Major Product:** ^{1}H NMR (400 MHz, $CDCl_3$) δ 7.47–7.41 (m, 1 H, NH), 7.47–7.18 (m, 9 H, 4 x Ar-H and 5 x Ph-H), 5.61 (t, *J* = 7.7 Hz, 1 H, OCH(Ar)), 4.68 (dd, *J* = 14.9, 6.5 Hz, 1 H, CONHC*H*HPh), 4.53 (dd, *J* = 14.9, 5.6 Hz, 1 H, CONHCH*H*Ph), 4.39–4.28 (m, 2 H, $CO_2CH_2CH_3$), 3.34 (dd, *J* = 12.3, 7.6 Hz, 1 H, C*H*H), 3.23 (dd, *J* = 12.3, 7.9 Hz, 1 H, CH*H*), 1.33 (t, *J* = 7.1 Hz, 3 H, $CO_2CH_2CH_3$); ^{13}C NMR (101 MHz, $CDCl_3$) δ 169.1 (C=O), 168.2 (C=O), 138.9 (Ar-C_q-CH(O)), 137.51 (Ph-C_q), 134.6 (Ar-C_q-Cl), 128.9 (2 x Ph-CH), 128.8 (2 x Ar-CH), 127.7 (Ph-CH), 127.6 (2 x Ph-CH), 127.3 (2 x Ar-CH), 81.3 (*C_q*(CO_2Et)(CONHBn)), 78.31 (OCH(Ar)), 62.5 ($CO_2CH_2CH_3$), 43.2 (CONHCH_2Ph), 36.5 (CH_2), 14.0 ($CO_2CH_2CH_3$); **Minor Product:** ^{1}H NMR (400 MHz, $CDCl_3$) δ 7.47–7.18 (m, 9 H, 4 x Ar-H and 5 x Ph-H), 7.13–7.05 (m, 1 H, NH), 5.74 (t, *J* = 7.6 Hz, 1 H, OCH(Ar)), 4.62 (dd, *J* = 14.9, 6.8 Hz, 1 H, CONHC*H*HPh), 4.44 (dd, *J* = 14.9, 5.6 Hz, 1 H, CONHCH*H*Ph), 4.39–4.28 (m, 2 H, $CO_2CH_2CH_3$), 3.68 (dd, *J* = 12.3, 7.9 Hz, 1 H, C*H*H), 2.97 (dd, *J* = 12.3, 7.3 Hz, 1 H, CH*H*), 1.36 (t, *J* = 7.1 Hz, 3 H, $CO_2CH_2CH_3$); ^{13}C NMR (101 MHz, $CDCl_3$) δ 171.3 (C=O), 168.7 (C=O), 138.7 (Ar-C_q-CH(O)), 137.46 (Ph-C_q), 134.6 (Ar-C_q-Cl), 128.9 (2 x Ph-CH), 128.8 (2 x Ar-CH), 127.7 (Ph-CH), 127.6 (2 x Ph-CH), 127.4 (2 x Ar-CH), 81.4 (*C_q*(CO_2Et)(CONHBn)), 78.27 (OCH(Ar)), 62.6 ($CO_2CH_2CH_3$), 43.1 (CONHCH_2Ph), 35.8 (CH_2), 14.00 ($CO_2CH_2CH_3$); HRMS (ESI-TOF) *m/z* Calcd for $C_{20}H_{21}ClNO_4^+$ $[M+H]^+$: 374.1159, Found: 374.1174.

(±)-Ethyl 4-(4-chlorophenyl)-2-(morpholine-4-carbonyl)oxetane-2-carboxylate (18)

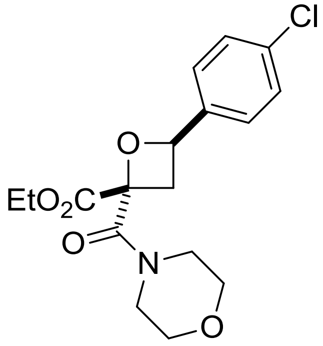

Major

A microwave vial (0.5–2.0 mL volume) was charged with oxetane **6d** (50 mg, 0.16 mmol). The reaction vial was flushed with argon, sealed with a cap and then further flushed with argon. Anhydrous ethanol (0.8 mL) was added followed by 1N NaOH (176 μL, 0.18 mmol). The stirred mixture was stirred in an oil bath at 30 °C for 4 d 12 h. The reaction mixture was concentrated *in vacuo* to afford (±)-4-(4-chlorophenyl)-2-(ethoxycarbonyl)oxetane-2-carboxylate sodium salt **16** as a white solid which was used without further purification.

HATU (71 mg, 0.19 mmol) was added to a flask containing (±)-4-(4-chlorophenyl)-2-(ethoxycarbonyl)oxetane-2-carboxylate sodium salt **16** (0.16 mmol) in DMF (2.5 mL). The reaction mixture was stirred at 30 °C for 3 h. Morpholine (22 μL, 0.25 mmol) was added in one portion and the reaction mixture was stirred at 40 °C for 24 h. The reaction mixture was concentrated *in vacuo*. Purification by flash chromatography (50% EtOAc in hexanes) afforded oxetane **18** as a pale yellow oil (23 mg, 40% over 2 steps, d.r. 2.5:1); R_f = 0.31 (50% EtOAc in hexanes); IR (film) / cm^{-1} 2976, 2909, 2855, 1745 (C=O ester), 1656 (C=O amide), 1493, 1440, 1367, 1262, 1159, 1115, 1091, 1066, 1028, 966, 828, 598; **Major Product:** ^1H NMR (400 MHz, $CDCl_3$) δ 7.53–7.47 (m, 2 H, 2 x Ar-H), 7.41–7.34 (m, 2 H, 2 x Ar-H), 5.65 (t, *J* = 7.6 Hz, 1 H, OCH(Ar)), 4.42–4.24 (m, 2 H, $CO_2CH_2CH_3$), 3.88 (dd, *J* = 11.9, 7.8 Hz, 1 H, C*H*H), 3.84–3.47 (m, 7 H, 2 x OCH_2 and NCH_2 and NC*H*H), 3.43–3.29 (m, 1 H, NCH*H*), 2.82 (dd, *J* = 11.9, 7.5 Hz, 1 H, CH*H*), 1.32 (t, *J* = 7.1 Hz, 3 H, $CO_2CH_2CH_3$); ^{13}C NMR (101 MHz, $CDCl_3$) δ 170.0 (C=O), 166.4 (C=O), 139.5 (Ar-C_q-CH(O)), 134.4 (Ar-C_q-Cl), 128.85 (2 x Ar-CH), 127.5 (2 x Ar-CH), 82.3 ($C_q(CO_2Et)(CON(CH_2)_2)$), 78.2 (OCH(Ar)), 66.8 (OCH_2), 66.6 (OCH_2), 62.4 ($CO_2CH_2CH_3$), 46.2 (NCH_2), 43.1 (NCH_2), 38.0 (CH_2), 14.2 ($CO_2CH_2CH_3$); **Minor Product:** ^1H NMR (400 MHz, $CDCl_3$) δ 7.41–7.34 (m, 2 H, 2 x Ar-H), 7.33–7.28 (m, 2 H, 2 x Ar-H), 5.71 (t, *J* = 7.7 Hz, 1 H, OCH(Ar)), 4.42–4.24 (m, 2 H, $CO_2CH_2CH_3$), 3.84–3.47 (m, 8 H, 2 x OCH_2 and NCH_2 and NC*H*H and C*H*H), 3.43–3.29 (m, 1 H, NCH*H*), 3.15 (dd, *J* = 12.2, 7.7 Hz, 1 H, CH*H*), 1.36 (t, *J* = 7.1 Hz, 3 H, $CO_2CH_2CH_3$); ^{13}C NMR (101 MHz, $CDCl_3$) δ 170.0 (C=O), 166.4 (C=O), 139.3 (Ar-C_q-CH(O)), 134.6 (Ar-C_q-Cl), 128.89 (2 x Ar-CH), 127.5 (2 x Ar-CH), 82.3 ($C_q(CO_2Et)(CON(CH_2)_2)$), 77.9 (OCH(Ar)), 66.9 (OCH_2), 66.7 (OCH_2), 62.5 ($CO_2CH_2CH_3$), 46.0 (NCH_2), 43.1 (NCH_2), 37.4 (CH_2), 14.3 ($CO_2CH_2CH_3$); HRMS (ESI-TOF) *m/z* Calcd for $C_{17}H_{21}ClNO_5^+$ $[M+H]^+$: 354.1108, Found: 354.1103.

(±)-Ethyl 4-(4-chlorophenyl)oxetane-2-carboxylate (19)

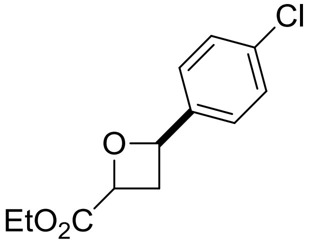

A microwave vial (0.5–2.0 mL volume) was charged with oxetane **6d** (50 mg, 0.16 mmol) and LiCl (7.7 mg, 0.18 mmol). The reaction vial was flushed with argon, sealed with a cap and then further flushed with argon. DMSO (0.56 mL) was then added. The stirred mixture was heated in an oil bath at 150 °C for 16 h and then cooled to rt, poured onto H_2O (20 mL) and extracted with EtOAc (6 x 5 mL). The organic extracts were combined, dried (Na_2SO_4) and concentrated *in vacuo*. Purification by flash chromatography (10% EtOAc in hexanes) afforded minor oxetane **19a (*trans*)** as a colourless oil, followed by major oxetane **19b (*cis*)** as a colourless oil (27 mg, 68%, 43:57 trans:cis).

Minor Product 19a (*trans*): R_f = 0.31 (20% EtOAc in hexanes); IR (film) / cm^{-1} 2979, 1746 (C=O), 1598, 1492, 1374, 1281, 1200, 1091, 1064, 996, 824; ^1H NMR (500 MHz, $CDCl_3$) δ 7.38 (s, 4 H, 4 x Ar-H), 5.86 (dd, *J* = 8.0, 7.0 Hz, 1 H, OCH(Ar)), 5.11 (dd, *J* = 9.5, 5.7 Hz, 1 H, OCH(CO_2Et)), 4.33 (q, *J* = 7.1 Hz, 2 H, $CO_2CH_2CH_3$), 3.09 (ddd, *J* = 11.6, 8.0, 5.7 Hz, 1 H, C*H*H), 2.91 (ddd, *J* = 11.6, 9.5, 7.0 Hz, 1 H, CH*H*), 1.36 (t, *J* = 7.1 Hz, 3 H, $CO_2CH_2CH_3$); ^{13}C NMR (126 MHz, $CDCl_3$) δ 172.5 (CO_2), 140.7 (Ar-C_q-CH(O)), 133.9 (Ar-C_q-Cl), 128.8 (2 x Ar-CH), 126.6 (2 x Ar-CH), 80.5 (OCH(Ar)), 74.7 (OCH(CO_2Et)), 61.4 ($CO_2CH_2CH_3$), 34.0 (CH_2), 14.2 ($CO_2CH_2CH_3$); HRMS (ESI-TOF) *m/z* Calcd for $C_{12}H_{14}ClO_3^+$ $[M+H]^+$: 241.0631, Found: 241.0634.

Major Product 19b (*cis*): R_f = 0.26 (20% EtOAc in hexanes); IR (film) / cm^{-1} 2983, 1736 (C=O), 1599, 1493, 1448, 1414, 1376, 1280, 1200, 1090, 1044, 1015, 992, 964, 824, 752; ^1H NMR (500 MHz, $CDCl_3$) δ 7.51–7.47 (m, 2 H, 2 x Ar-H), 7.37–7.35 (m, 2 H, 2 x Ar-H), 5.73 (t, *J* = 7.5 Hz, 1 H, OCH(Ar)), 5.21 (t, *J* = 8.2 Hz, 1 H, OCH(CO_2Et)), 4.35–4.23 (m, 2 H, $CO_2CH_2CH_3$), 3.28 (ddd, *J* = 11.5, 8.2, 7.5 Hz, 1 H, C*H*H), 2.72–2.65 (m, 1 H, CH*H*), 1.33 (t, *J* = 7.1 Hz, 3 H, $CO_2CH_2CH_3$); ^{13}C NMR (126 MHz, $CDCl_3$) δ 171.7 (CO_2), 140.5 (Ar-C_q-CH(O)), 134.1 (Ar-C_q-Cl), 128.7 (2 x Ar-CH), 127.3 (2 x Ar-CH), 79.3 (OCH(Ar)), 73.4

(O*C*H(CO_2Et)), 61.3 ($CO_2CH_2CH_3$), 34.6 (CH_2), 14.2 ($CO_2CH_2CH_3$); HRMS (ESI-TOF) *m/z* Calcd for $C_{12}H_{14}ClO_3^+$ $[M+H]^+$: 241.0631, Found: 241.0616.

(±)-2,2-Diethyl 4-(azidomethyl)oxetane-2,2-dicarboxylate (20)

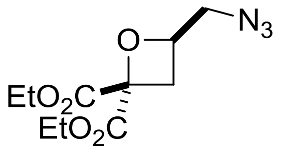

A microwave vial was charged with oxetane **9c** (80 mg, 0.27 mmol) and sodium azide (26 mg, 0.40 mmol). The reaction vial was flushed with argon, sealed with a cap and then further flushed with argon. Anhydrous DMF (5.42 mL) was added to the sealed vial. The stirred mixture was heated in an oil bath at 60 °C for 20 h and then cooled to rt and concentrated *in vacuo*. Water (10 mL) was added and the aqueous mixture was extracted with EtOAc (6 x 10 mL). The organic extracts were combined, dried (Na_2SO_4) and concentrated *in vacuo*. Purification by flash chromatography (30% EtOAc in hexanes) afforded oxetane **20** as a pale yellow oil (65 mg, 92%); R_f = 0.37 (30% EtOAc in hexanes); IR (film) / cm^{-1} 2983, 2934, 2098 (N=N=N), 1741 (C=O),1447, 1369, 1258, 1136, 1111, 1076, 1050, 1012, 919, 892, 856, 787, 756, 663, 557; ^{1}H NMR (400 MHz, $CDCl_3$) δ 4.98–4.89 (m, 1 H, OC*H*(CH_2N_3)) 4.35–4.27 (m, 4 H, 2 x $CO_2CH_2CH_3$), 3.57 (dd, *J* = 13.6, 3.7 Hz, 1 H, C*H*HN$_3$), 3.40 (dd, *J* = 13.6, 4.4 Hz, 1 H, CH*H*N$_3$), 3.04 (d, *J* = 7.2 Hz, 2 H, CH_2), 1.314 (t, *J* = 7.1 Hz, 3 H, $CO_2CH_2CH_3$), 1.306 (t, *J* = 7.1 Hz, 3 H, $CO_2CH_2CH_3$); ^{13}C NMR (101 MHz, $CDCl_3$) δ 168.4 (CO_2), 168.1 (CO_2), 81.3 (C_q(CO_2Et)$_2$), 76.4 (O*C*H(CH_2N_3)), 62.4 ($CO_2CH_2CH_3$), 62.3 ($CO_2CH_2CH_3$), 54.6 (CH_2N_3), 30.4 (CH_2), 14.0 (2 x $CO_2CH_2CH_3$); HRMS (ESI-TOF) *m/z* Calcd for $C_{10}H_{16}N_3O_5^+$ $[M+H]^+$: 258.1090, Found: 258.1090.

(±)-2,2-Diethyl 4-[(4-phenyl-1H-1,2,3-triazol-1-yl)methyl]oxetane-2,2-dicarboxylate (21)

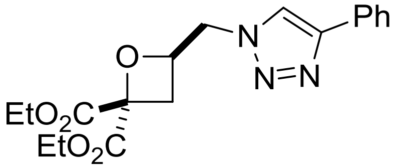

Copper(II) sulfate pentahydrate (6.1 mg, 0.024 mmol) and sodium ascorbate (14.6 mg, 0.074 mmol) were added to a solution of oxetane **20** (63 mg, 0.24 mmol) and phenylacetylene (32 mL, 0.29 mmol) in CH_2Cl_2 (2.4 mL) and water (2.4 mL). The resulting mixture was vigourously stirred in the dark at rt for 3 h. The reaction mixture was diluted with water (10 mL) and extracted with CH_2Cl_2 (4 x 10 mL). The orgnaic extracts were combined, dried (Na_2SO_4) and concentrated *in vacuo*. Purificiation by flash chromatography (40% EtOAc in hexanes) afforded oxetane **21** as a crystalline white solid (55 mg, 63%); R_f = 0.33 (60% EtOAc in hexanes); mp = 88–90 °C; IR (film) / cm^{-1} 2984, 1741 (C=O), 1611, 1465, 1443, 1391, 1369, 1284, 1262, 1232, 1135, 1069, 1041, 1000, 918, 856, 810, 766, 696, 564; ^{1}H NMR (400 MHz, $CDCl_3$) δ 8.03 (s, 1 H, NCH=C(Ph)), 7.91–7.83 (m, 2 H, 2 x Ph-H), 7.47–7.40 (m, 2 H, 2 x Ph-H), 7.37–7.31 (m, 1 H, Ph-H), 5.24–5.14 (m, 1 H, OC*H*(CH_2N)), 4.75 (dd, *J* = 14.9, 5.1 Hz, 1 H, OCH(C*H*HN)), 4.67 (dd, *J* = 14.9, 3.3 Hz, 1 H, OCH(CH*H*N)), 4.35–4.26 (m, 2 H, $CO_2CH_2CH_3$), 4.15–4.01 (m, 2 H, $CO_2CH_2CH_3$), 3.16 (dd, *J* = 12.4, 7.7 Hz, 1 H, C*H*H), 2.81 (dd, *J* = 12.4, 6.9 Hz, 1 H, CH*H*), 1.30 (t, *J* = 7.1 Hz, 3 H, $CO_2CH_2CH_3$), 1.16 (t, *J* = 7.1 Hz, 3 H, $CO_2CH_2CH_3$); ^{13}C NMR (101 MHz, $CDCl_3$) δ 168.1 (CO_2), 167.8 (CO_2), 148.0 (NCH=C_q(Ph)), 130.4 (Ph-C_q), 128.9 (2 x Ph-CH), 128.1 (Ph-CH), 125.7 (2 x Ph-CH), 121.6 (N*C*H=C(Ph), 81.1 (C_q(CO_2Et)$_2$), 76.0 (O*C*H(CH_2N)), 62.6 ($CO_2CH_2CH_3$), 62.4 ($CO_2CH_2CH_3$), 53.7 (OCH(CH_2N)), 30.4 (CH_2), 14.0 ($CO_2CH_2CH_3$), 13.8 ($CO_2CH_2CH_3$); HRMS (ESI-TOF) *m/z* Calcd for $C_{18}H_{22}N_3O_5^+$ $[M+H]^+$: 360.1559, Found: 360.1569.

(±)-2,2-Diethyl 4-(1H-imidazol-1-ylmethyl)oxetane-2,2-dicarboxylate (22)

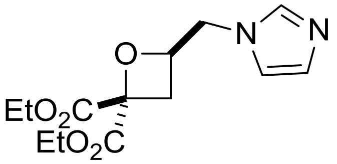

A microwave vial was charged with oxetane **9c** (65 mg, 0.22 mmol), imidazole (23 mg, 0.34 mmol), K_2CO_3 (37 mg, 0.27 mmol) and NaI (40 mg, 0.27 mmol). The reaction vial was flushed with argon, sealed with a cap and then further flushed with argon. Anhydrous DMF (0.87 mL) was added to the sealed vial. The stirred mixture was heated in an oil bath at 80 °C for 64 h and then cooled to rt and concentrated *in vacuo*. Purification by flash chromatography (3% MeOH in CH_2Cl_2) afforded oxetane **22** as a pale yellow oil (38 mg, 62%); R_f = 0.21 (5% MeOH in CH_2Cl_2); IR (film) / cm^{-1} 2984, 2938, 1742 (C=O), 1508, 1447, 1370, 1286, 1235, 1140, 1108, 1076, 1050, 1004, 915, 856, 744, 663, 622; ^1H NMR (400 MHz, $CDCl_3$) δ 7.54 (br s, 1 H, N-CH=N), 7.09 (br s, 1 H, *H*C=CH), 7.02 (br s, 1 H, HC=C*H*), 5.06–4.98 (m, 1 H, OC*H*(CH_2N)), 4.39–4.14 (m, 6 H, 2 x $CO_2CH_2CH_3$ and OCH(CH_2N)), 3.06 (dd, *J* = 12.2, 7.6 Hz, 1 H, C*H*H), 2.76 (dd, *J* = 12.2, 6.9 Hz, 1 H, CH*H*), 1.31 (t, *J* = 7.1 Hz, 3 H, $CO_2CH_2CH_3$), 1.30 (t, *J* = 7.1 Hz, 3 H, $CO_2CH_2CH_3$); ^{13}C NMR (101 MHz, $CDCl_3$) δ 168.4 (CO_2), 167.8 (CO_2), 81.0 ($C_q(CO_2Et)_2$), 76.3 (O*C*H(CH_2N)), 62.53 ($CO_2CH_2CH_3$), 62.46 ($CO_2CH_2CH_3$), 51.5 (OCH(CH_2N)), 30.5 (CH_2), 13.99 ($CO_2CH_2CH_3$), 13.96 ($CO_2CH_2CH_3$); HRMS (ESI-TOF) *m/z* Calcd for $C_{13}H_{19}N_2O_5^+$ $[M+H]^+$: 283.1294, Found: 283.1304.

Note: The resonance from the imidazole carbons in the ^{13}C NMR were very broad due to quadrupole coupling to ^{14}N nuclei, and thus extremely difficult to observe. In this case, the carbon resonances are not reported. The methylene CH_2 resonance α- to the imidazole was also very broad due to quadrupole coupling to ^{14}N nuclei. However, a clear correlation could be observed between the ^{13}C and ^1H atoms in the HSQC spectra (vide infra).

^{1}H and ^{13}C NMR spectra of selected compounds

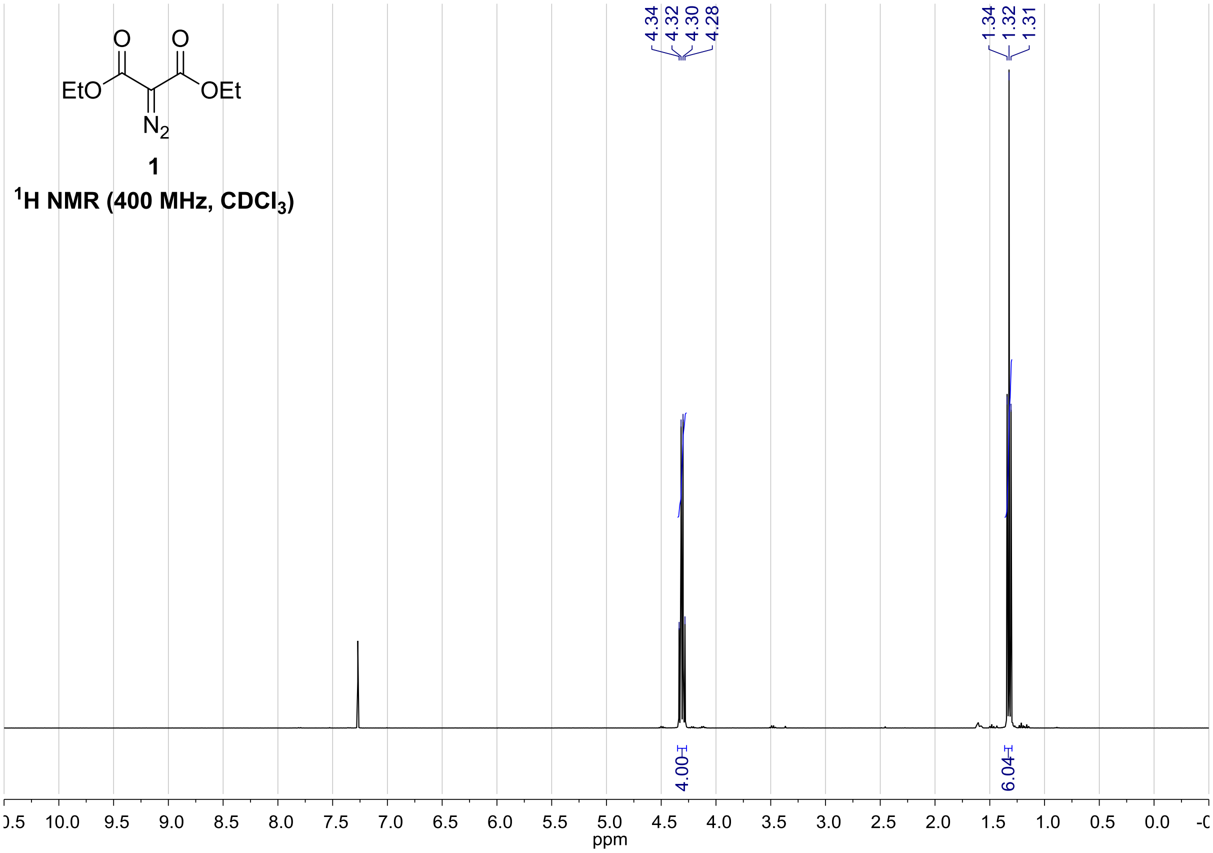

EtO
OEt
N2
1
1H NMR (400 MHz, CDCl3)
4.34
4.32
4.30
4.28
1.34
1.32
1.31
4.00
6.04
ppm

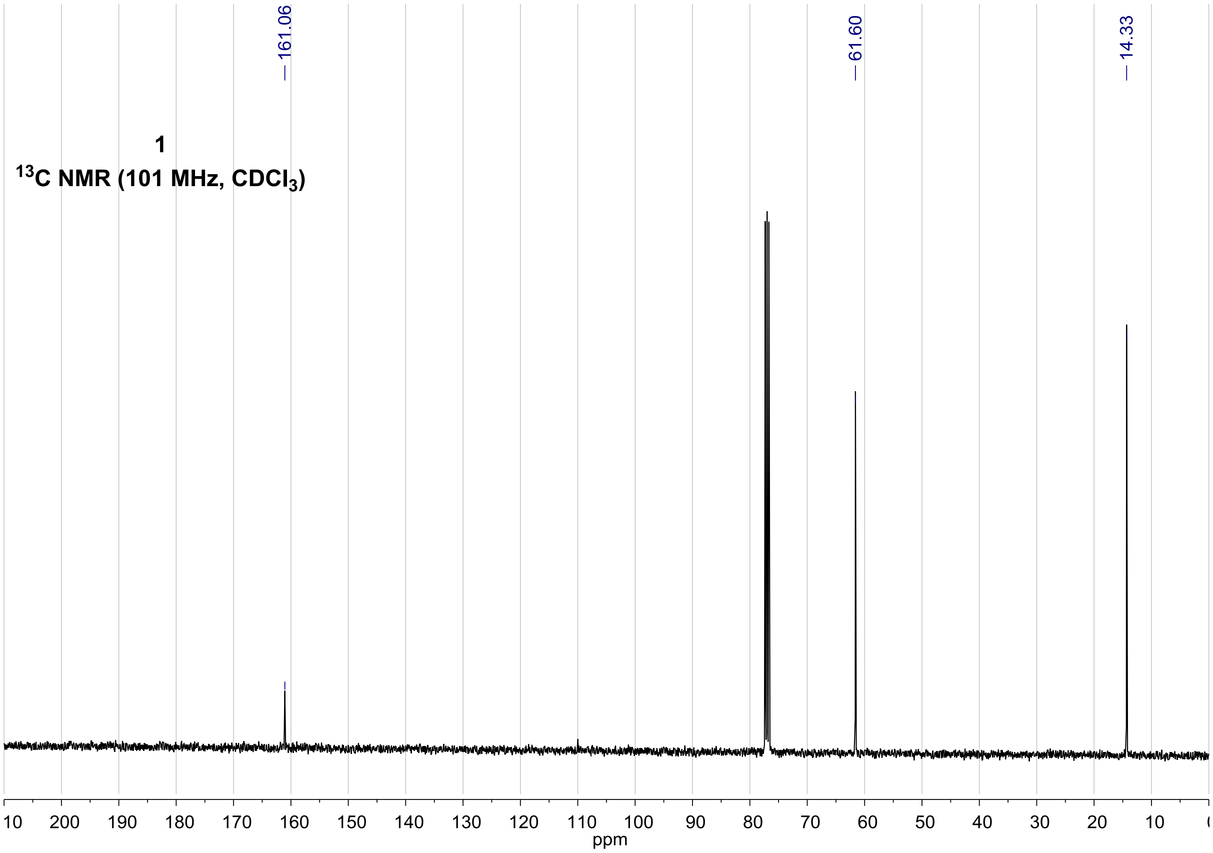

161.06
61.60
14.33
1
13C NMR (101 MHz, CDCl3)
ppm

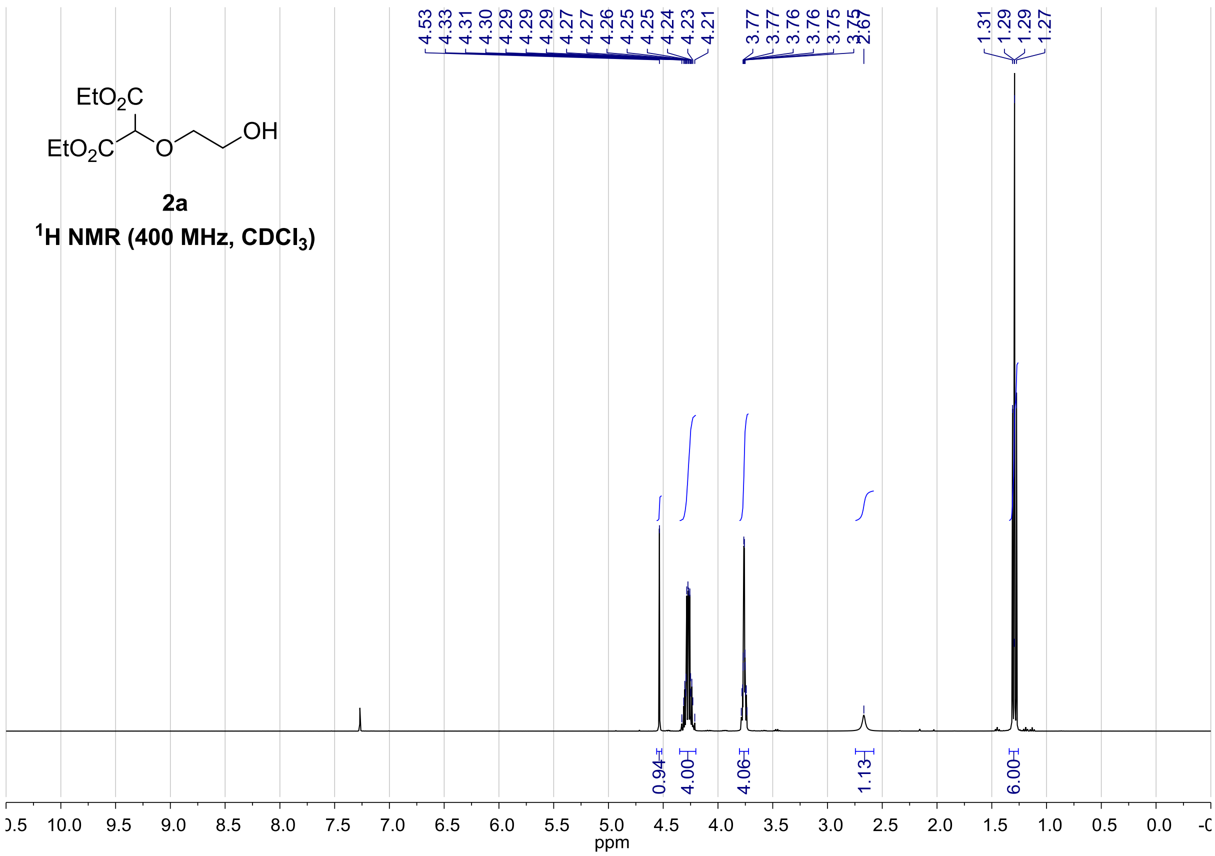
EtO2C
EtO2C
O
OH
2a
1H NMR (400 MHz, CDCl3)
4.53 4.33 4.31 4.30 4.29 4.29 4.29 4.27 4.27 4.26 4.25 4.25 4.24 4.23 4.21
3.77 3.77 3.76 3.76 3.75 3.75 2.67
1.31 1.29 1.29 1.27
0.94 4.00 4.06 1.13 6.00
ppm

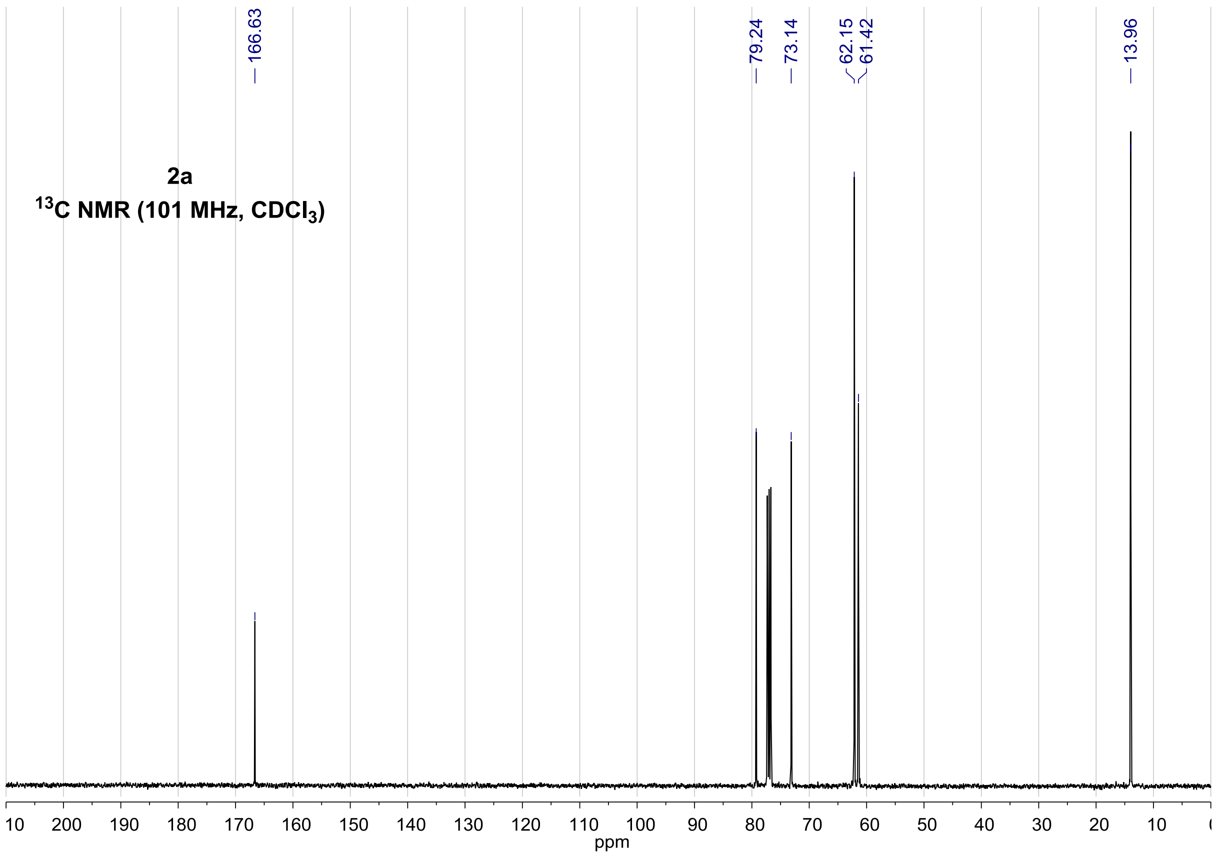
2a
13C NMR (101 MHz, CDCl3)
166.63
79.24
73.14
62.15
61.42
13.96
ppm

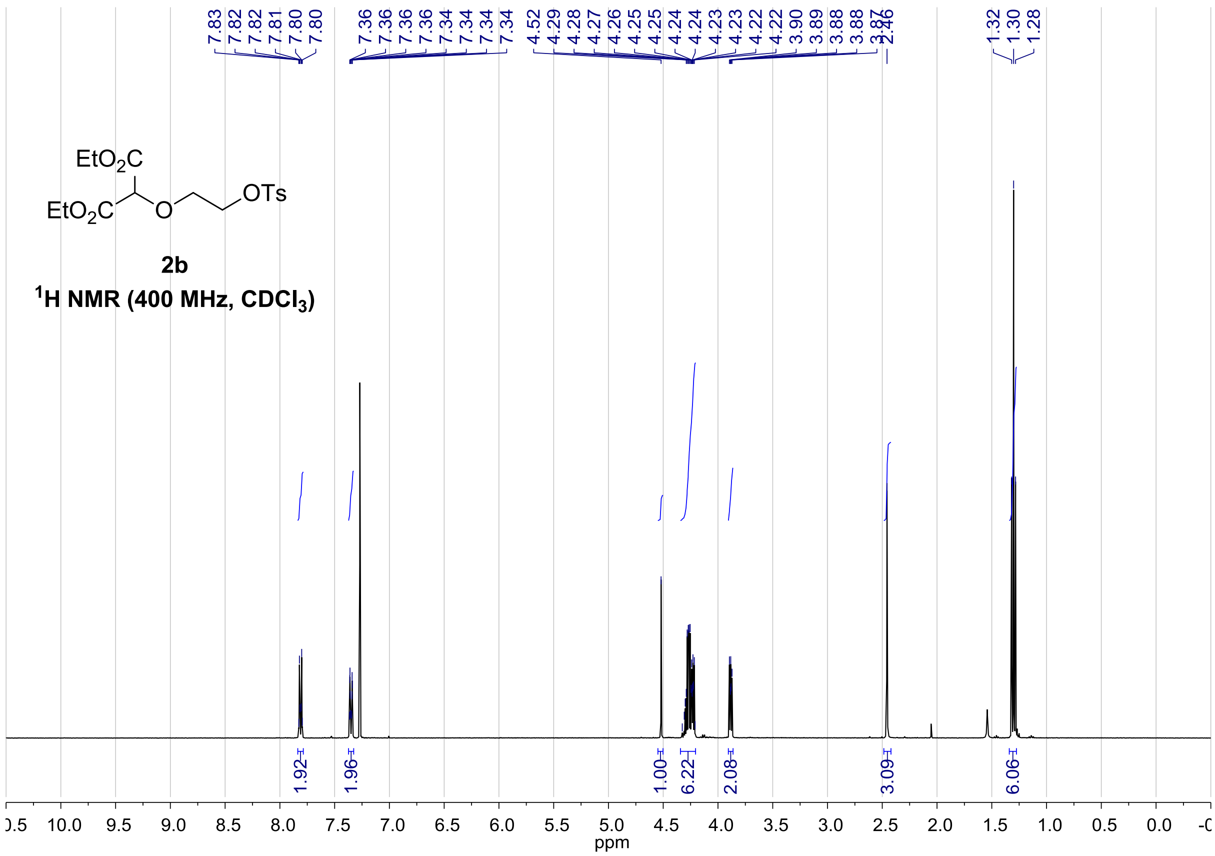
7.83
7.82
7.82
7.81
7.80
7.80
7.36
7.36
7.36
7.36
7.34
7.34
7.34
7.34
4.52
4.29
4.28
4.27
4.26
4.25
4.25
4.24
4.24
4.23
4.23
4.22
4.22
3.90
3.89
3.89
3.88
3.88
3.87
2.46
1.32
1.30
1.28
EtO2C
EtO2C
O
OTs
2b
1H NMR (400 MHz, CDCl3)
1.92
1.96
1.00
6.22
2.08
3.09
6.06
ppm

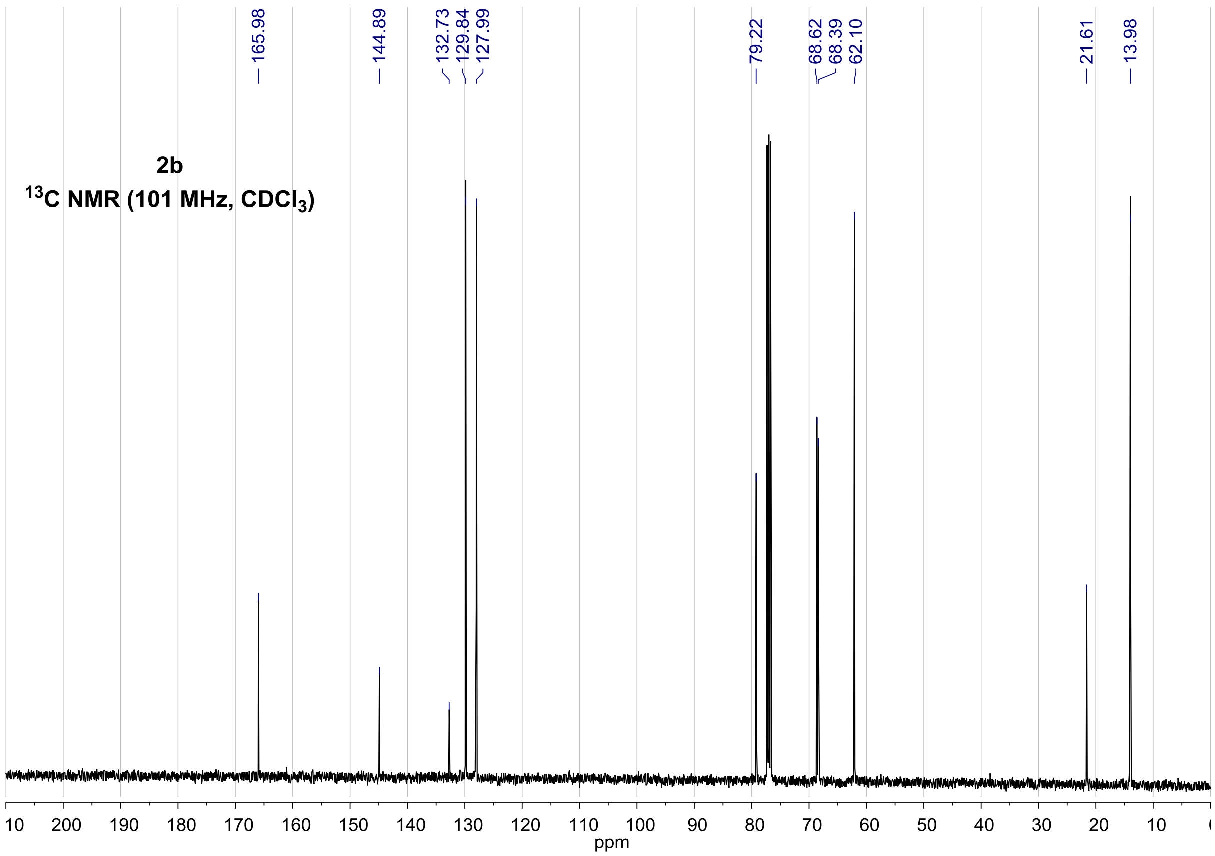
165.98
144.89
132.73
129.84
127.99
79.22
68.62
68.39
62.10
21.61
13.98
2b
13C NMR (101 MHz, CDCl3)
ppm

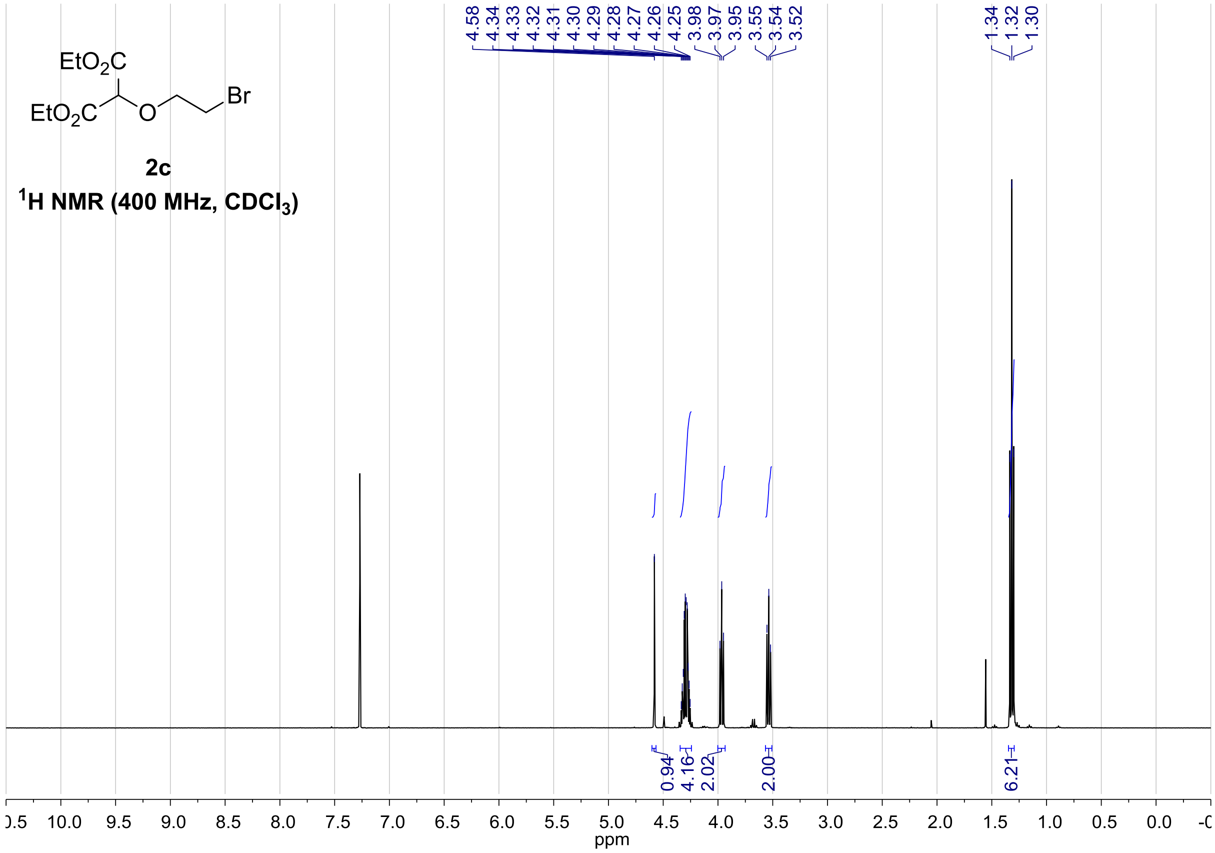
EtO2C
EtO2C
O
Br
2c
1H NMR (400 MHz, CDCl3)
4.58 4.34 4.33 4.32 4.31 4.30 4.29 4.28 4.27 4.26 4.25 3.98 3.97 3.95 3.55 3.54 3.52
1.34 1.32 1.30
0.94 4.16 2.02 2.00 6.21
ppm

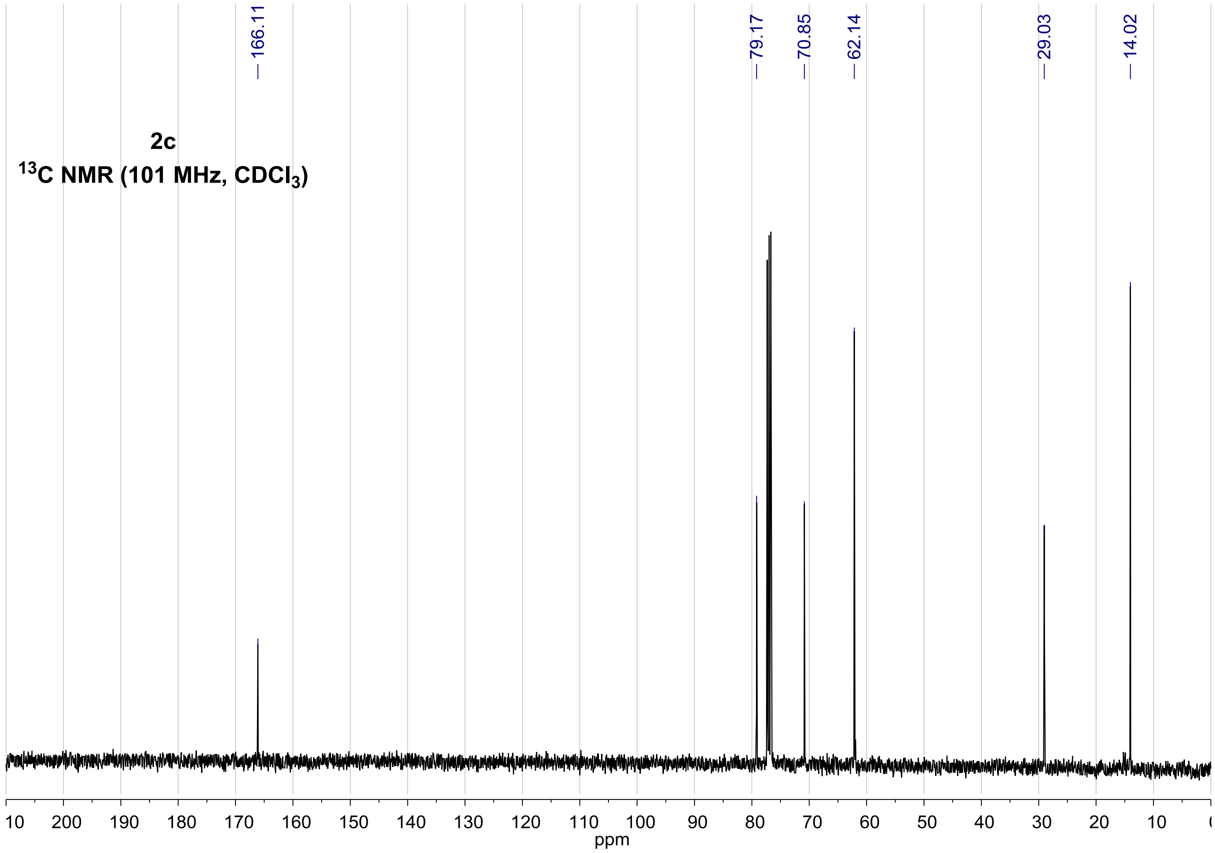
166.11
79.17
70.85
62.14
29.03
14.02
2c
13C NMR (101 MHz, CDCl3)
ppm

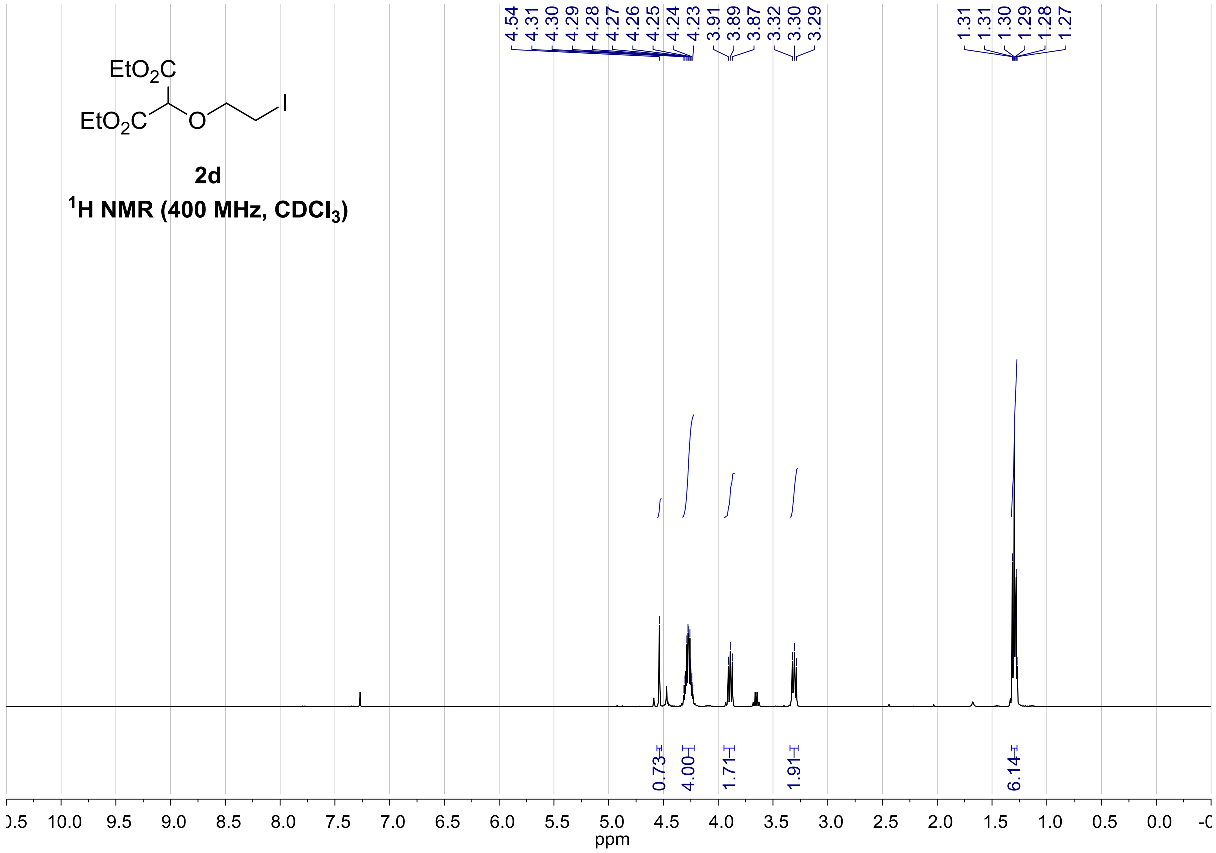

2d

^{1}H NMR (400 MHz, $CDCl_3$)

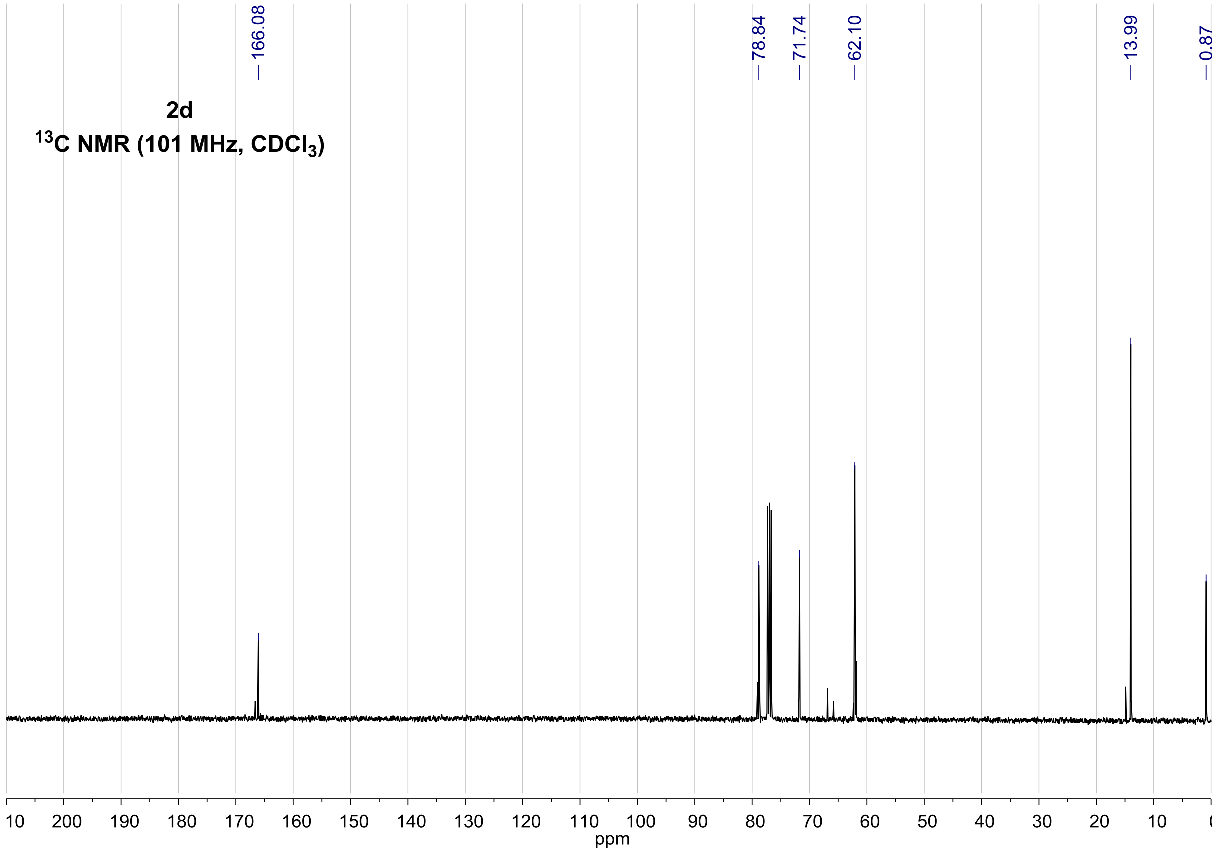

2d

^{13}C NMR (101 MHz, $CDCl_3$)

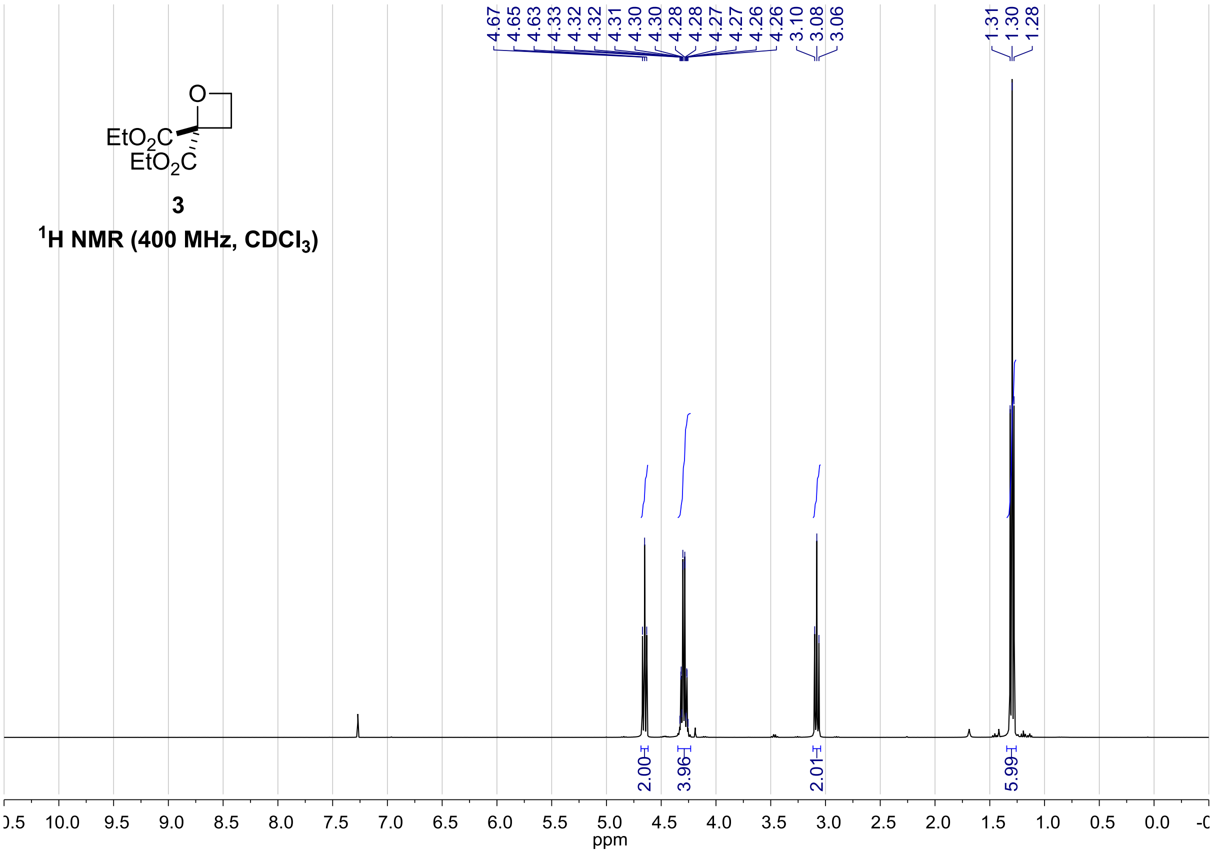
EtO2C
EtO2C
O
3
1H NMR (400 MHz, CDCl3)
4.67
4.65
4.63
4.33
4.32
4.32
4.31
4.30
4.30
4.28
4.28
4.27
4.27
4.26
4.26
3.10
3.08
3.06
1.31
1.30
1.28
2.00
3.96
2.01
5.99
ppm

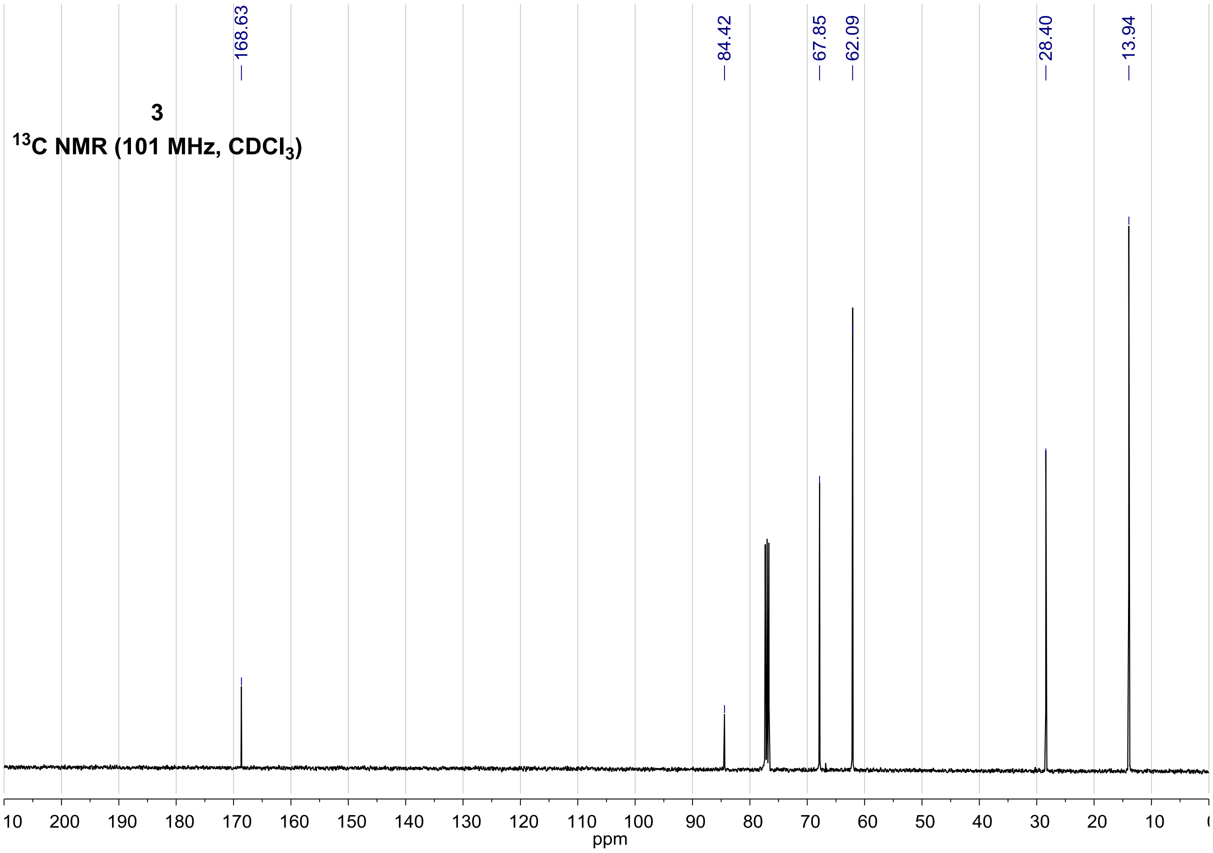
3
13C NMR (101 MHz, CDCl3)
168.63
84.42
67.85
62.09
28.40
13.94
ppm

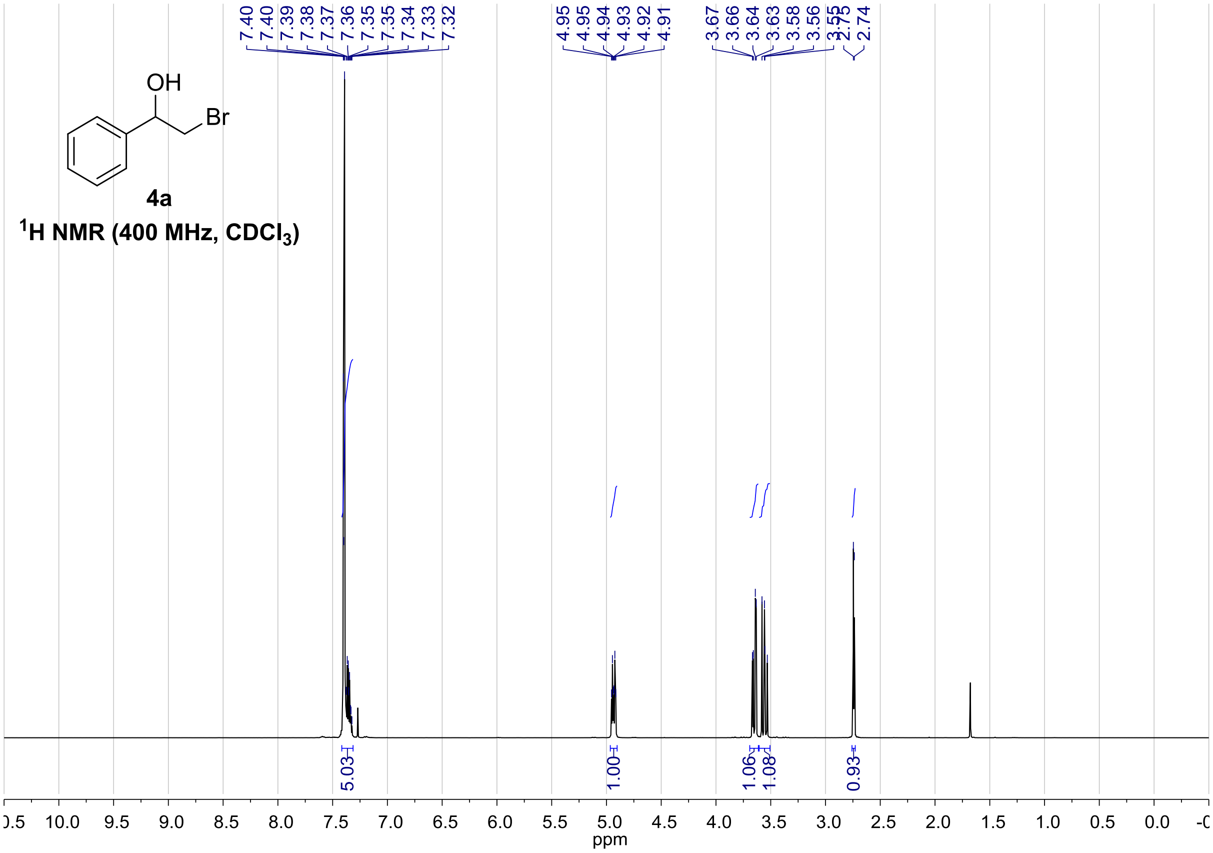
OH
Br
4a
1H NMR (400 MHz, CDCl3)
7.40 7.40 7.39 7.38 7.37 7.36 7.35 7.35 7.34 7.33 7.32
4.95 4.95 4.94 4.93 4.92 4.91
3.67 3.66 3.64 3.63 3.58 3.56 3.55 2.75 2.74
5.03 1.00 1.06 1.08 0.93
ppm

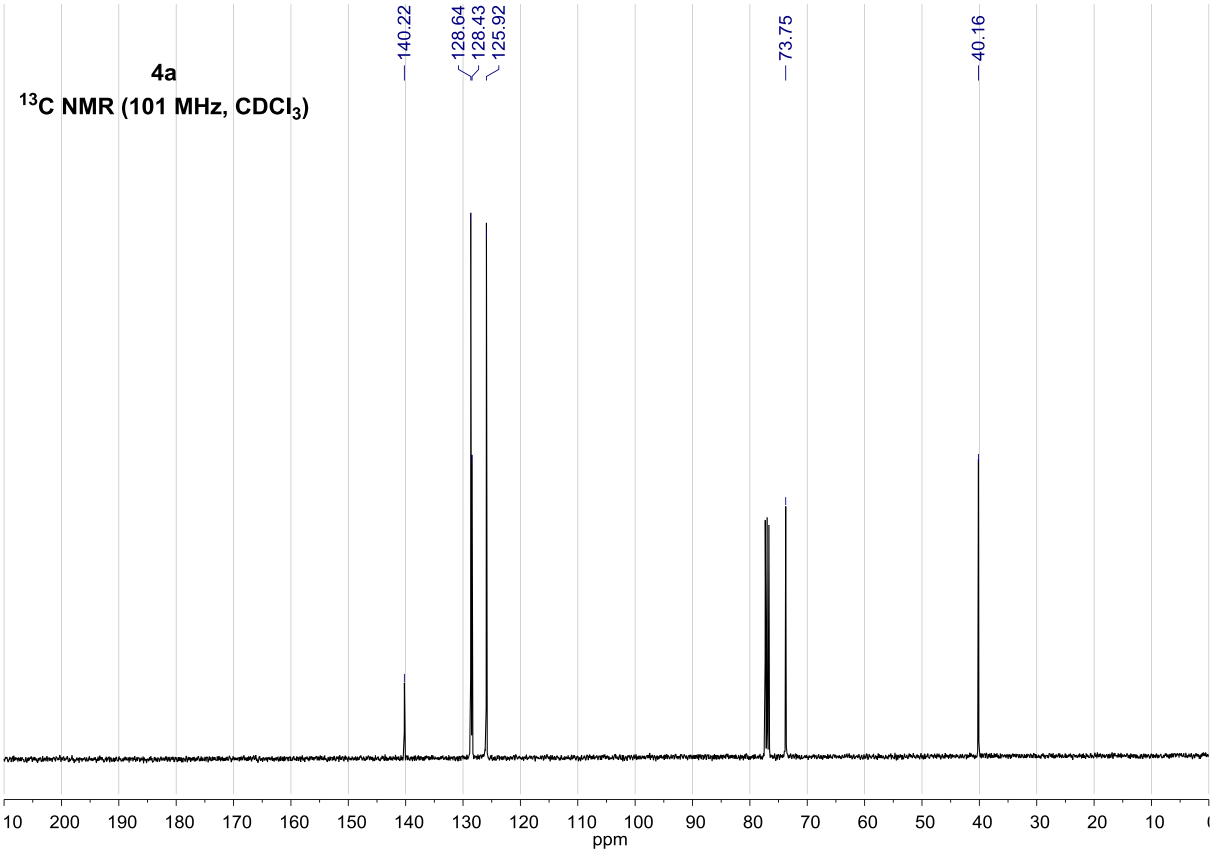
4a
13C NMR (101 MHz, CDCl3)
140.22
128.64 128.43 125.92
73.75
40.16
ppm

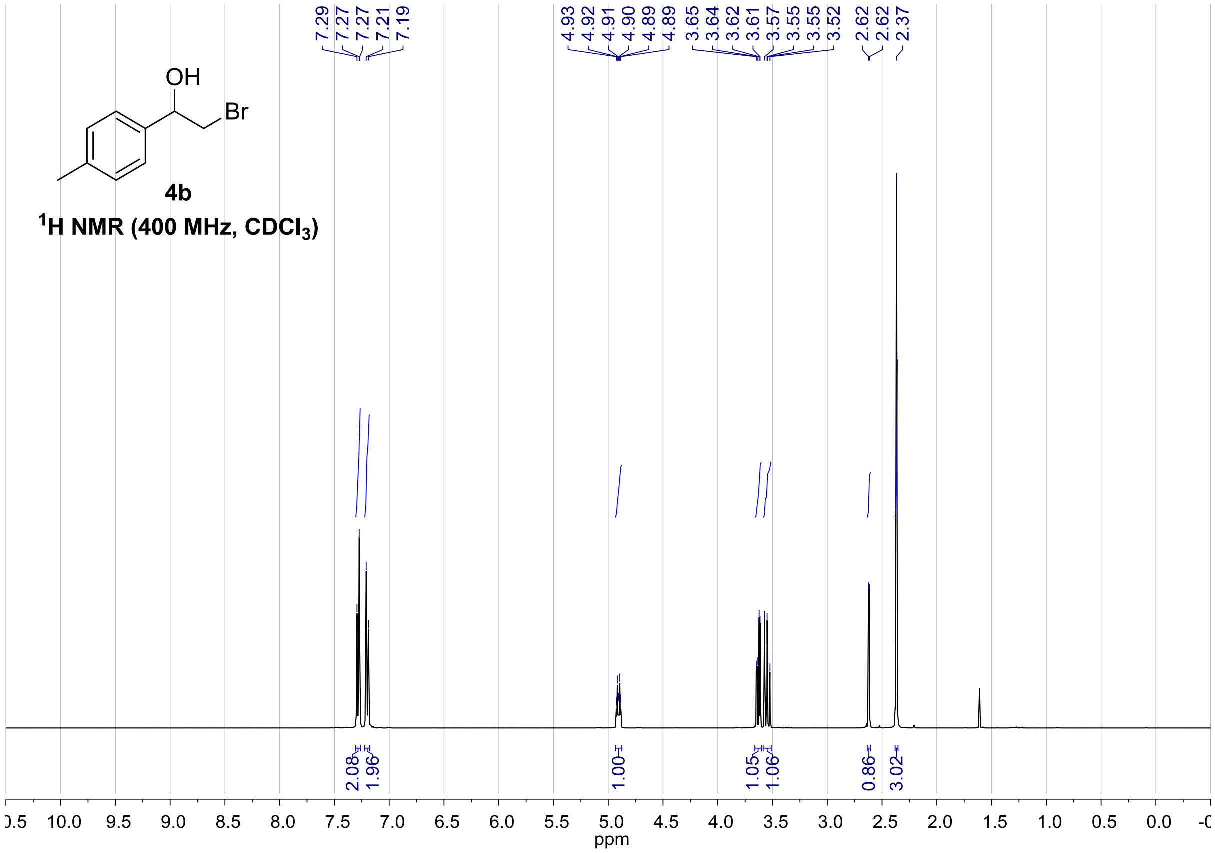
OH
Br
4b
1H NMR (400 MHz, CDCl3)
7.29
7.27
7.27
7.21
7.19
4.93
4.92
4.91
4.90
4.89
4.89
3.65
3.64
3.62
3.61
3.57
3.55
3.55
3.52
2.62
2.62
2.37
2.08
1.96
1.00
1.05
1.06
0.86
3.02
10.0 9.5 9.0 8.5 8.0 7.5 7.0 6.5 6.0 5.5 5.0 4.5 4.0 3.5 3.0 2.5 2.0 1.5 1.0 0.5 0.0
ppm

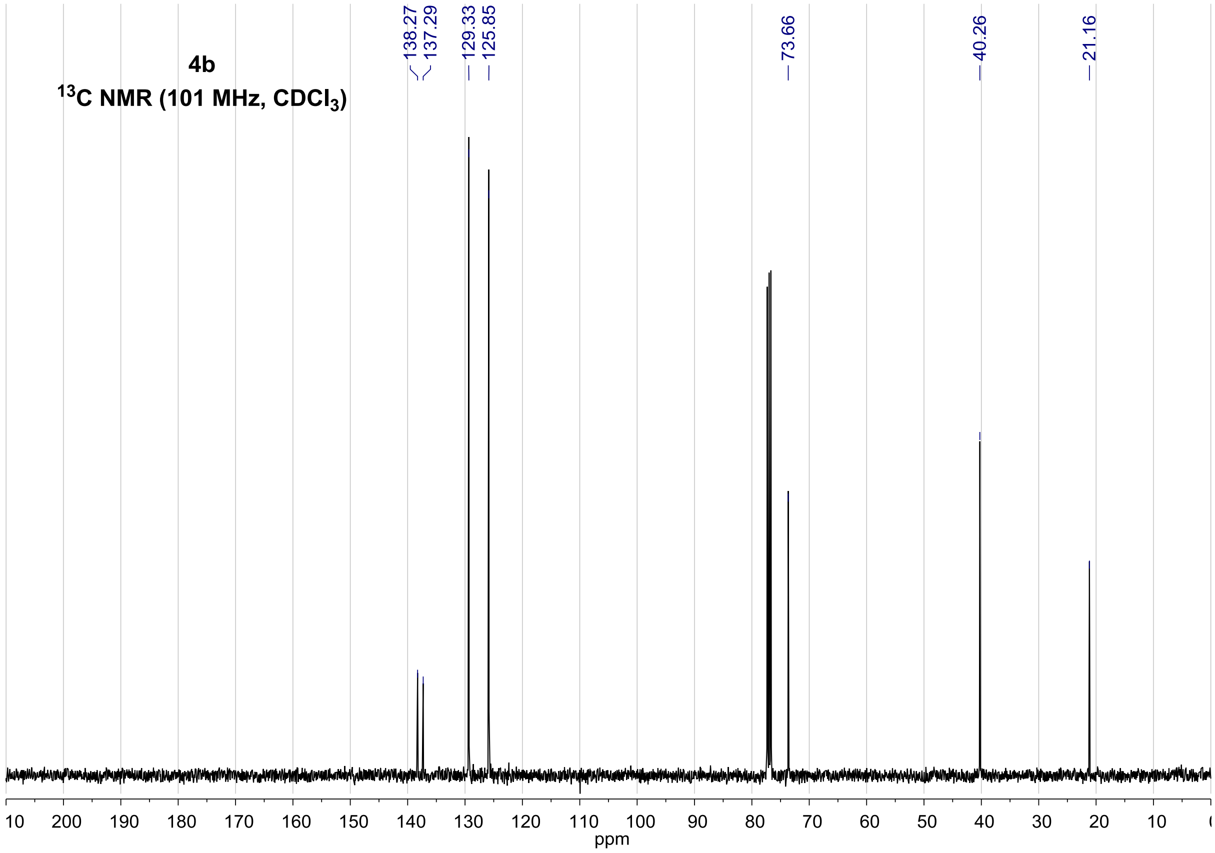
4b
13C NMR (101 MHz, CDCl3)
138.27
137.29
129.33
125.85
73.66
40.26
21.16
200 190 180 170 160 150 140 130 120 110 100 90 80 70 60 50 40 30 20 10
ppm

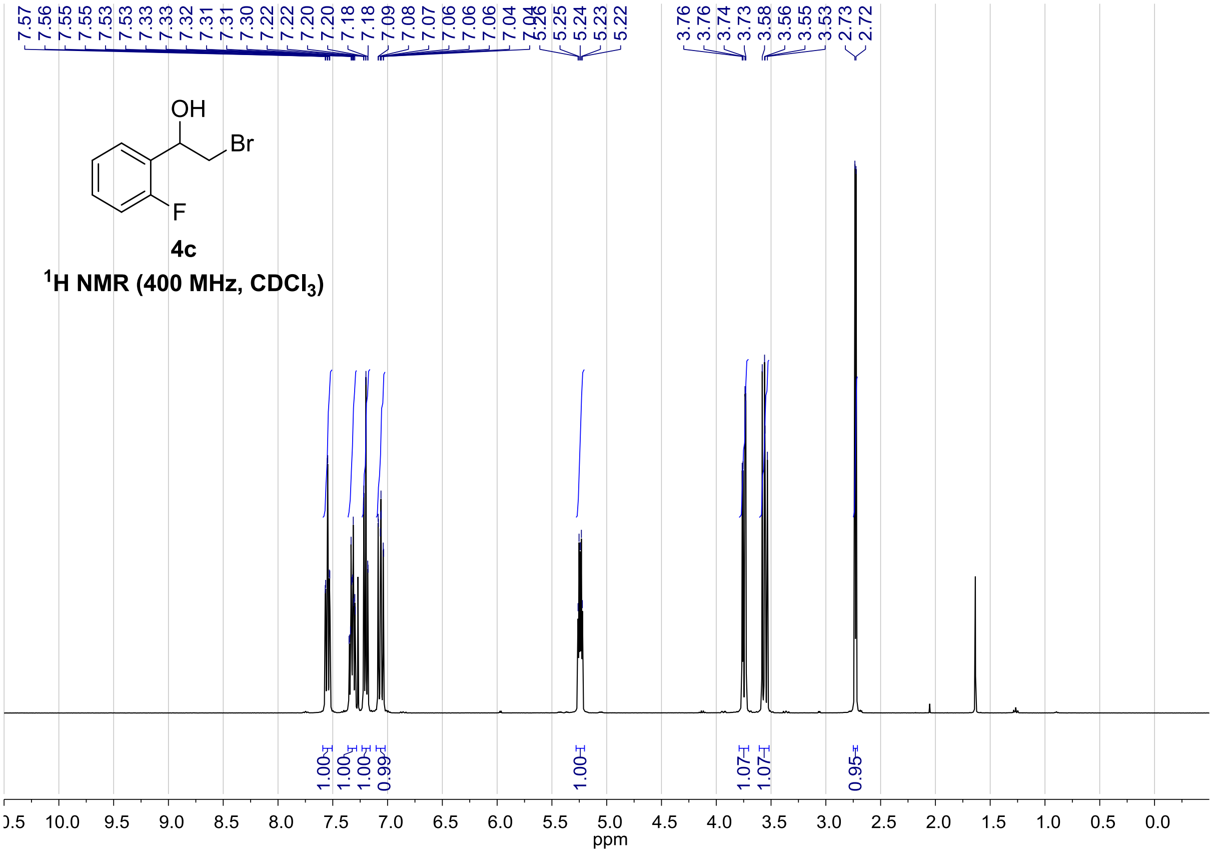

4c

^{1}H NMR (400 MHz, $CDCl_3$)

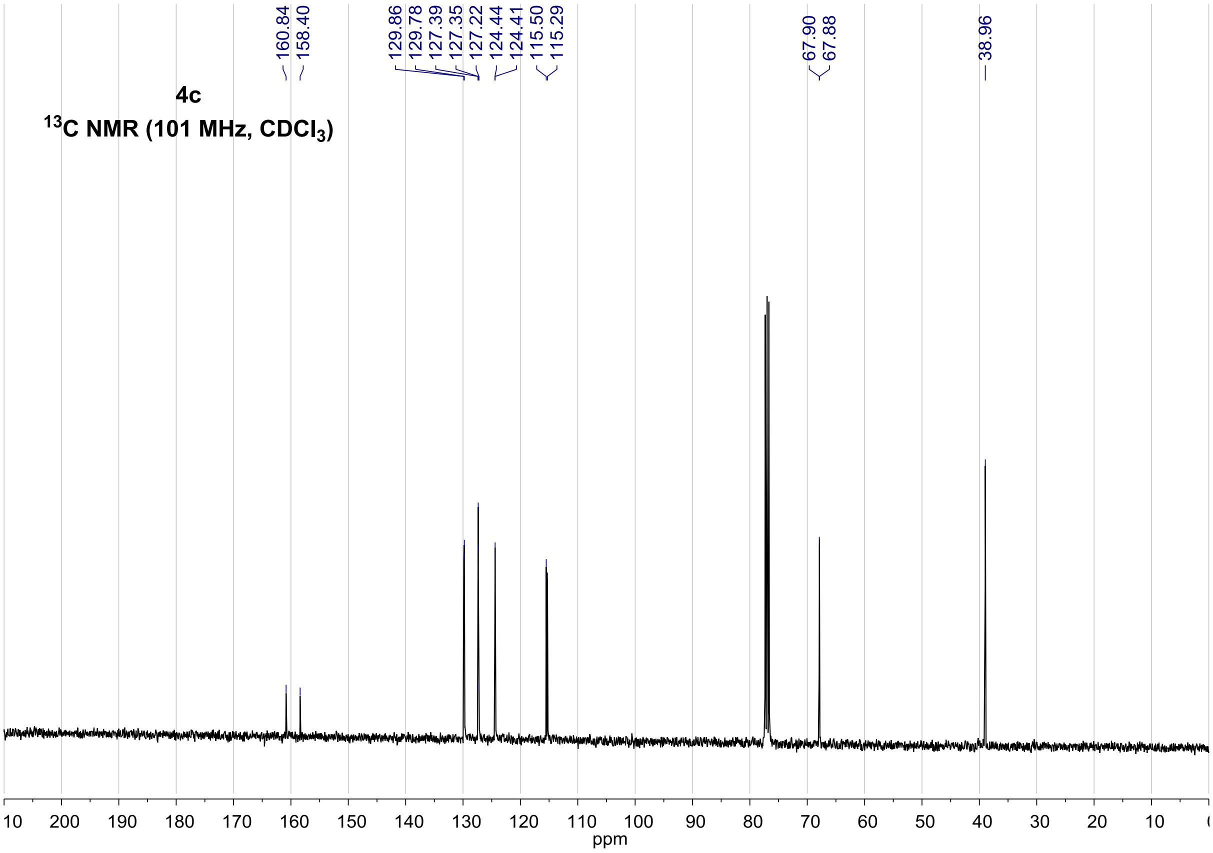

4c

^{13}C NMR (101 MHz, $CDCl_3$)

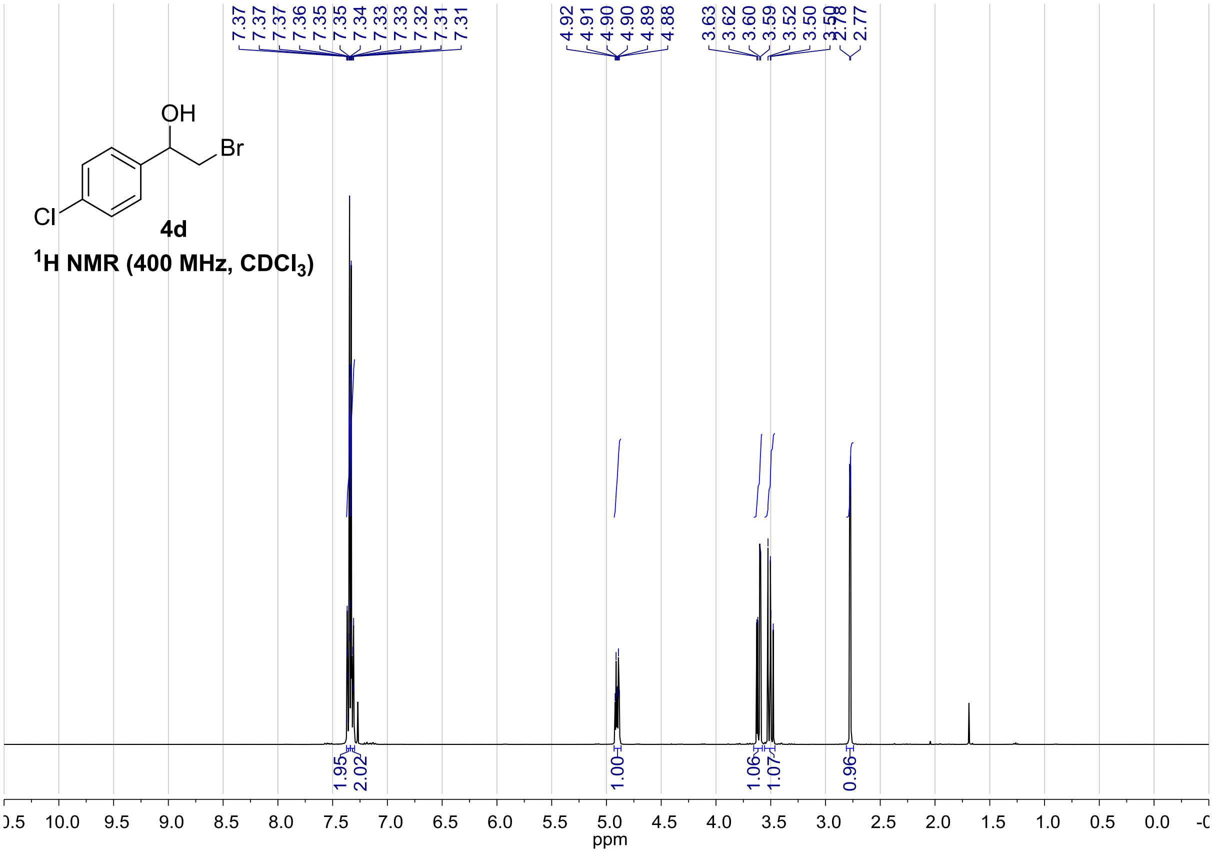
7.37
7.37
7.37
7.36
7.35
7.35
7.34
7.33
7.33
7.32
7.31
7.31
4.92
4.91
4.90
4.90
4.89
4.88
3.63
3.62
3.60
3.59
3.52
3.50
2.78
2.77
OH
Br
Cl
4d
^{1}H NMR (400 MHz, CDCl$_3$)
1.95
2.02
1.00
1.06
1.07
0.96
10.0
9.5
9.0
8.5
8.0
7.5
7.0
6.5
6.0
5.5
5.0
4.5
4.0
3.5
3.0
2.5
2.0
1.5
1.0
0.5
0.0
ppm

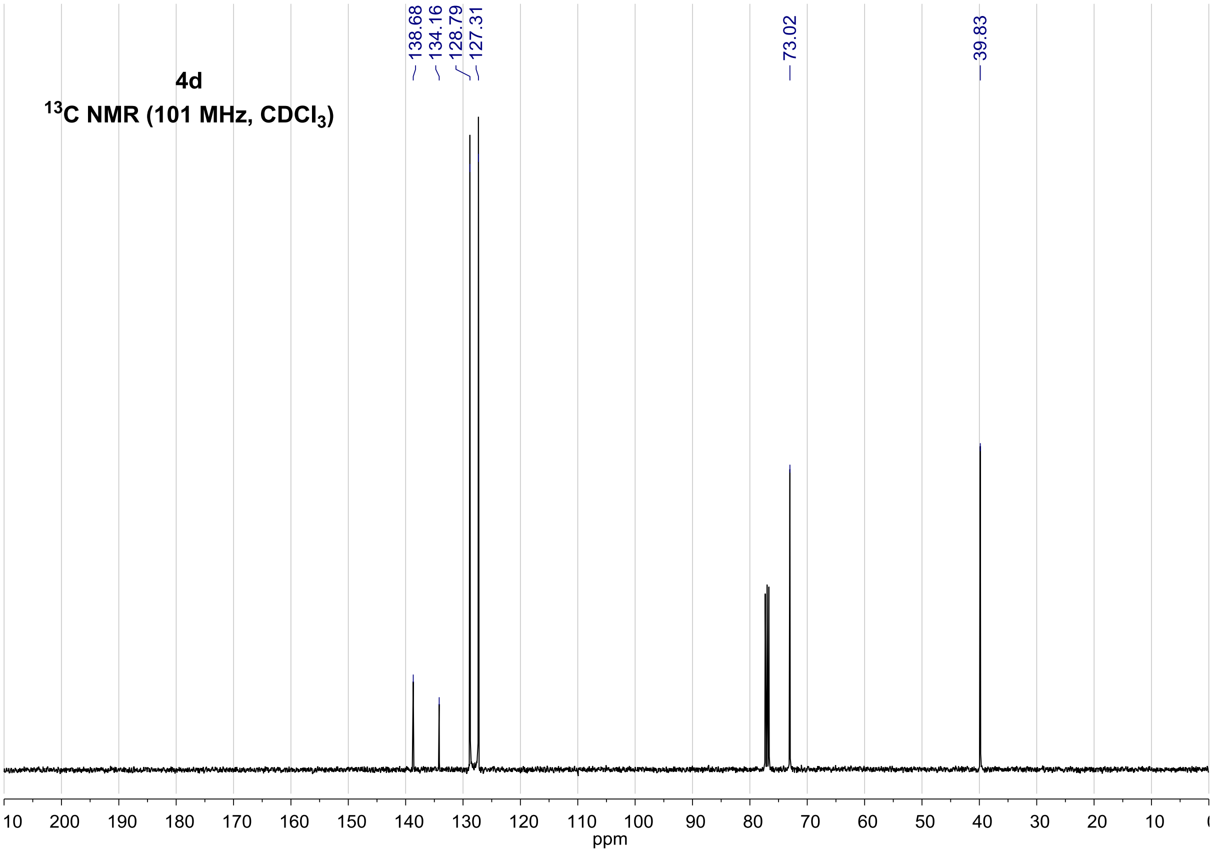
138.68
134.16
128.79
127.31
73.02
39.83
4d
^{13}C NMR (101 MHz, CDCl$_3$)
200
190
180
170
160
150
140
130
120
110
100
90
80
70
60
50
40
30
20
10
ppm

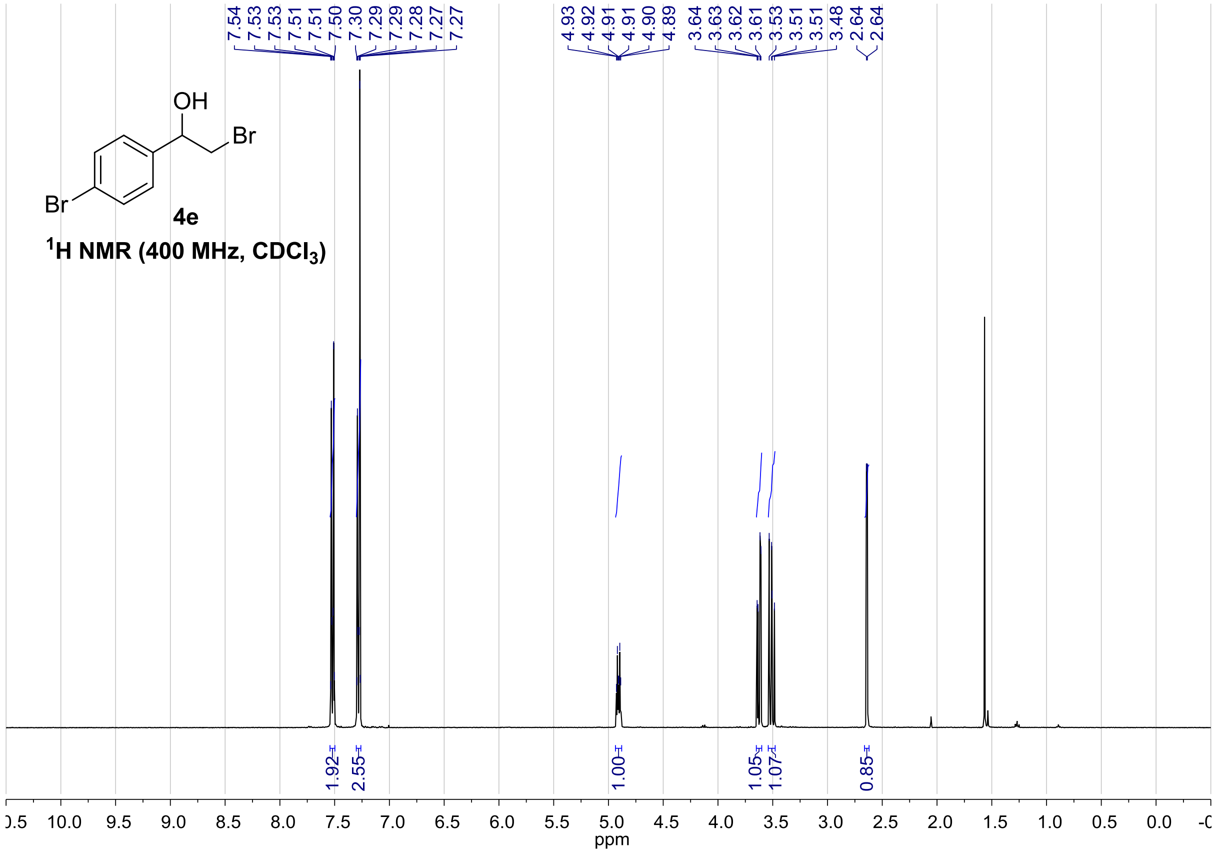

OH
Br
Br
4e
^{1}H NMR (400 MHz, $CDCl_3$)
7.54 7.53 7.53 7.51 7.51 7.50 7.30 7.29 7.29 7.28 7.27 7.27
4.93 4.92 4.91 4.91 4.90 4.89
3.64 3.63 3.62 3.61 3.53 3.51 3.51 3.48 2.64 2.64
1.92 2.55 1.00 1.05 1.07 0.85
ppm

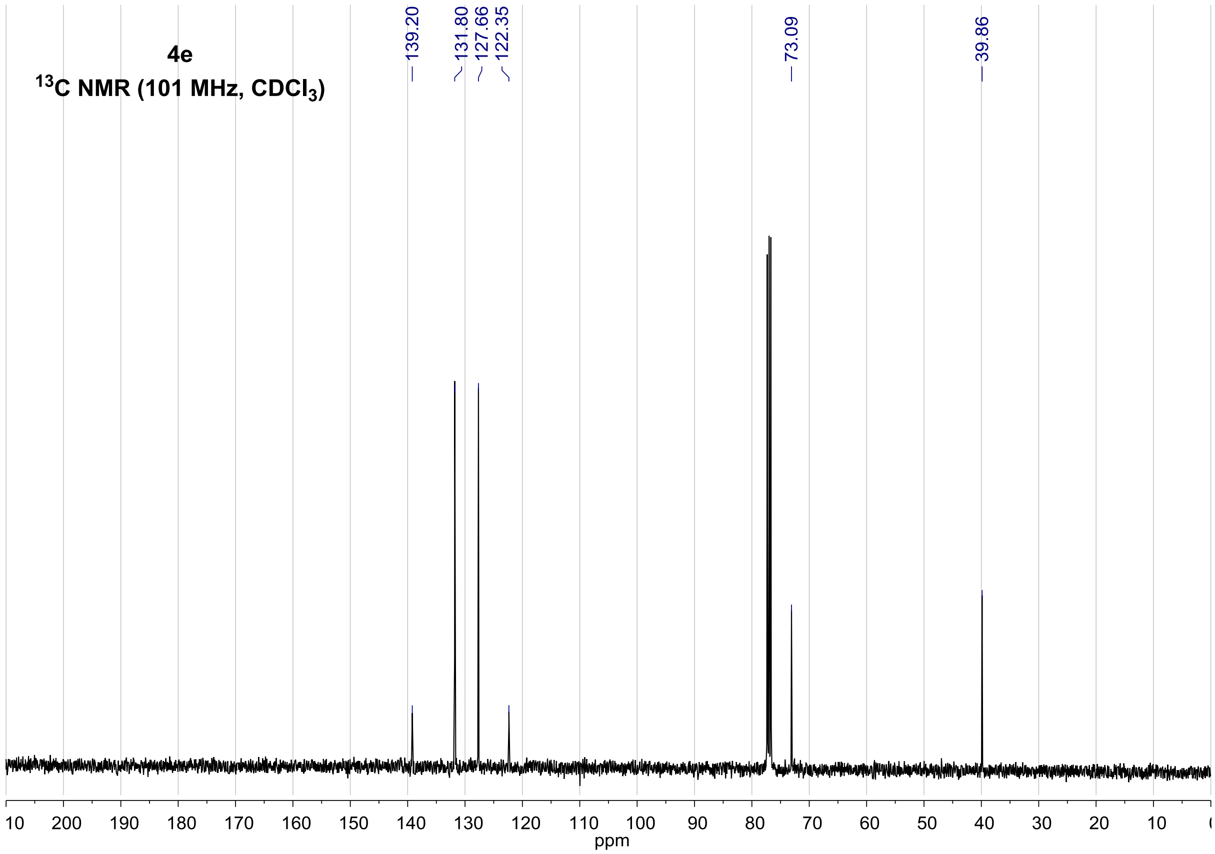

4e
^{13}C NMR (101 MHz, $CDCl_3$)
139.20
131.80
127.66
122.35
73.09
39.86
ppm

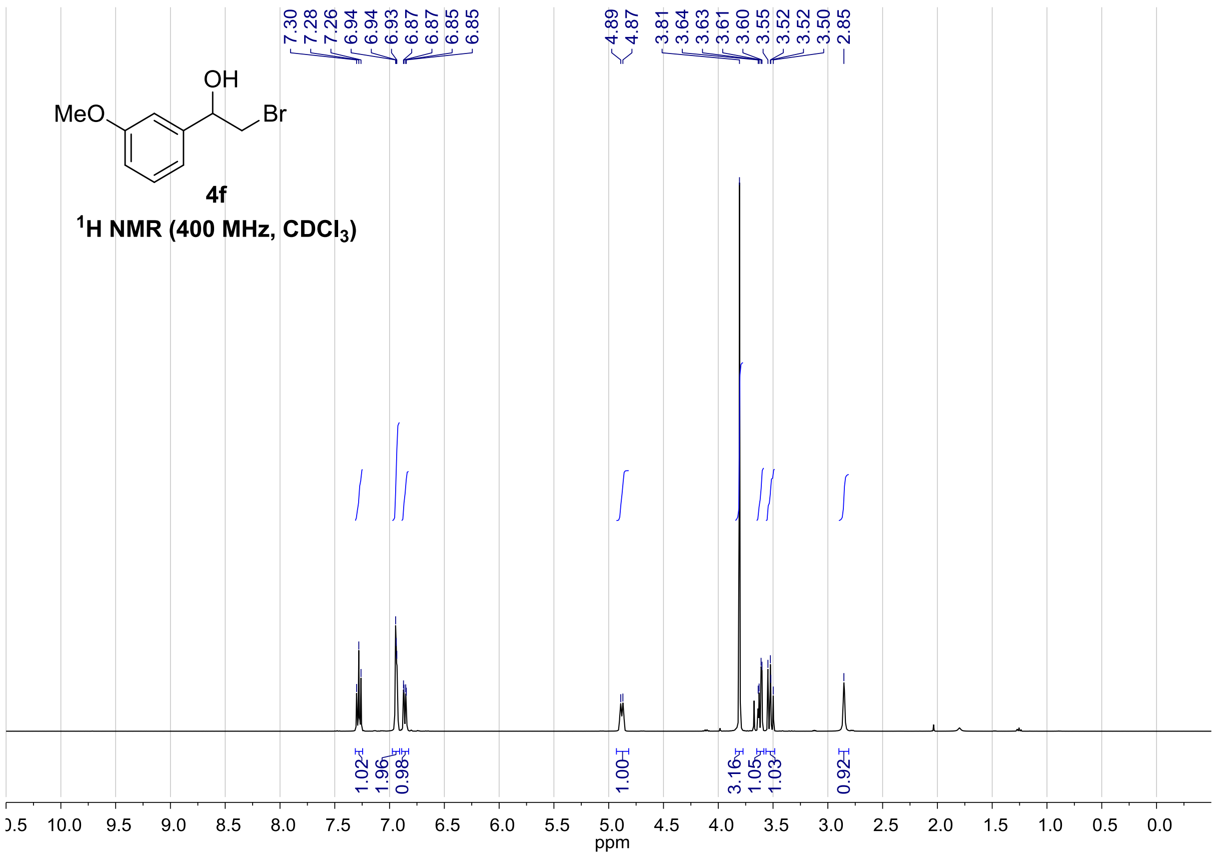
7.30
7.28
7.26
6.94
6.94
6.93
6.87
6.87
6.85
6.85
4.89
4.87
3.81
3.64
3.63
3.61
3.60
3.55
3.52
3.52
3.50
2.85
OH
MeO
Br
4f
1H NMR (400 MHz, CDCl3)
1.02
1.96
0.98
1.00
3.16
1.05
1.03
0.92
ppm

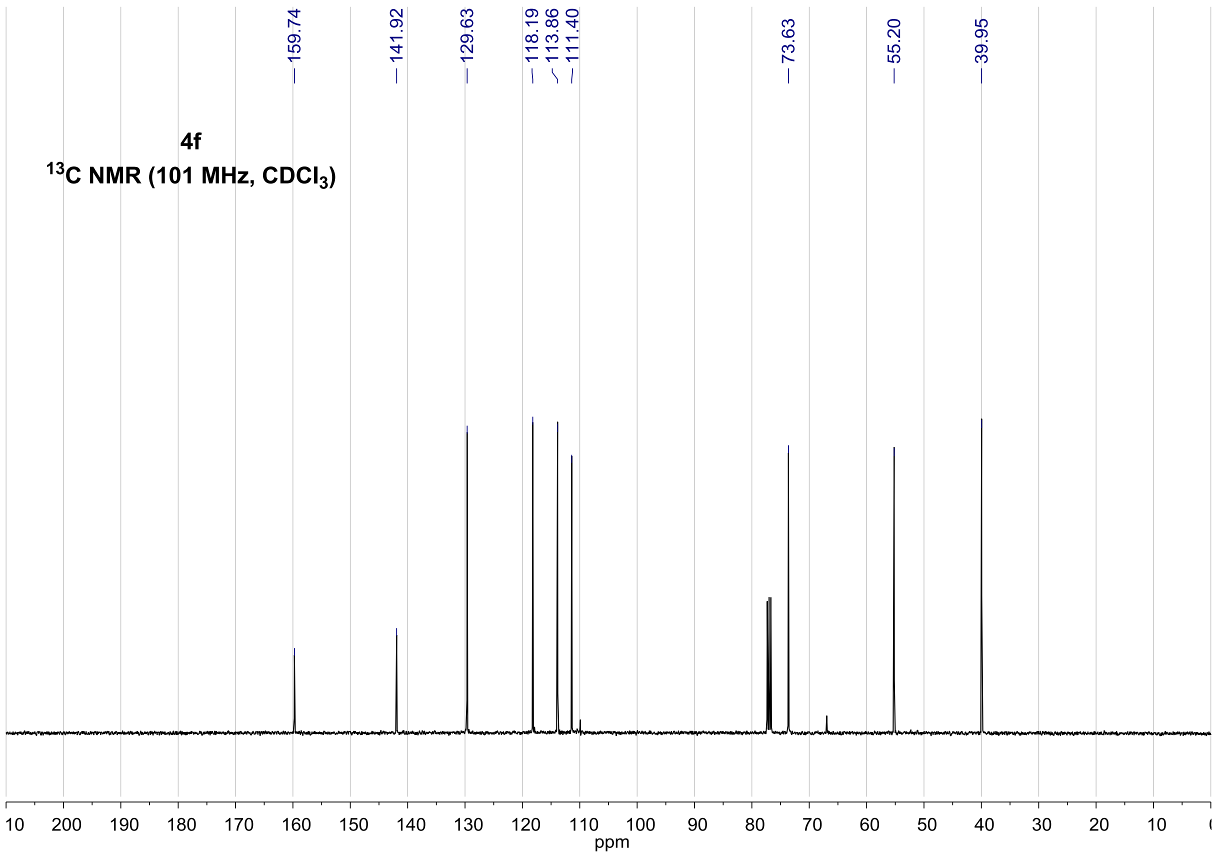
159.74
141.92
129.63
118.19
113.86
111.40
73.63
55.20
39.95
4f
13C NMR (101 MHz, CDCl3)
ppm

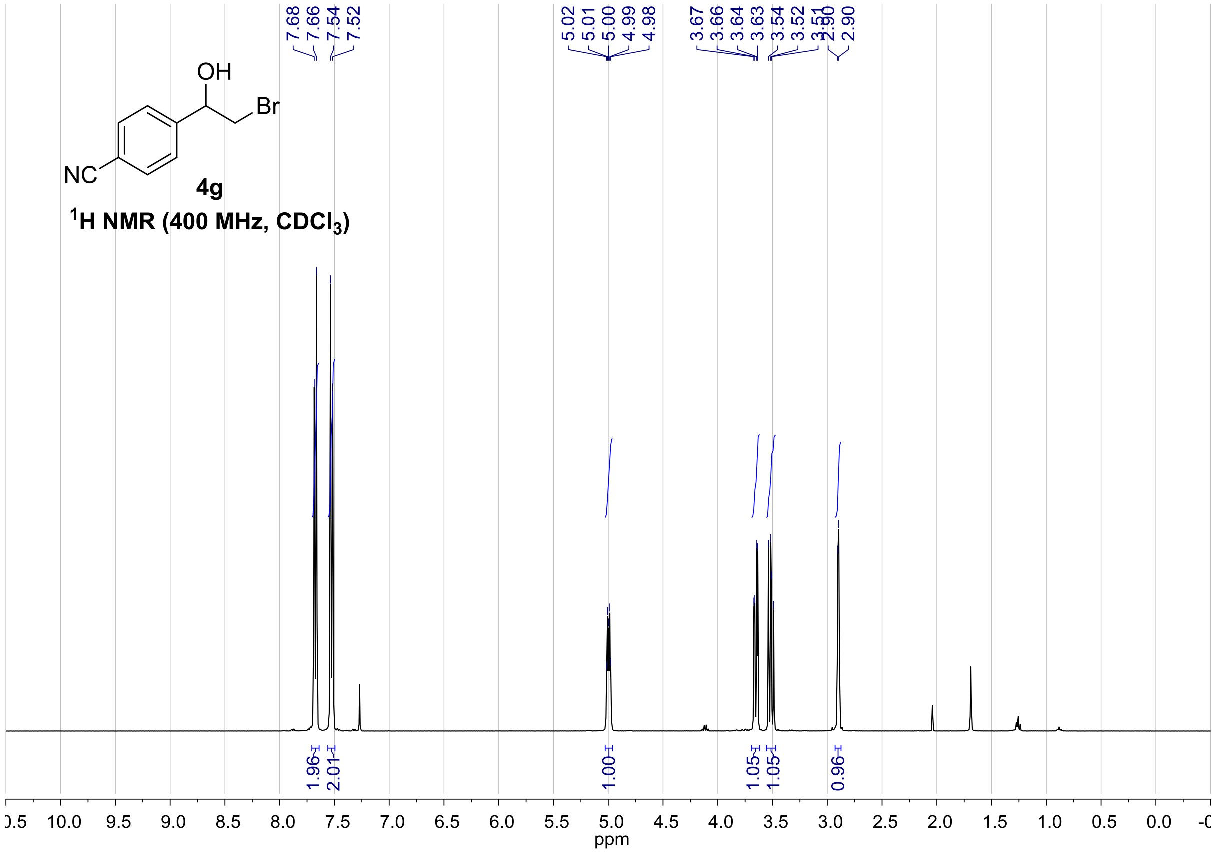
7.68
7.66
7.54
7.52
5.02
5.01
5.00
4.99
4.98
3.67
3.66
3.64
3.63
3.54
3.52
3.51
2.90
2.90
OH
Br
NC
4g
1H NMR (400 MHz, CDCl3)
1.96
2.01
1.00
1.05
1.05
0.96
ppm

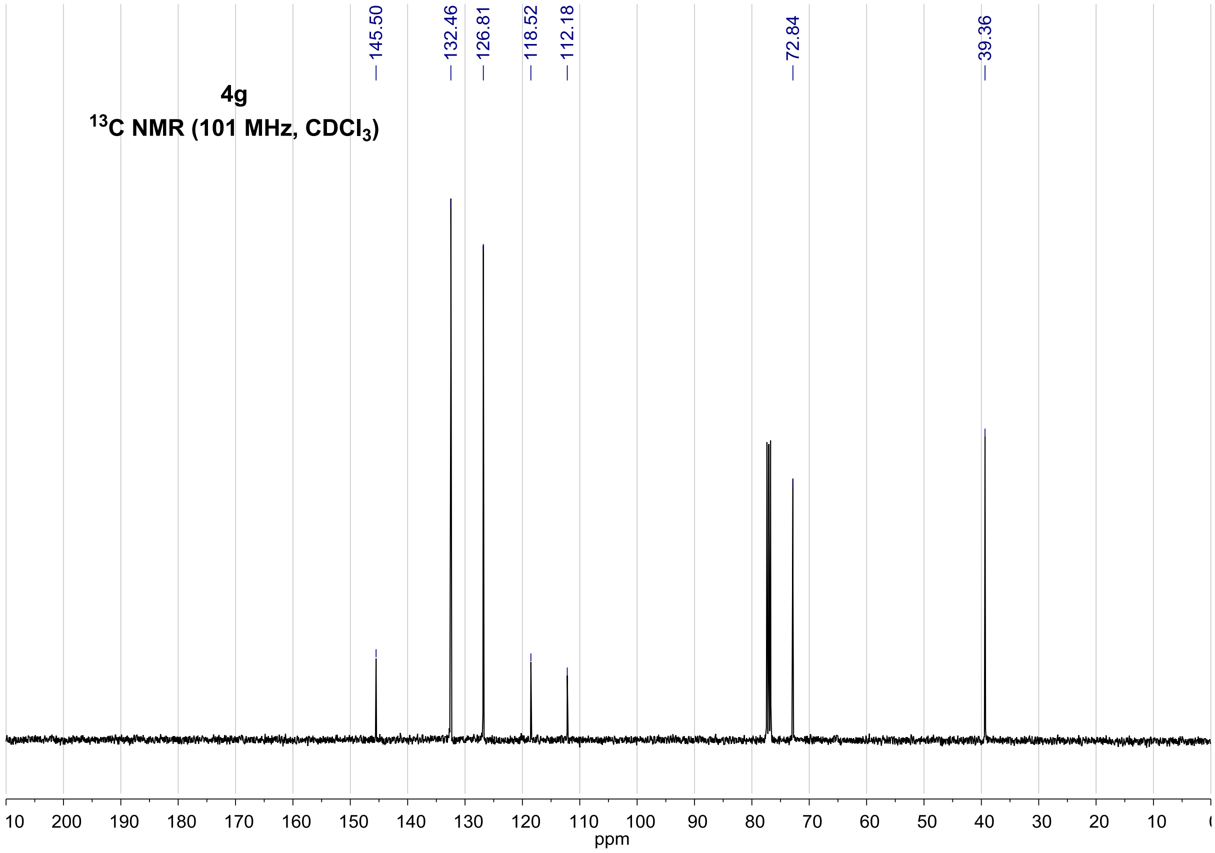
145.50
132.46
126.81
118.52
112.18
72.84
39.36
4g
13C NMR (101 MHz, CDCl3)
ppm

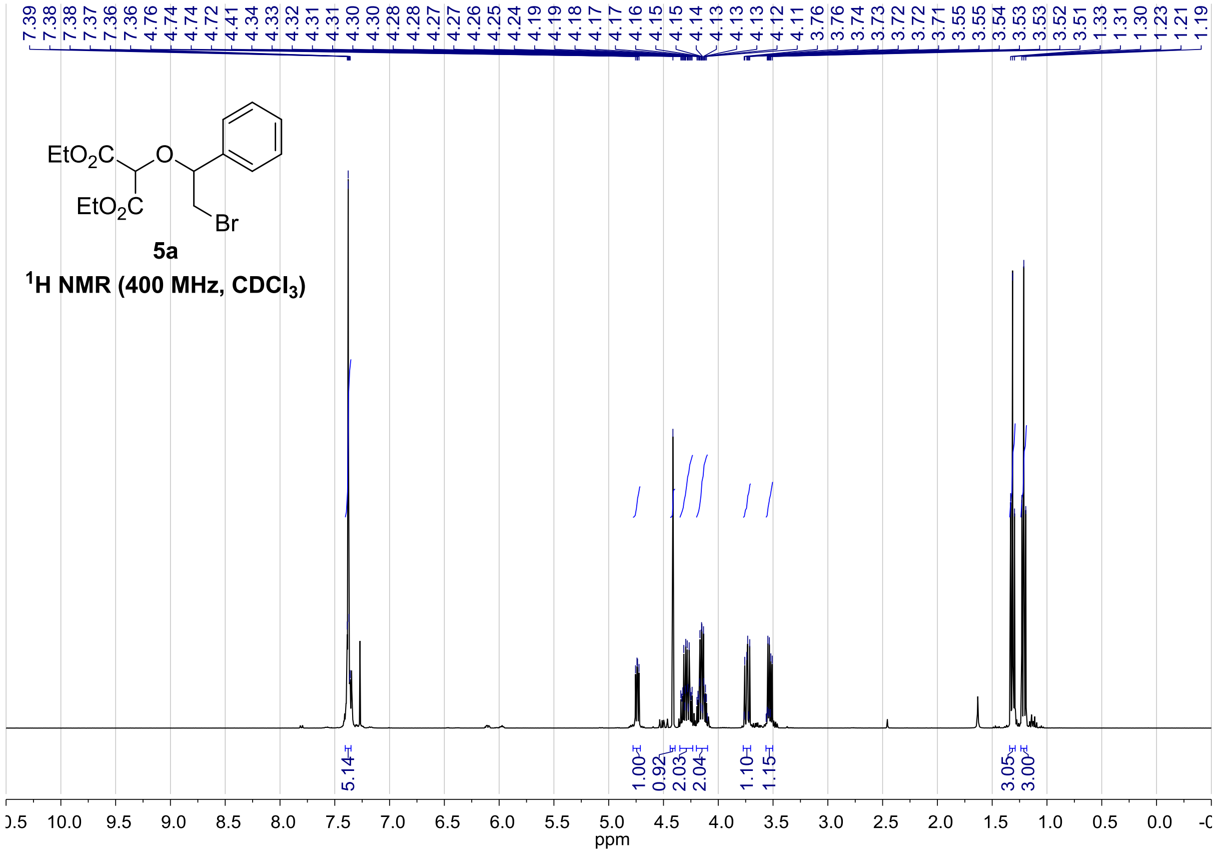
7.39 7.38 7.38 7.37 7.36 7.36 4.76 4.74 4.74 4.72 4.41 4.34 4.33 4.32 4.31 4.31 4.30 4.30 4.28 4.28 4.27 4.27 4.26 4.25 4.24 4.19 4.19 4.18 4.17 4.17 4.16 4.15 4.15 4.14 4.13 4.13 4.13 4.12 4.11 3.76 3.76 3.74 3.73 3.72 3.72 3.71 3.55 3.55 3.54 3.53 3.53 3.52 3.51 1.33 1.31 1.30 1.23 1.21 1.19
EtO_2C
O
EtO_2C
Br
5a
^{1}H NMR (400 MHz, $CDCl_3$)
5.14
1.00
0.92
2.03
2.04
1.10
1.15
3.05
3.00
ppm

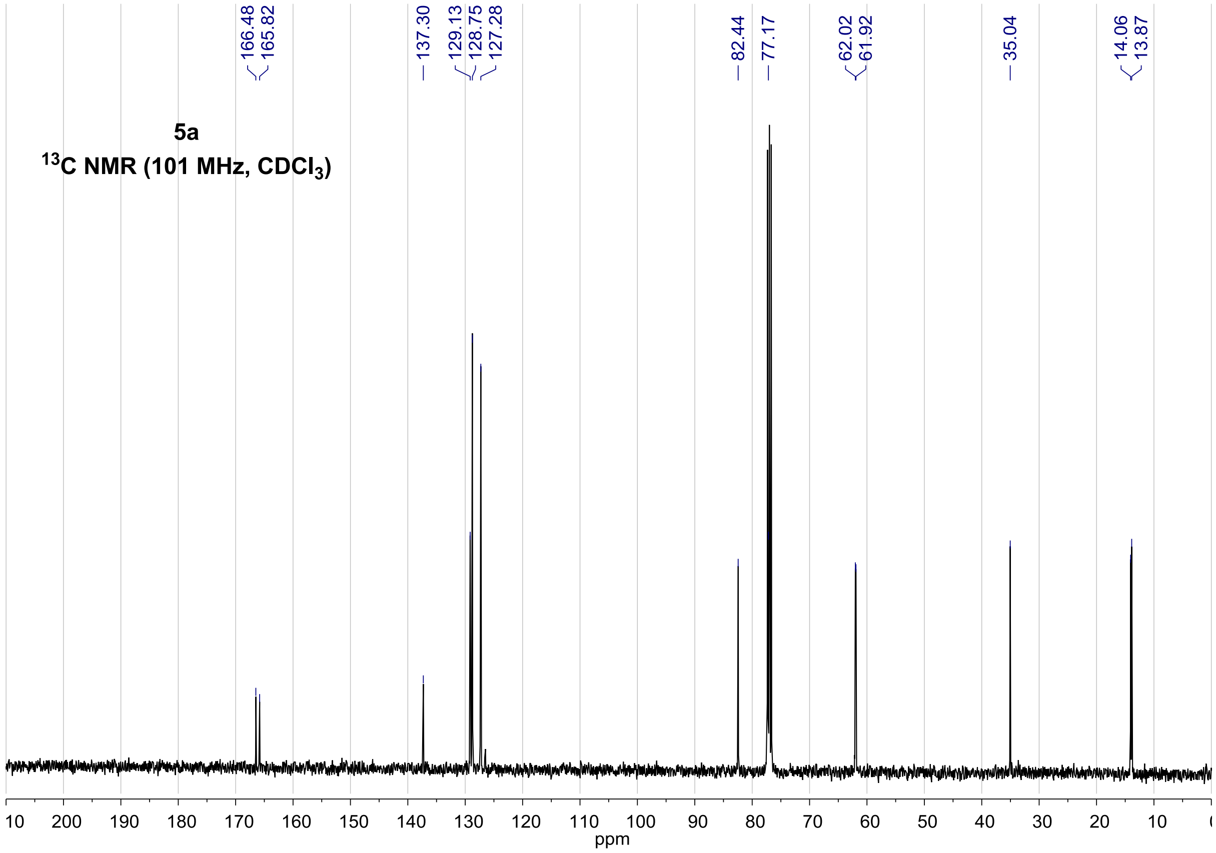
166.48 165.82 137.30 129.13 128.75 127.28 82.44 77.17 62.02 61.92 35.04 14.06 13.87
5a
^{13}C NMR (101 MHz, $CDCl_3$)
ppm

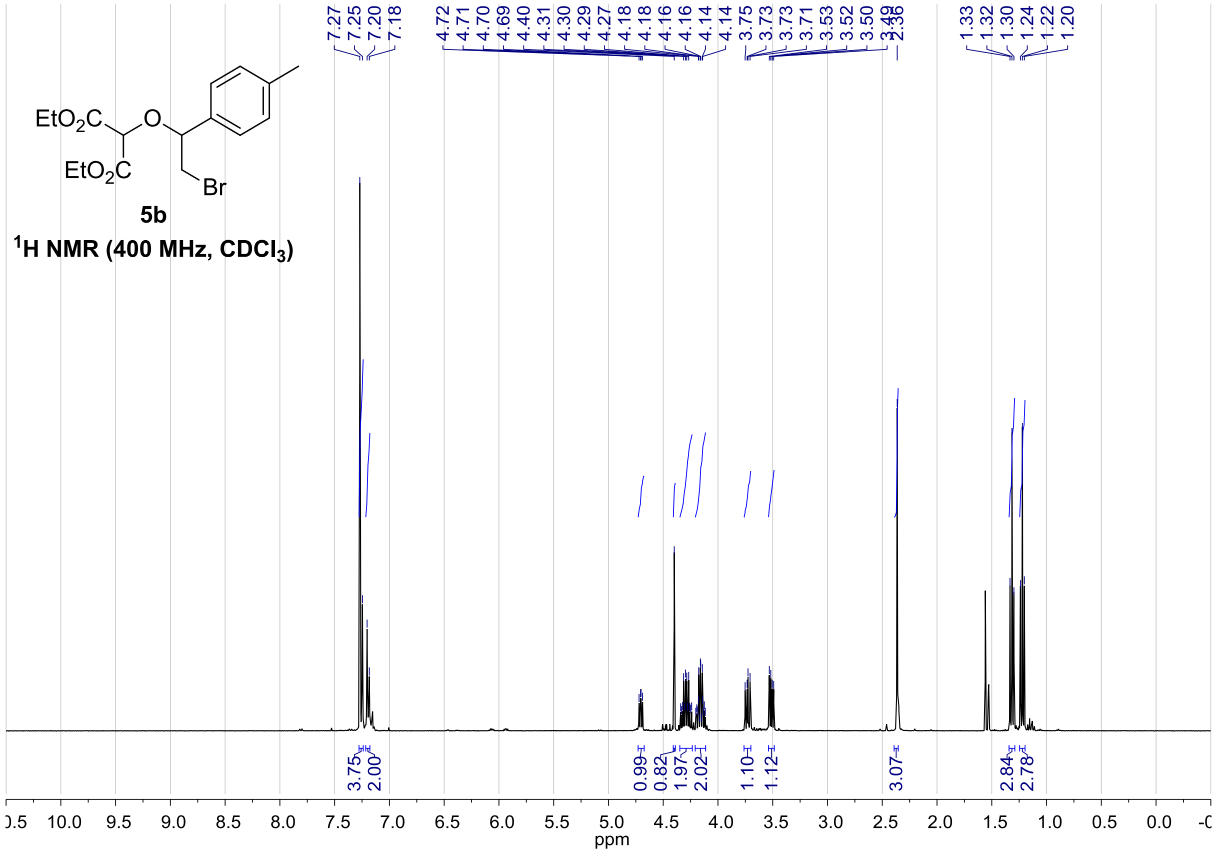
EtO2C
O
EtO2C
Br
5b
1H NMR (400 MHz, CDCl3)
7.27
7.25
7.20
7.18
4.72
4.71
4.70
4.69
4.40
4.31
4.30
4.29
4.27
4.18
4.18
4.16
4.16
4.14
4.14
3.75
3.73
3.73
3.71
3.53
3.52
3.50
3.49
2.36
1.33
1.32
1.30
1.24
1.22
1.20
3.75
2.00
0.99
0.82
1.97
2.02
1.10
1.12
3.07
2.84
2.78
ppm

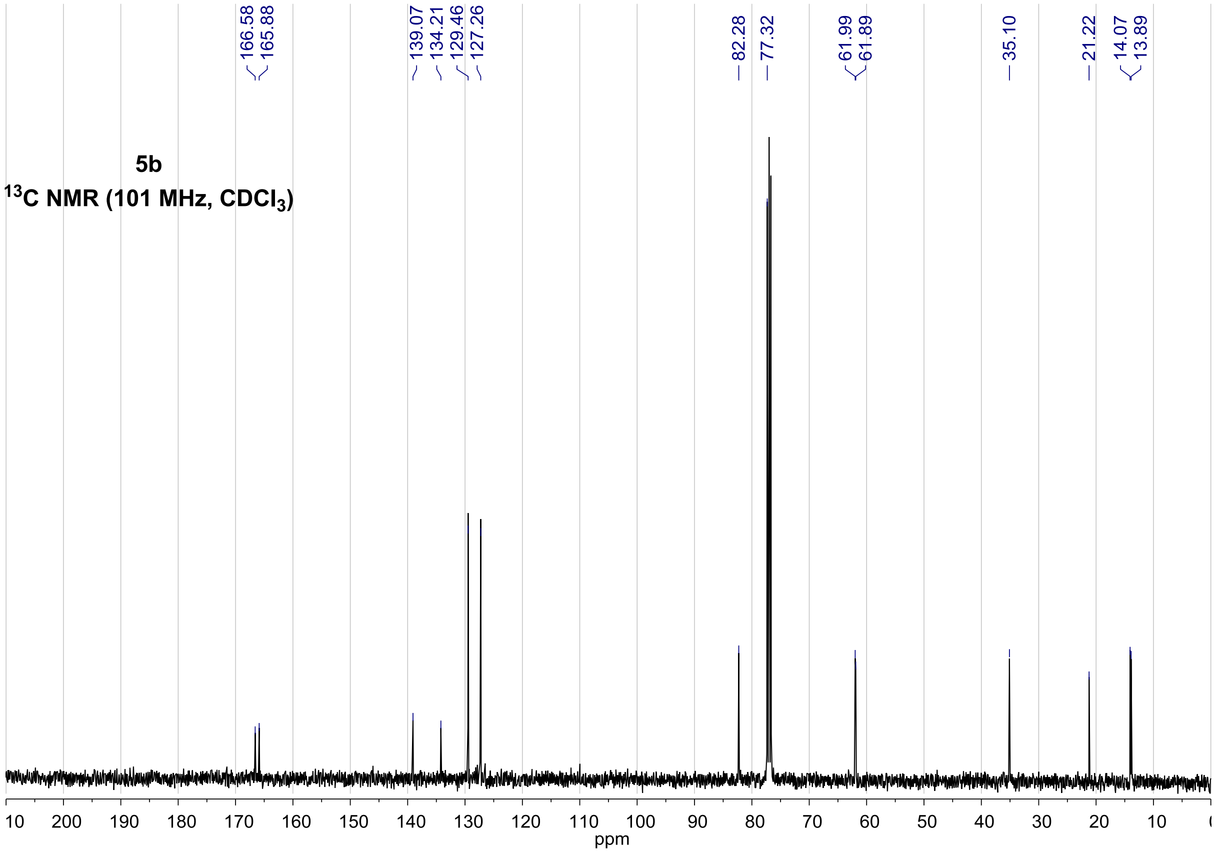
5b
13C NMR (101 MHz, CDCl3)
166.58
165.88
139.07
134.21
129.46
127.26
82.28
77.32
61.99
61.89
35.10
21.22
14.07
13.89
ppm

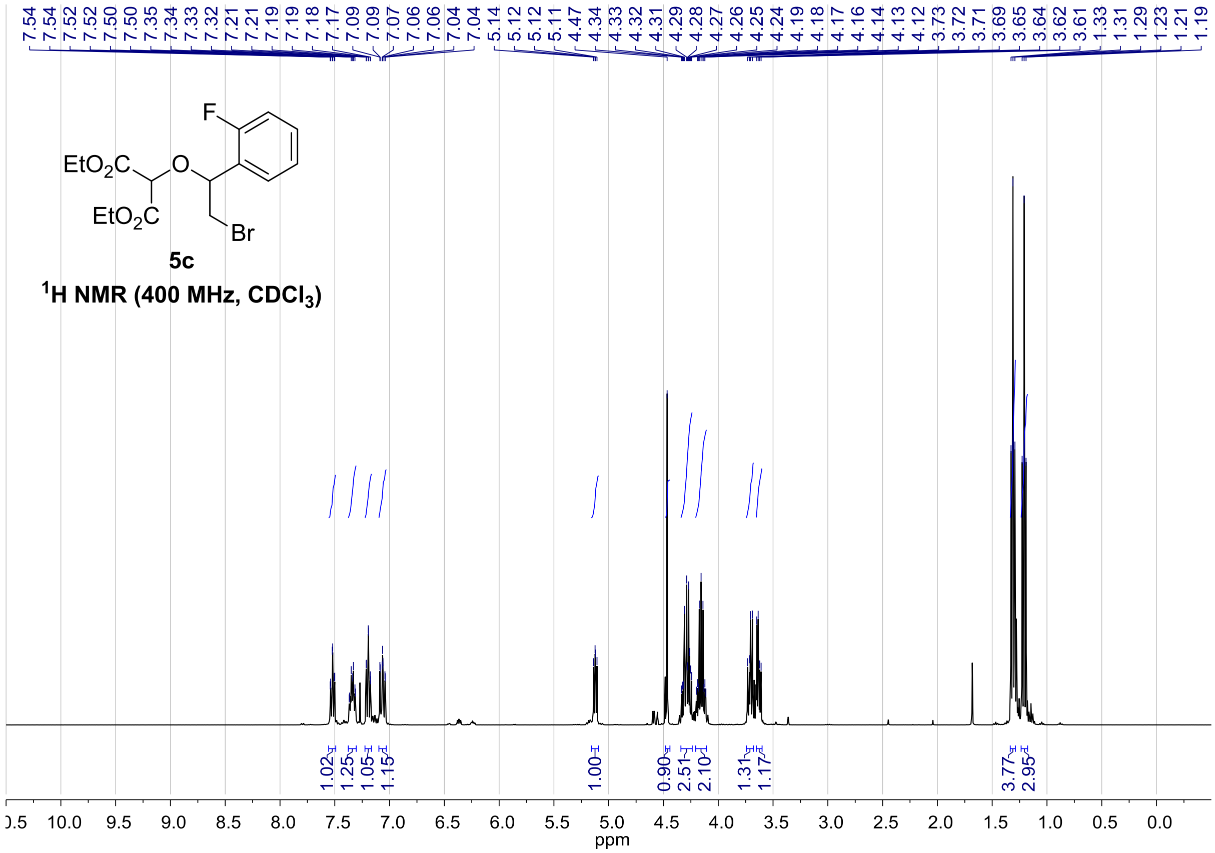
7.54
7.54
7.52
7.52
7.50
7.50
7.35
7.34
7.33
7.32
7.21
7.21
7.19
7.19
7.18
7.17
7.09
7.09
7.07
7.06
7.06
7.04
7.04
5.14
5.12
5.12
5.11
4.47
4.34
4.33
4.32
4.31
4.29
4.28
4.27
4.26
4.25
4.24
4.19
4.18
4.17
4.16
4.14
4.13
4.12
3.73
3.72
3.71
3.69
3.65
3.64
3.62
3.61
1.33
1.31
1.29
1.23
1.21
1.19
F
EtO_2C
O
EtO_2C
Br
5c
^{1}H NMR (400 MHz, $CDCl_3$)
1.02
1.25
1.05
1.15
1.00
0.90
2.51
2.10
1.31
1.17
3.77
2.95
ppm

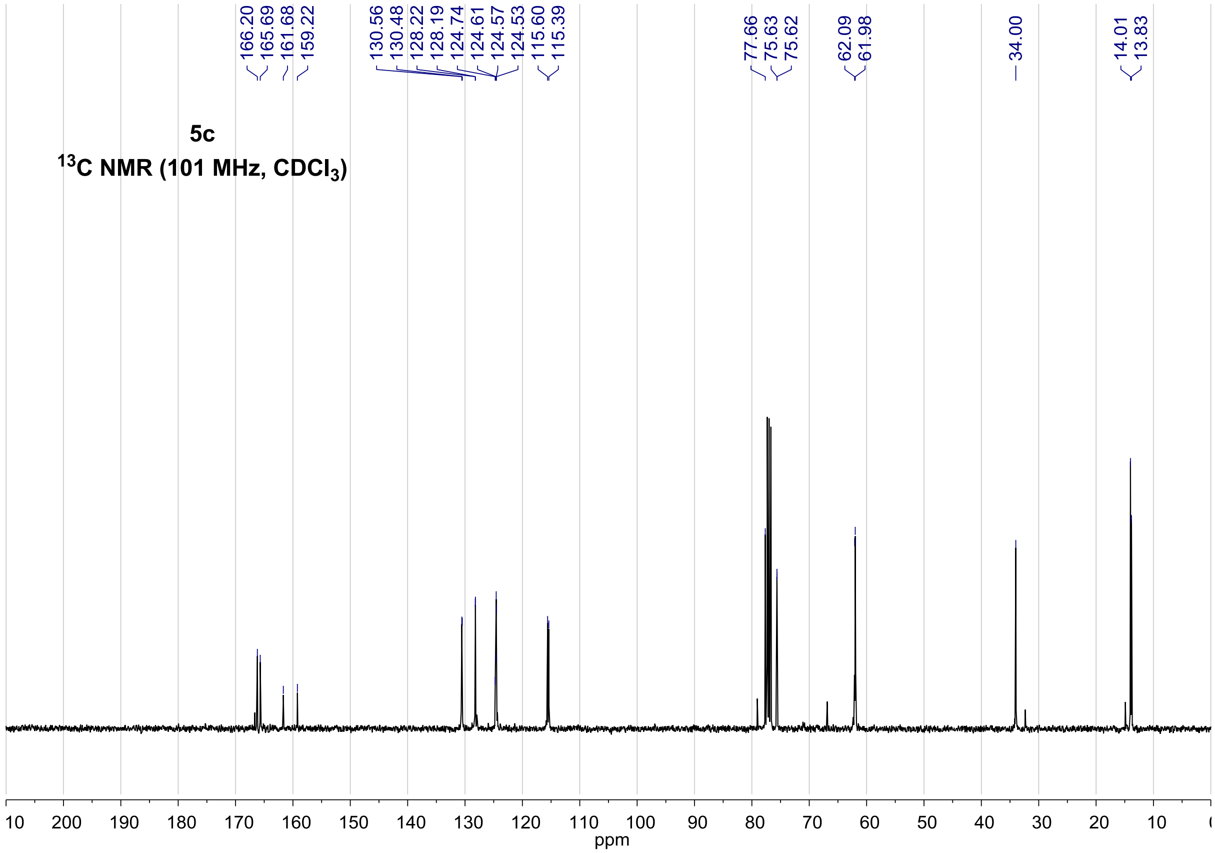
166.20
165.69
161.68
159.22
130.56
130.48
128.22
128.19
124.74
124.61
124.57
124.53
115.60
115.39
77.66
75.63
75.62
62.09
61.98
34.00
14.01
13.83
5c
^{13}C NMR (101 MHz, $CDCl_3$)
ppm

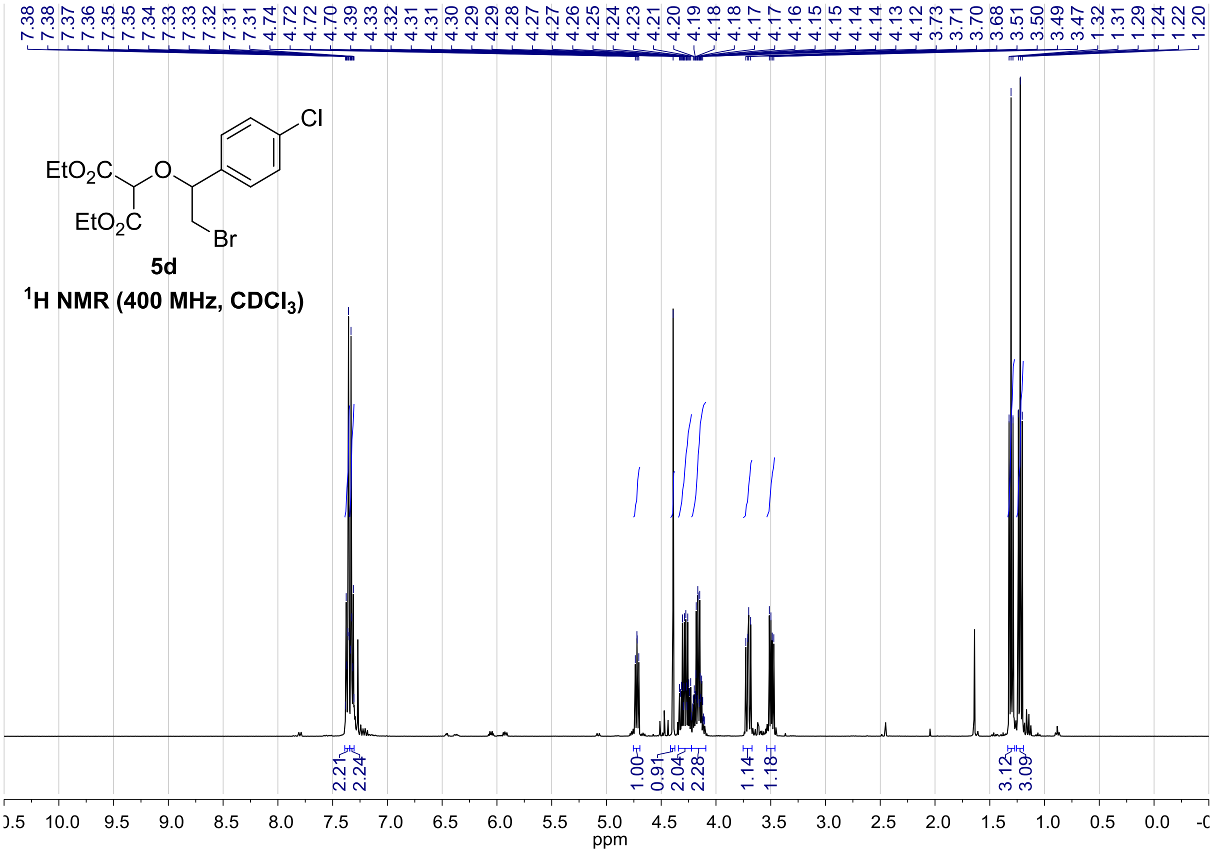

Cl
EtO2C
O
EtO2C
Br
5d
1H NMR (400 MHz, CDCl3)
7.38 7.38 7.37 7.36 7.35 7.35 7.34 7.33 7.33 7.32 7.31 7.31 4.74 4.72 4.72 4.70 4.39 4.33 4.32 4.31 4.31 4.30 4.29 4.29 4.28 4.27 4.27 4.26 4.25 4.24 4.23 4.21 4.20 4.19 4.18 4.18 4.17 4.17 4.16 4.15 4.15 4.14 4.14 4.13 4.12 3.73 3.71 3.70 3.68 3.51 3.50 3.49 3.47 1.32 1.31 1.29 1.24 1.22 1.20
2.21 2.24 1.00 0.91 2.04 2.28 1.14 1.18 3.12 3.09
ppm

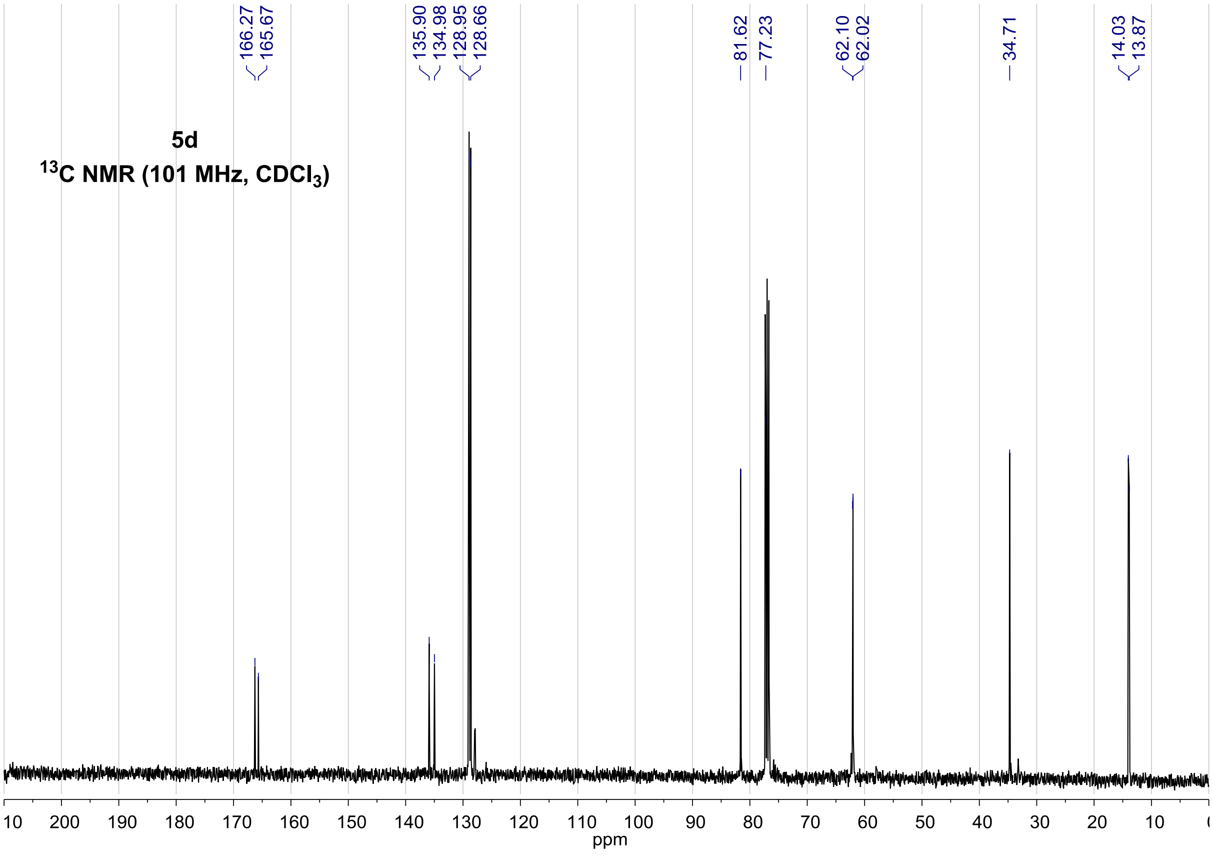

166.27 165.67 135.90 134.98 128.95 128.66 81.62 77.23 62.10 62.02 34.71 14.03 13.87
5d
13C NMR (101 MHz, CDCl3)
ppm

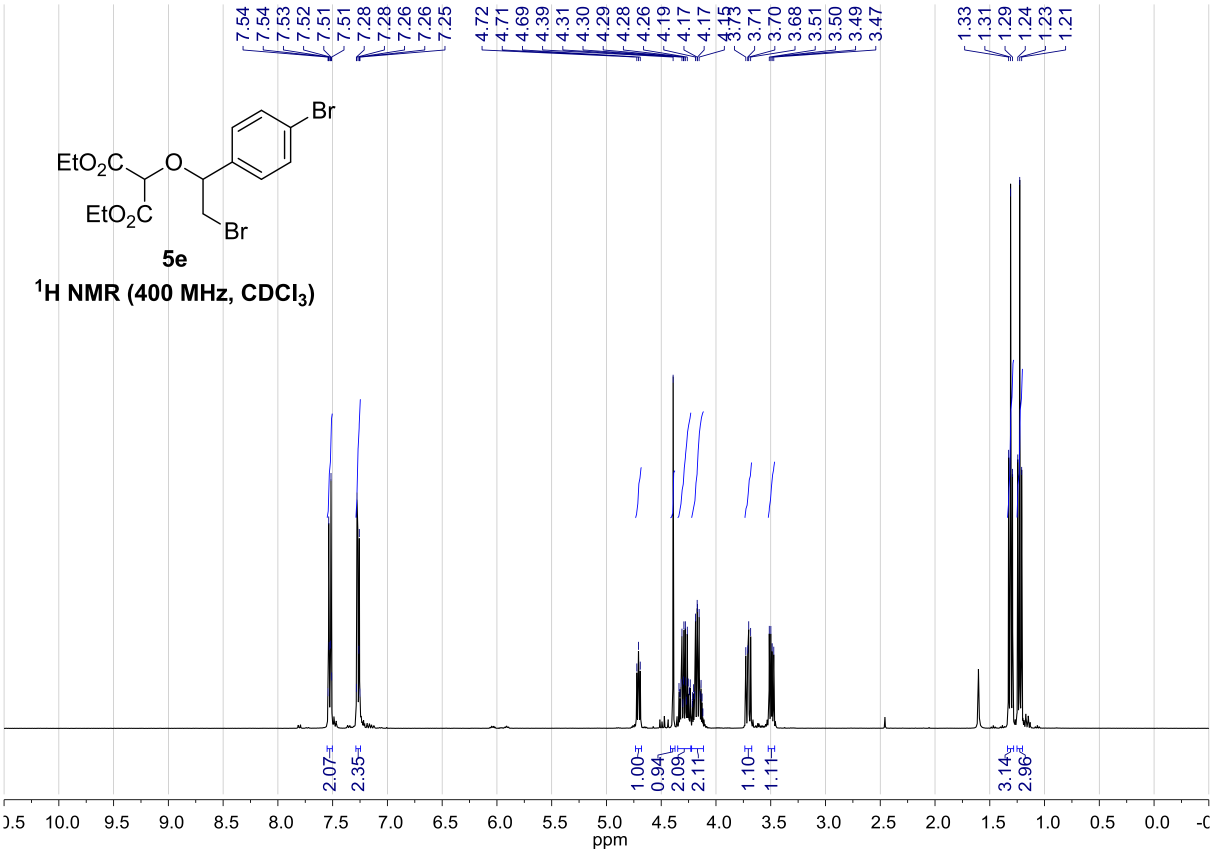
EtO2C
O
Br
EtO2C
Br
5e
1H NMR (400 MHz, CDCl3)
ppm

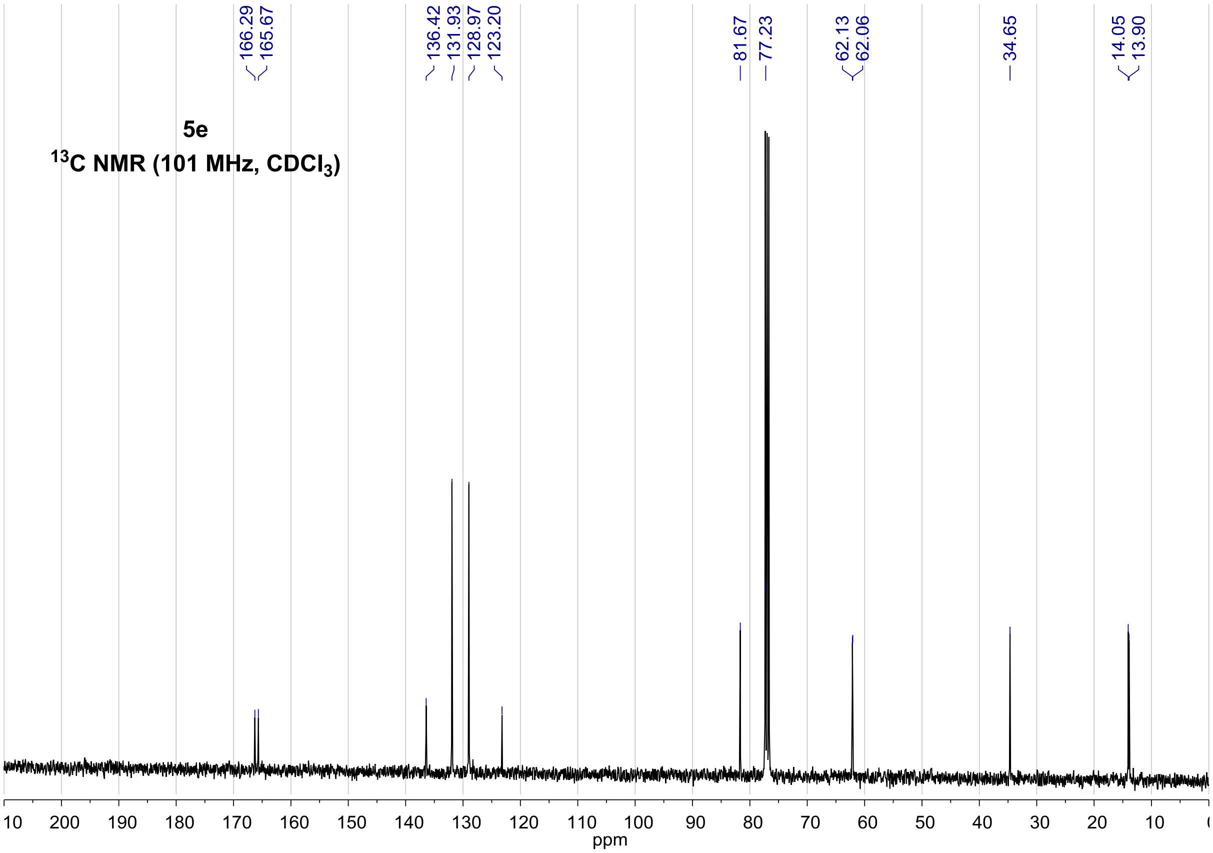
5e
13C NMR (101 MHz, CDCl3)
166.29
165.67
136.42
131.93
128.97
123.20
81.67
77.23
62.13
62.06
34.65
14.05
13.90
ppm

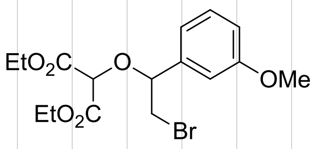

5f

^{1}H NMR (400 MHz, CDCl$_3$)

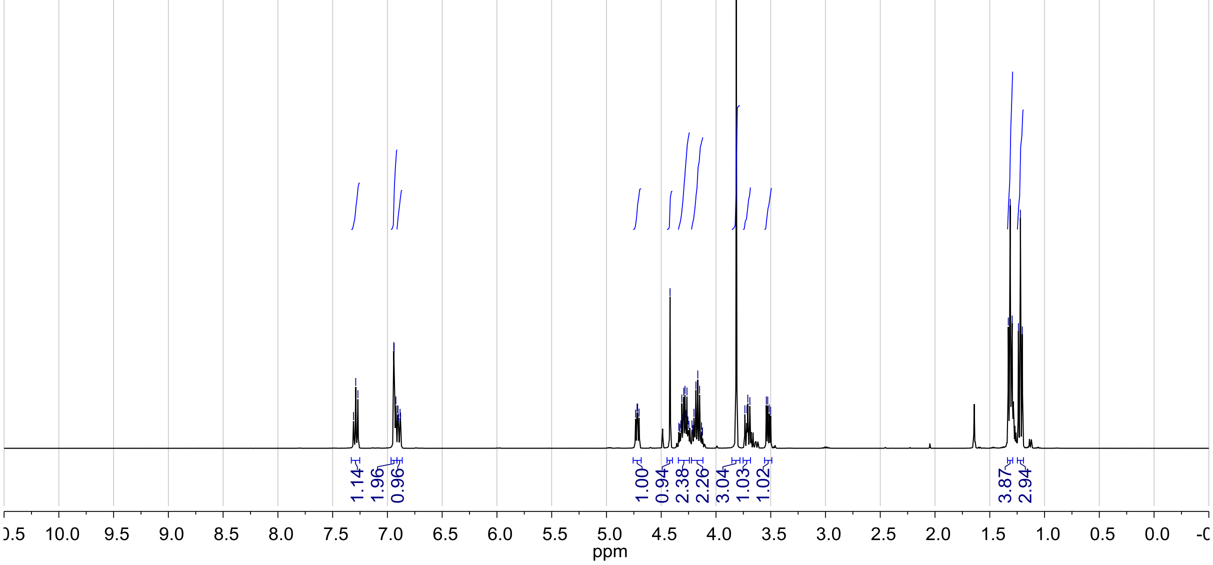

5f

^{13}C NMR (101 MHz, CDCl$_3$)

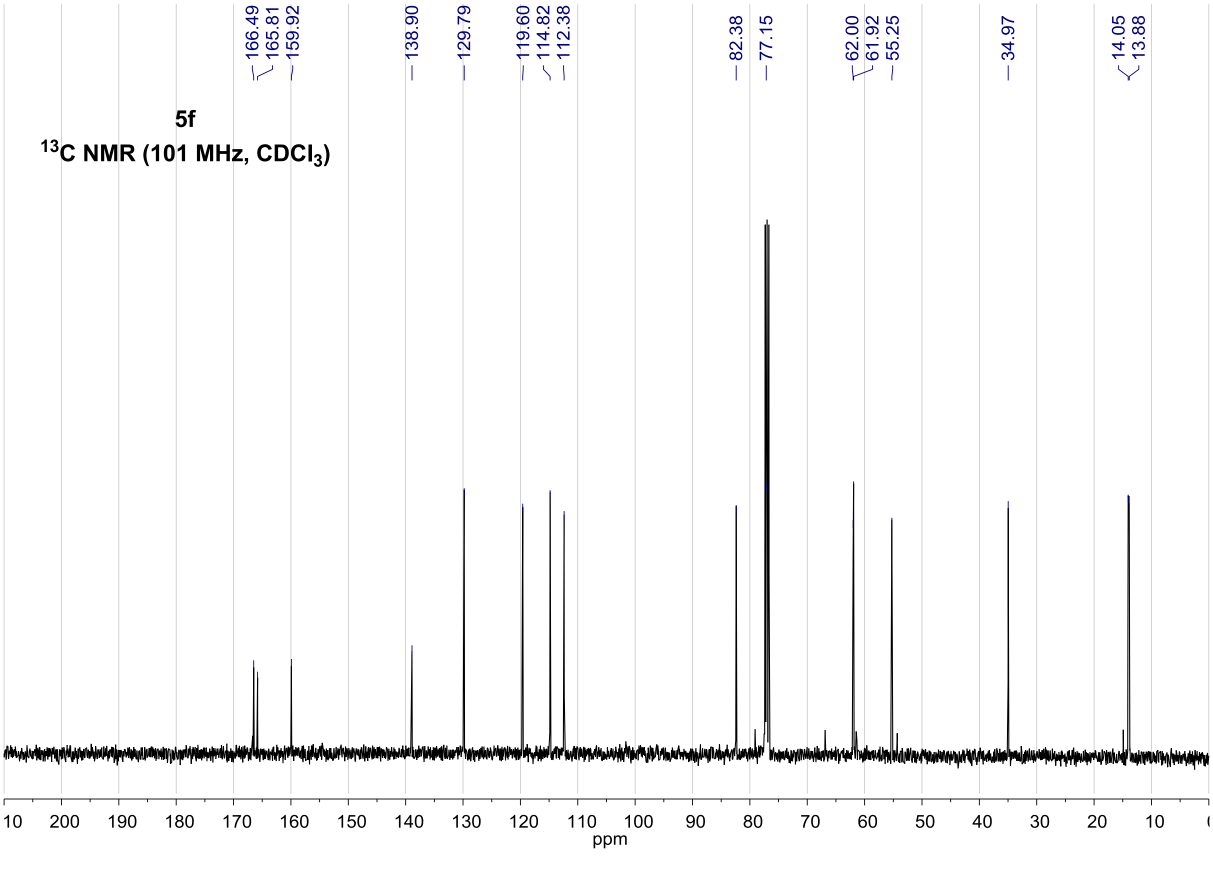

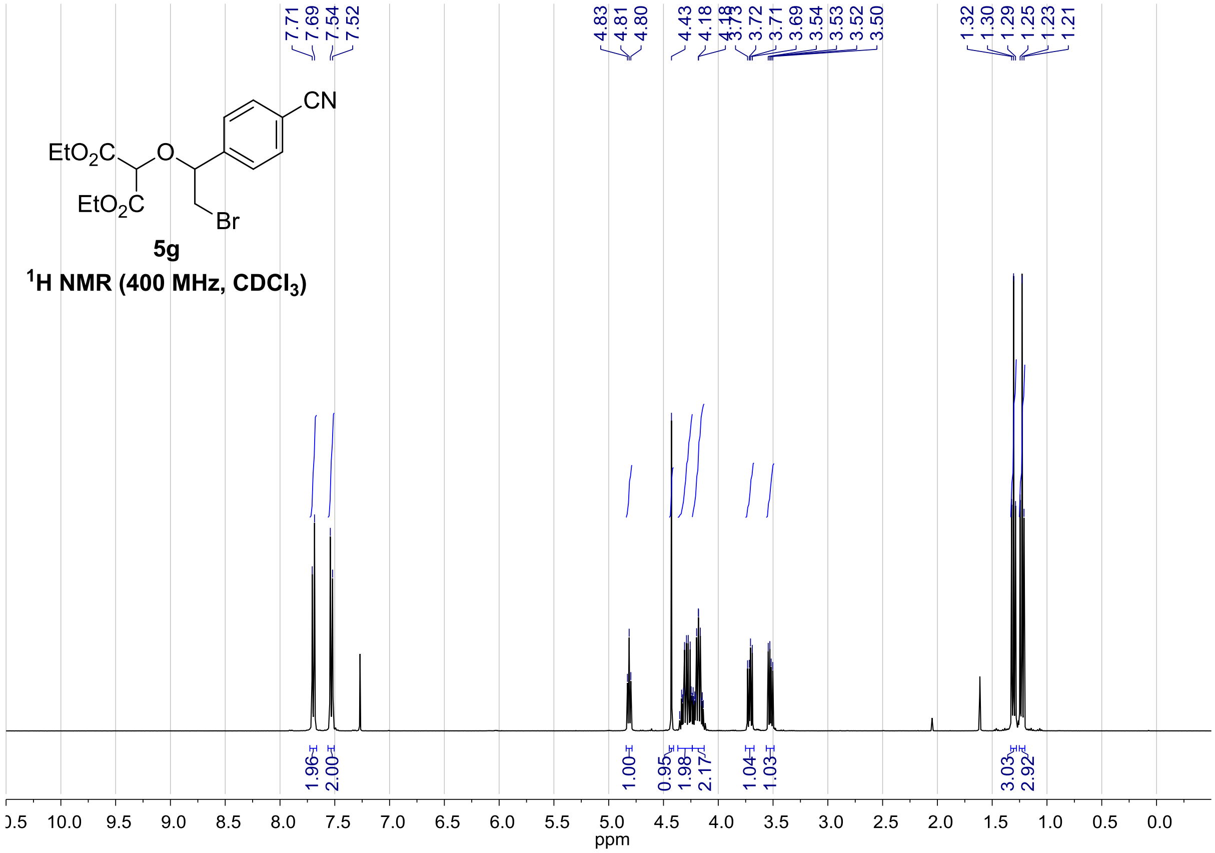
7.71
7.69
7.54
7.52
4.83
4.81
4.80
4.43
4.18
3.73
3.72
3.71
3.69
3.54
3.53
3.52
3.50
1.32
1.30
1.29
1.25
1.23
1.21
CN
EtO2C
O
EtO2C
Br
5g
1H NMR (400 MHz, CDCl3)
1.96
2.00
1.00
0.95
1.98
2.17
1.04
1.03
3.03
2.92
ppm

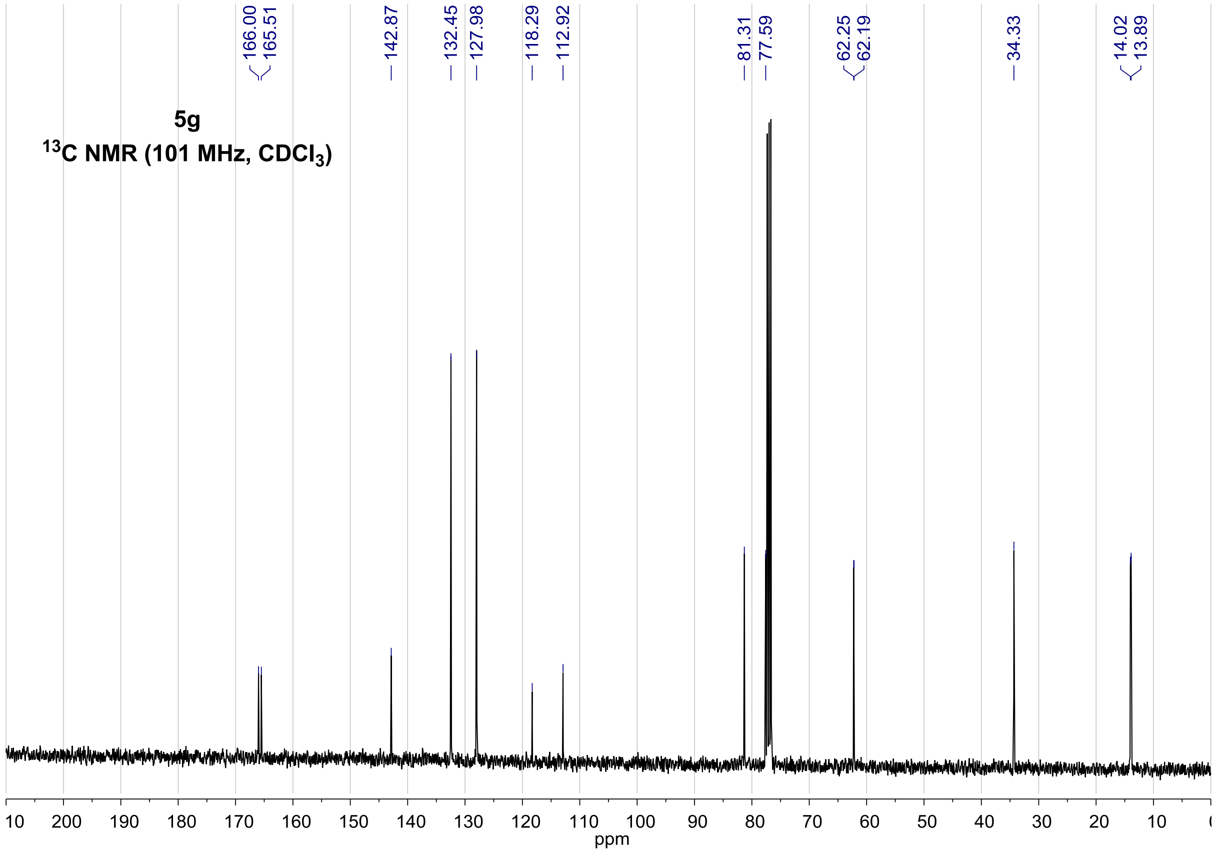
166.00
165.51
142.87
132.45
127.98
118.29
112.92
81.31
77.59
62.25
62.19
34.33
14.02
13.89
5g
13C NMR (101 MHz, CDCl3)
ppm

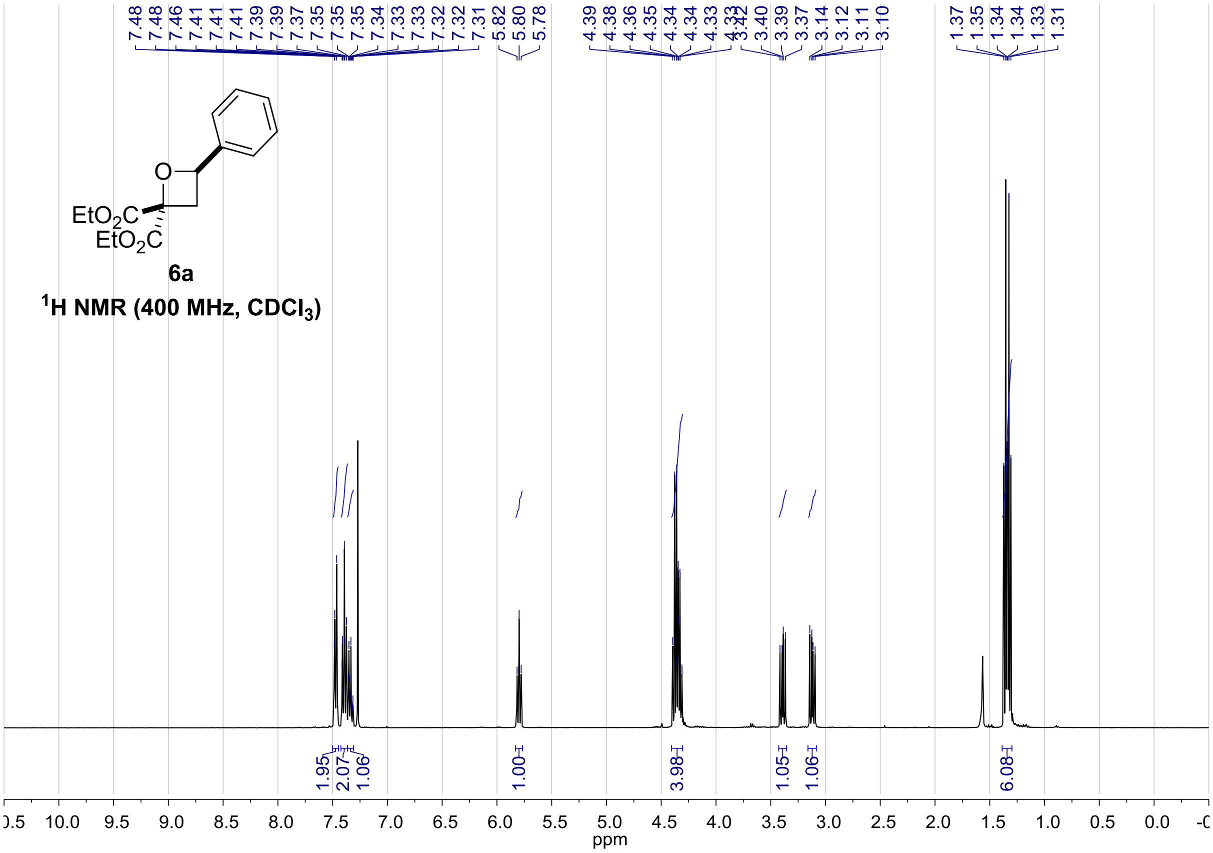
7.48 7.48 7.46 7.41 7.41 7.41 7.39 7.39 7.37 7.35 7.35 7.35 7.34 7.33 7.33 7.32 7.32 7.31 5.82 5.80 5.78 4.39 4.38 4.36 4.35 4.34 4.34 4.33 4.33 3.42 3.40 3.39 3.37 3.14 3.12 3.11 3.10 1.37 1.35 1.34 1.34 1.33 1.31
EtO2C
EtO2C
O
6a
1H NMR (400 MHz, CDCl3)
1.95 2.07 1.06 1.00 3.98 1.05 1.06 6.08
ppm

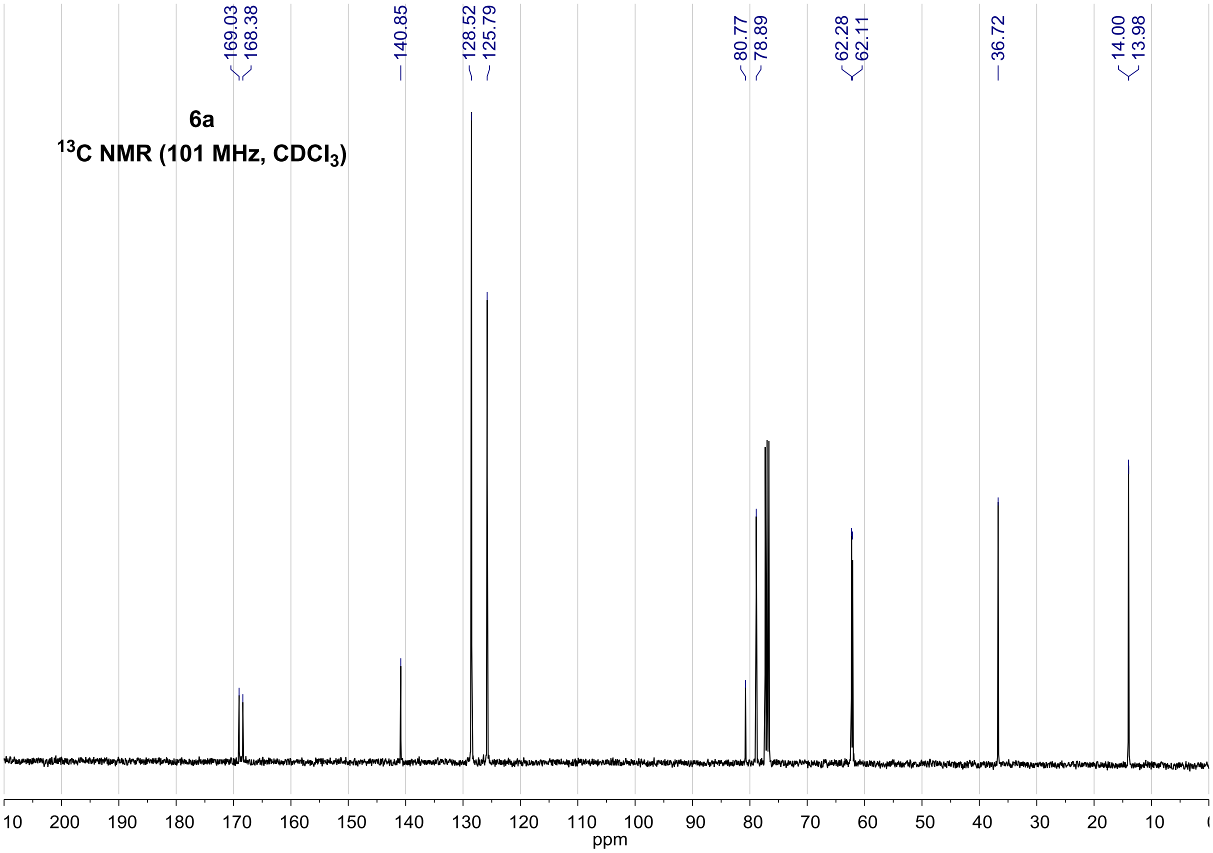
169.03 168.38 140.85 128.52 125.79 80.77 78.89 62.28 62.11 36.72 14.00 13.98
6a
13C NMR (101 MHz, CDCl3)
ppm

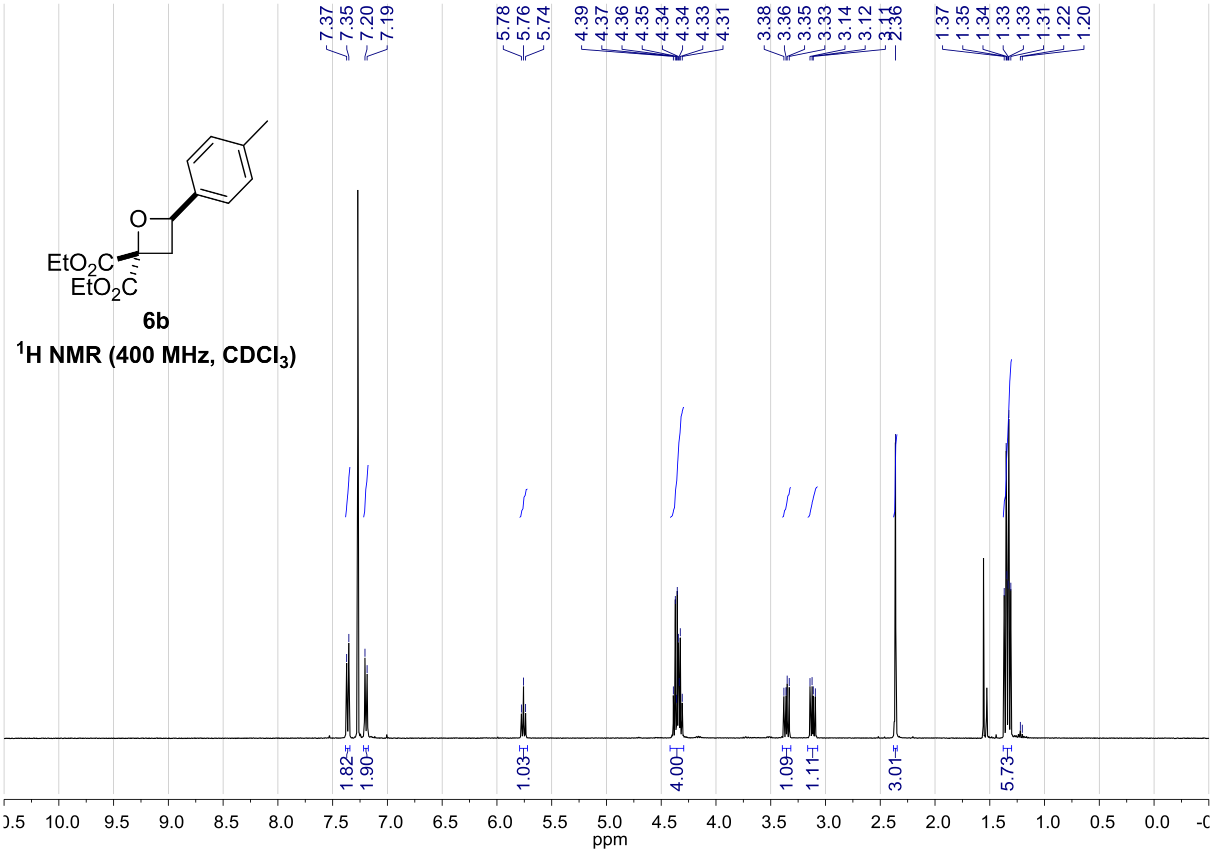
7.37
7.35
7.20
7.19
5.78
5.76
5.74
4.39
4.37
4.36
4.35
4.34
4.34
4.33
4.31
3.38
3.36
3.35
3.33
3.14
3.12
3.11
2.36
1.37
1.35
1.34
1.33
1.33
1.31
1.22
1.20
O
EtO2C
EtO2C
6b
1H NMR (400 MHz, CDCl3)
1.82
1.90
1.03
4.00
1.09
1.11
3.01
5.73
ppm

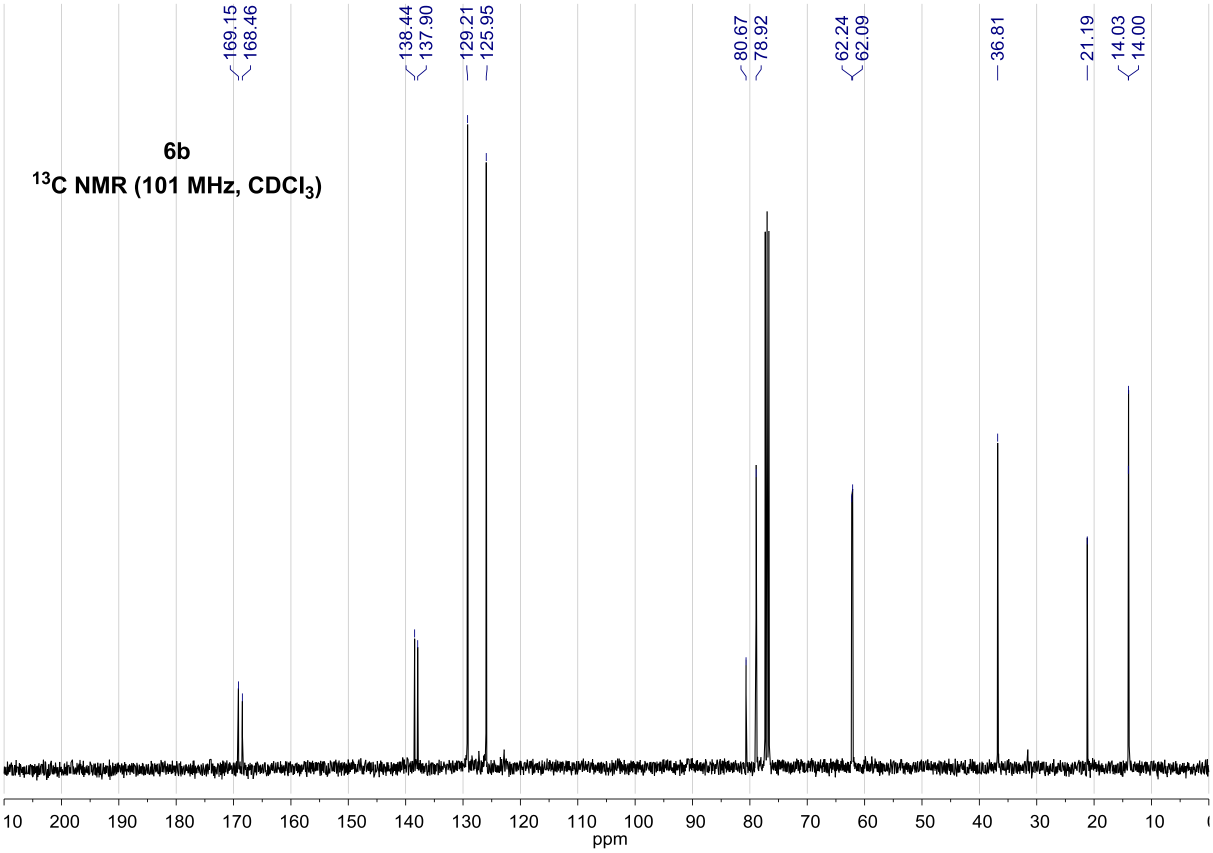
169.15
168.46
138.44
137.90
129.21
125.95
80.67
78.92
62.24
62.09
36.81
21.19
14.03
14.00
6b
13C NMR (101 MHz, CDCl3)
ppm

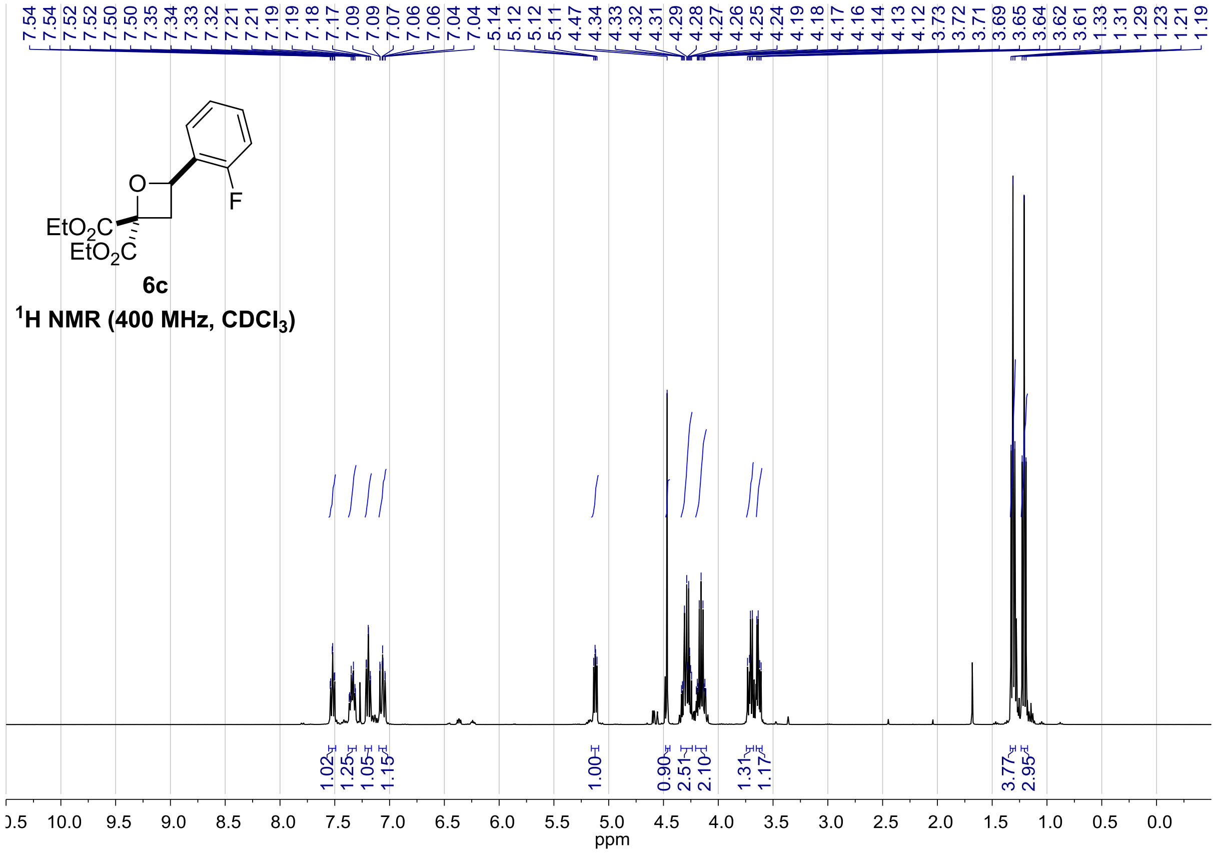
6c
^{1}H NMR (400 MHz, CDCl$_3$)
EtO$_2$C
EtO$_2$C
O
F
ppm

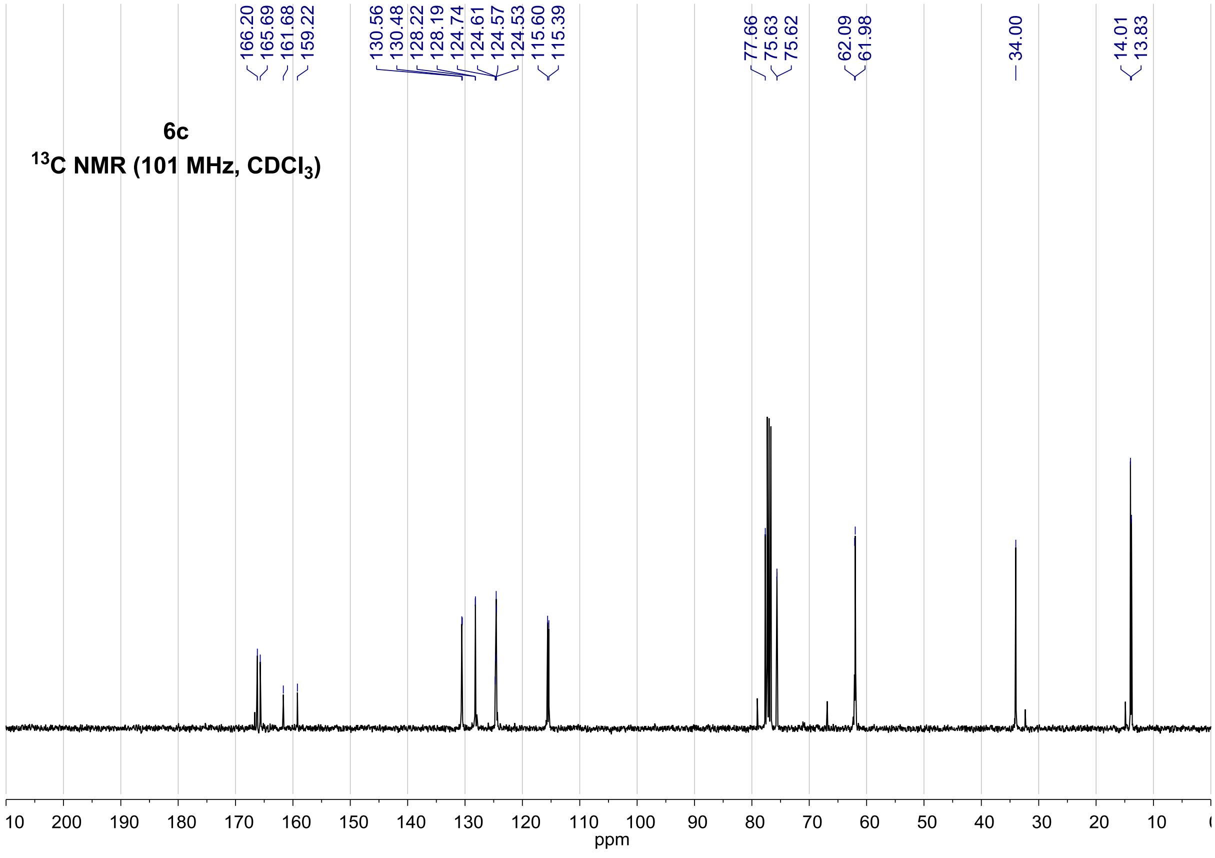
6c
^{13}C NMR (101 MHz, CDCl$_3$)
ppm

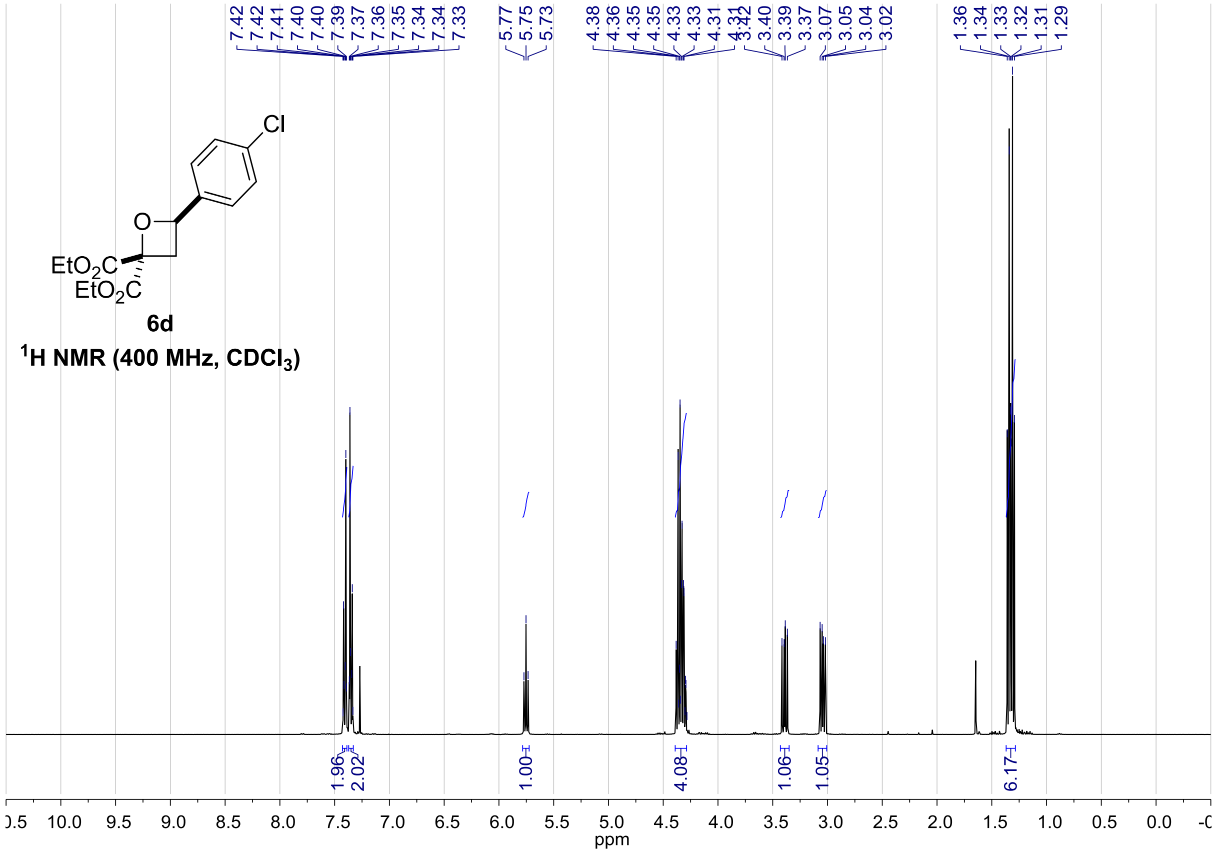

6d

^{1}H NMR (400 MHz, $CDCl_3$)

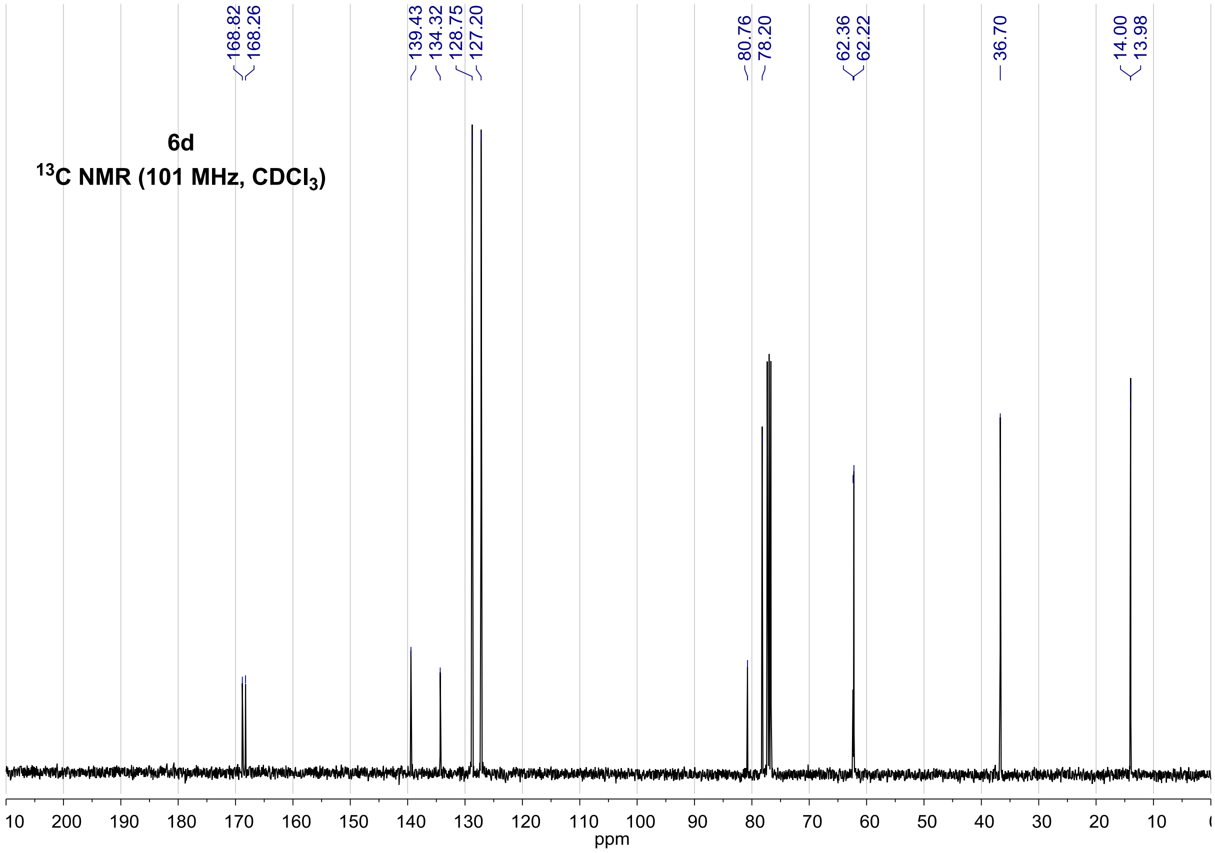

6d

^{13}C NMR (101 MHz, $CDCl_3$)

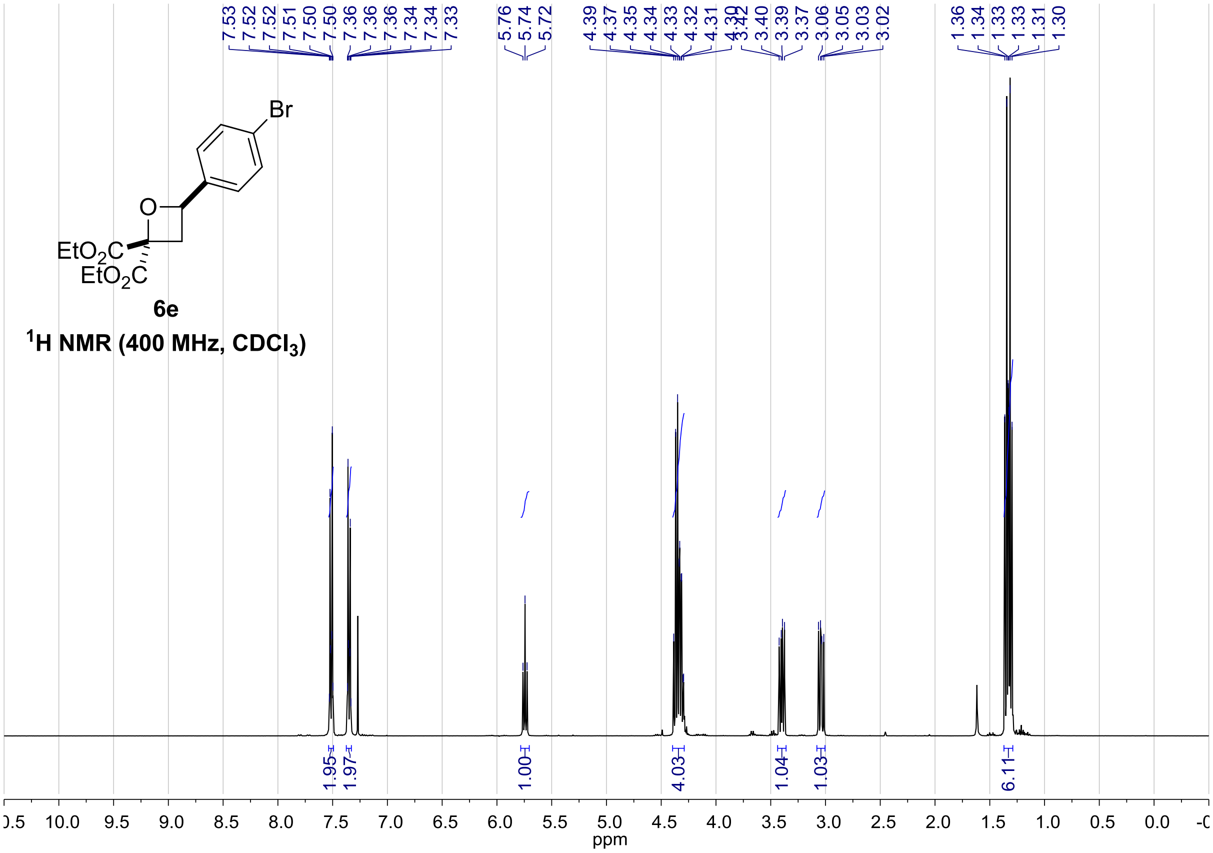
7.53
7.52
7.52
7.51
7.50
7.50
7.36
7.36
7.36
7.34
7.34
7.33
5.76
5.74
5.72
4.39
4.37
4.35
4.34
4.33
4.32
4.31
4.30
3.42
3.40
3.39
3.37
3.06
3.05
3.03
3.02
1.36
1.34
1.33
1.33
1.31
1.30
Br
O
EtO2C
EtO2C
6e
1H NMR (400 MHz, CDCl3)
1.95
1.97
1.00
4.03
1.04
1.03
6.11
ppm

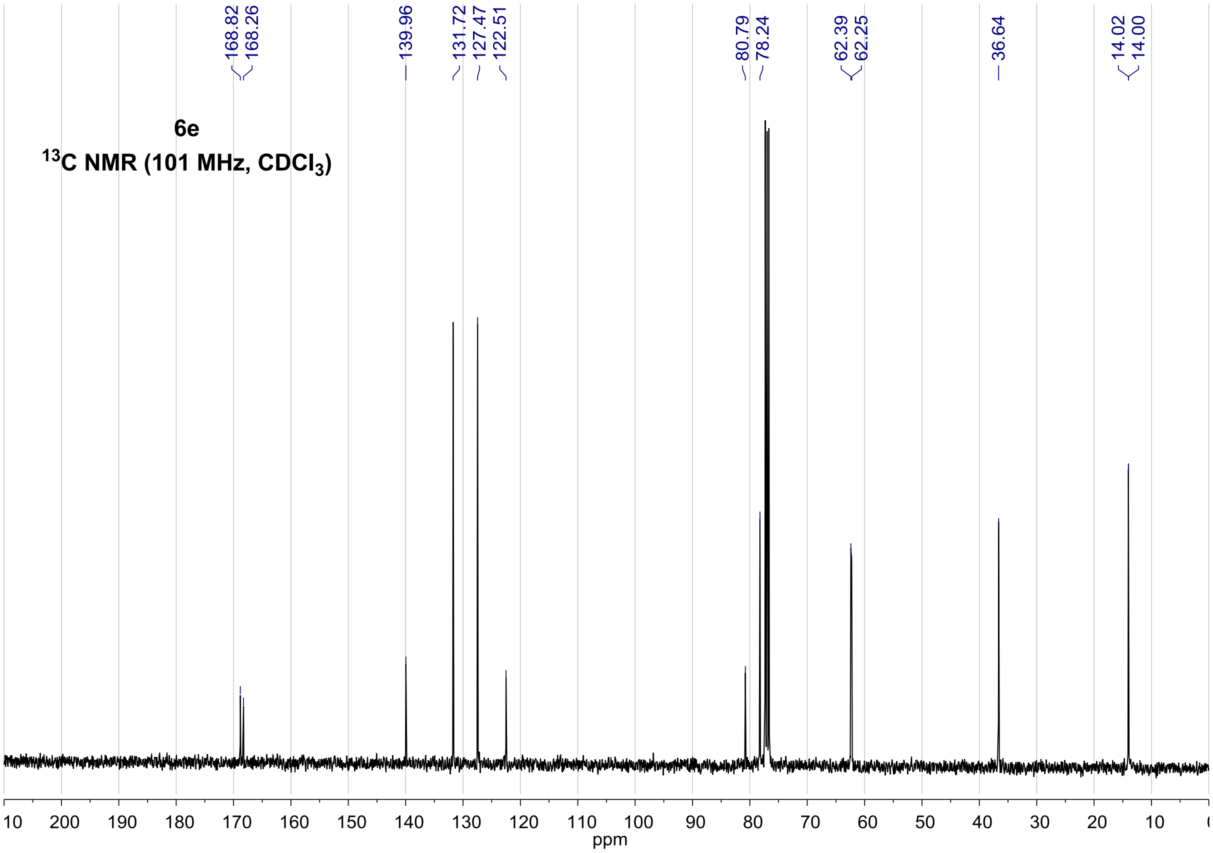
168.82
168.26
139.96
131.72
127.47
122.51
80.79
78.24
62.39
62.25
36.64
14.02
14.00
6e
13C NMR (101 MHz, CDCl3)
ppm

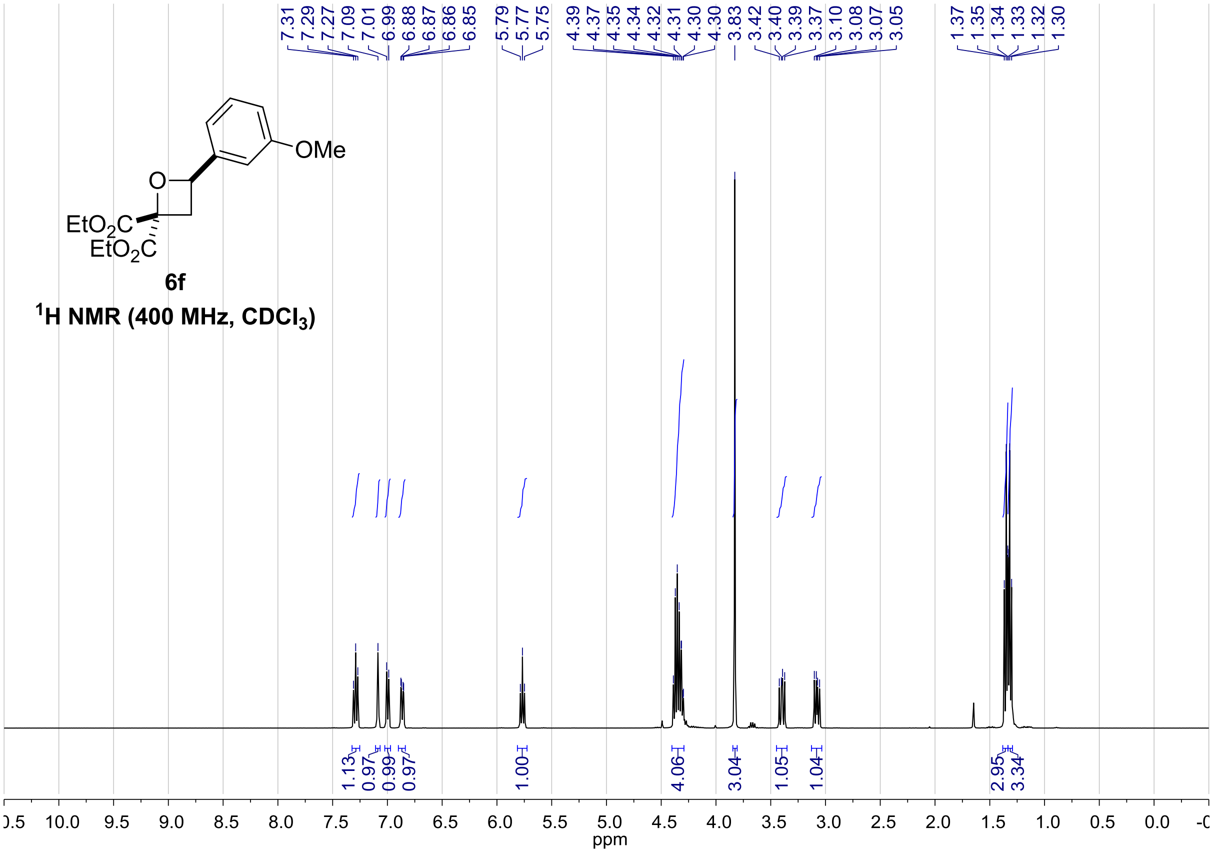
7.31
7.29
7.27
7.09
7.01
6.99
6.88
6.87
6.86
6.85
5.79
5.77
5.75
4.39
4.37
4.35
4.34
4.32
4.31
4.30
4.30
3.83
3.42
3.40
3.39
3.37
3.10
3.08
3.07
3.05
1.37
1.35
1.34
1.33
1.32
1.30
OMe
O
EtO2C
EtO2C
6f
1H NMR (400 MHz, CDCl3)
1.13
0.97
0.99
0.97
1.00
4.06
3.04
1.05
1.04
2.95
3.34
ppm

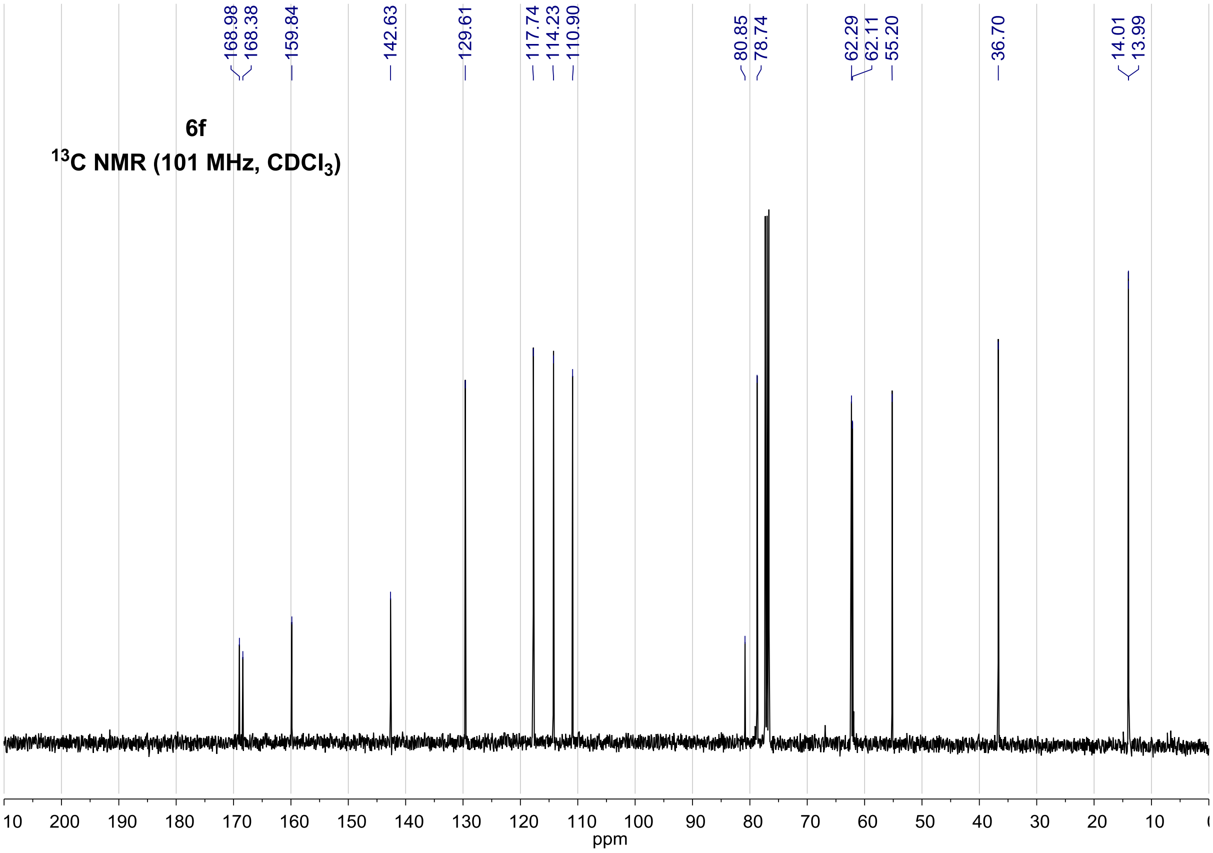
168.98
168.38
159.84
142.63
129.61
117.74
114.23
110.90
80.85
78.74
62.29
62.11
55.20
36.70
14.01
13.99
6f
13C NMR (101 MHz, CDCl3)
ppm

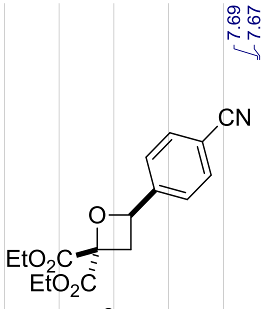

6g

^{1}H NMR (400 MHz, $CDCl_3$)

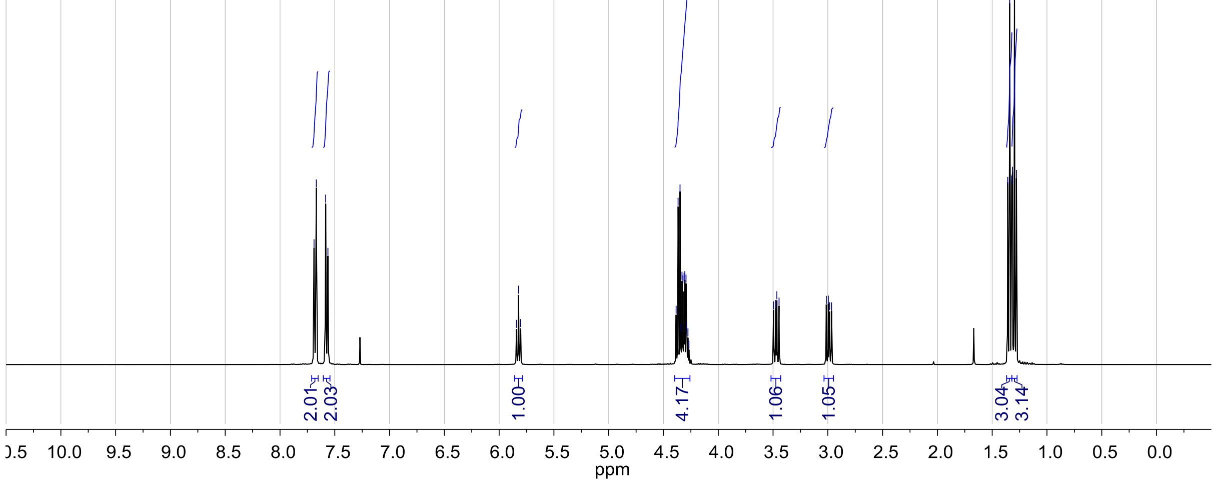

6g

^{13}C NMR (101 MHz, $CDCl_3$)

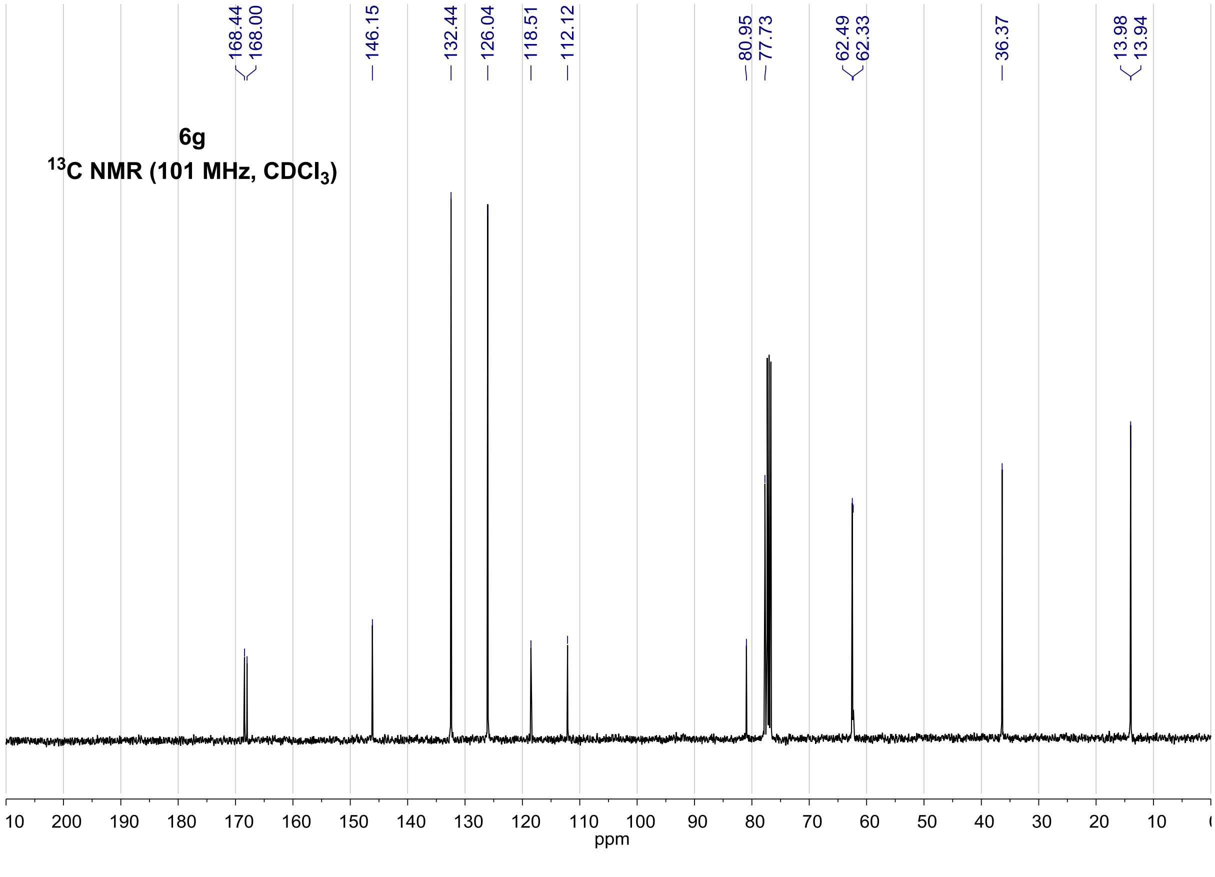

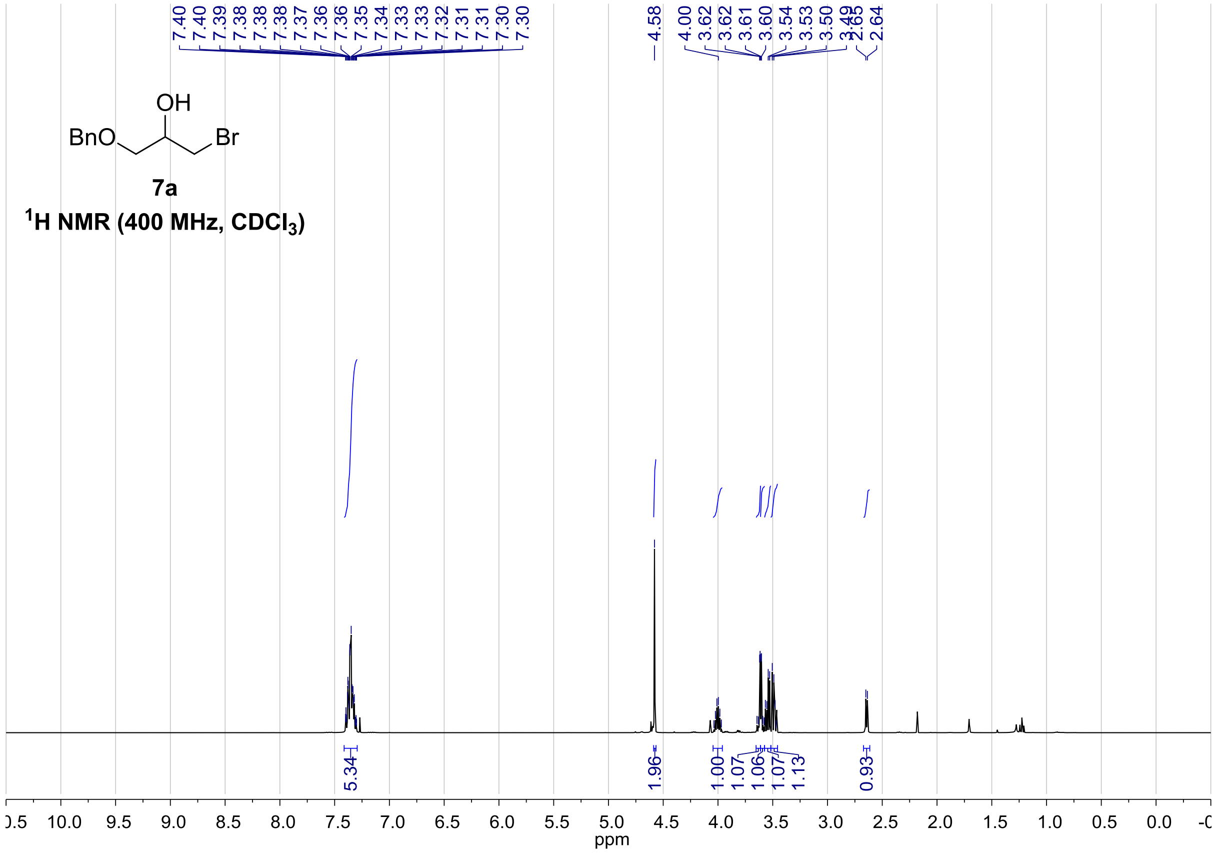
7.40 7.40 7.39 7.38 7.38 7.38 7.37 7.36 7.36 7.35 7.34 7.33 7.33 7.32 7.31 7.31 7.30 7.30
4.58
4.00 3.62 3.62 3.61 3.60 3.54 3.53 3.50 3.49 2.65 2.64
OH
BnO
Br
7a
1H NMR (400 MHz, CDCl3)
5.34
1.96
1.00 1.07 1.06 1.07 1.13
0.93
ppm

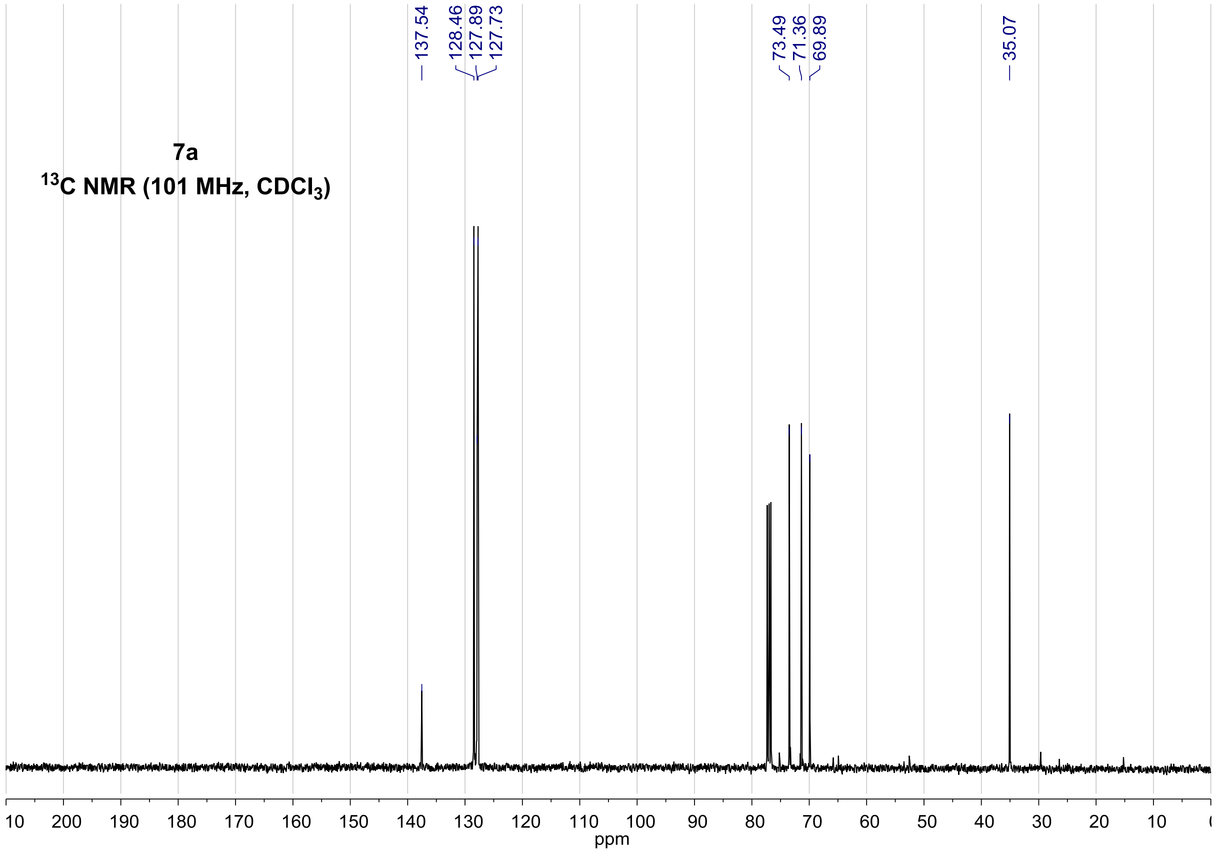
137.54
128.46 127.89 127.73
73.49 71.36 69.89
35.07
7a
13C NMR (101 MHz, CDCl3)
ppm

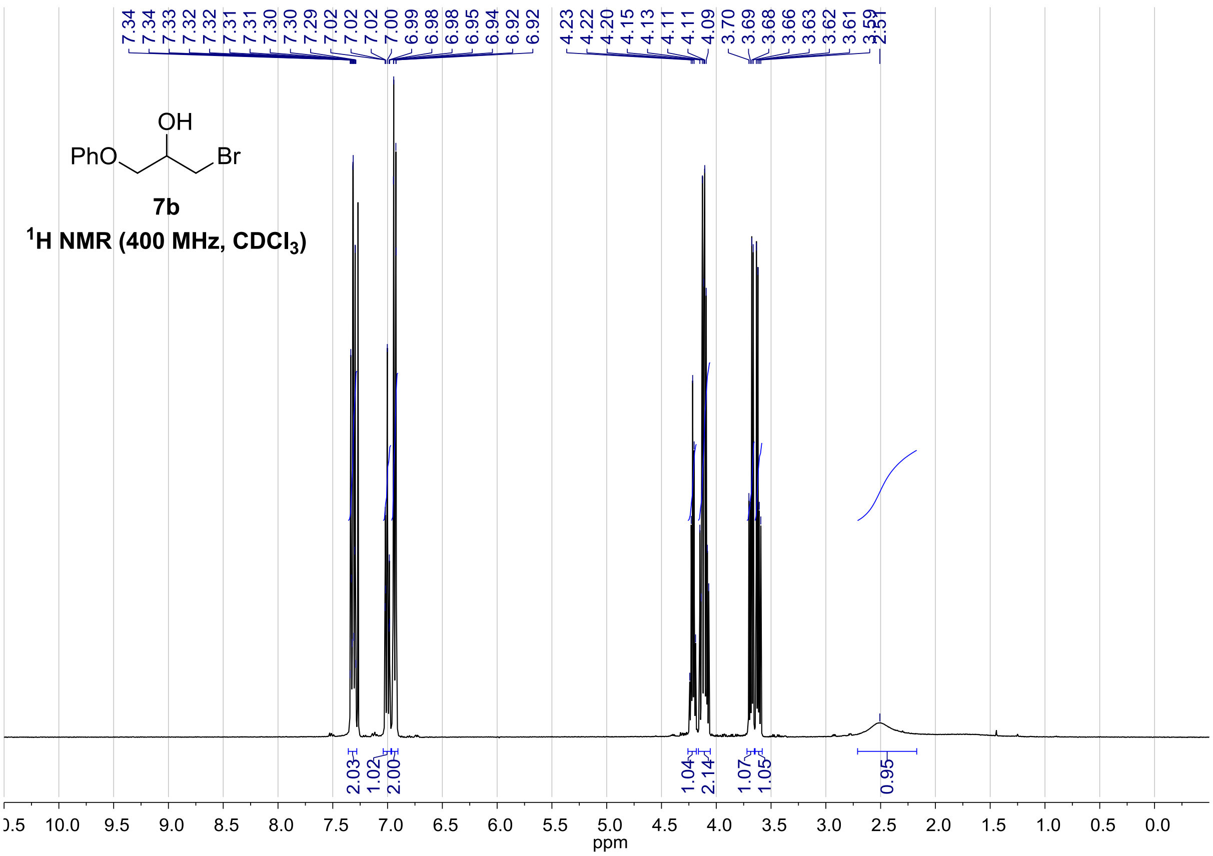
PhO OH Br
7b
1H NMR (400 MHz, CDCl3)
ppm

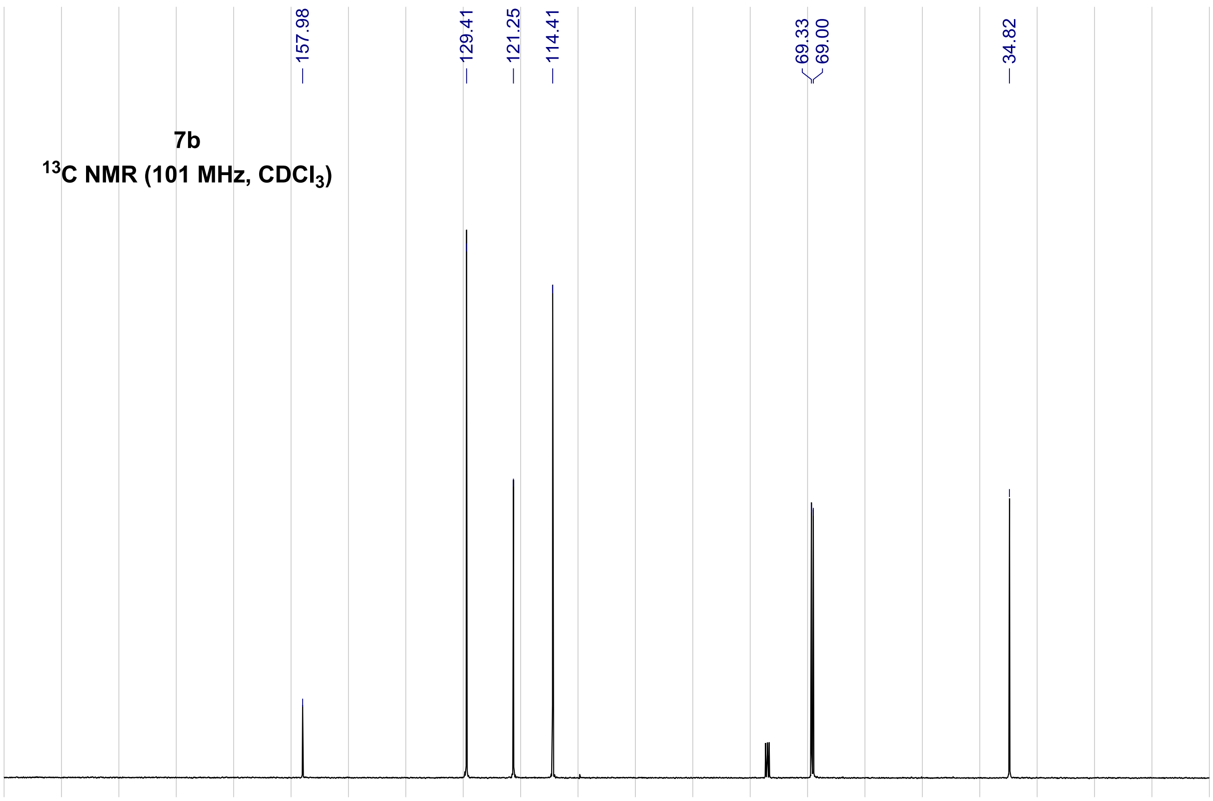
157.98
129.41
121.25
114.41
69.33
69.00
34.82
7b
13C NMR (101 MHz, CDCl3)
ppm

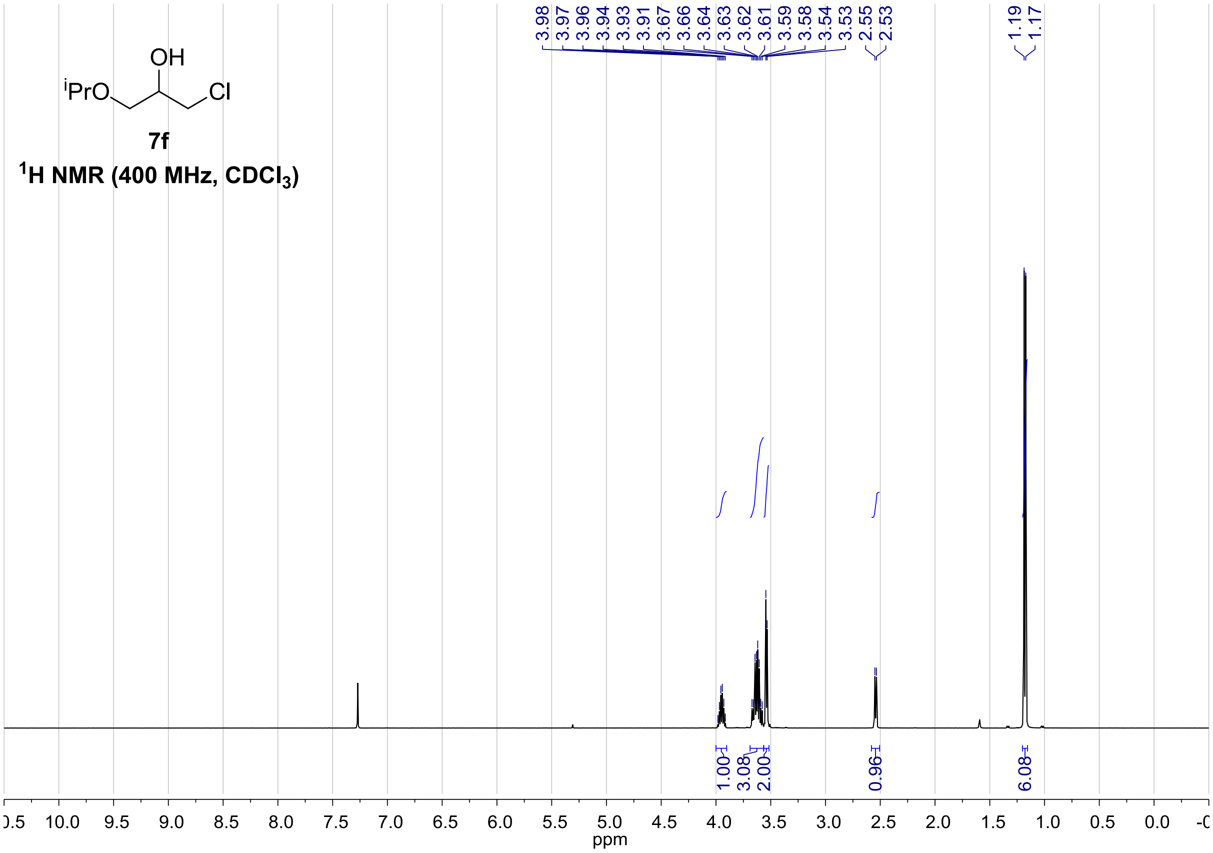
OH
iPrO
Cl
7f
1H NMR (400 MHz, CDCl3)
3.98
3.97
3.96
3.94
3.93
3.91
3.67
3.66
3.64
3.63
3.62
3.61
3.59
3.58
3.54
3.53
2.55
2.53
1.19
1.17
1.00
3.08
2.00
0.96
6.08
ppm

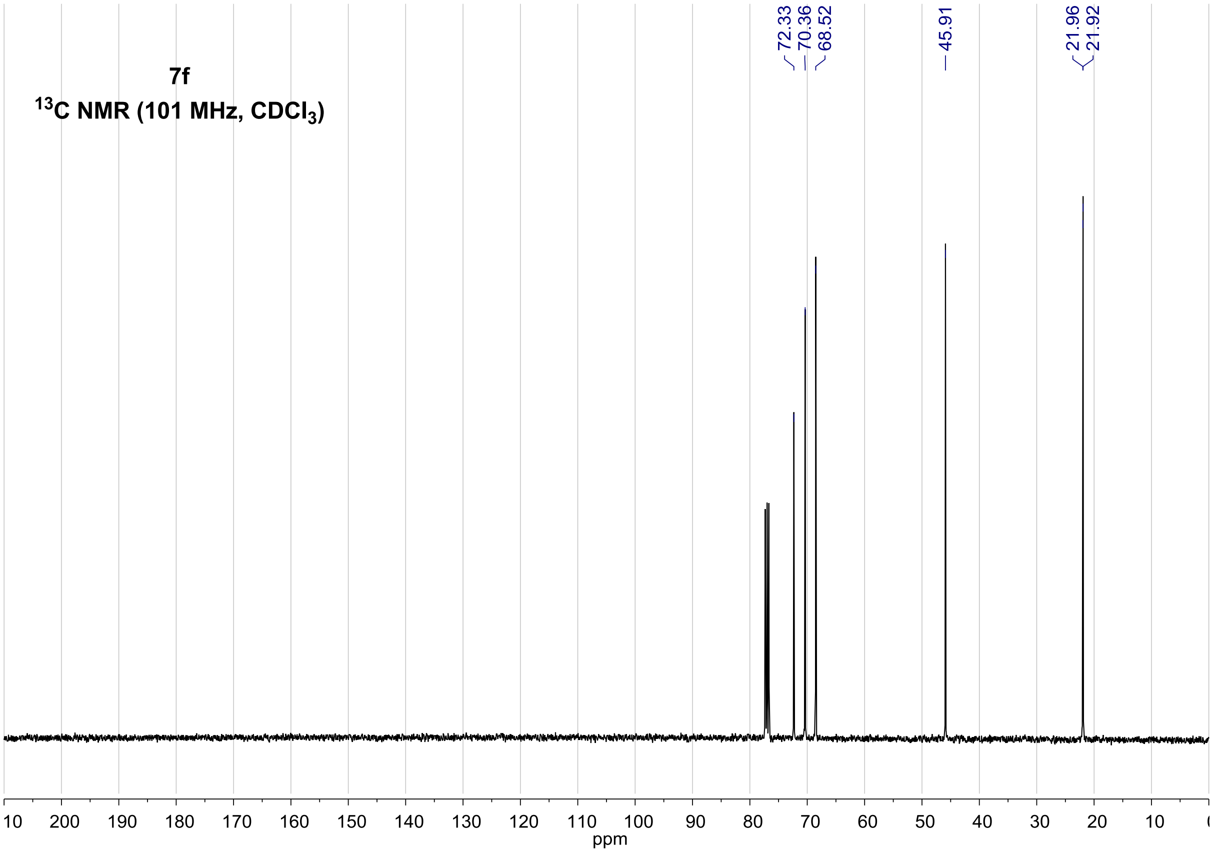
7f
13C NMR (101 MHz, CDCl3)
72.33
70.36
68.52
45.91
21.96
21.92
ppm

7g

^{1}H NMR (400 MHz, $CDCl_3$)

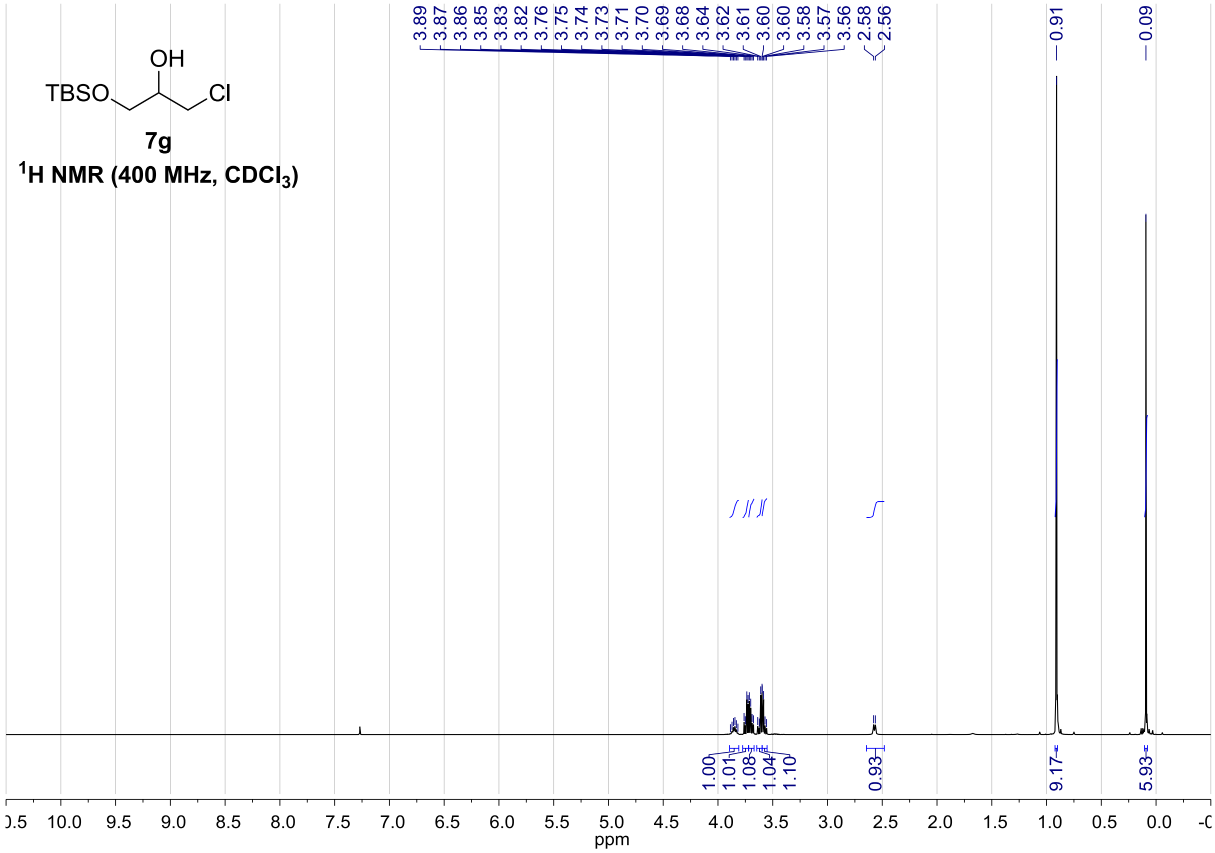

7g

^{13}C NMR (101 MHz, $CDCl_3$)

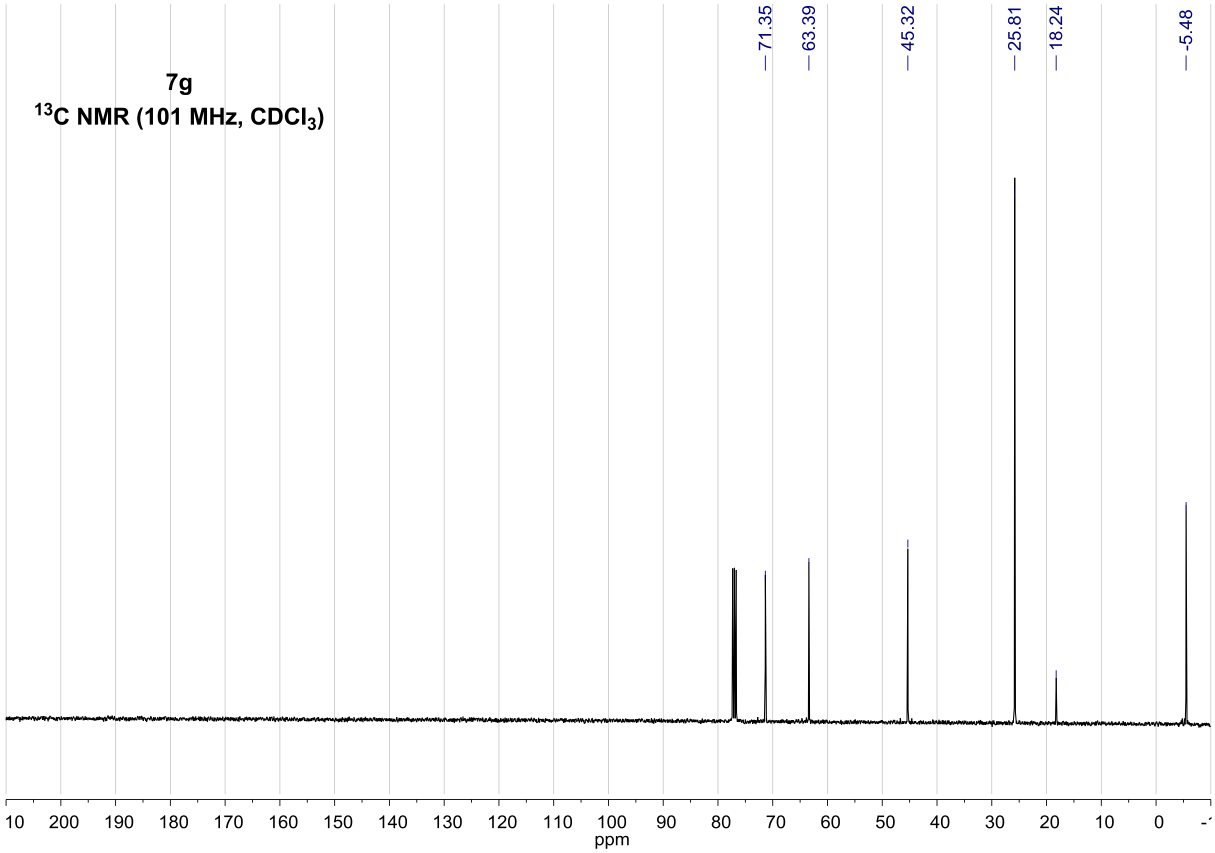

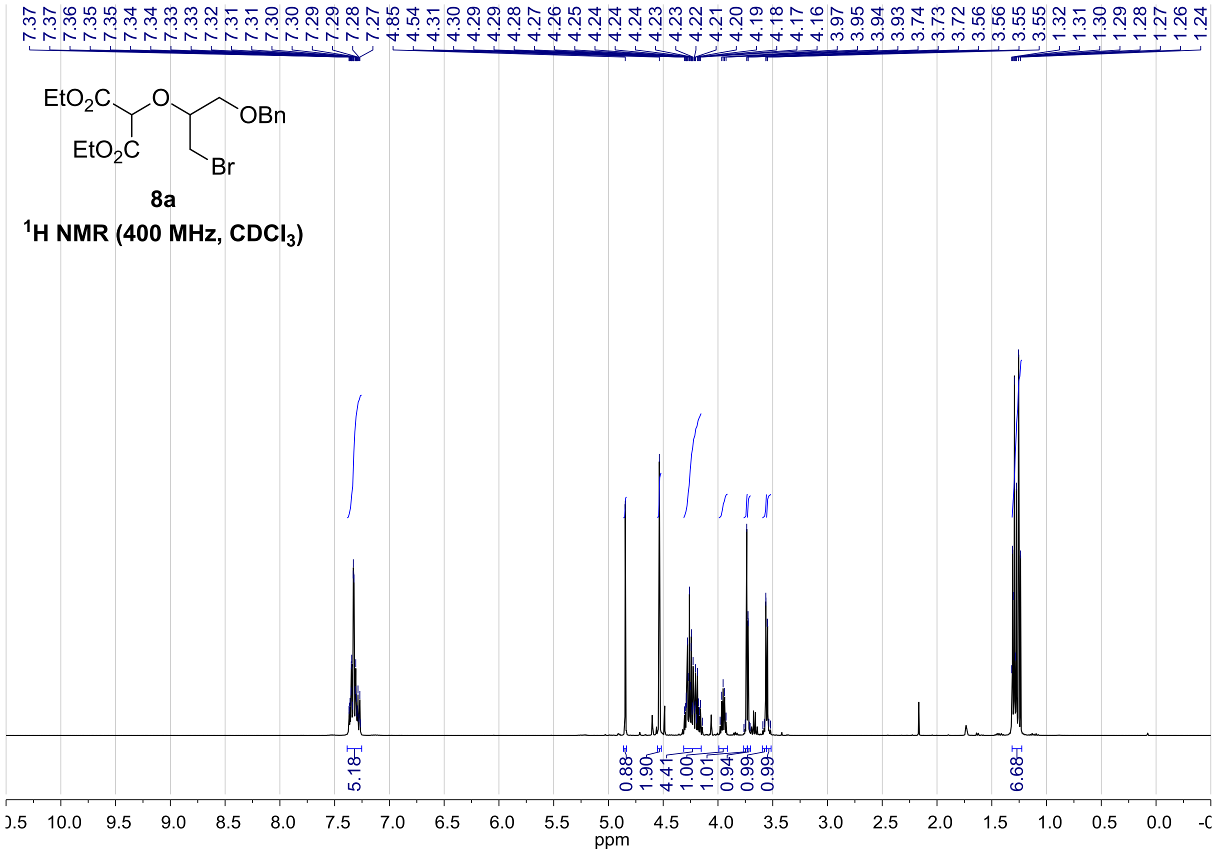
7.37 7.37 7.36 7.35 7.35 7.34 7.34 7.33 7.33 7.32 7.31 7.31 7.30 7.30 7.29 7.29 7.28 7.27 4.85 4.54 4.31 4.30 4.29 4.29 4.28 4.27 4.26 4.25 4.24 4.24 4.24 4.23 4.23 4.22 4.21 4.20 4.19 4.18 4.17 4.16 3.97 3.95 3.94 3.93 3.74 3.73 3.72 3.56 3.55 3.55 1.32 1.31 1.30 1.29 1.28 1.27 1.26 1.24
EtO2C
O
OBn
EtO2C
Br
8a
1H NMR (400 MHz, CDCl3)
5.18
0.88
1.90
4.41
1.00
1.01
0.94
0.99
0.99
6.68
ppm

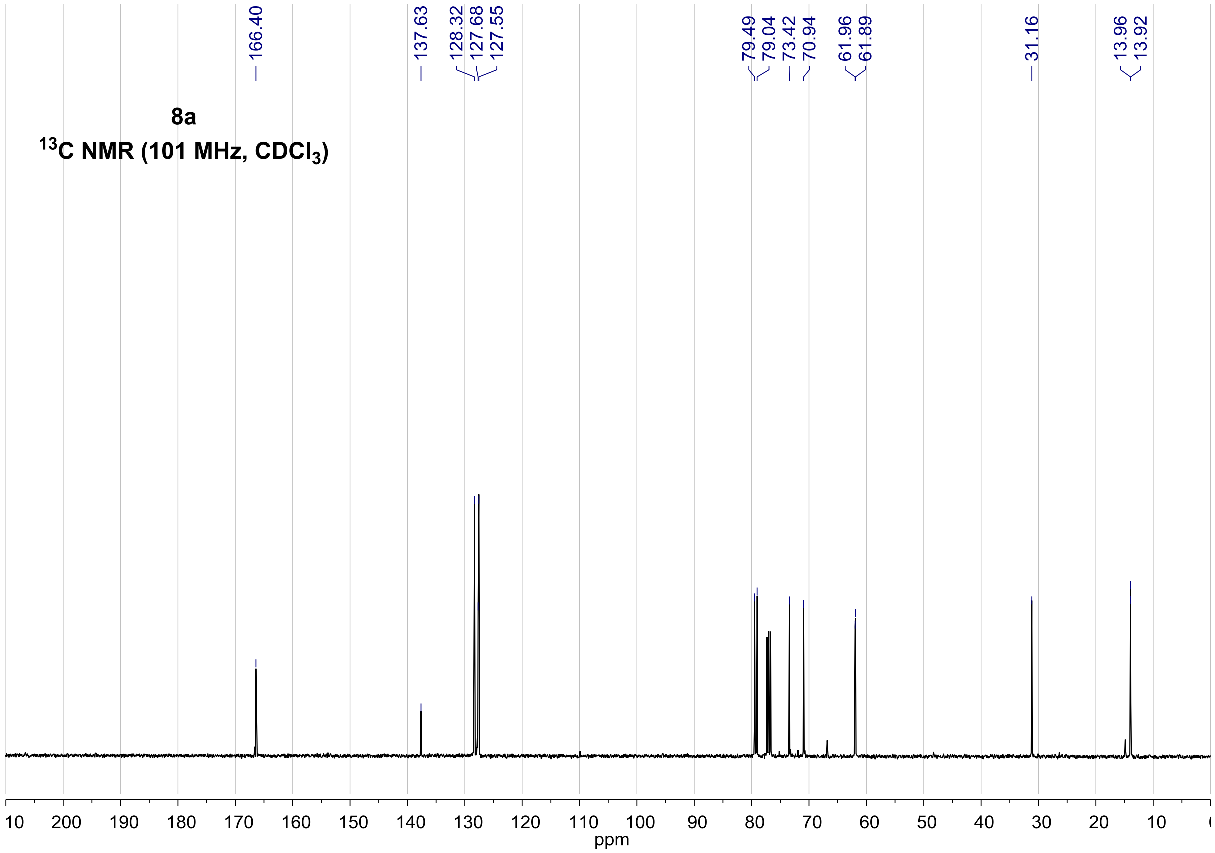
166.40
137.63
128.32
127.68
127.55
79.49
79.04
73.42
70.94
61.96
61.89
31.16
13.96
13.92
8a
13C NMR (101 MHz, CDCl3)
ppm

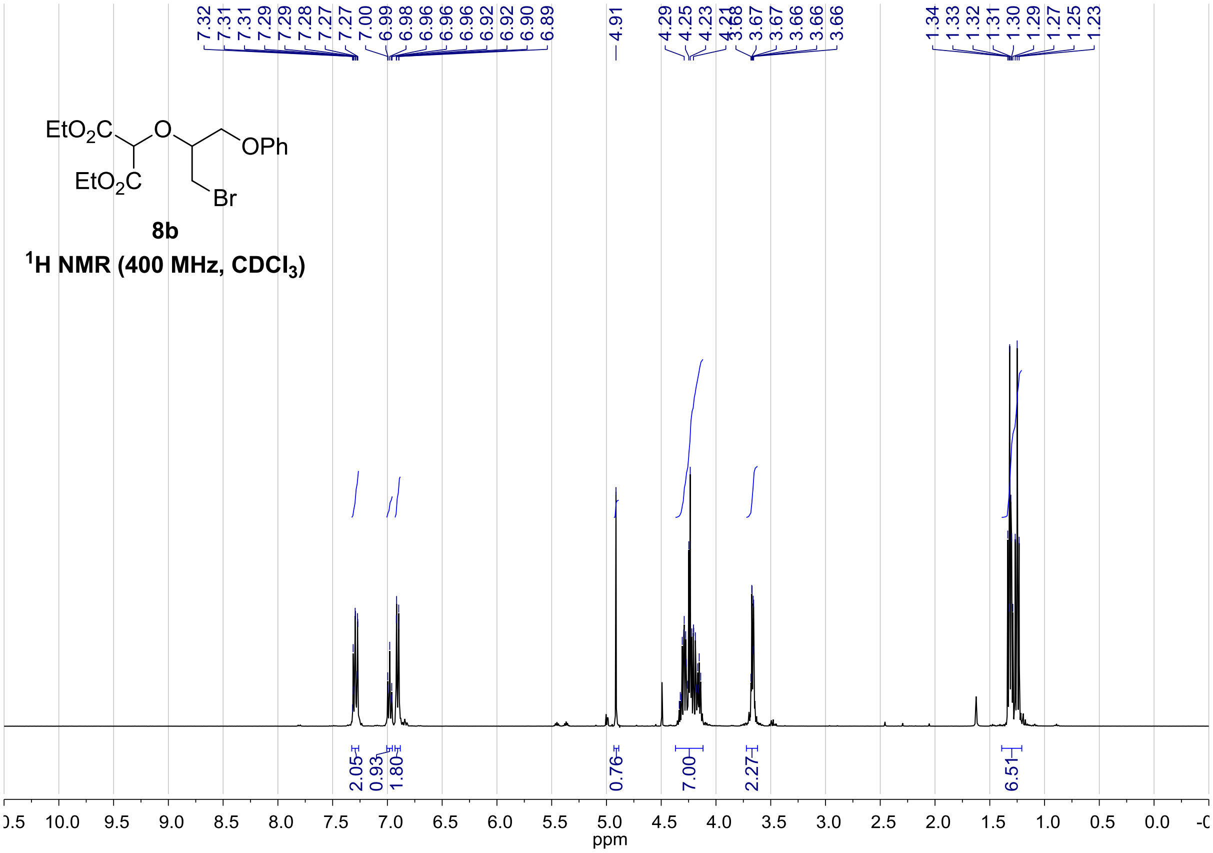
7.32 7.31 7.31 7.29 7.29 7.28 7.27 7.27 7.00 6.99 6.98 6.96 6.96 6.96 6.92 6.92 6.90 6.89
4.91
4.29 4.25 4.23 4.21 3.68 3.67 3.67 3.66 3.66 3.66
1.34 1.33 1.32 1.31 1.30 1.29 1.27 1.25 1.23
EtO2C
O
OPh
EtO2C
Br
8b
1H NMR (400 MHz, CDCl3)
2.05
0.93
1.80
0.76
7.00
2.27
6.51
ppm

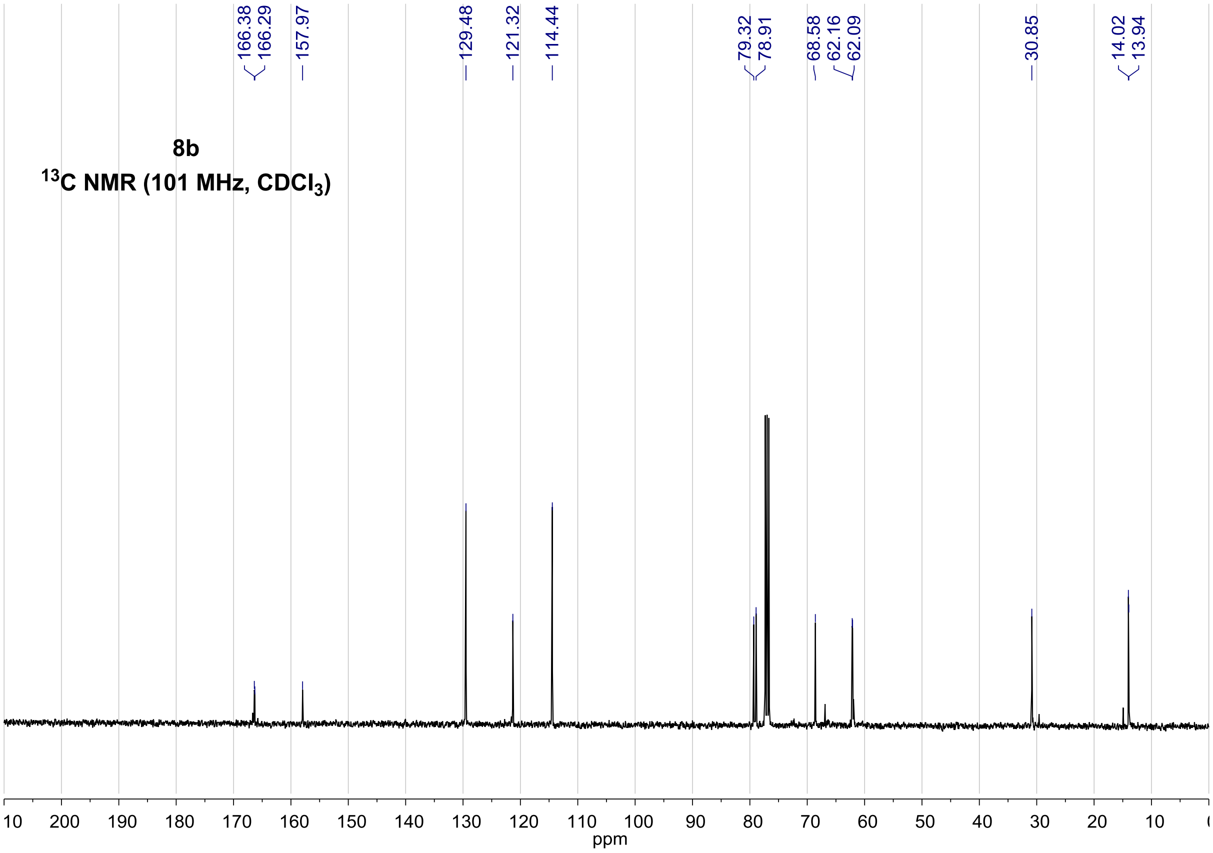
166.38 166.29
157.97
129.48
121.32
114.44
79.32 78.91
68.58
62.16 62.09
30.85
14.02 13.94
8b
13C NMR (101 MHz, CDCl3)
ppm

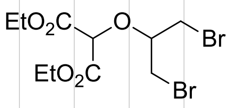

8c

^{1}H NMR (400 MHz, $CDCl_3$)

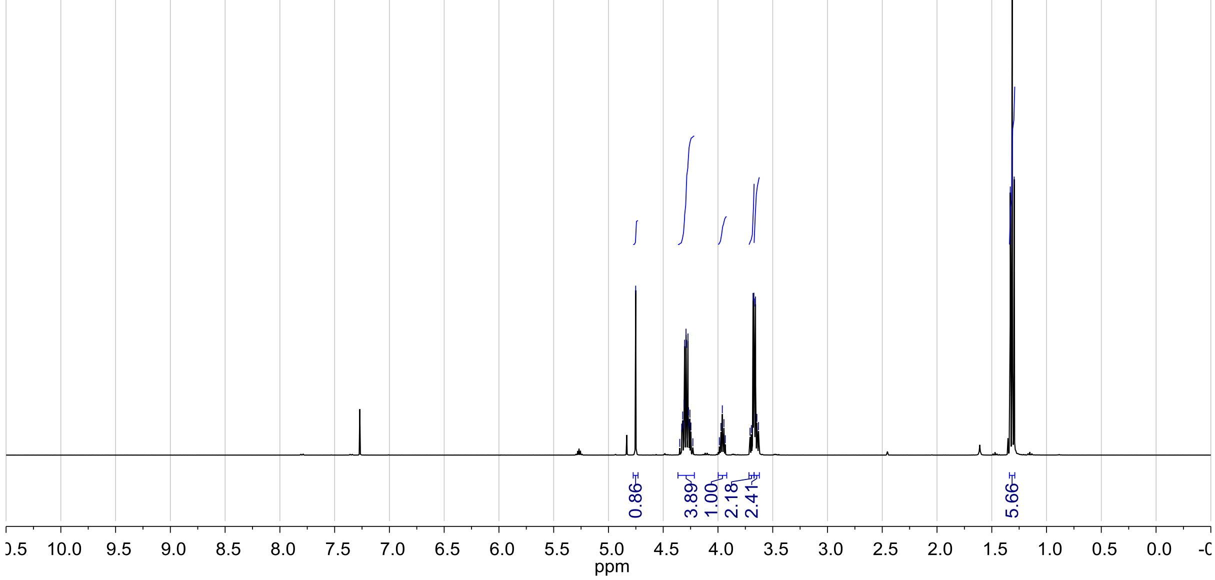

8c

^{13}C NMR (101 MHz, $CDCl_3$)

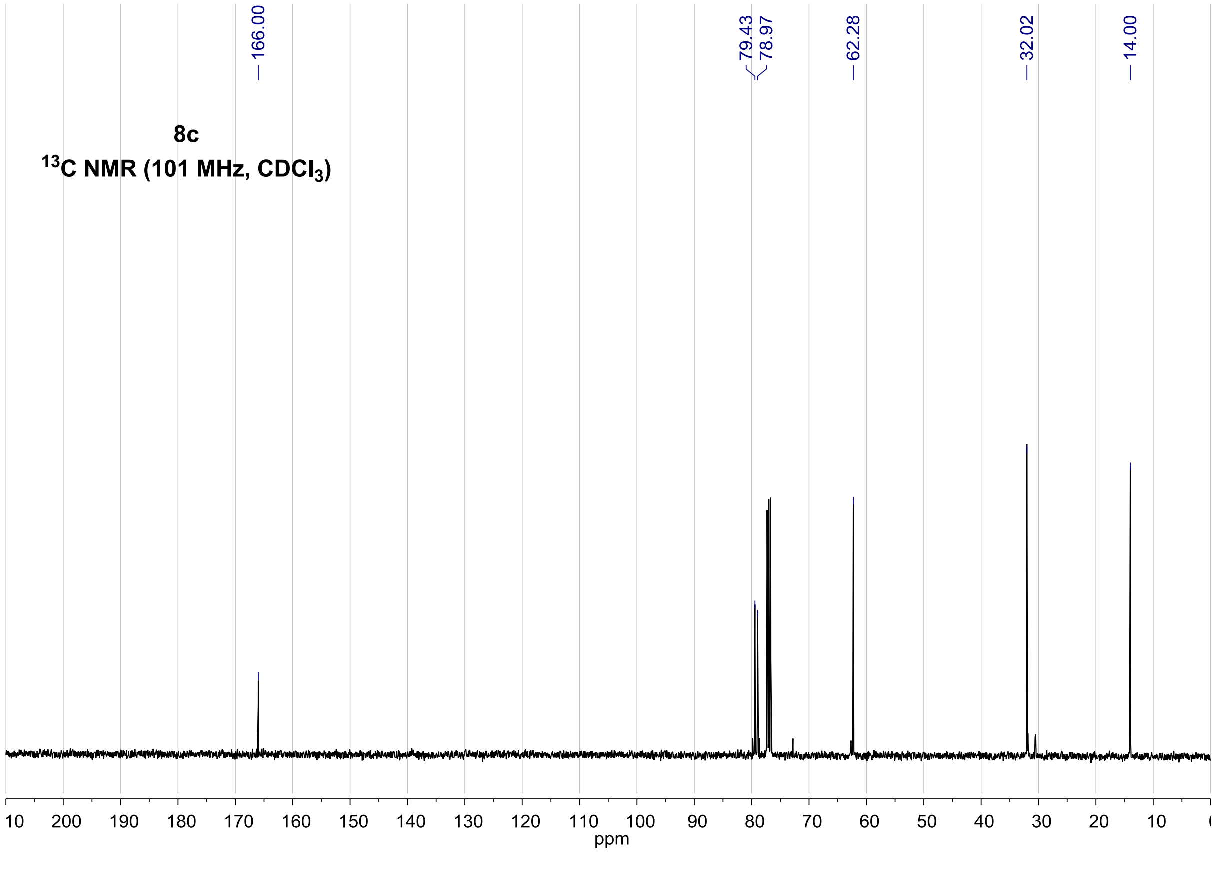

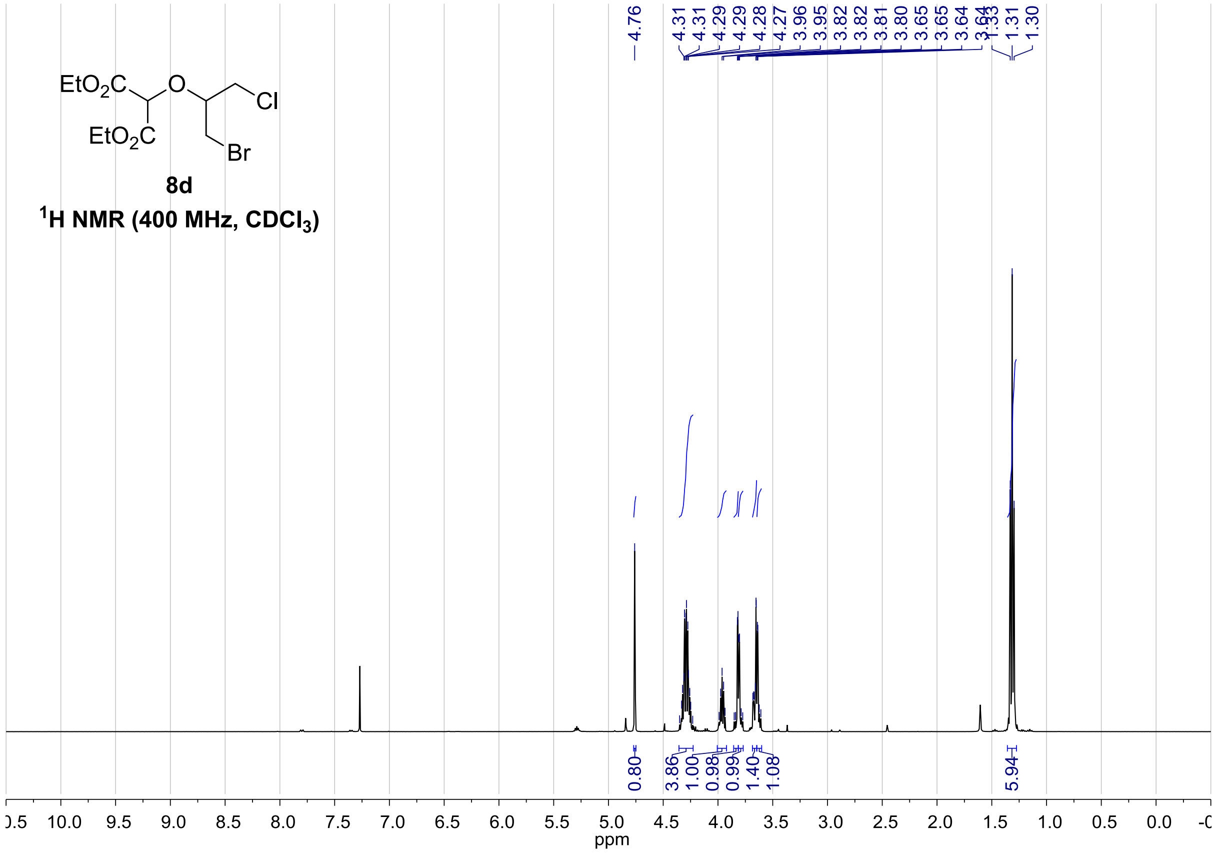
EtO2C
O
Cl
EtO2C
Br
8d
1H NMR (400 MHz, CDCl3)
4.76
4.31 4.31 4.29 4.29 4.28 4.27 3.96 3.95 3.82 3.82 3.81 3.80 3.65 3.64 3.64 1.33 1.31 1.30
0.80
3.86 1.00 0.98 0.99 1.40 1.08
5.94
ppm

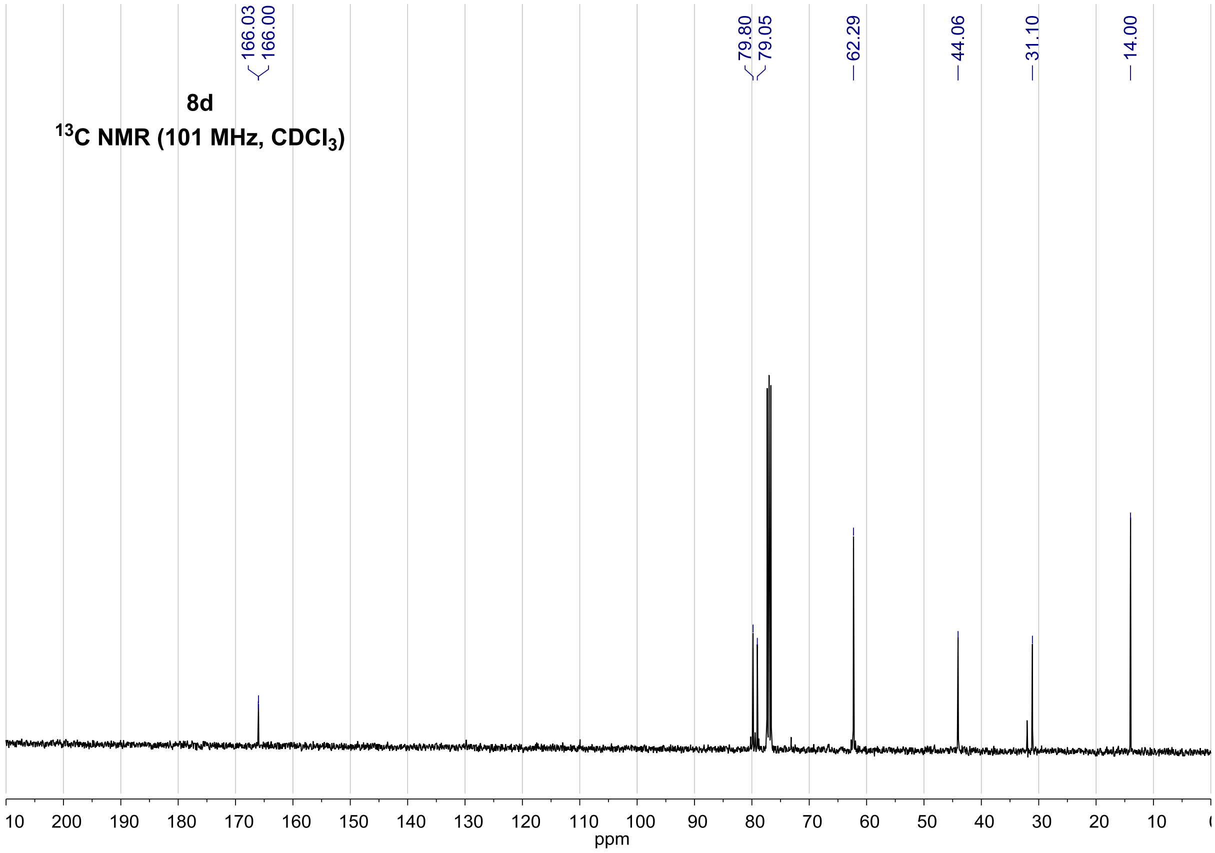
166.03
166.00
79.80
79.05
62.29
44.06
31.10
14.00
8d
13C NMR (101 MHz, CDCl3)
ppm

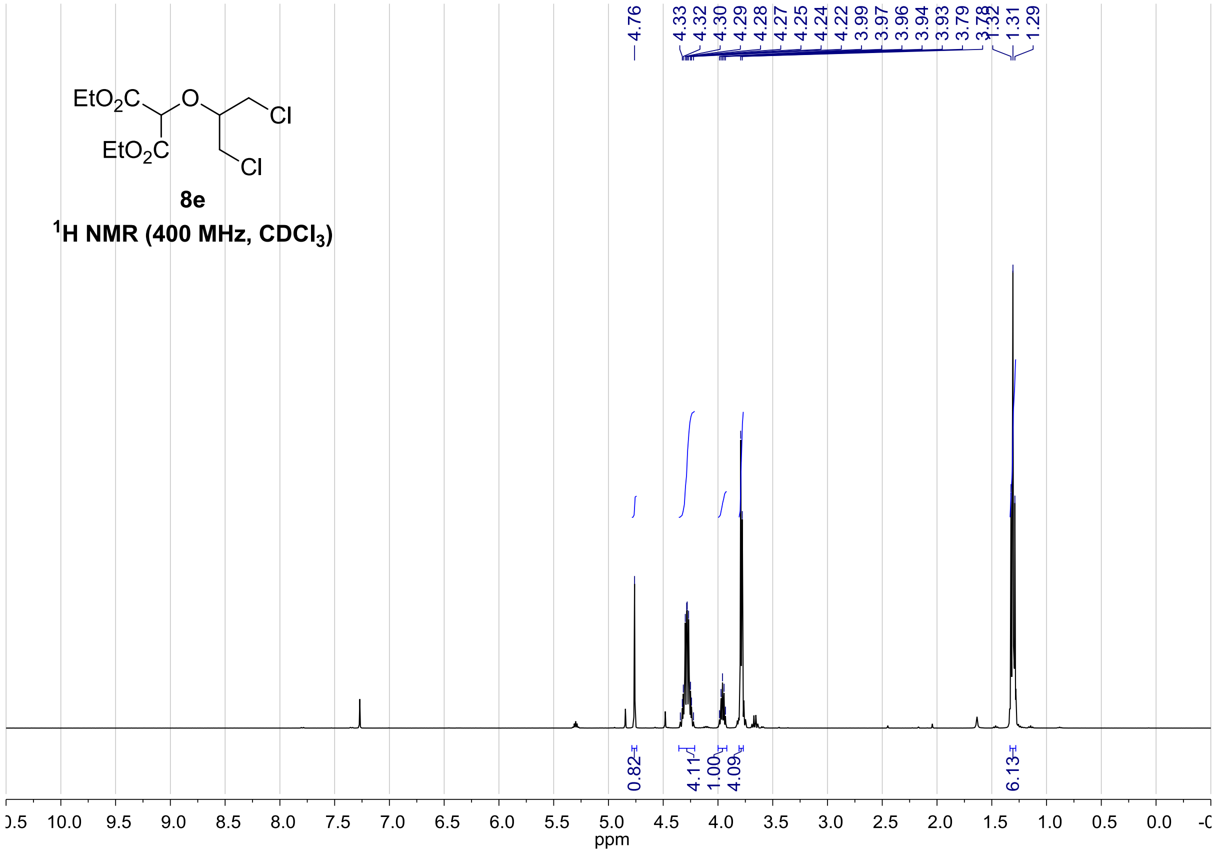
4.76
4.33 4.32 4.30 4.29 4.28 4.27 4.25 4.24 4.22 3.99 3.97 3.96 3.94 3.93 3.79 3.78 1.32 1.31 1.29
EtO2C
O
Cl
EtO2C
Cl
8e
1H NMR (400 MHz, CDCl3)
0.82 4.11 1.00 4.09 6.13
ppm

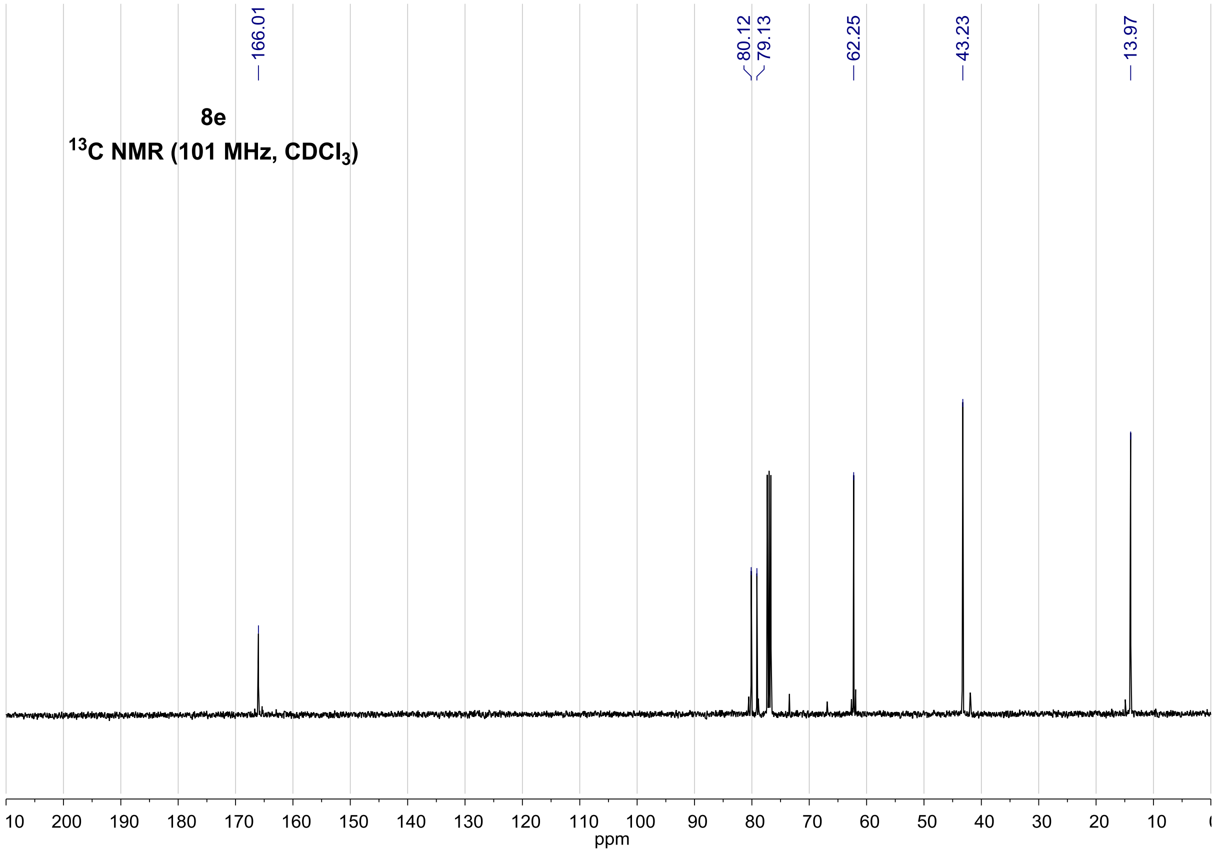
166.01
80.12 79.13
62.25
43.23
13.97
8e
13C NMR (101 MHz, CDCl3)
ppm

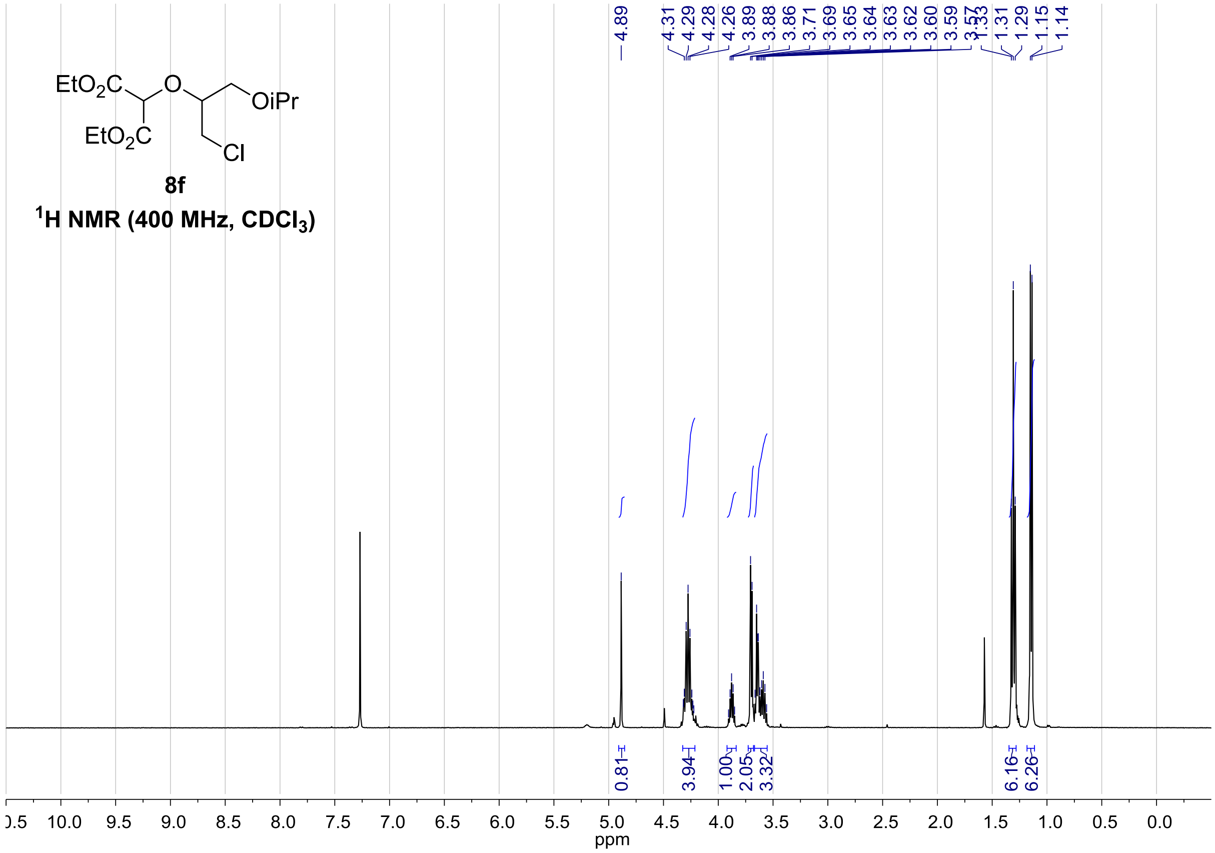
EtO2C
O
OiPr
EtO2C
Cl
8f
1H NMR (400 MHz, CDCl3)
4.89
4.31
4.29
4.28
4.26
3.89
3.88
3.86
3.71
3.69
3.65
3.64
3.63
3.62
3.60
3.59
3.57
1.33
1.31
1.29
1.15
1.14
0.81
3.94
1.00
2.05
3.32
6.16
6.26
ppm

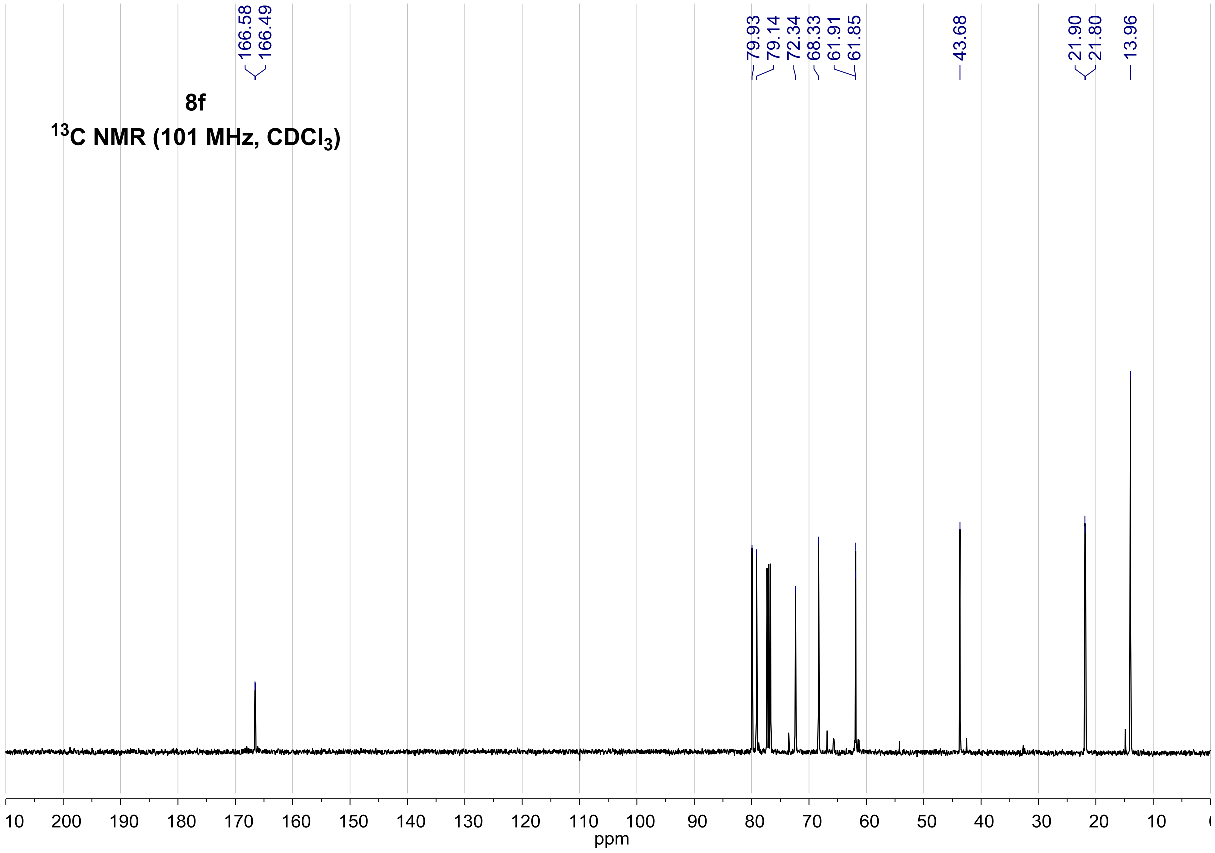
8f
13C NMR (101 MHz, CDCl3)
166.58
166.49
79.93
79.14
72.34
68.33
61.91
61.85
43.68
21.90
21.80
13.96
ppm

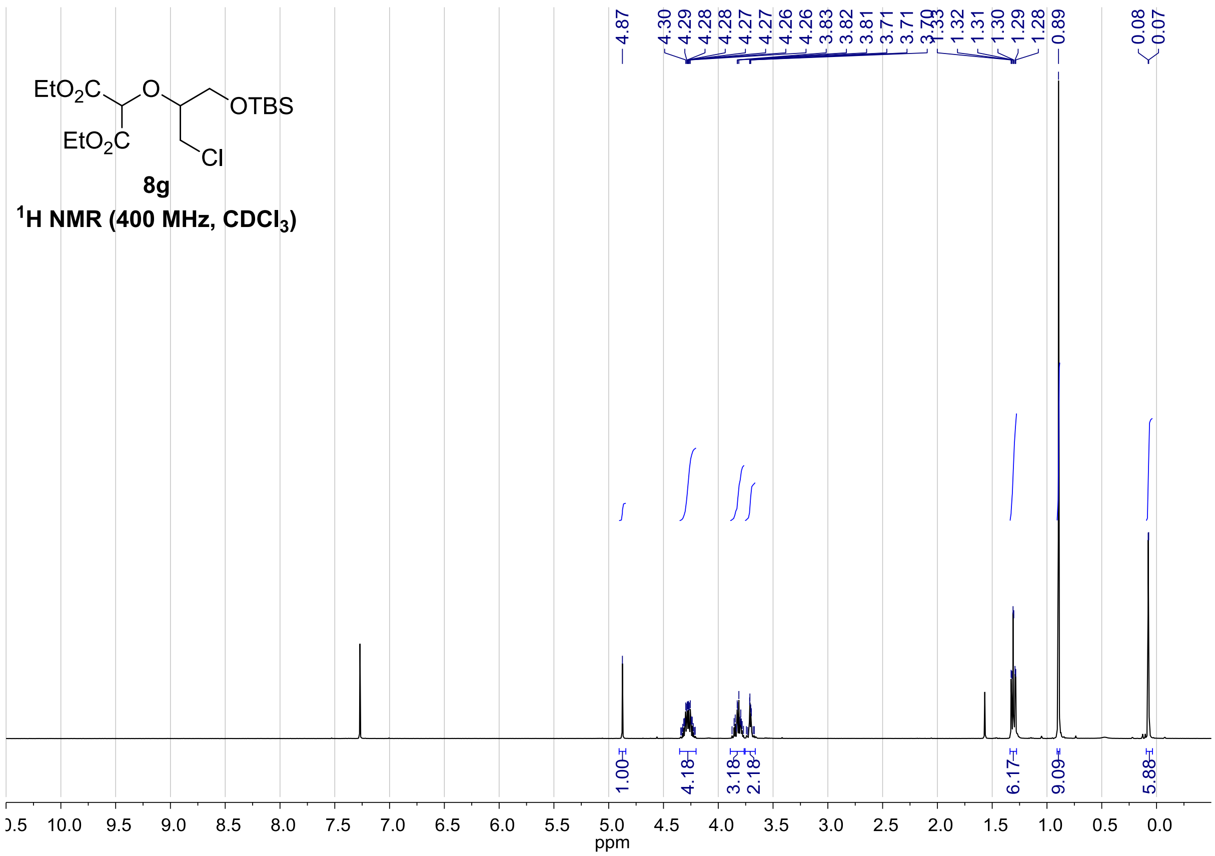
EtO2C
O
OTBS
EtO2C
Cl
8g
1H NMR (400 MHz, CDCl3)
4.87
4.30
4.29
4.28
4.28
4.27
4.27
4.26
4.26
3.83
3.82
3.81
3.71
3.71
3.70
1.33
1.32
1.31
1.30
1.29
1.28
0.89
0.08
0.07
1.00
4.18
3.18
2.18
6.17
9.09
5.88
ppm

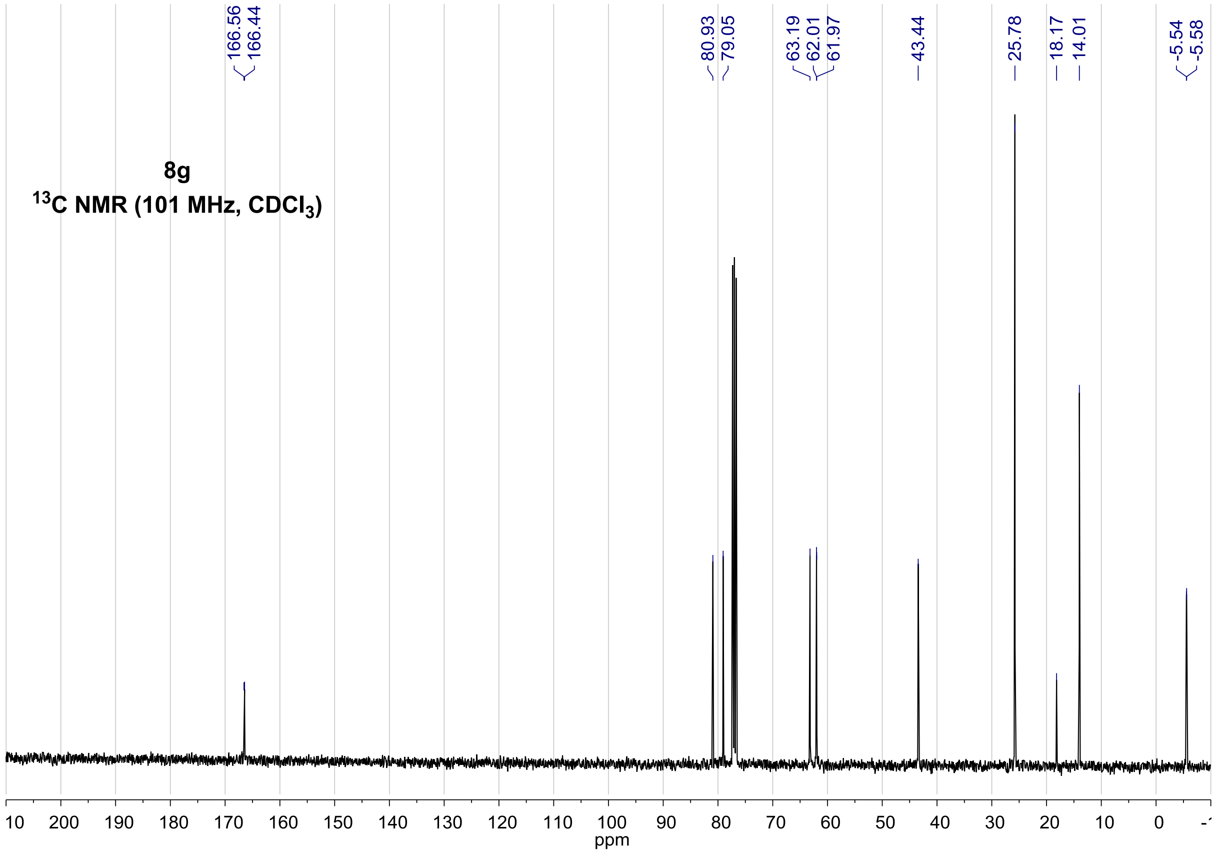
166.56
166.44
80.93
79.05
63.19
62.01
61.97
43.44
25.78
18.17
14.01
-5.54
-5.58
8g
13C NMR (101 MHz, CDCl3)
ppm

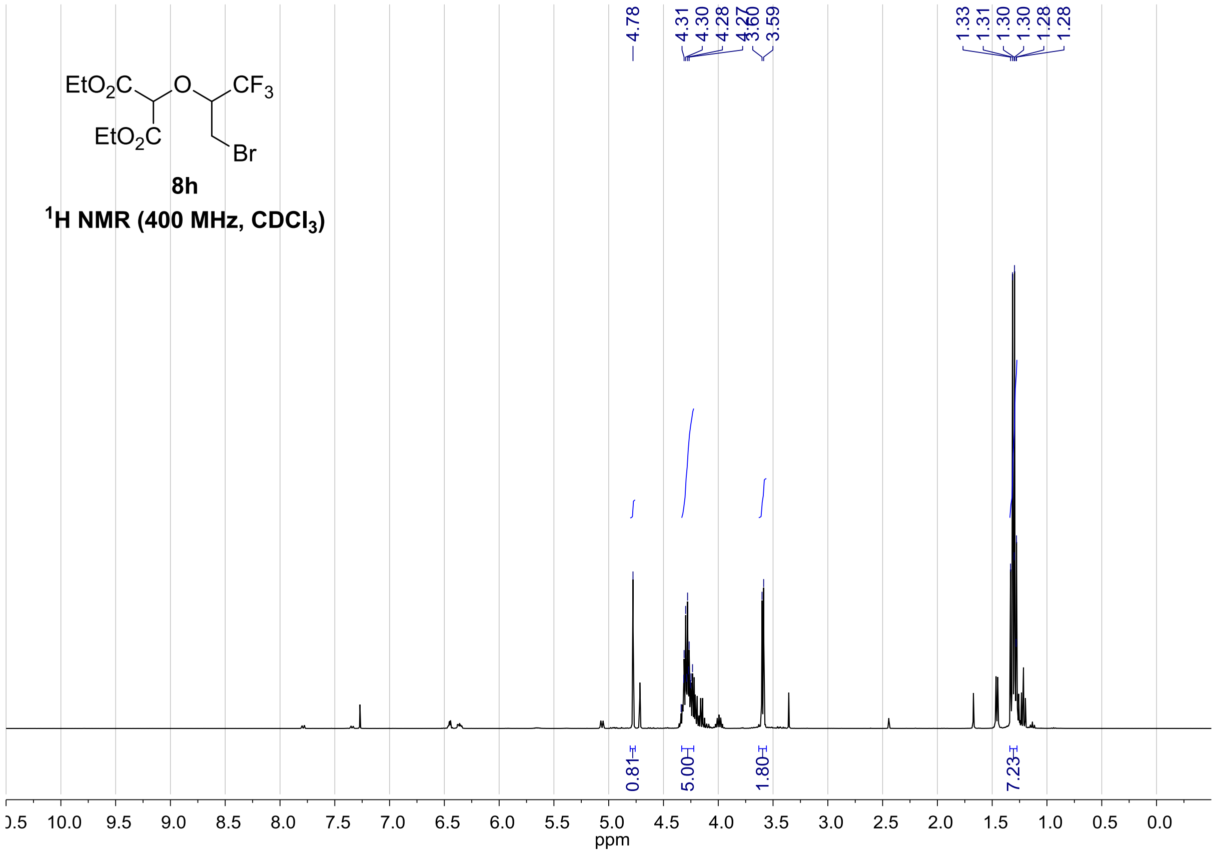
EtO2C O CF3
EtO2C Br
8h
1H NMR (400 MHz, CDCl3)
4.78
4.31 4.30 4.28 4.27 3.60 3.59
1.33 1.31 1.30 1.30 1.28 1.28
0.81 5.00 1.80 7.23
0.5 10.0 9.5 9.0 8.5 8.0 7.5 7.0 6.5 6.0 5.5 5.0 4.5 4.0 3.5 3.0 2.5 2.0 1.5 1.0 0.5 0.0
ppm

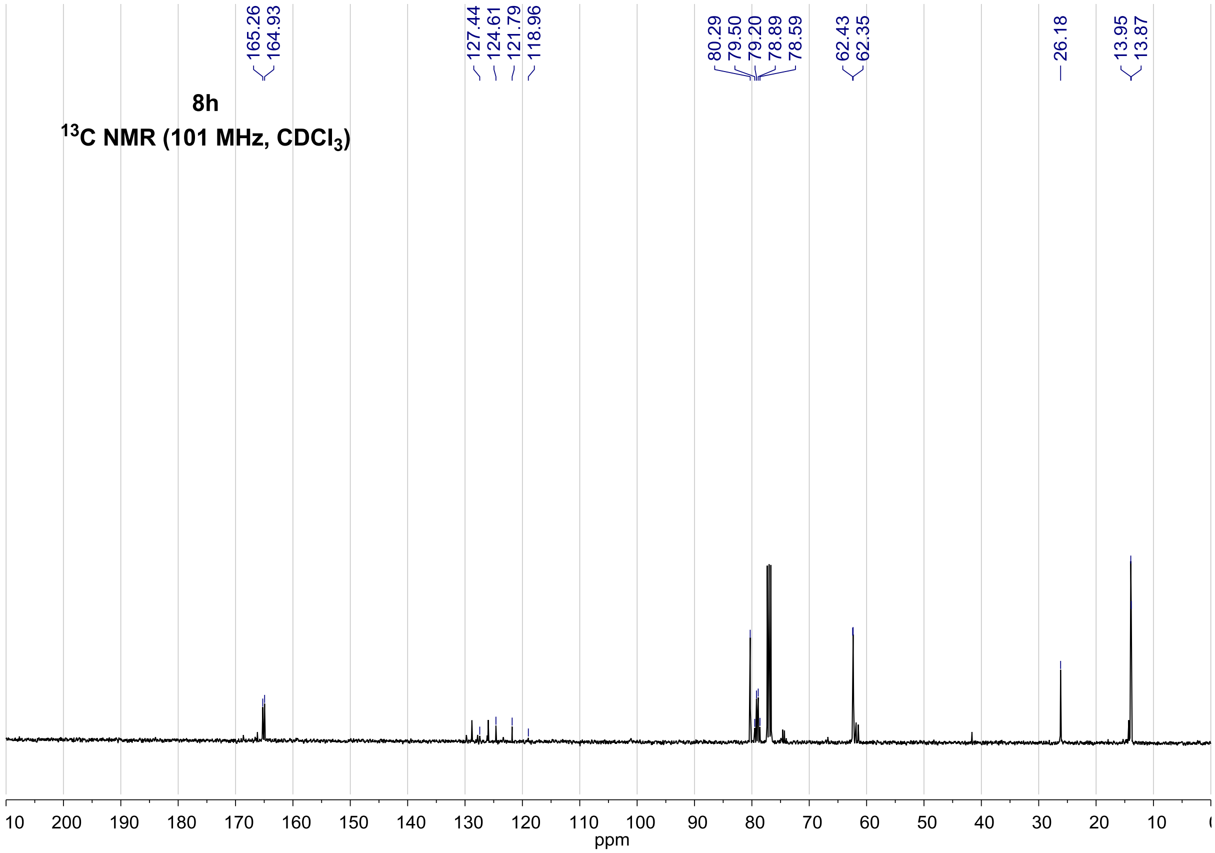
8h
13C NMR (101 MHz, CDCl3)
165.26 164.93
127.44 124.61 121.79 118.96
80.29 79.50 79.20 78.89 78.59
62.43 62.35
26.18
13.95 13.87
10 200 190 180 170 160 150 140 130 120 110 100 90 80 70 60 50 40 30 20 10
ppm

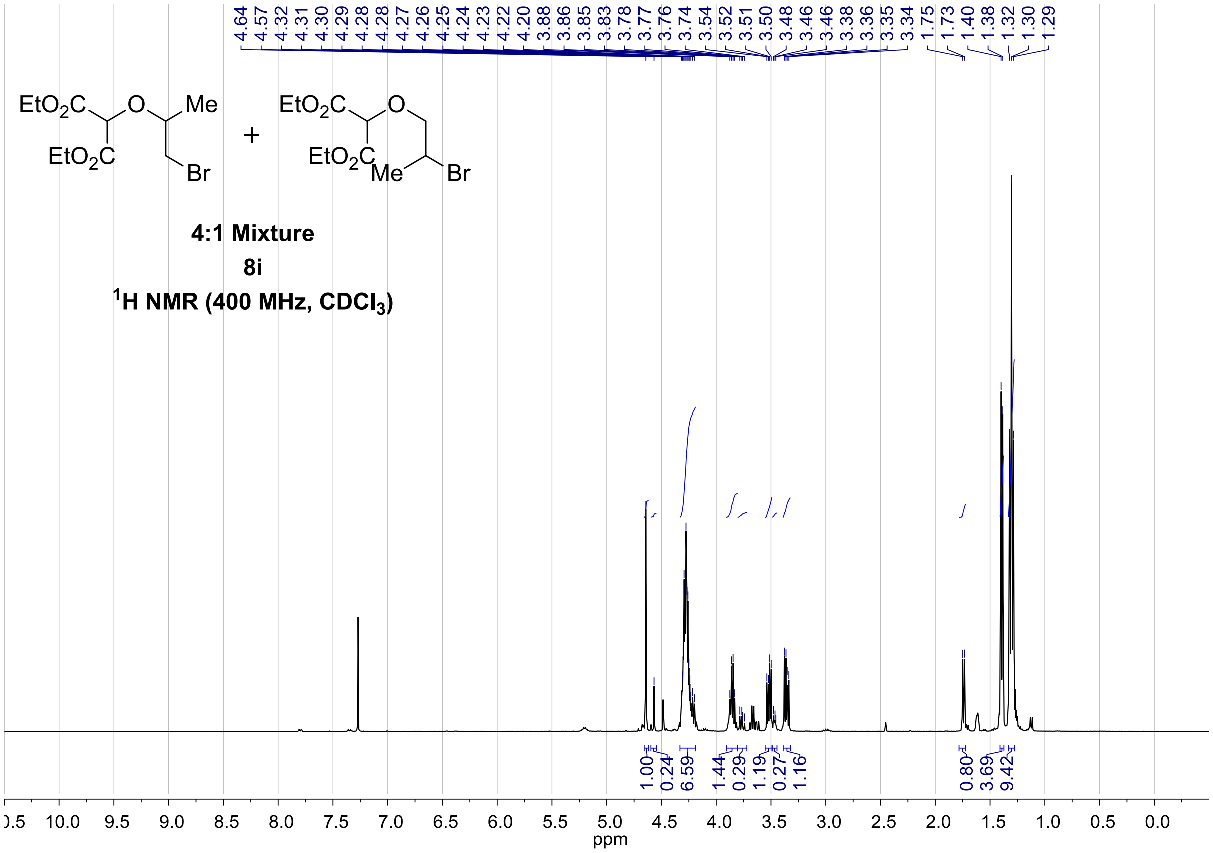
4.64
4.57
4.32
4.31
4.30
4.29
4.28
4.28
4.27
4.26
4.25
4.24
4.23
4.22
4.20
3.88
3.86
3.85
3.83
3.78
3.77
3.76
3.74
3.54
3.52
3.51
3.50
3.48
3.46
3.46
3.38
3.36
3.35
3.34
1.75
1.73
1.40
1.38
1.32
1.30
1.29
EtO_2C
EtO_2C
O
Me
Br
+
EtO_2C
EtO_2C
O
Me
Br
4:1 Mixture
8i
^{1}H NMR (400 MHz, $CDCl_3$)
1.00
0.24
6.59
1.44
0.29
1.19
0.27
1.16
0.80
3.69
9.42
ppm

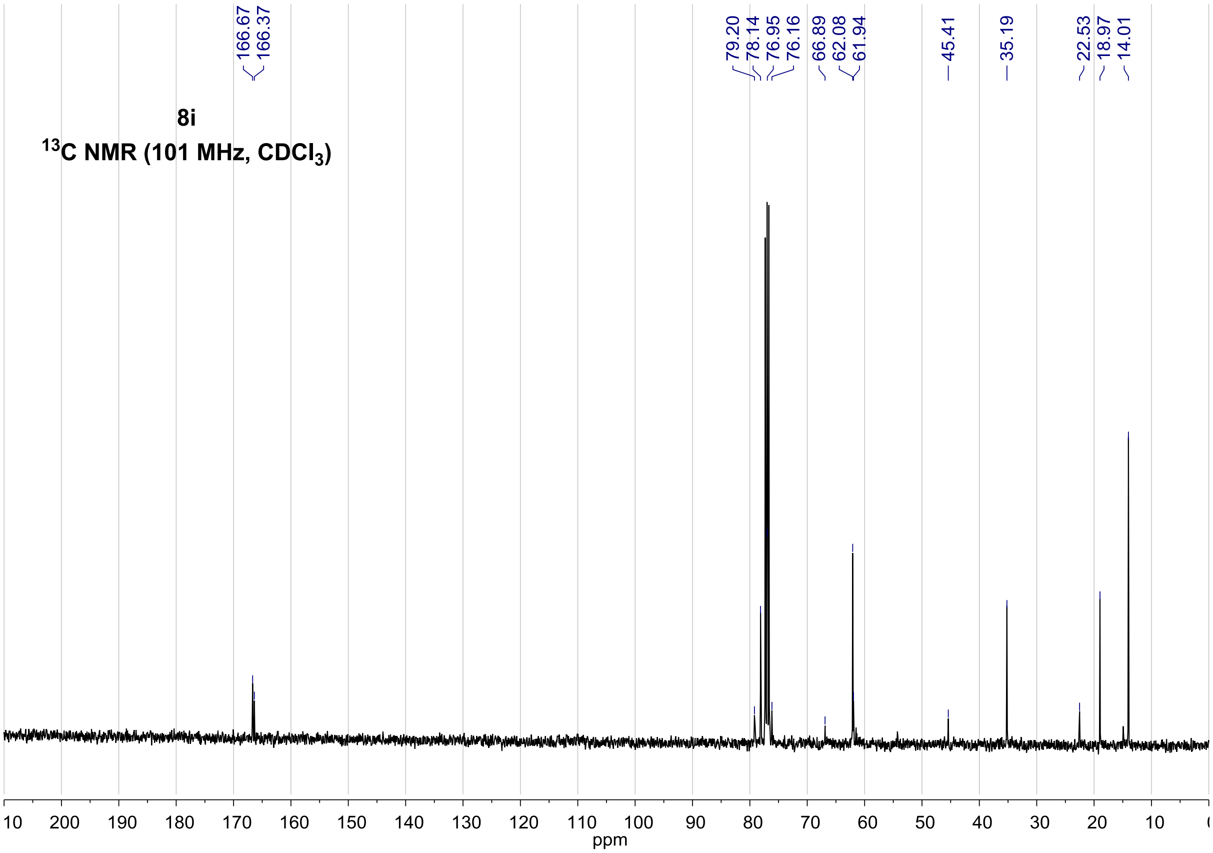
166.67
166.37
79.20
78.14
76.95
76.16
66.89
62.08
61.94
45.41
35.19
22.53
18.97
14.01
8i
^{13}C NMR (101 MHz, $CDCl_3$)
ppm

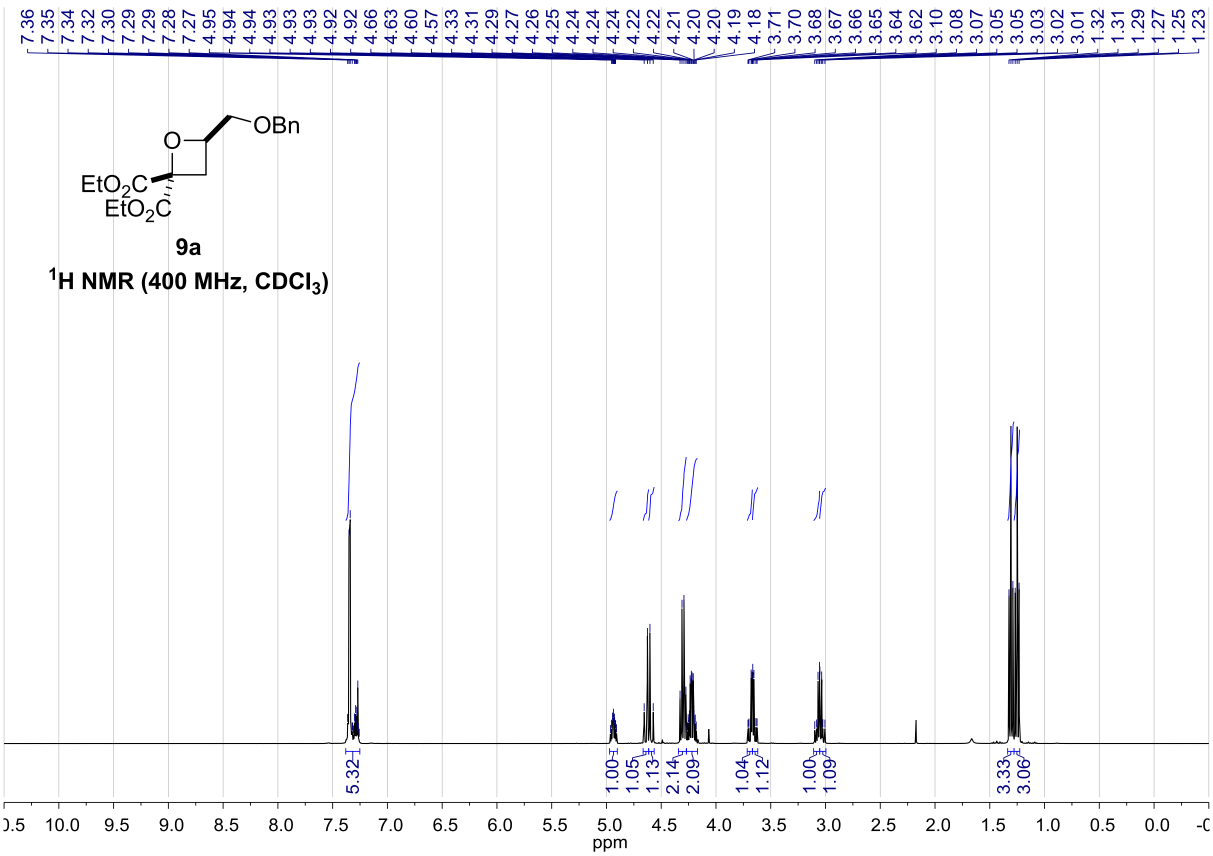
7.36 7.35 7.34 7.32 7.30 7.29 7.29 7.28 7.27 4.95 4.94 4.94 4.93 4.93 4.93 4.92 4.92 4.66 4.63 4.60 4.57 4.33 4.31 4.29 4.27 4.26 4.25 4.24 4.24 4.24 4.24 4.22 4.22 4.21 4.20 4.20 4.19 4.18 3.71 3.70 3.68 3.67 3.66 3.65 3.64 3.62 3.10 3.08 3.07 3.05 3.05 3.03 3.02 3.01 1.32 1.31 1.29 1.27 1.25 1.23
OBn
O
EtO_2C
EtO_2C
9a
^{1}H NMR (400 MHz, $CDCl_3$)
5.32 1.00 1.05 1.13 2.14 2.09 1.04 1.12 1.00 1.09 3.33 3.06
ppm

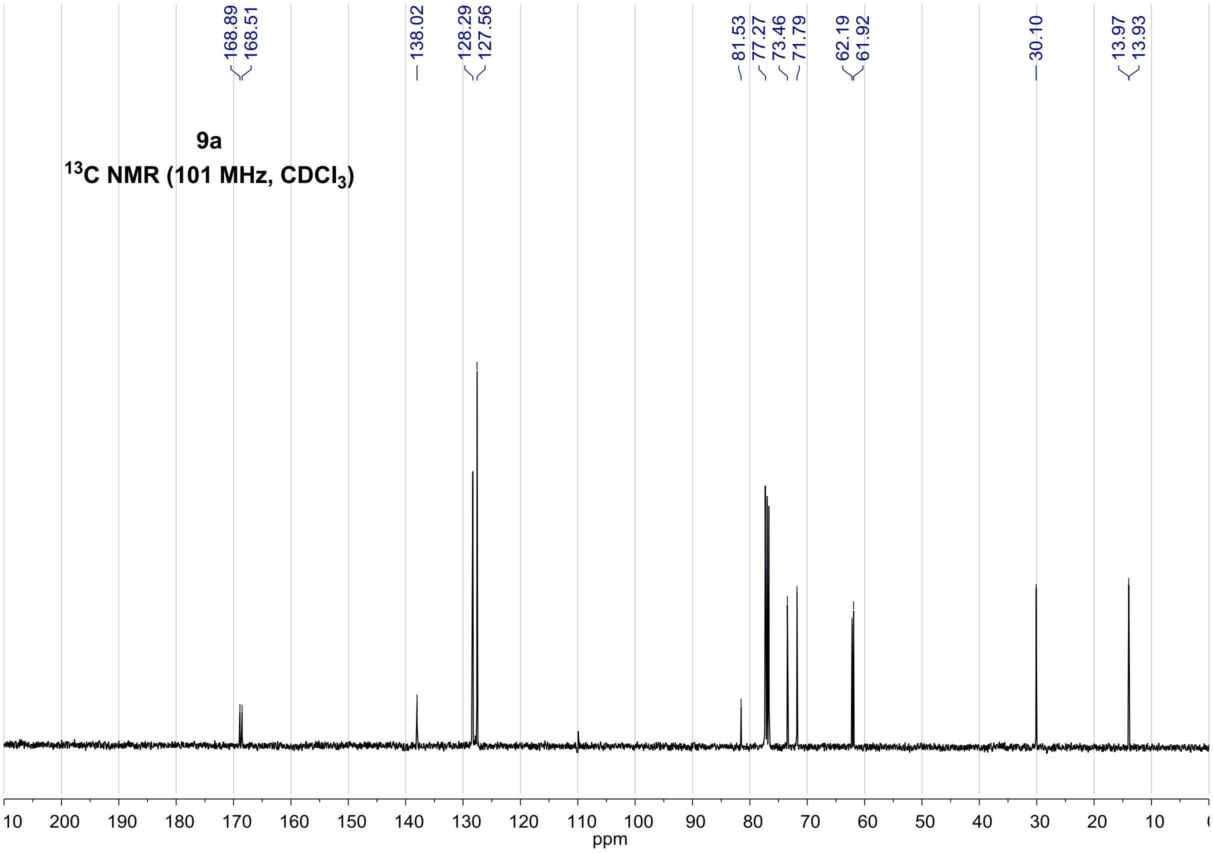
168.89 168.51 138.02 128.29 127.56 81.53 77.27 73.46 71.79 62.19 61.92 30.10 13.97 13.93
9a
^{13}C NMR (101 MHz, $CDCl_3$)
ppm

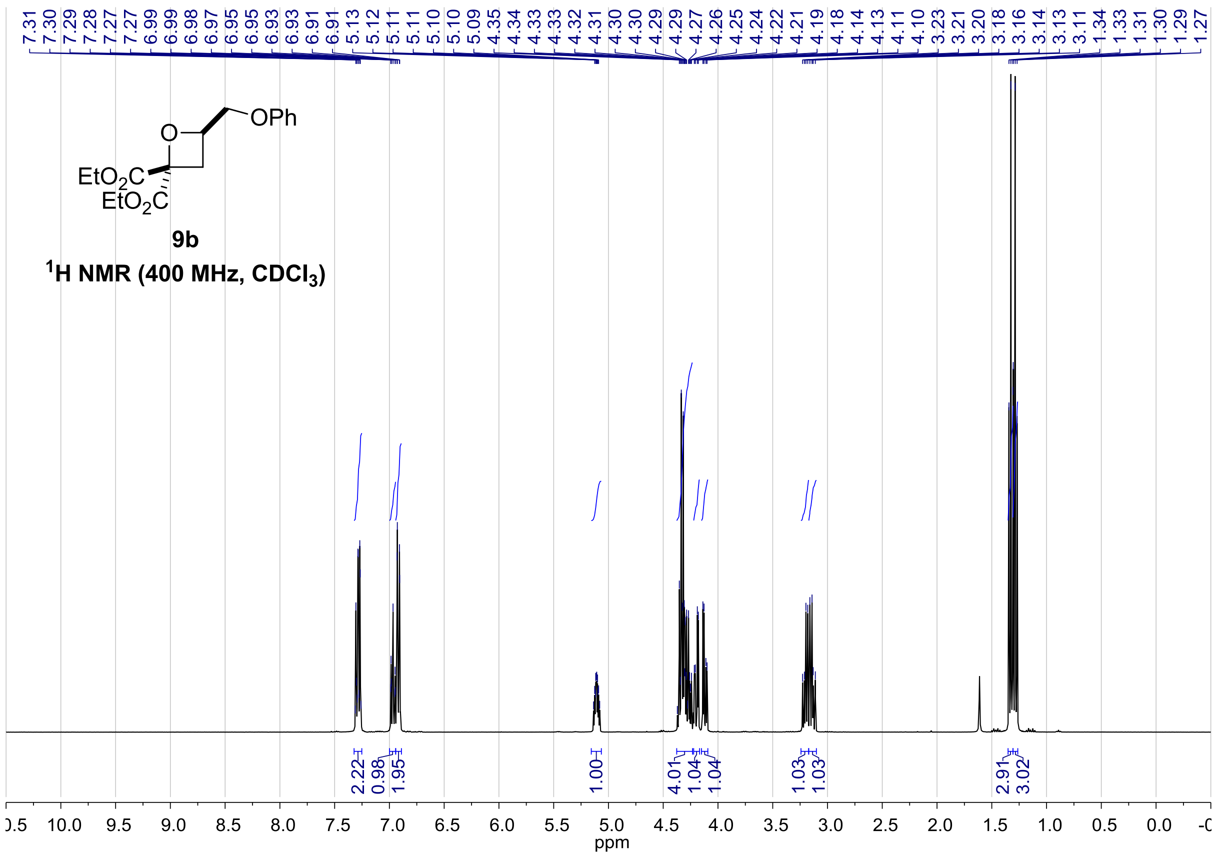

OPh
O
EtO2C
EtO2C
9b
1H NMR (400 MHz, CDCl3)
7.31 7.30 7.29 7.28 7.27 7.27 6.99 6.99 6.98 6.97 6.95 6.95 6.93 6.93 6.91 6.91 5.13 5.12 5.11 5.11 5.10 5.09 4.35 4.34 4.33 4.33 4.32 4.31 4.30 4.30 4.29 4.29 4.27 4.26 4.25 4.24 4.22 4.21 4.19 4.18 4.14 4.13 4.11 4.10 3.23 3.21 3.20 3.18 3.16 3.14 3.13 3.11 1.34 1.33 1.31 1.30 1.29 1.27
2.22 0.98 1.95 1.00 4.01 1.04 1.04 1.03 1.03 2.91 3.02
ppm

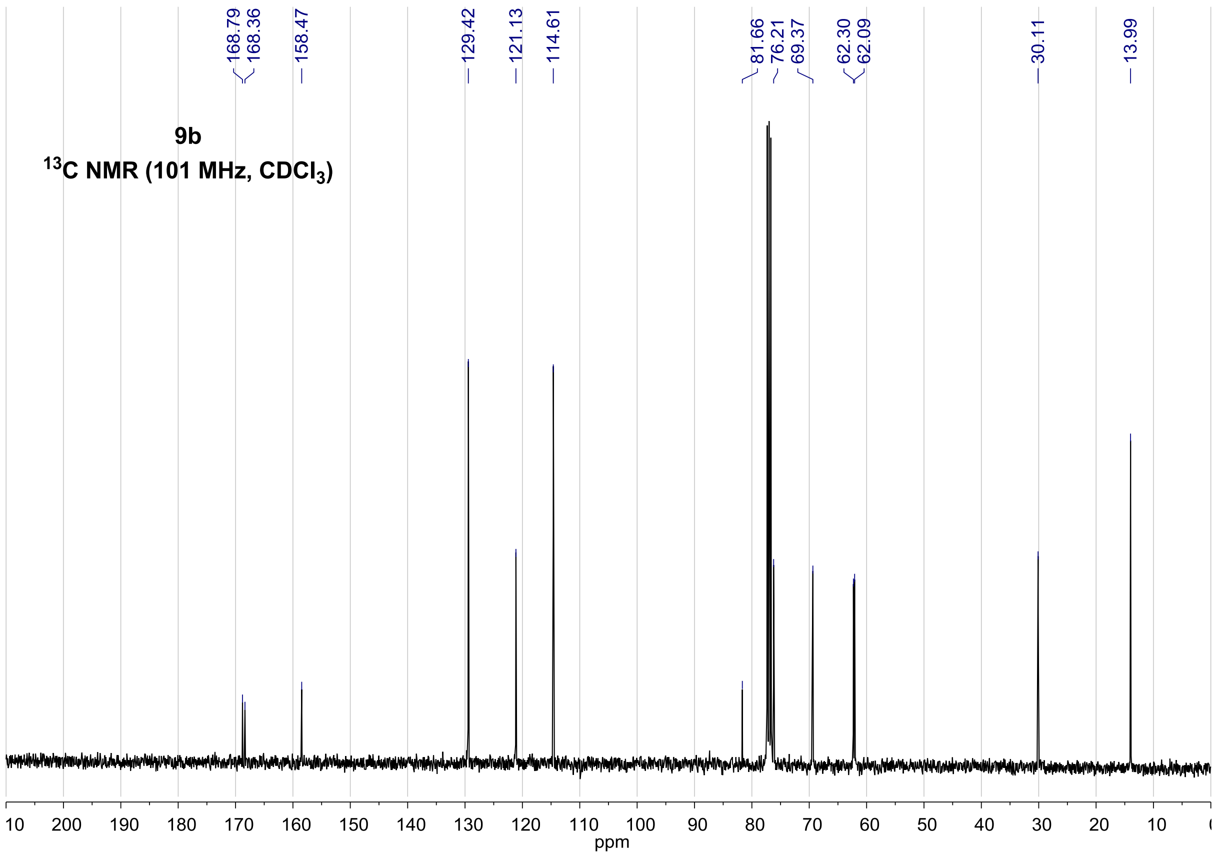

168.79 168.36 158.47 129.42 121.13 114.61 81.66 76.21 69.37 62.30 62.09 30.11 13.99
9b
13C NMR (101 MHz, CDCl3)
ppm

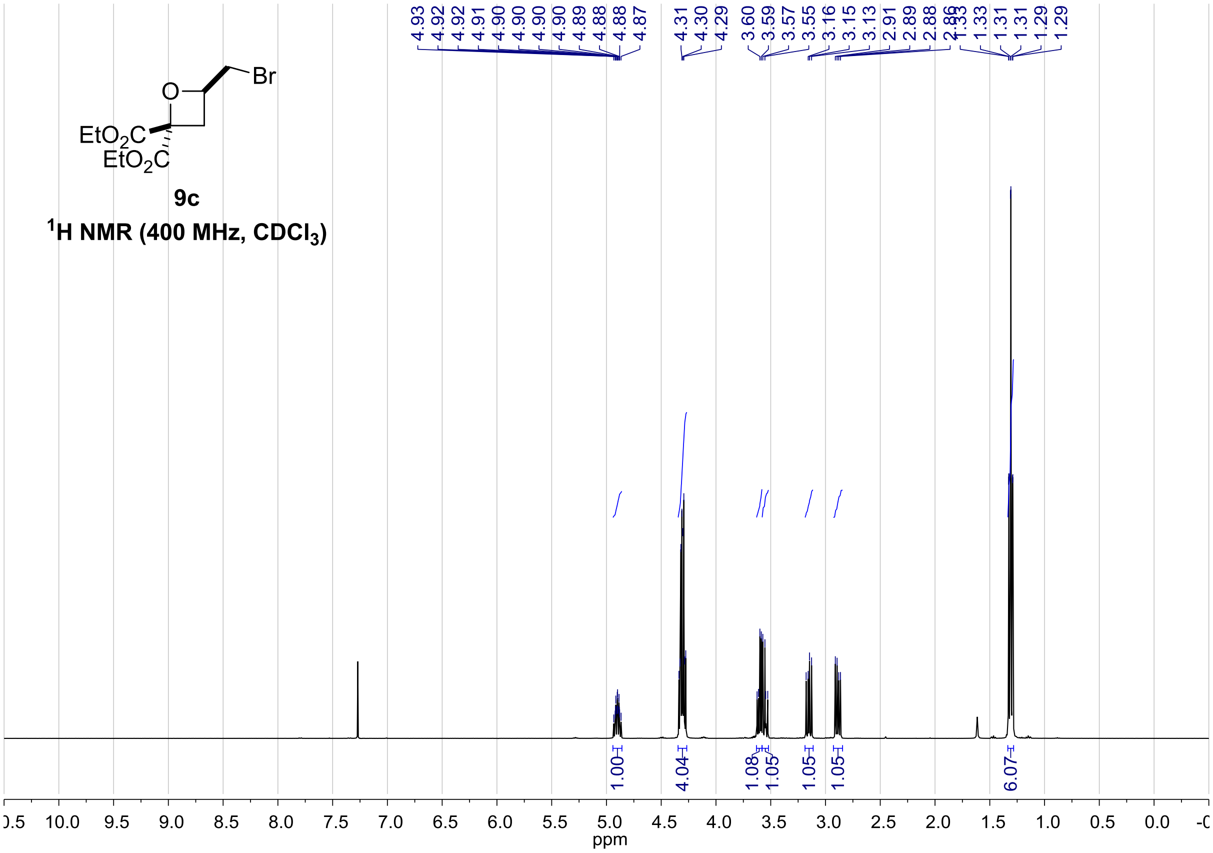
Br
O
EtO_2C
EtO_2C
9c
^{1}H NMR (400 MHz, $CDCl_3$)
4.93 4.92 4.92 4.91 4.90 4.90 4.90 4.89 4.88 4.88 4.87 4.31 4.30 4.29 3.60 3.59 3.57 3.55 3.16 3.15 3.13 2.91 2.89 2.88 2.86 1.33 1.33 1.31 1.31 1.29 1.29
1.00 4.04 1.08 1.05 1.05 1.05 6.07
ppm

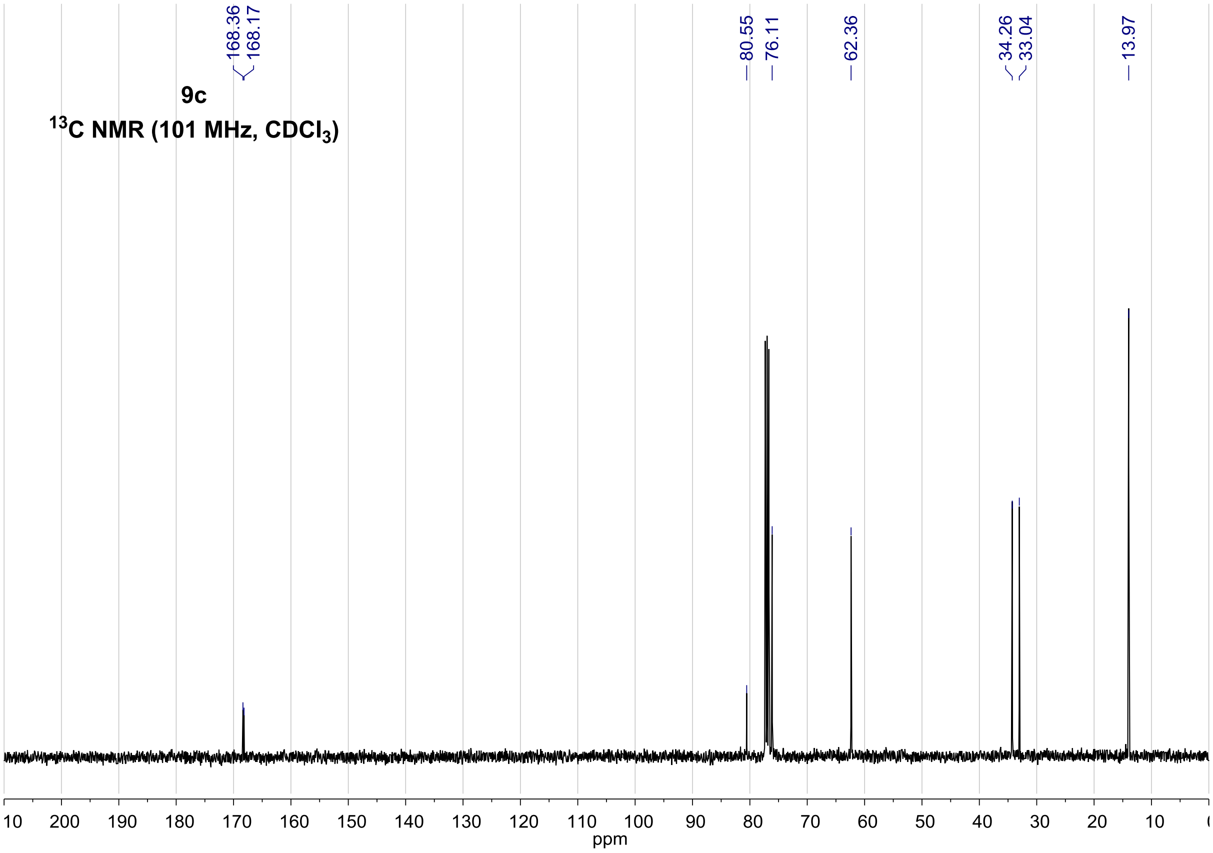
168.36 168.17 80.55 76.11 62.36 34.26 33.04 13.97
9c
^{13}C NMR (101 MHz, $CDCl_3$)
ppm

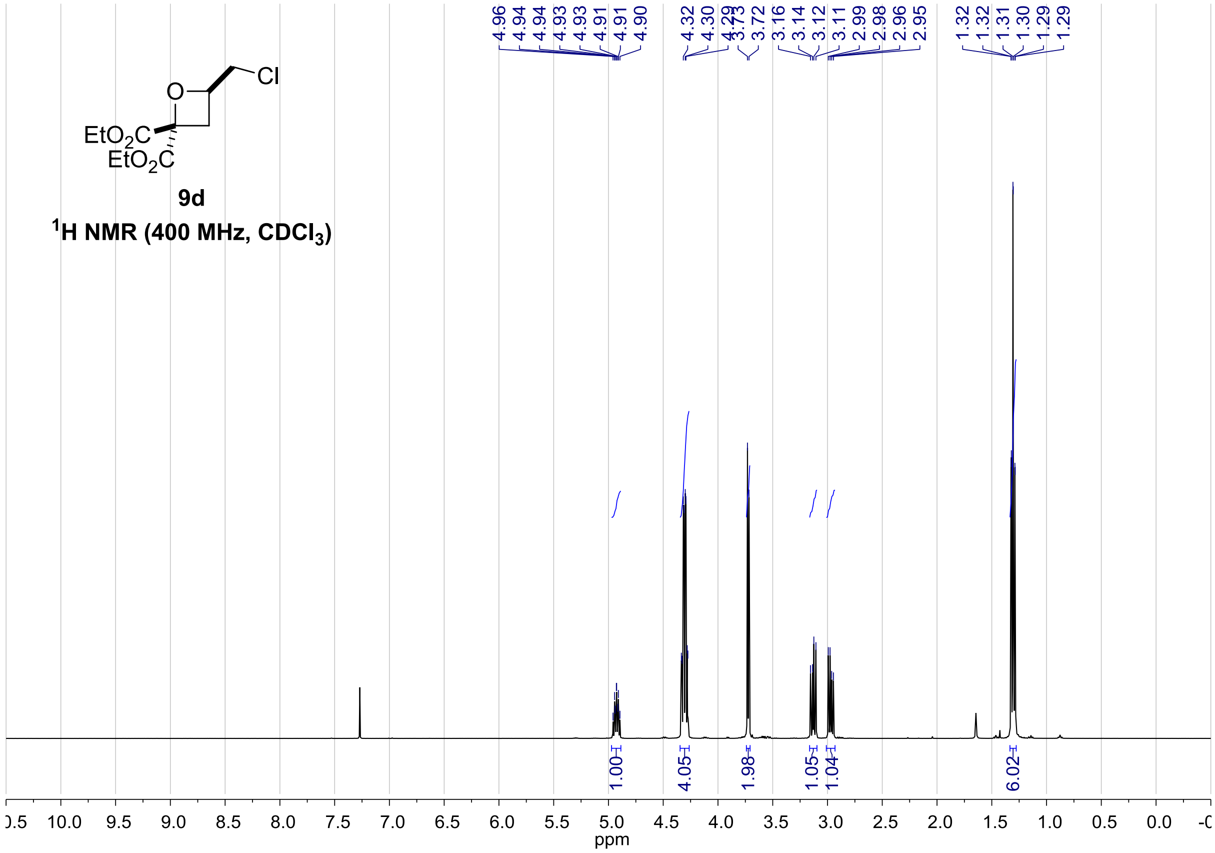
Cl
O
EtO2C
EtO2C
9d
1H NMR (400 MHz, CDCl3)
4.96 4.94 4.94 4.93 4.93 4.91 4.91 4.90 4.32 4.30 4.29 3.73 3.72 3.16 3.14 3.12 3.11 2.99 2.98 2.96 2.95 1.32 1.32 1.31 1.30 1.29 1.29
1.00 4.05 1.98 1.05 1.04 6.02
ppm

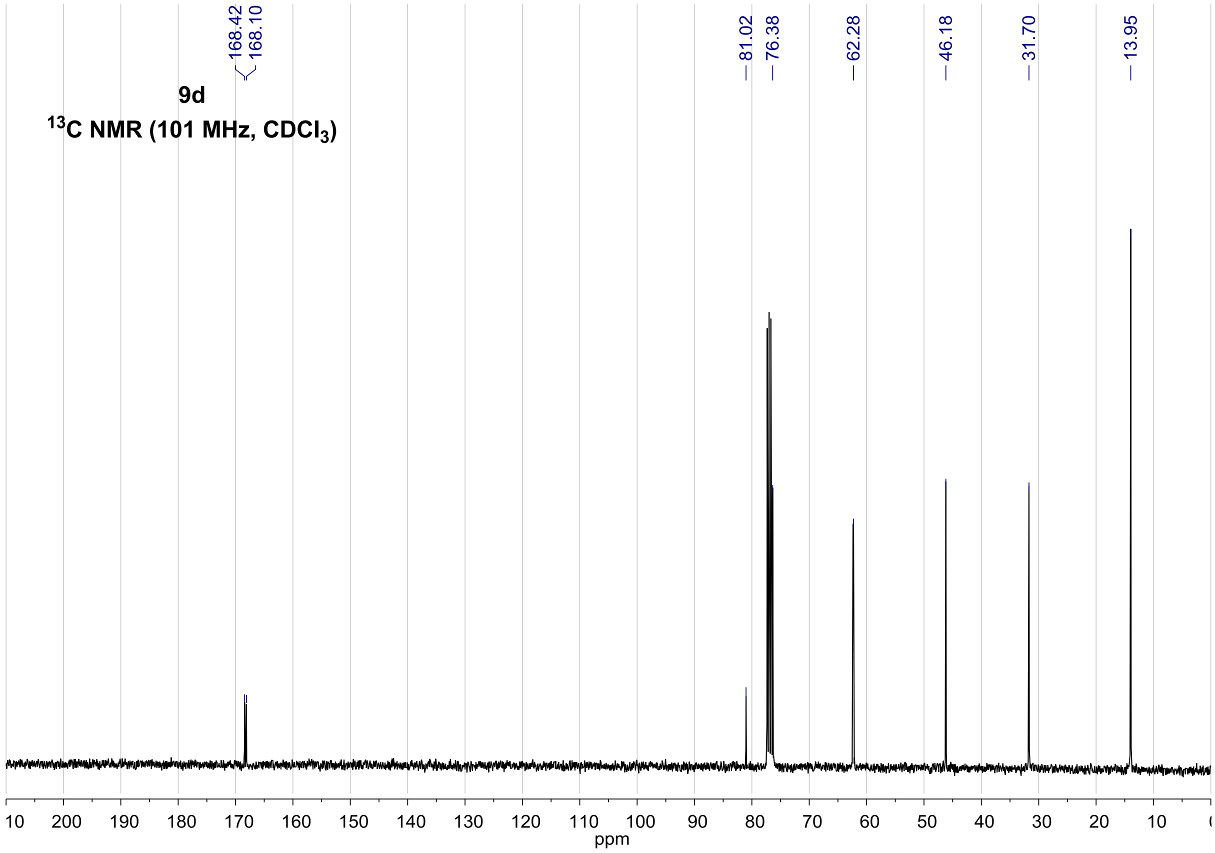
168.42 168.10 81.02 76.38 62.28 46.18 31.70 13.95
9d
13C NMR (101 MHz, CDCl3)
ppm

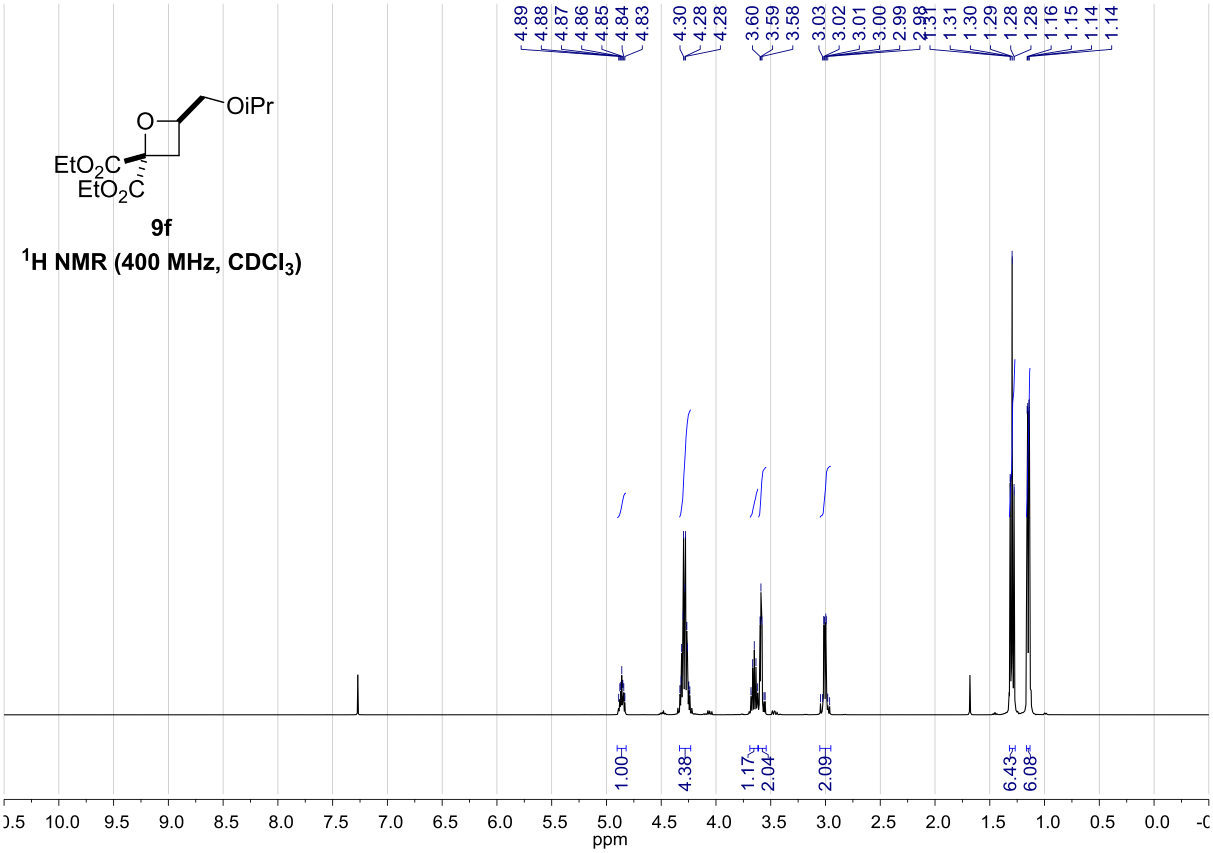
OiPr
O
EtO_2C
EtO_2C
9f
^1H NMR (400 MHz, $CDCl_3$)
4.89 4.88 4.87 4.86 4.85 4.84 4.83 4.30 4.28 4.28 3.60 3.59 3.58 3.03 3.02 3.01 3.00 2.99 2.98 1.31 1.31 1.30 1.29 1.28 1.28 1.16 1.15 1.14 1.14
1.00 4.38 1.17 2.04 2.09 6.43 6.08
ppm

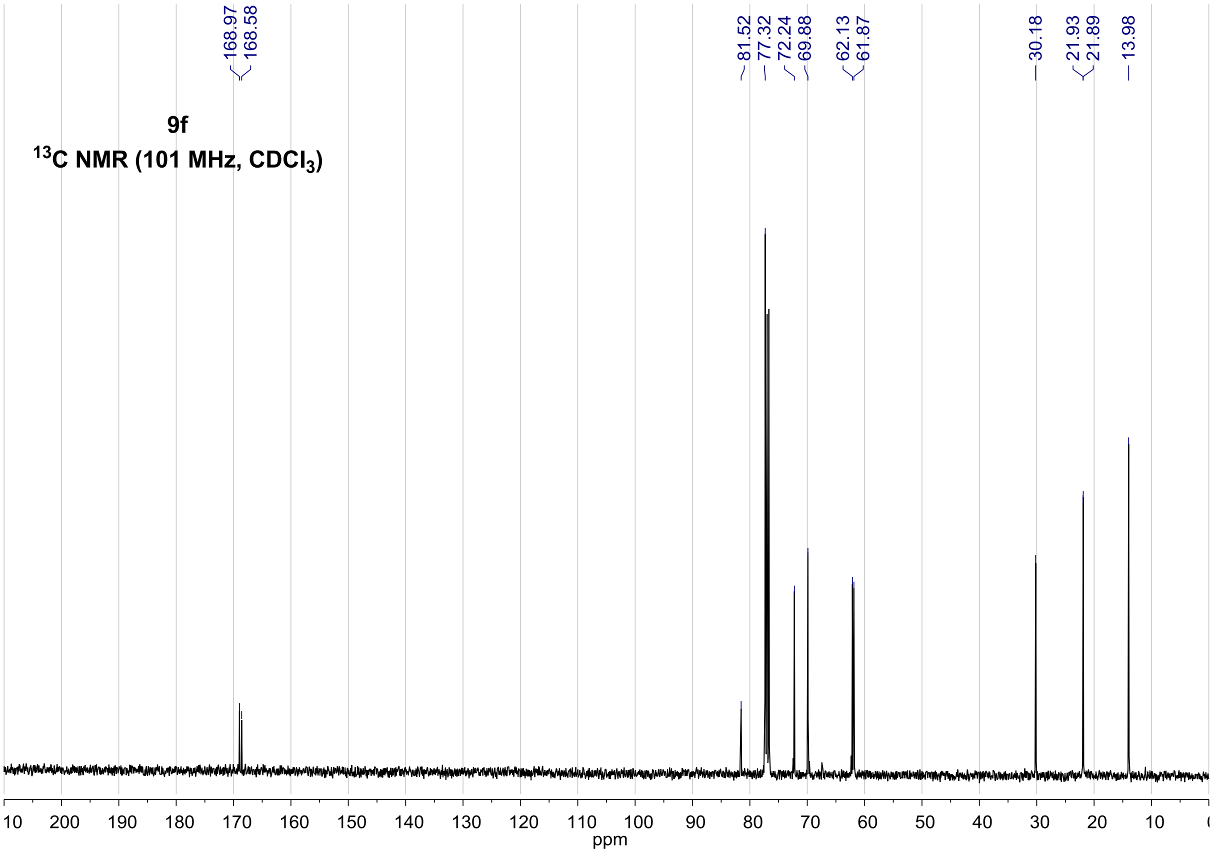
9f
^{13}C NMR (101 MHz, $CDCl_3$)
168.97 168.58 81.52 77.32 72.24 69.88 62.13 61.87 30.18 21.93 21.89 13.98
ppm

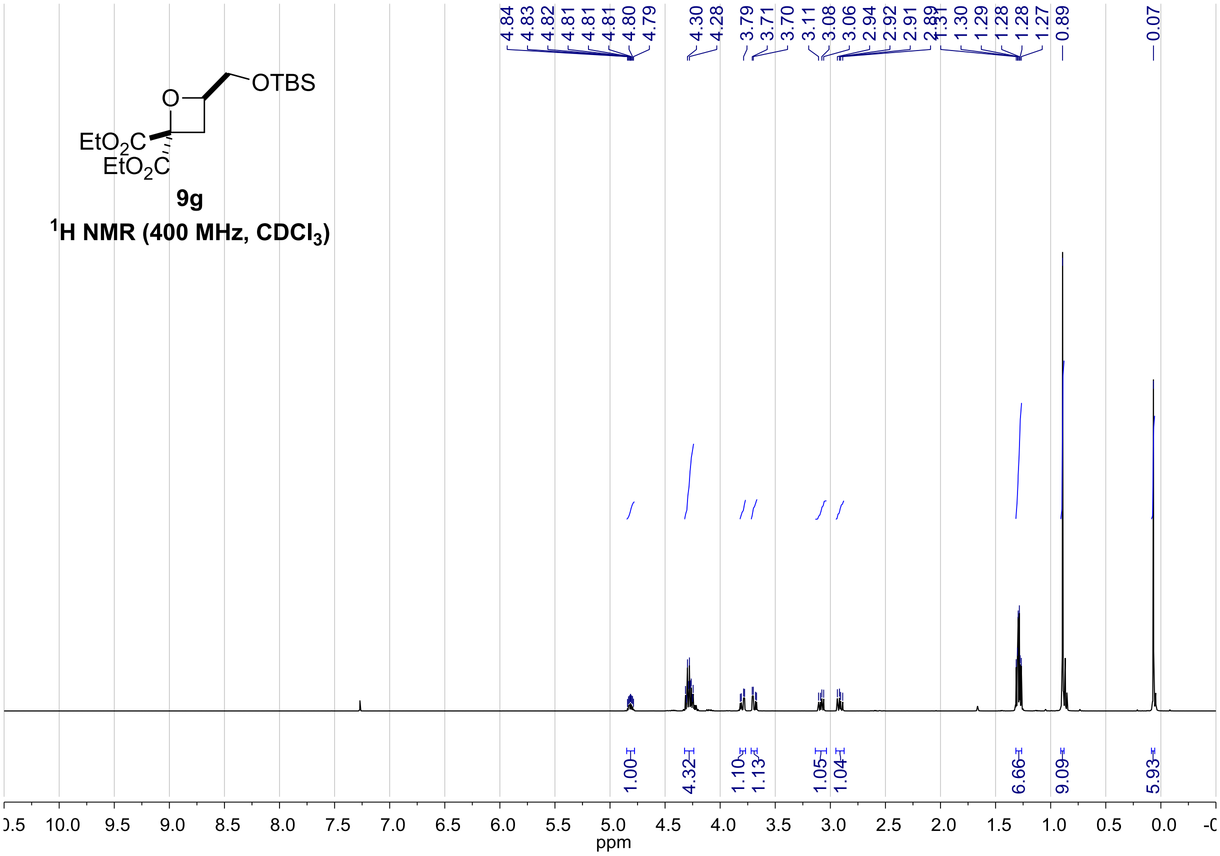
OTBS
O
EtO2C
EtO2C
9g
1H NMR (400 MHz, CDCl3)
4.84 4.83 4.82 4.81 4.81 4.81 4.80 4.79 4.30 4.28 3.79 3.71 3.70 3.11 3.08 3.06 2.94 2.92 2.91 2.89 1.31 1.30 1.29 1.28 1.28 1.27 0.89 0.07
1.00 4.32 1.10 1.13 1.05 1.04 6.66 9.09 5.93
ppm

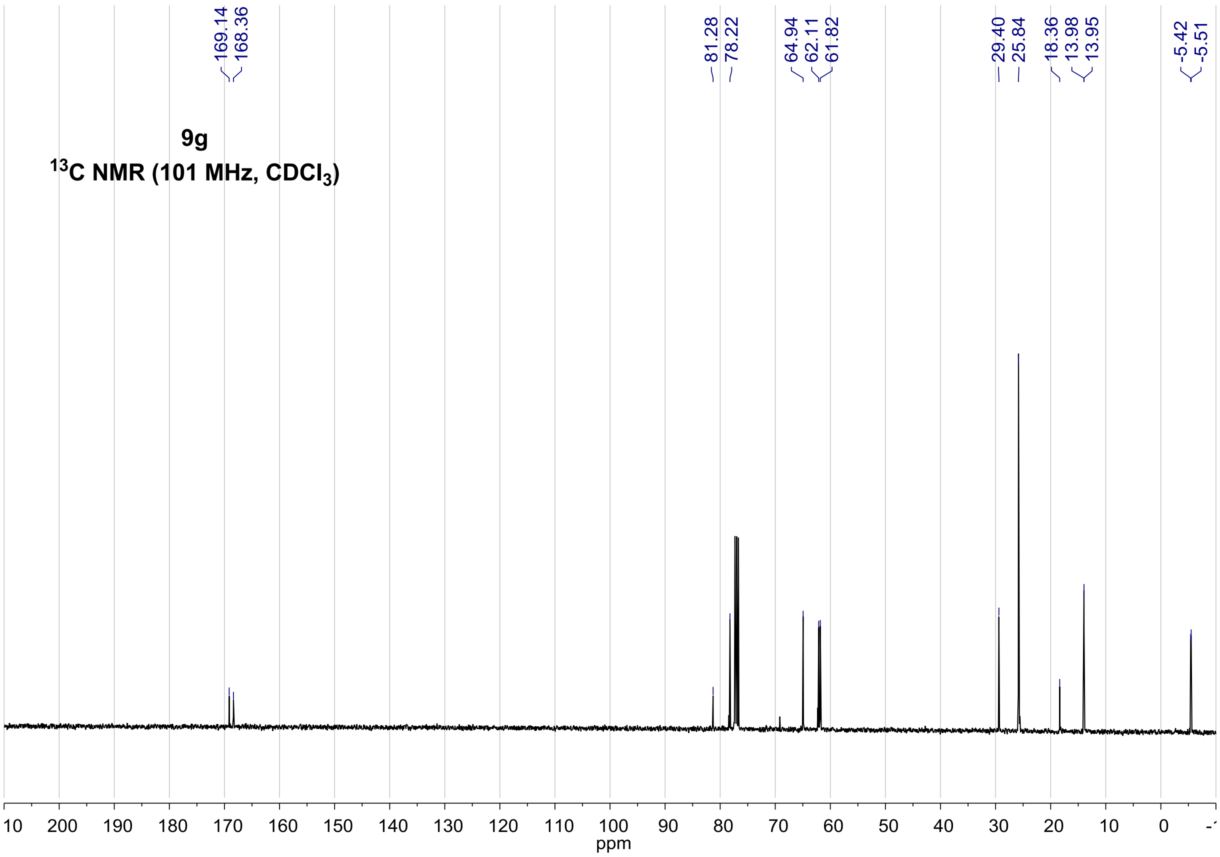
169.14 168.36 81.28 78.22 64.94 62.11 61.82 29.40 25.84 18.36 13.98 13.95 -5.42 -5.51
9g
13C NMR (101 MHz, CDCl3)
ppm

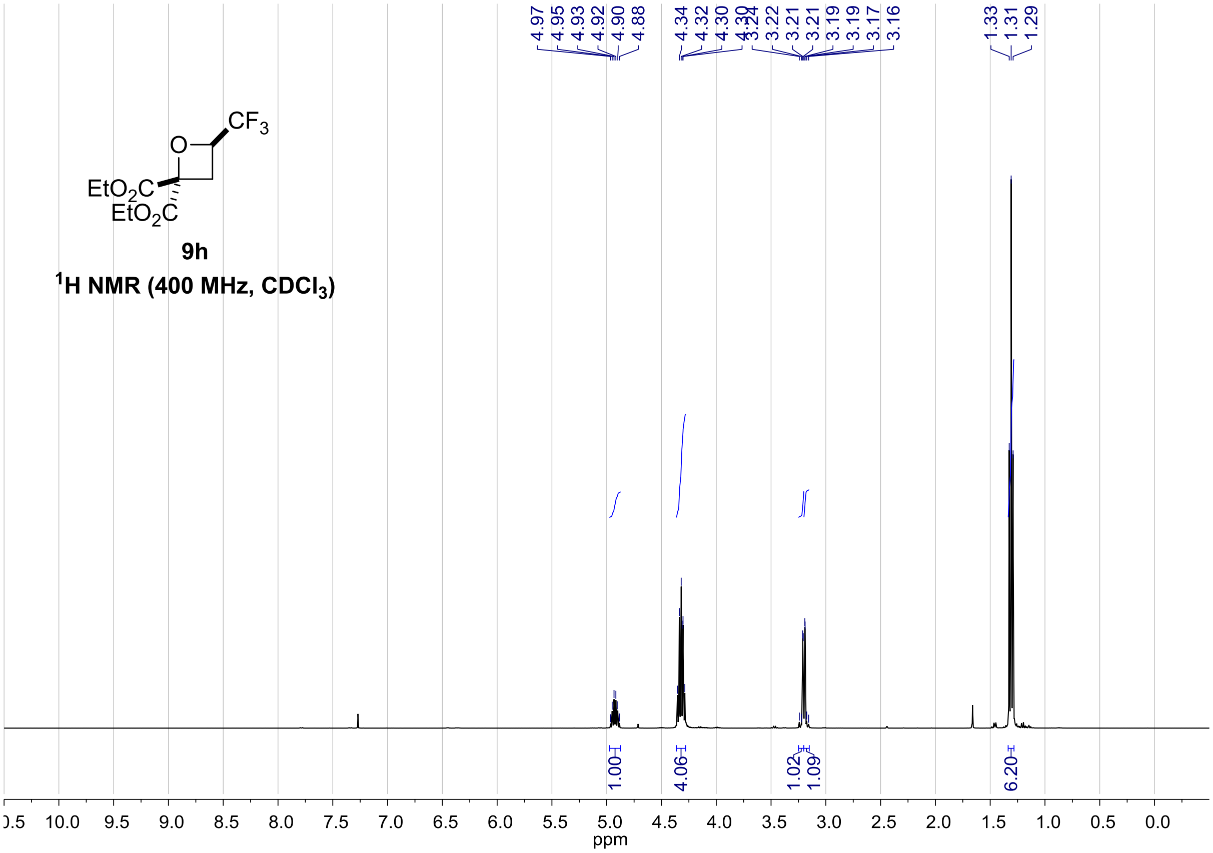
9h
^{1}H NMR (400 MHz, $CDCl_3$)
ppm

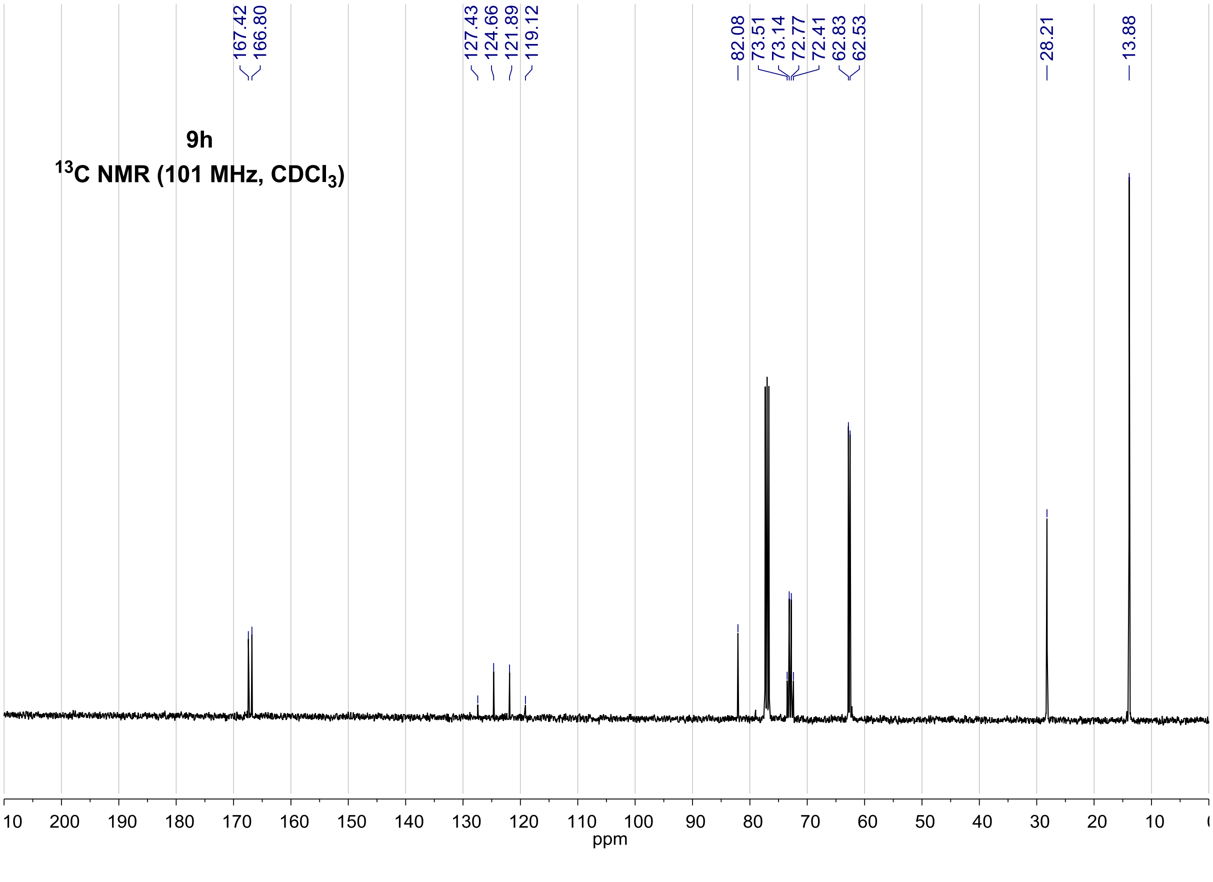
9h
^{13}C NMR (101 MHz, $CDCl_3$)
ppm

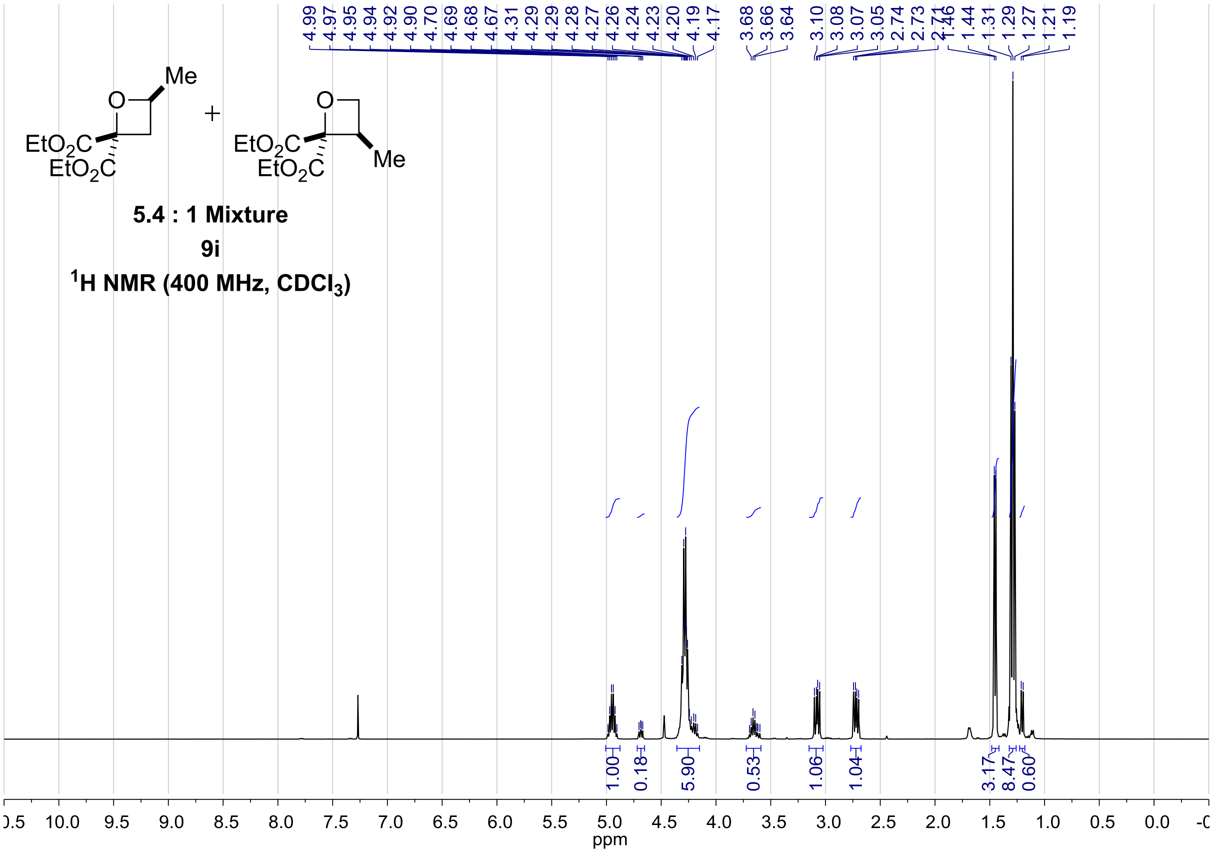
5.4 : 1 Mixture
9i
^{1}H NMR (400 MHz, $CDCl_3$)
4.99 4.97 4.95 4.94 4.92 4.90 4.70 4.69 4.68 4.67 4.31 4.29 4.29 4.28 4.27 4.26 4.24 4.23 4.20 4.19 4.17 3.68 3.66 3.64 3.10 3.08 3.07 3.05 2.74 2.73 2.71 1.46 1.44 1.31 1.29 1.27 1.21 1.19
1.00 0.18 5.90 0.53 1.06 1.04 3.17 8.47 0.60
ppm

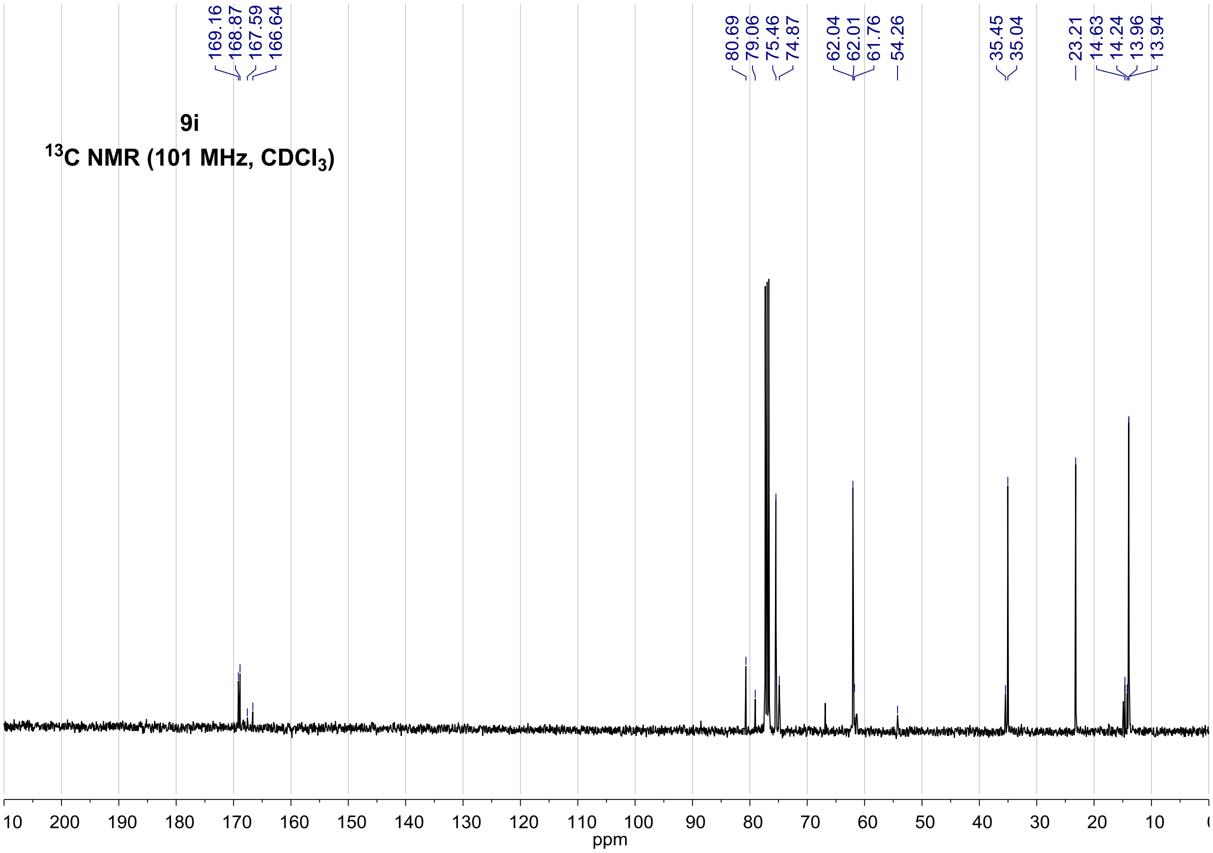
9i
^{13}C NMR (101 MHz, $CDCl_3$)
169.16 168.87 167.59 166.64 80.69 79.06 75.46 74.87 62.04 62.01 61.76 54.26 35.45 35.04 23.21 14.63 14.24 13.96 13.94
ppm

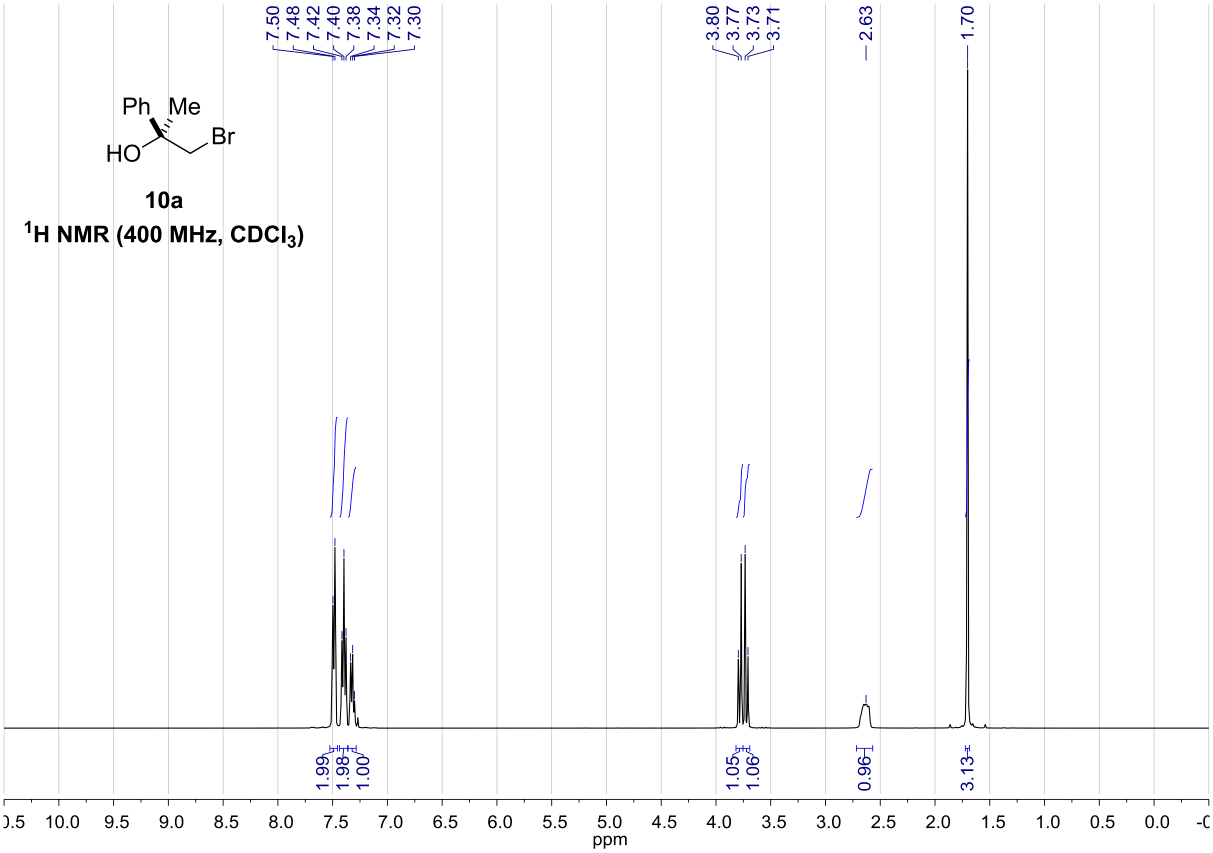
7.50
7.48
7.42
7.40
7.38
7.34
7.32
7.30
3.80
3.77
3.73
3.71
2.63
1.70
Ph
Me
Br
HO
10a
1H NMR (400 MHz, CDCl3)
1.99
1.98
1.00
1.05
1.06
0.96
3.13
10.0 9.5 9.0 8.5 8.0 7.5 7.0 6.5 6.0 5.5 5.0 4.5 4.0 3.5 3.0 2.5 2.0 1.5 1.0 0.5 0.0
ppm

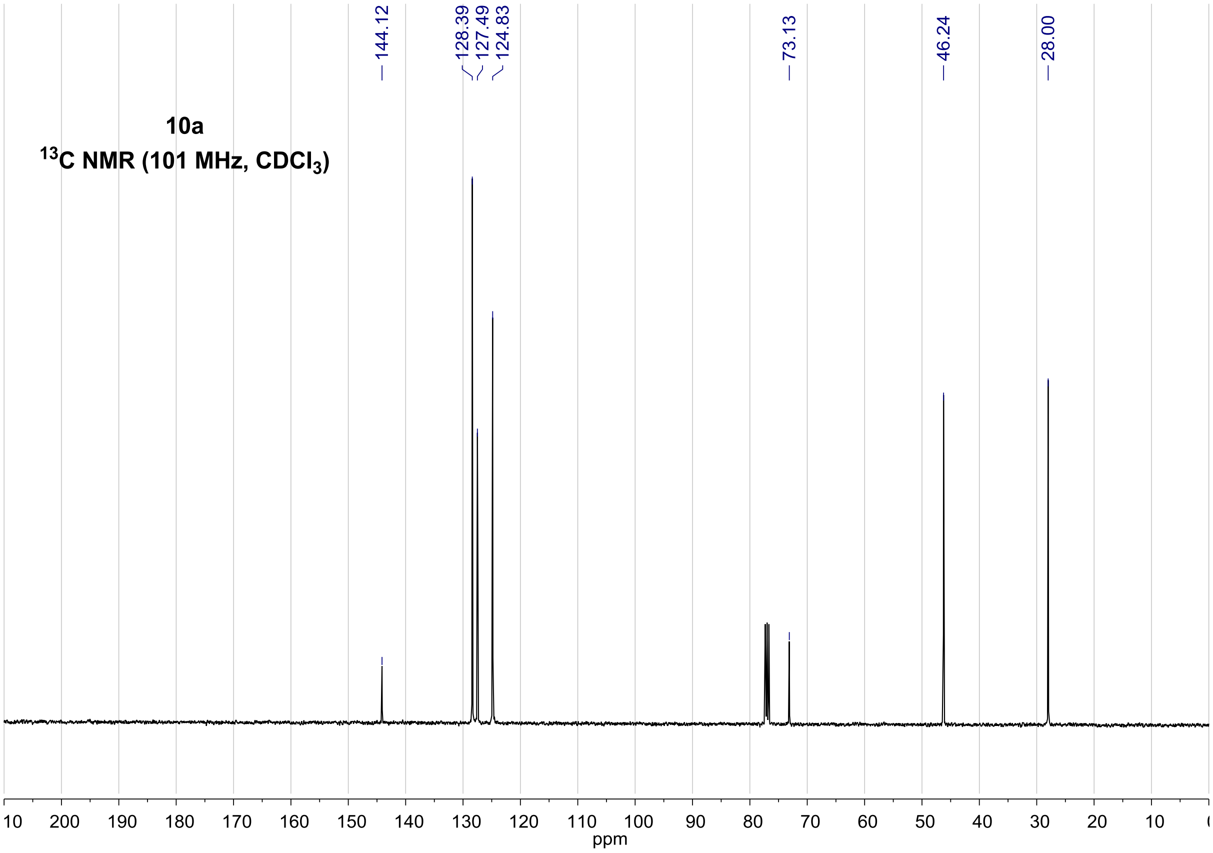
144.12
128.39
127.49
124.83
73.13
46.24
28.00
10a
13C NMR (101 MHz, CDCl3)
200 190 180 170 160 150 140 130 120 110 100 90 80 70 60 50 40 30 20 10
ppm

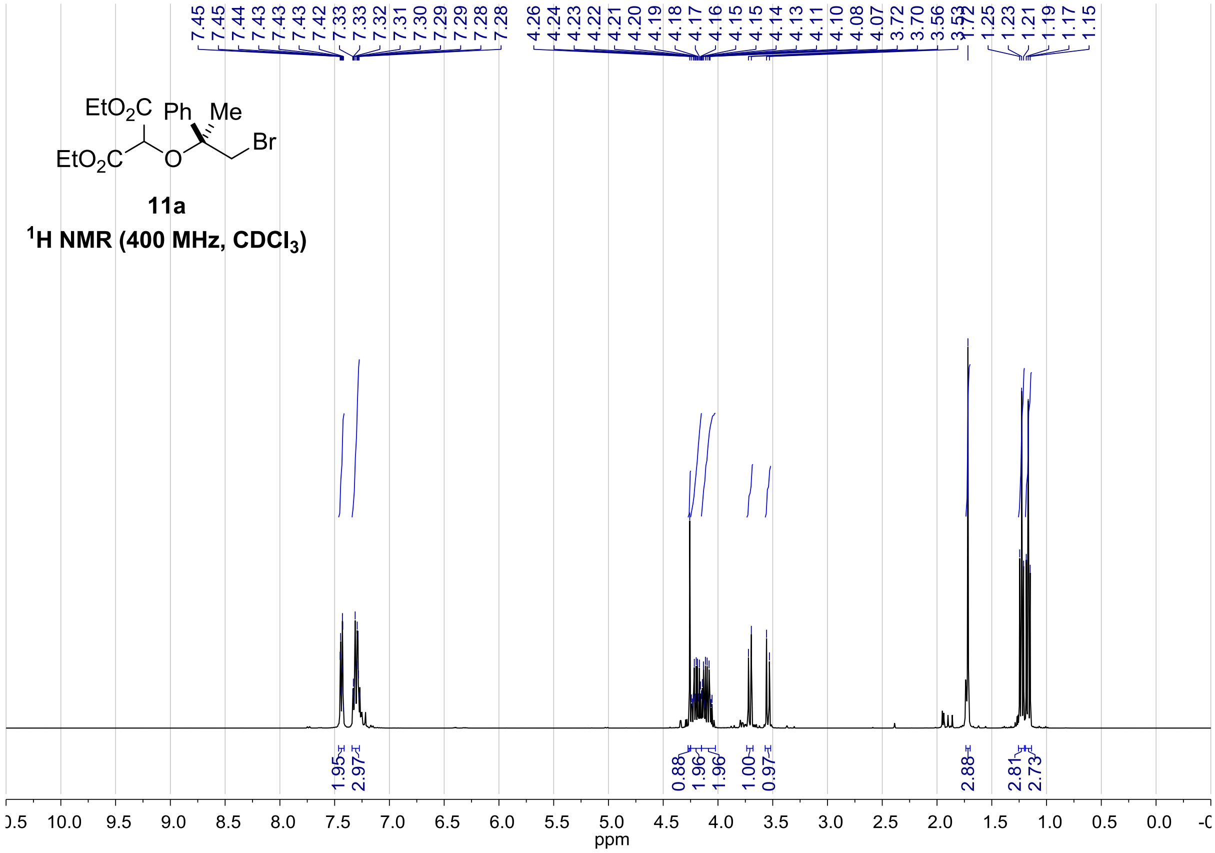

7.45 7.45 7.44 7.43 7.43 7.43 7.42 7.33 7.33 7.32 7.31 7.30 7.29 7.29 7.28 7.28 4.26 4.24 4.23 4.22 4.21 4.20 4.19 4.18 4.17 4.16 4.15 4.15 4.14 4.13 4.11 4.10 4.08 4.07 3.72 3.70 3.56 3.53 1.72 1.25 1.23 1.21 1.19 1.17 1.15
EtO2C
EtO2C
Ph
Me
O
Br
11a
1H NMR (400 MHz, CDCl3)
1.95 2.97 0.88 1.96 1.96 1.00 0.97 2.88 2.81 2.73
ppm

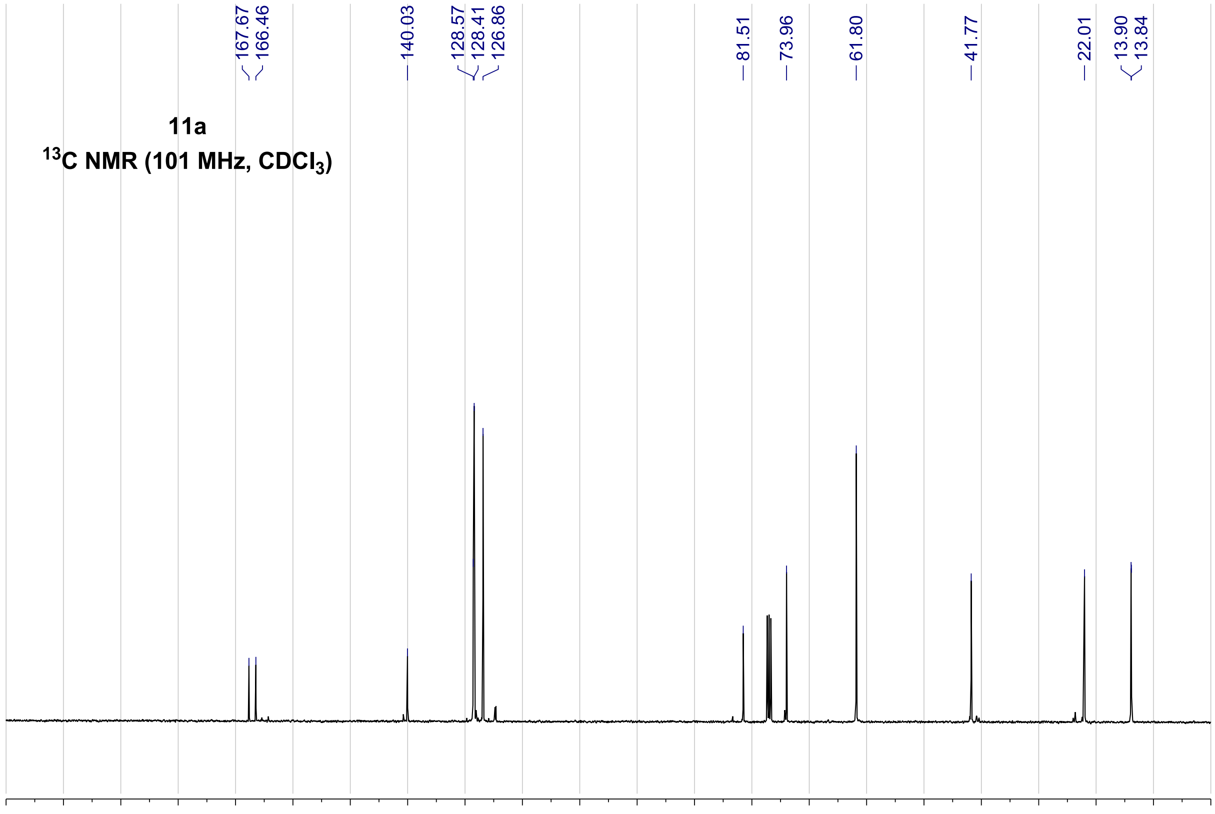

167.67 166.46 140.03 128.57 128.41 126.86 81.51 73.96 61.80 41.77 22.01 13.90 13.84
11a
13C NMR (101 MHz, CDCl3)
ppm

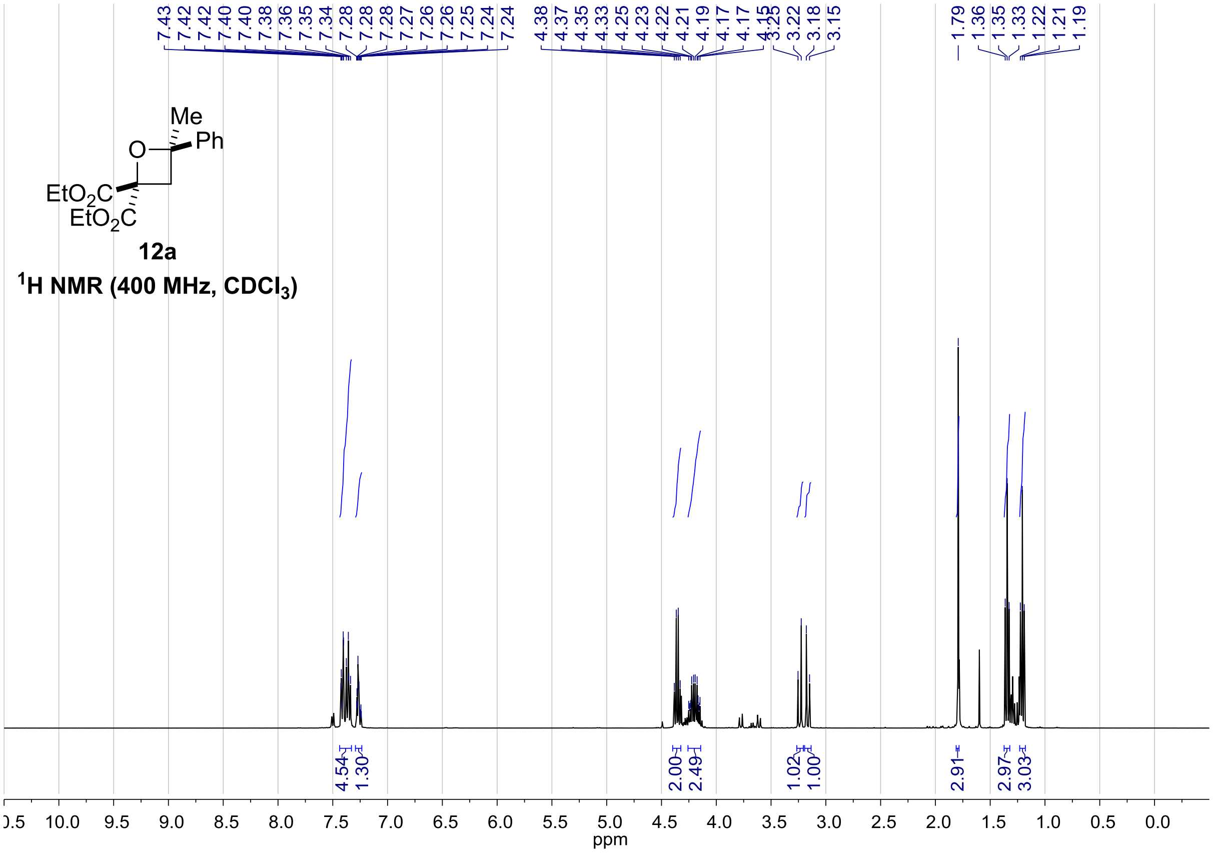

12a

^{1}H NMR (400 MHz, $CDCl_3$)

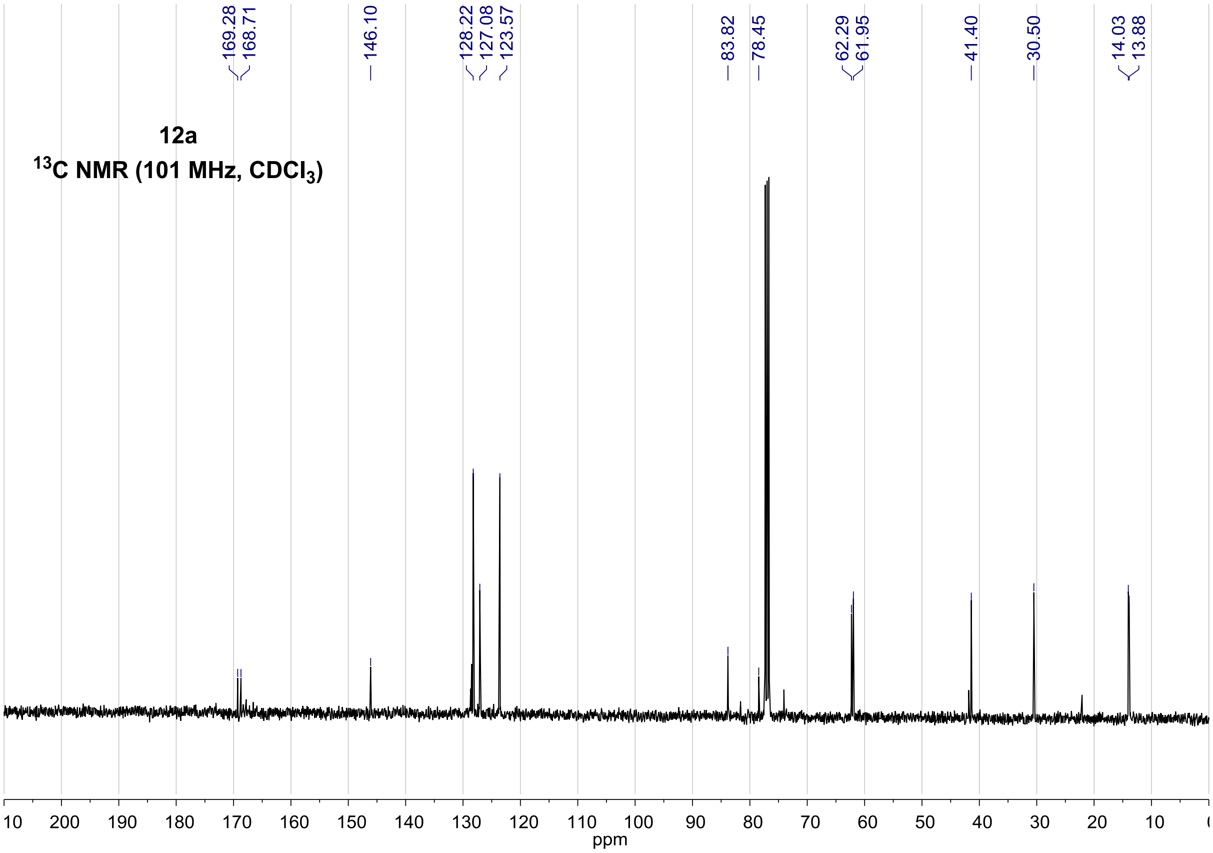

12a

^{13}C NMR (101 MHz, $CDCl_3$)

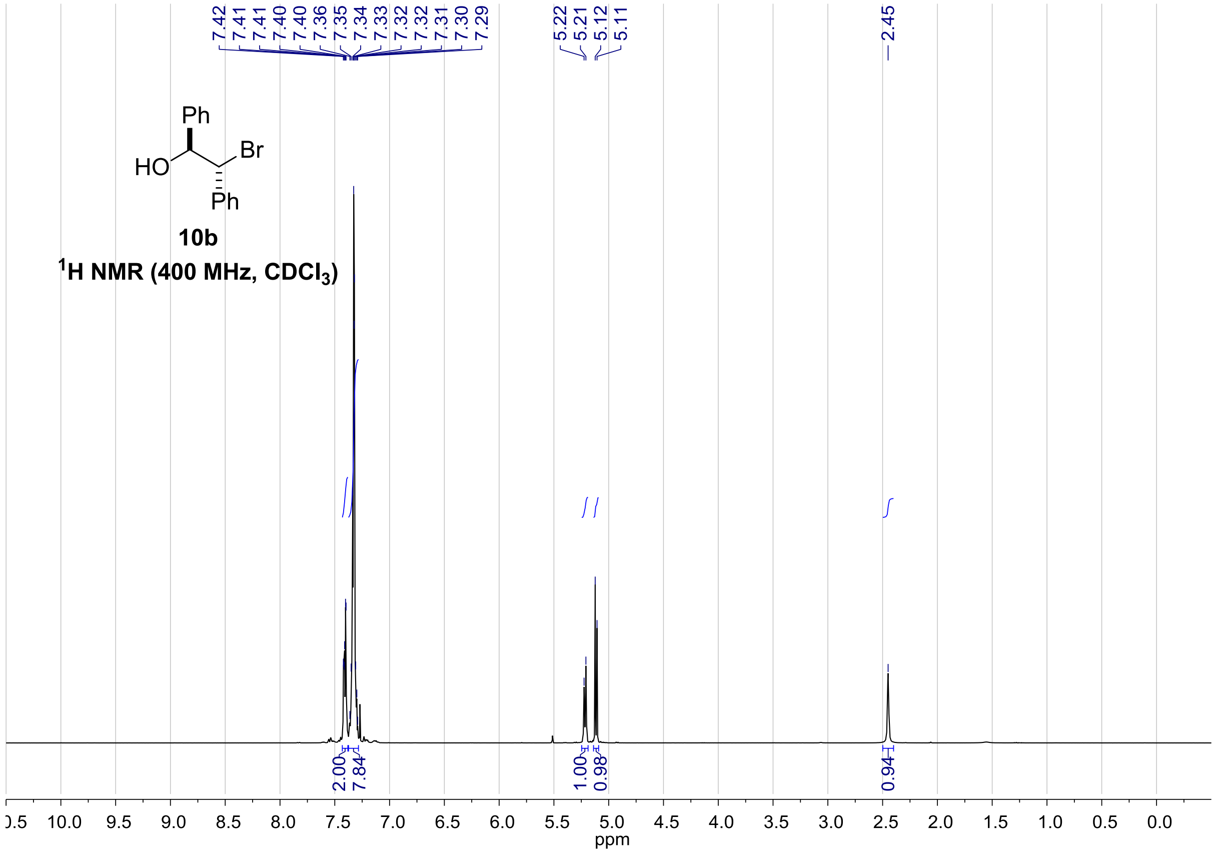
7.42 7.41 7.41 7.40 7.40 7.36 7.35 7.34 7.33 7.32 7.32 7.31 7.30 7.29
5.22 5.21 5.12 5.11
2.45
Ph
HO
Br
Ph
10b
1H NMR (400 MHz, CDCl3)
2.00 7.84 1.00 0.98 0.94
ppm

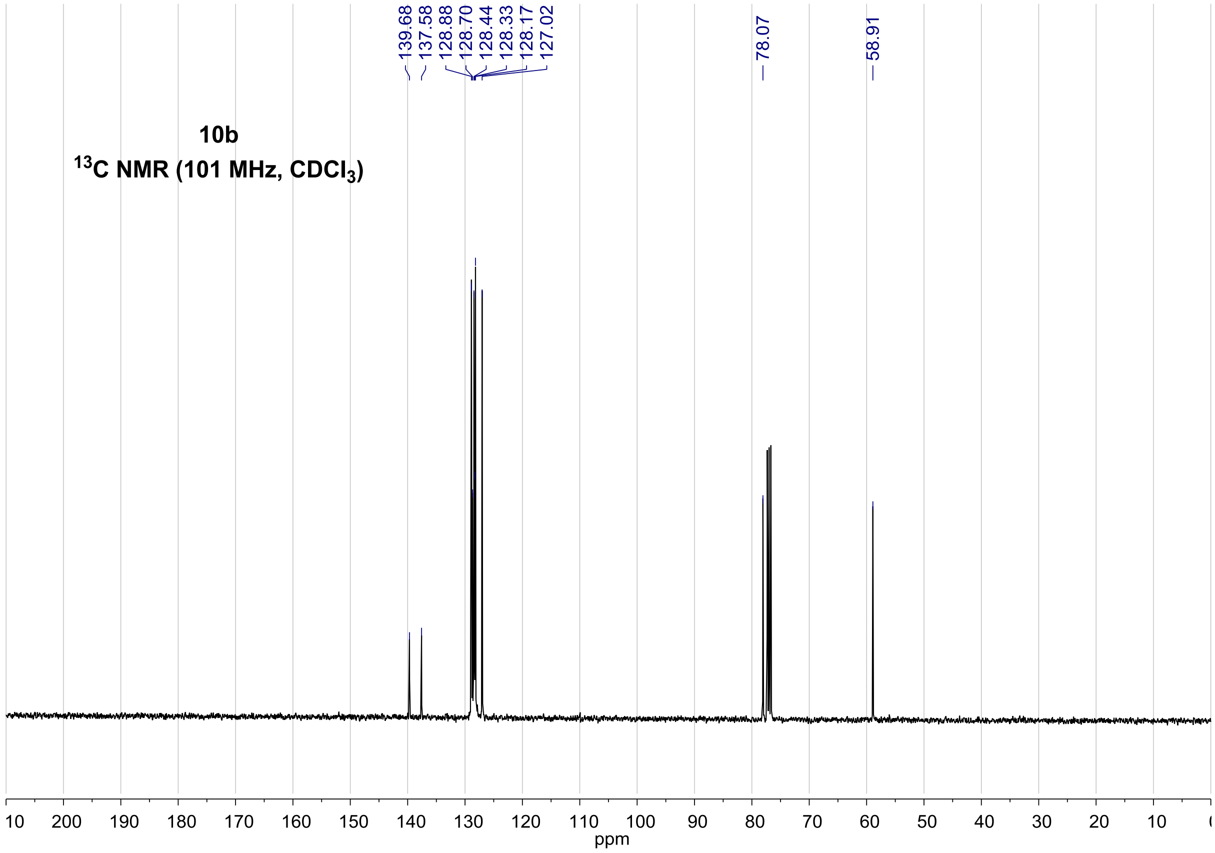
139.68 137.58 128.88 128.70 128.44 128.33 128.17 127.02
78.07
58.91
10b
13C NMR (101 MHz, CDCl3)
ppm

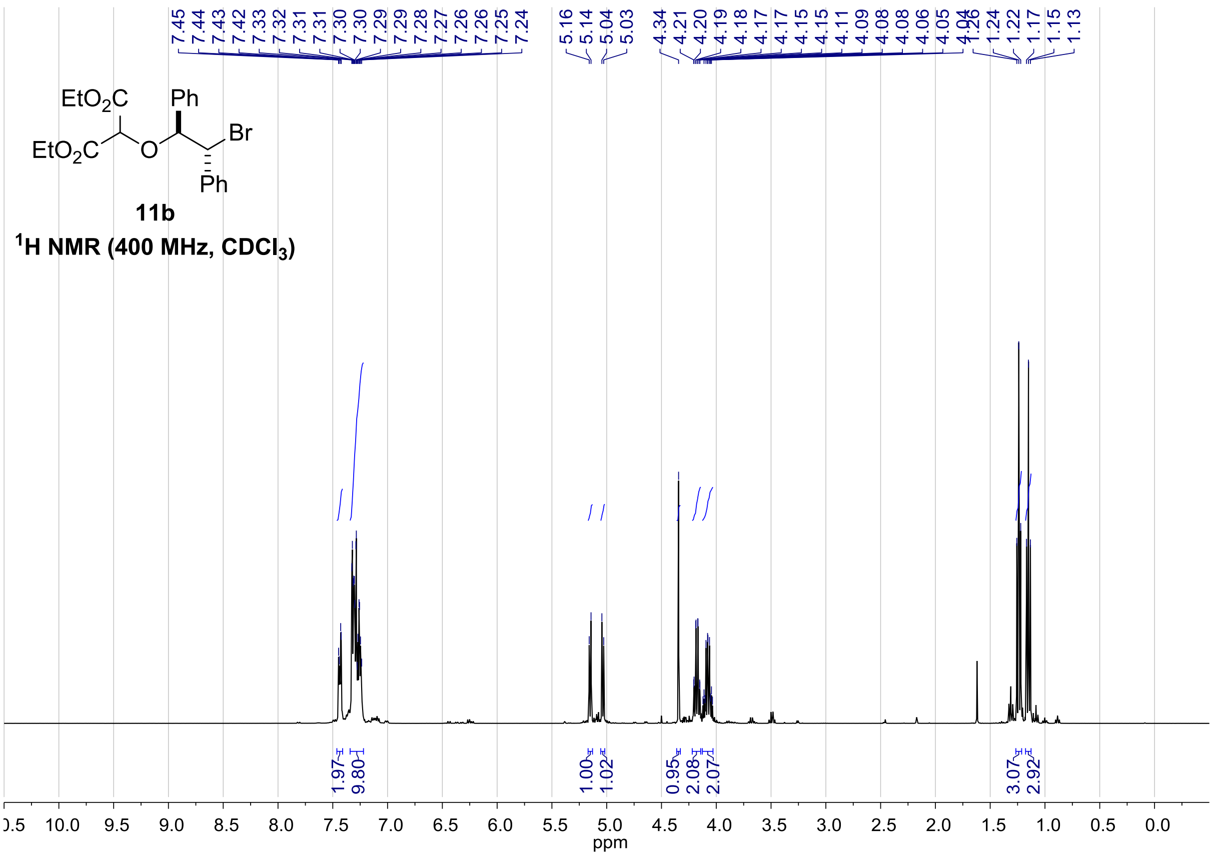
7.45 7.44 7.43 7.42 7.33 7.32 7.31 7.31 7.30 7.30 7.29 7.29 7.28 7.27 7.26 7.26 7.25 7.24
5.16 5.14 5.04 5.03
4.34 4.21 4.20 4.19 4.18 4.17 4.15 4.15 4.11 4.09 4.08 4.08 4.06 4.05 4.04
1.26 1.24 1.22 1.17 1.15 1.13
EtO2C
Ph
Br
EtO2C
O
Ph
11b
1H NMR (400 MHz, CDCl3)
1.97 9.80 1.00 1.02 0.95 2.08 2.07 3.07 2.92
ppm

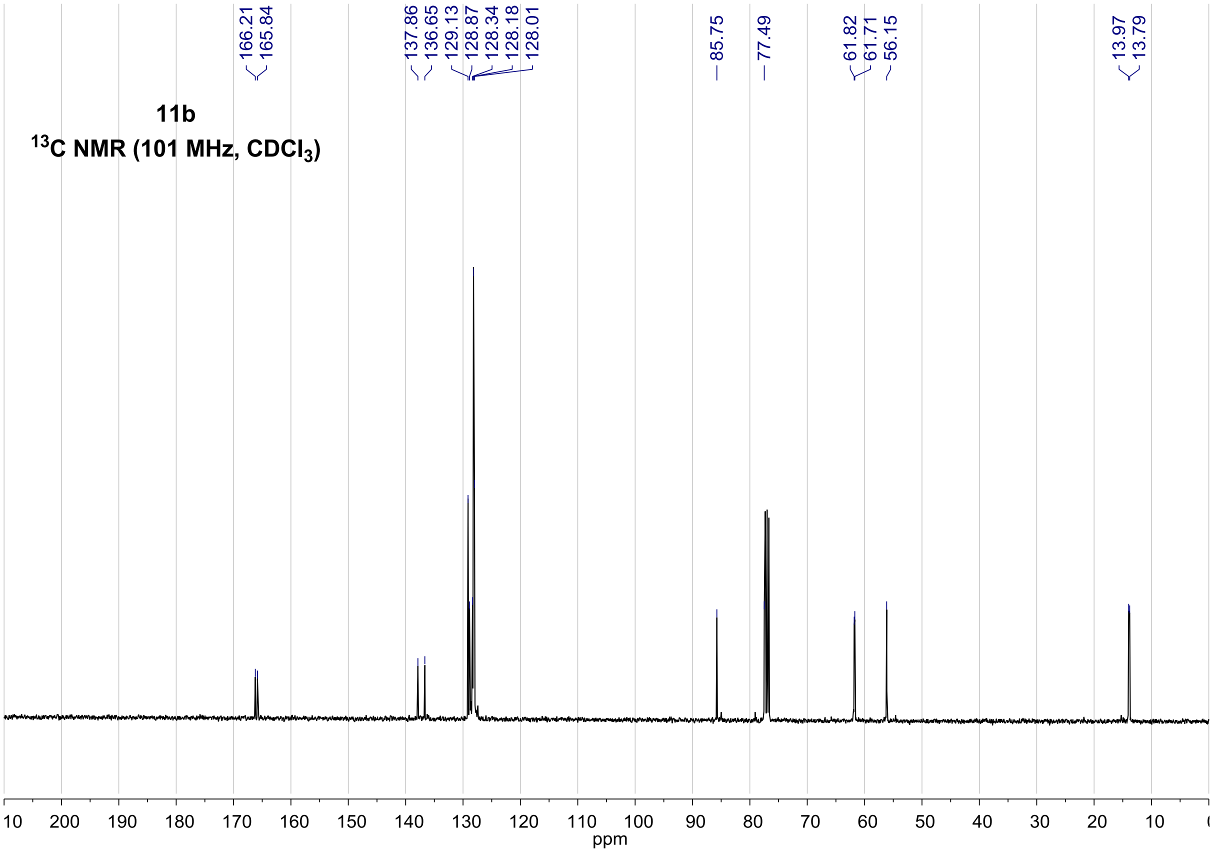
166.21 165.84
137.86 136.65 129.13 128.87 128.34 128.18 128.01
85.75
77.49
61.82 61.71 56.15
13.97 13.79
11b
13C NMR (101 MHz, CDCl3)
ppm

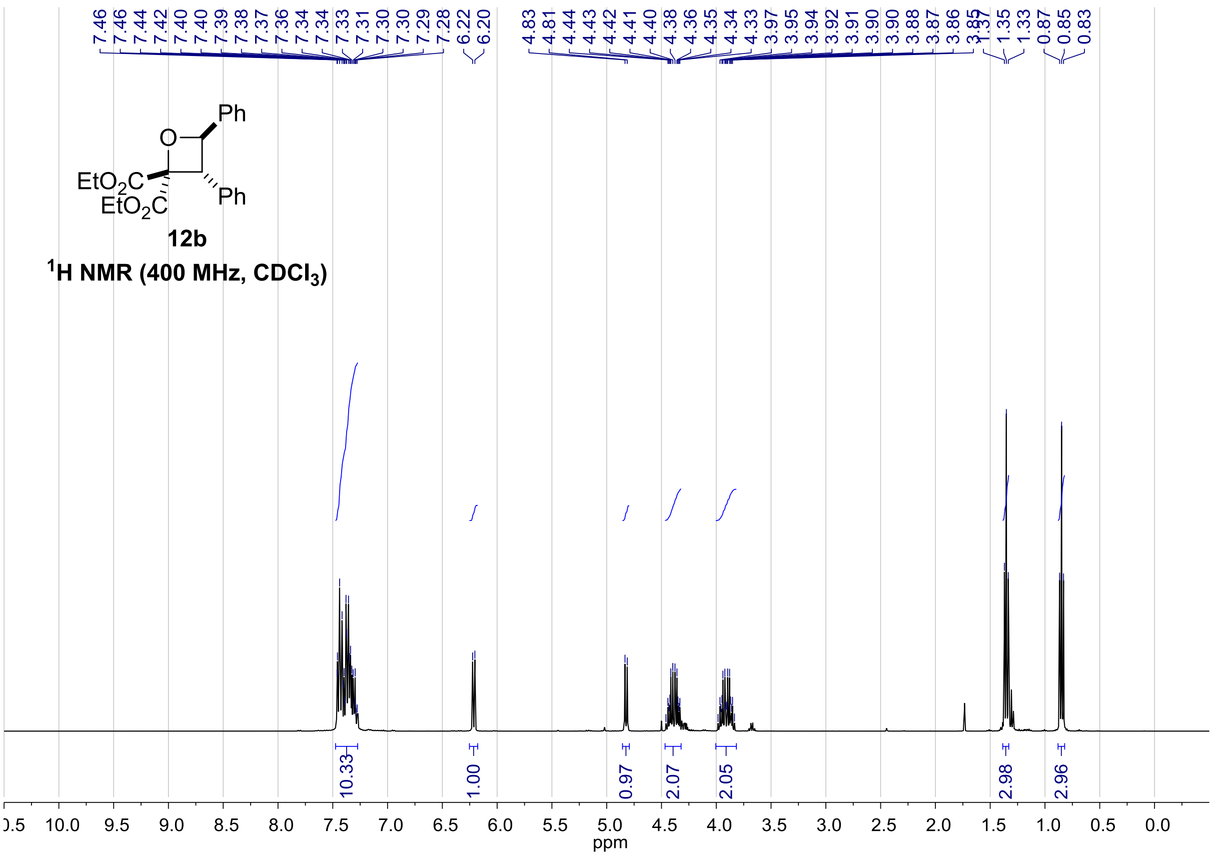
7.46
7.46
7.44
7.42
7.40
7.40
7.39
7.38
7.37
7.36
7.34
7.34
7.33
7.31
7.30
7.30
7.29
7.28
6.22
6.20
4.83
4.81
4.44
4.43
4.42
4.41
4.40
4.38
4.36
4.35
4.34
4.33
3.97
3.95
3.94
3.92
3.91
3.90
3.90
3.88
3.87
3.86
3.85
1.37
1.35
1.33
0.87
0.85
0.83
Ph
O
EtO2C
EtO2C
Ph
12b
1H NMR (400 MHz, CDCl3)
10.33
1.00
0.97
2.07
2.05
2.98
2.96
ppm

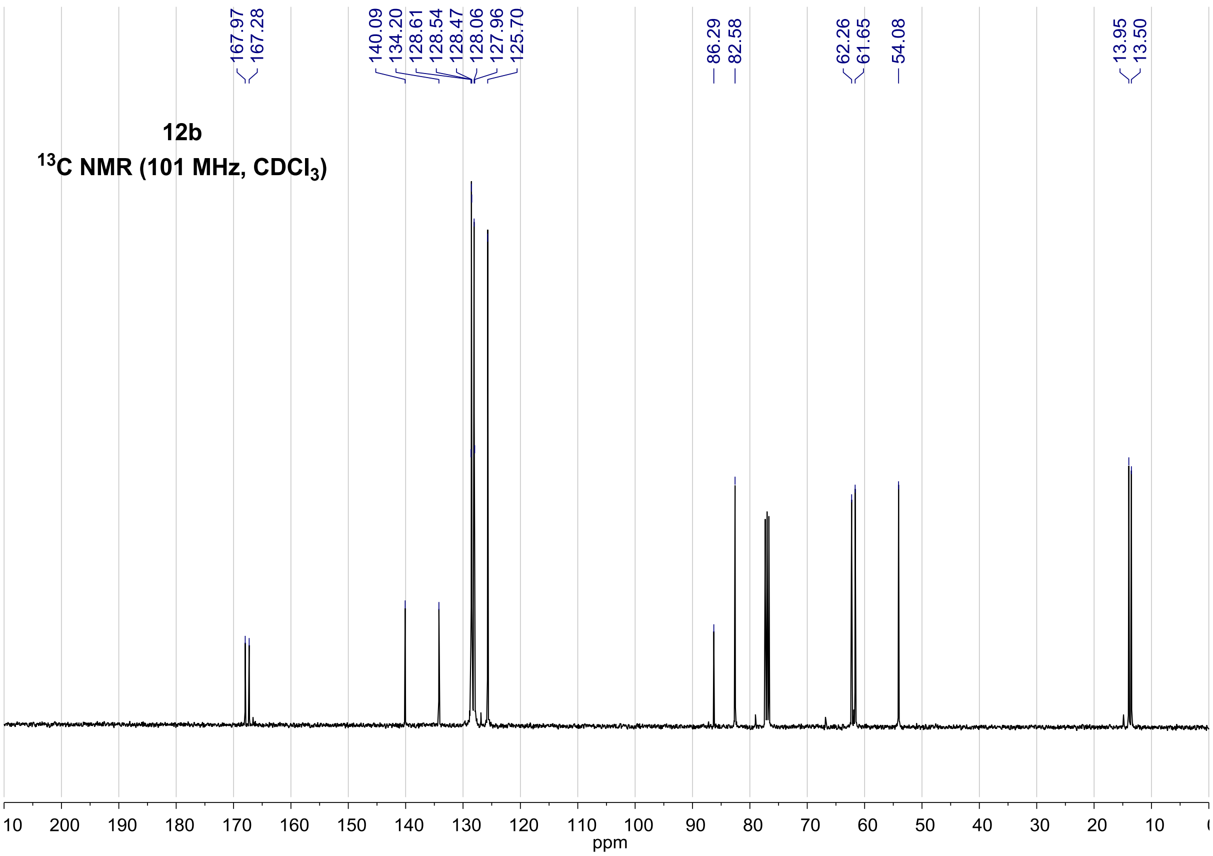
167.97
167.28
140.09
134.20
128.61
128.54
128.47
128.06
127.96
125.70
86.29
82.58
62.26
61.65
54.08
13.95
13.50
12b
13C NMR (101 MHz, CDCl3)
ppm

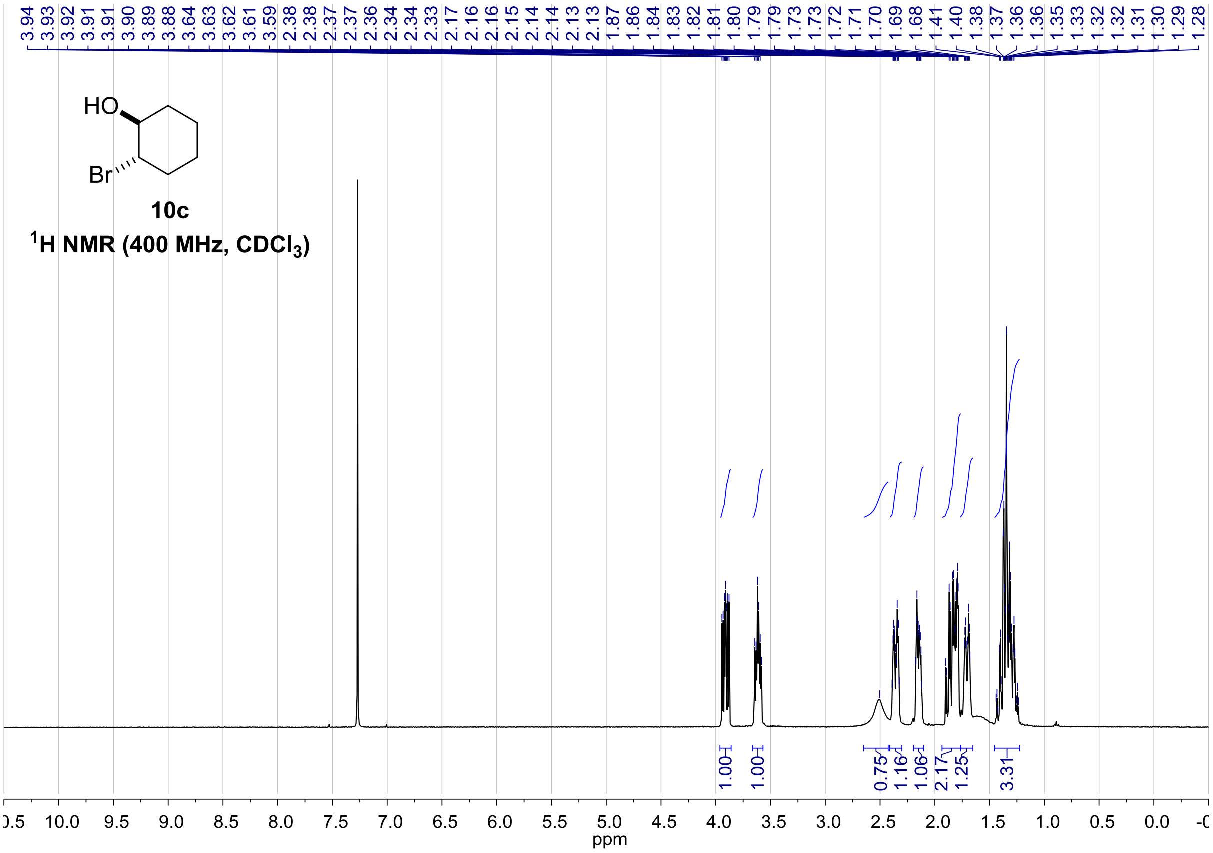
HO
Br
10c
1H NMR (400 MHz, CDCl3)
ppm

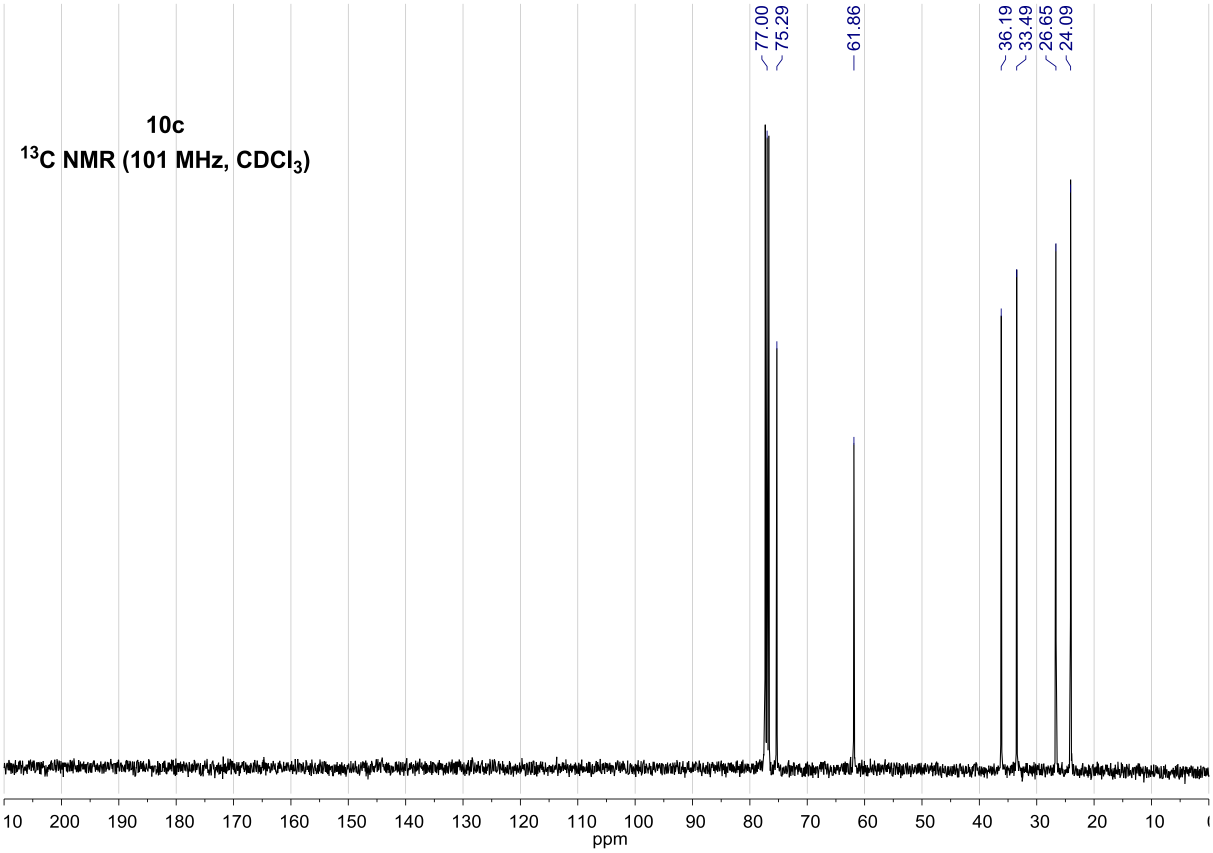
10c
13C NMR (101 MHz, CDCl3)
77.00
75.29
61.86
36.19
33.49
26.65
24.09
ppm

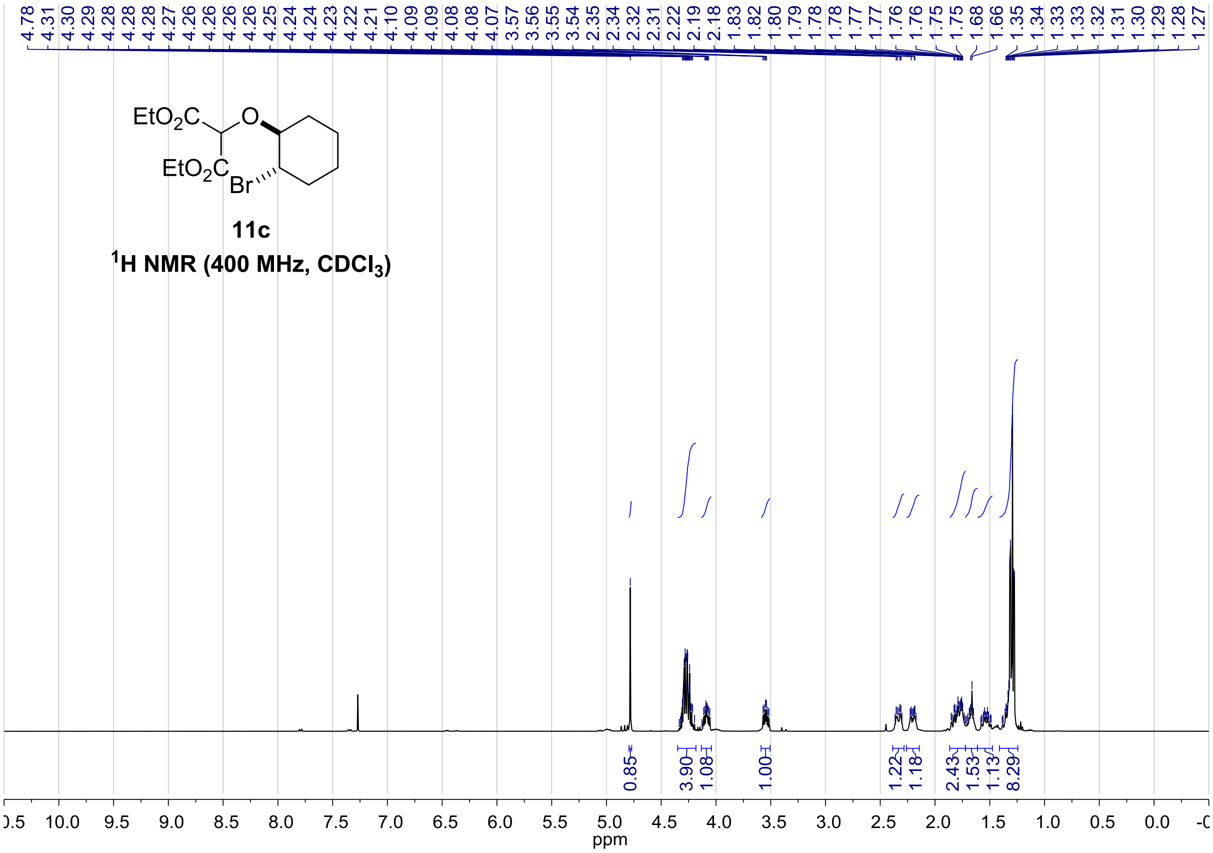
4.78 4.31 4.30 4.29 4.28 4.28 4.28 4.27 4.26 4.26 4.26 4.26 4.25 4.24 4.24 4.23 4.22 4.21 4.10 4.09 4.09 4.08 4.08 4.07 3.57 3.56 3.55 3.54 2.35 2.34 2.32 2.31 2.22 2.19 2.18 1.83 1.82 1.80 1.79 1.78 1.78 1.77 1.77 1.76 1.76 1.75 1.75 1.68 1.66 1.35 1.34 1.33 1.33 1.32 1.31 1.30 1.29 1.28 1.27
EtO2C
O
EtO2C
Br
11c
1H NMR (400 MHz, CDCl3)
0.85 3.90 1.08 1.00 1.22 1.18 2.43 1.53 1.13 8.29
ppm

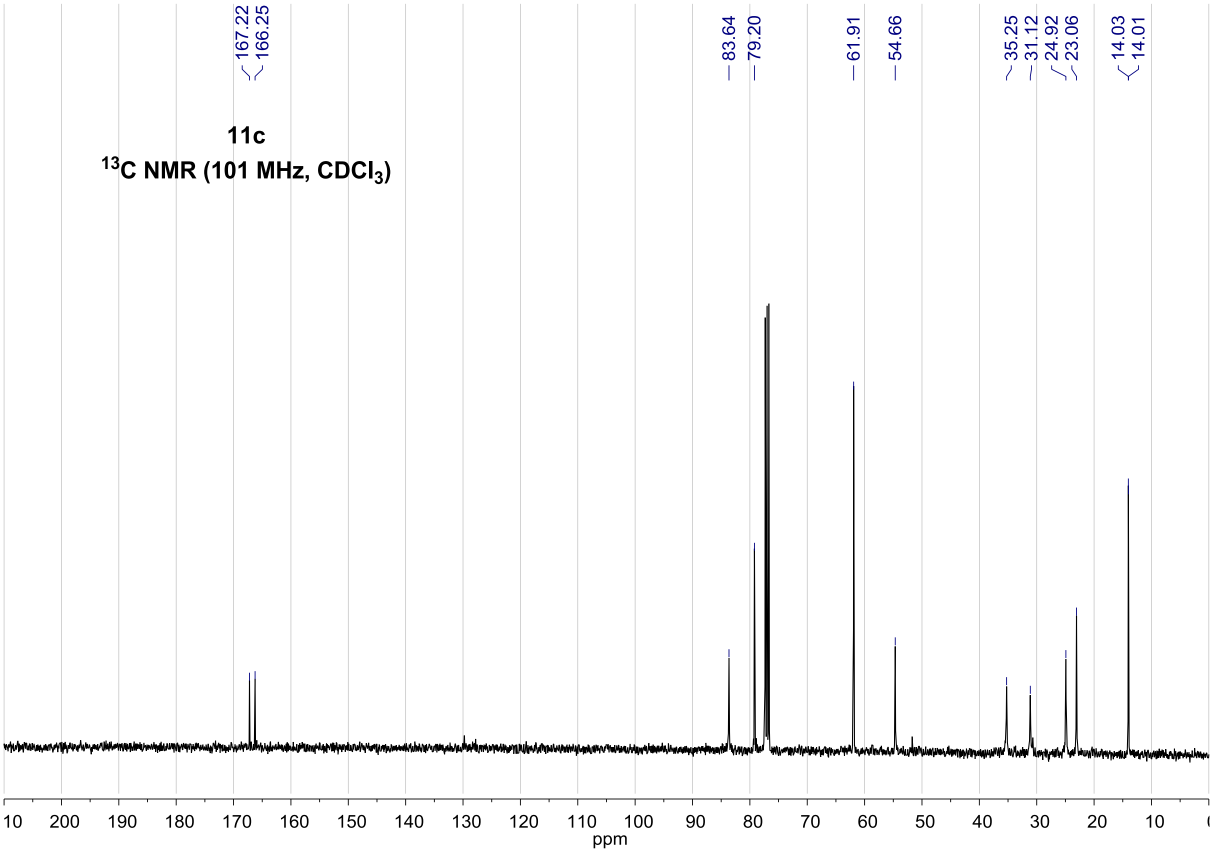
167.22 166.25 83.64 79.20 61.91 54.66 35.25 31.12 24.92 23.06 14.03 14.01
11c
13C NMR (101 MHz, CDCl3)
ppm

12c

^1H NMR (400 MHz, $CDCl_3$)

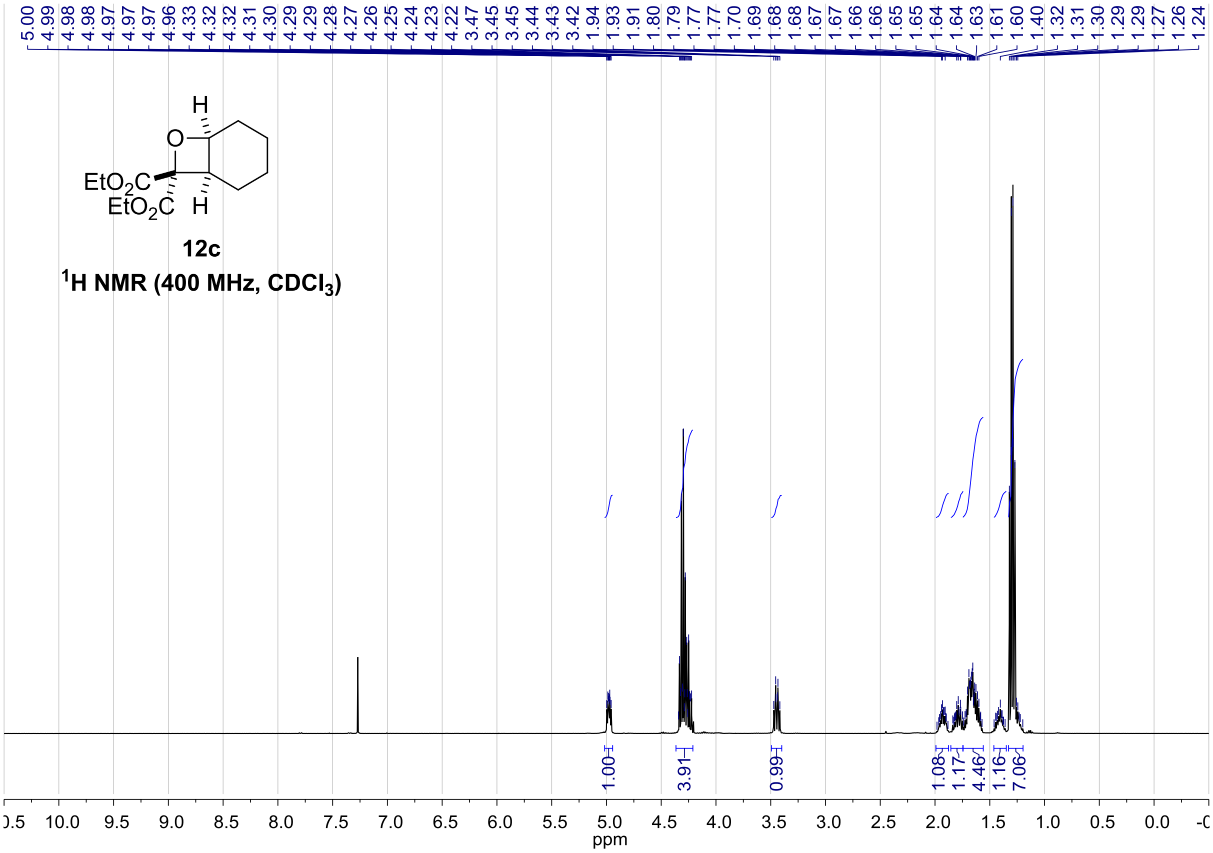

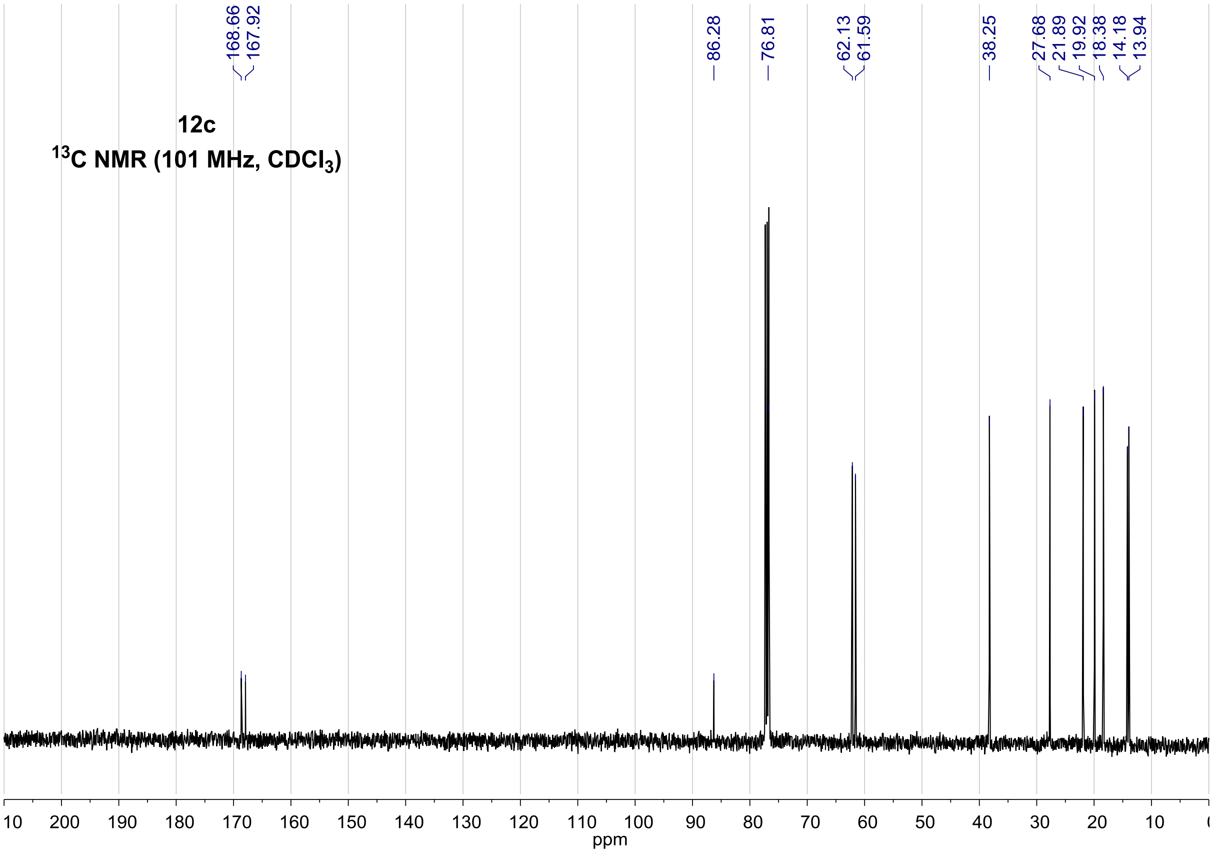

12c

^{13}C NMR (101 MHz, $CDCl_3$)

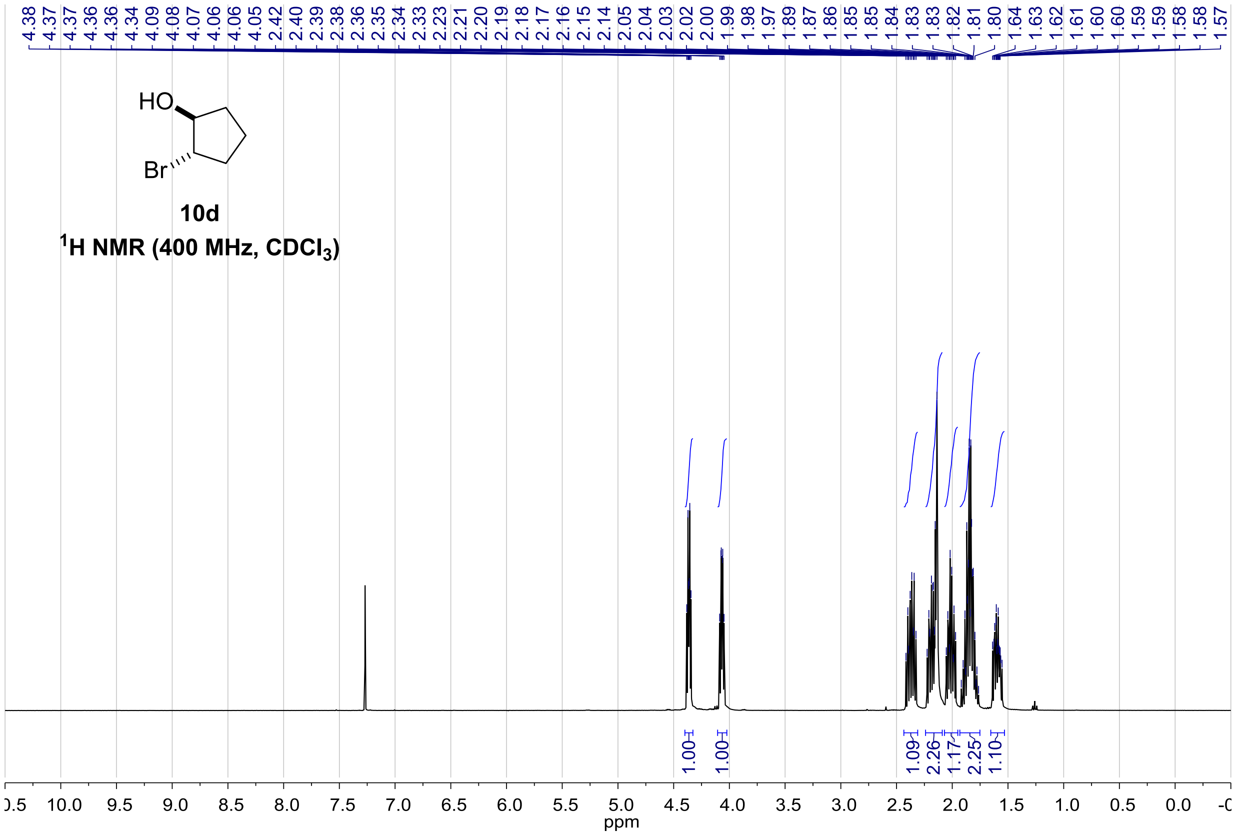
HO
Br
10d
^{1}H NMR (400 MHz, $CDCl_3$)
4.38 4.37 4.36 4.36 4.34 4.09 4.08 4.07 4.06 4.06 4.05 2.42 2.40 2.39 2.38 2.36 2.35 2.34 2.33 2.23 2.21 2.20 2.19 2.18 2.17 2.16 2.15 2.14 2.05 2.04 2.03 2.02 2.00 1.99 1.98 1.97 1.89 1.87 1.86 1.85 1.85 1.84 1.83 1.83 1.82 1.81 1.80 1.64 1.63 1.62 1.61 1.60 1.60 1.59 1.59 1.58 1.57
1.00 1.00 1.09 2.26 1.17 2.25 1.10
ppm

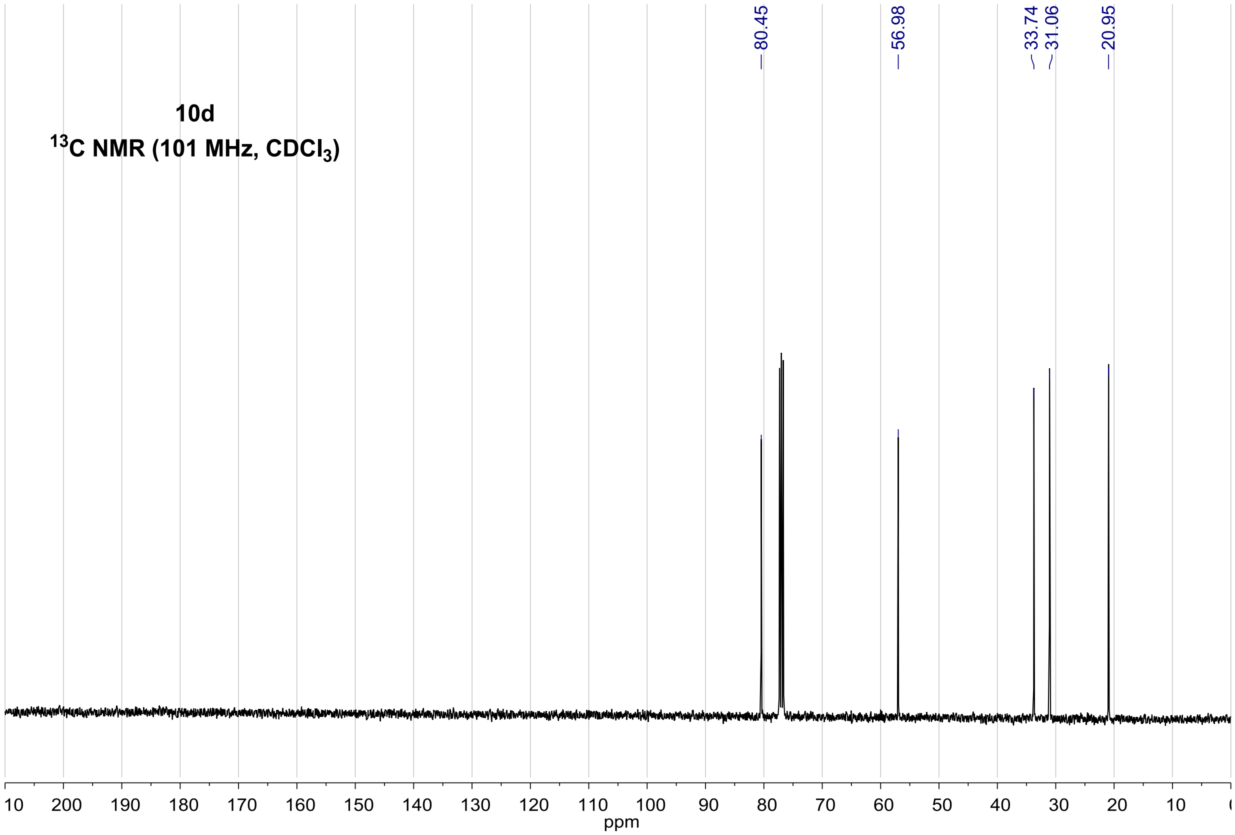
80.45
56.98
33.74
31.06
20.95
10d
^{13}C NMR (101 MHz, $CDCl_3$)
ppm

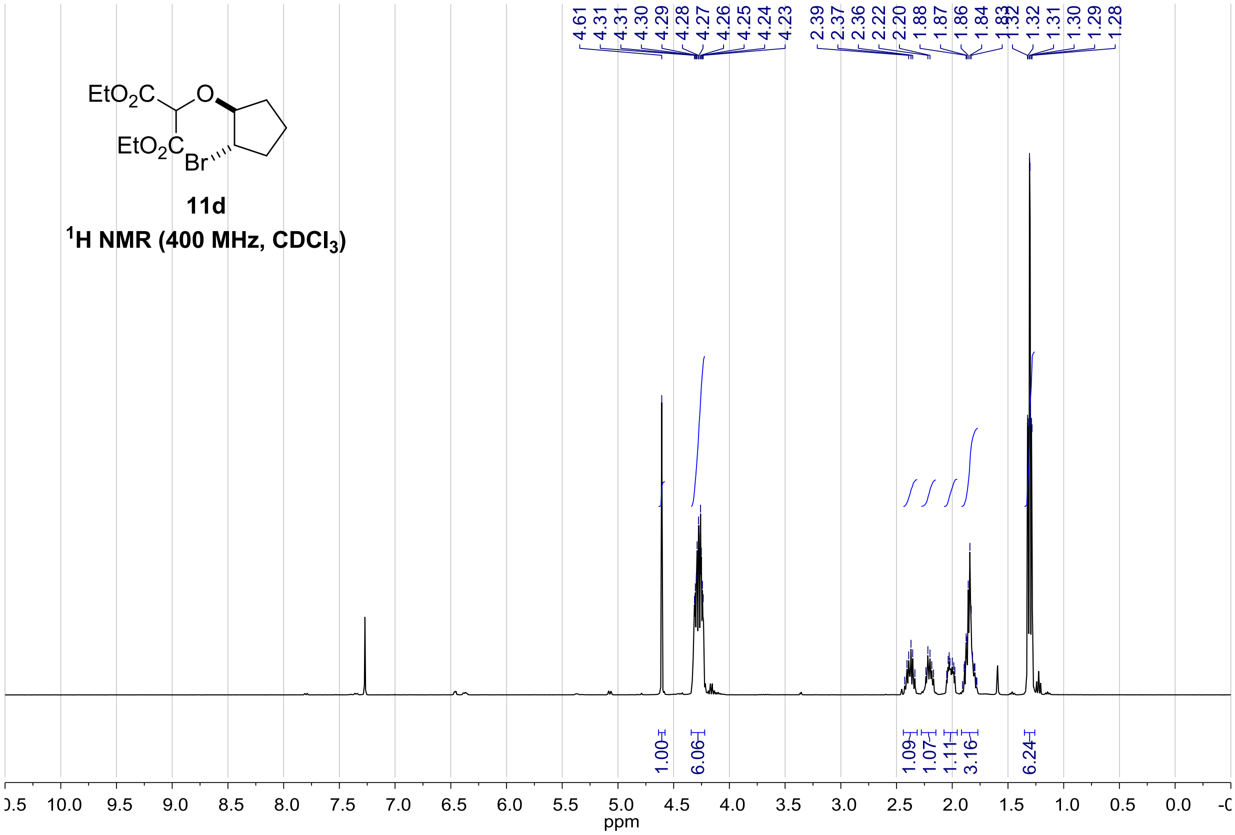

11d

^{1}H NMR (400 MHz, $CDCl_3$)

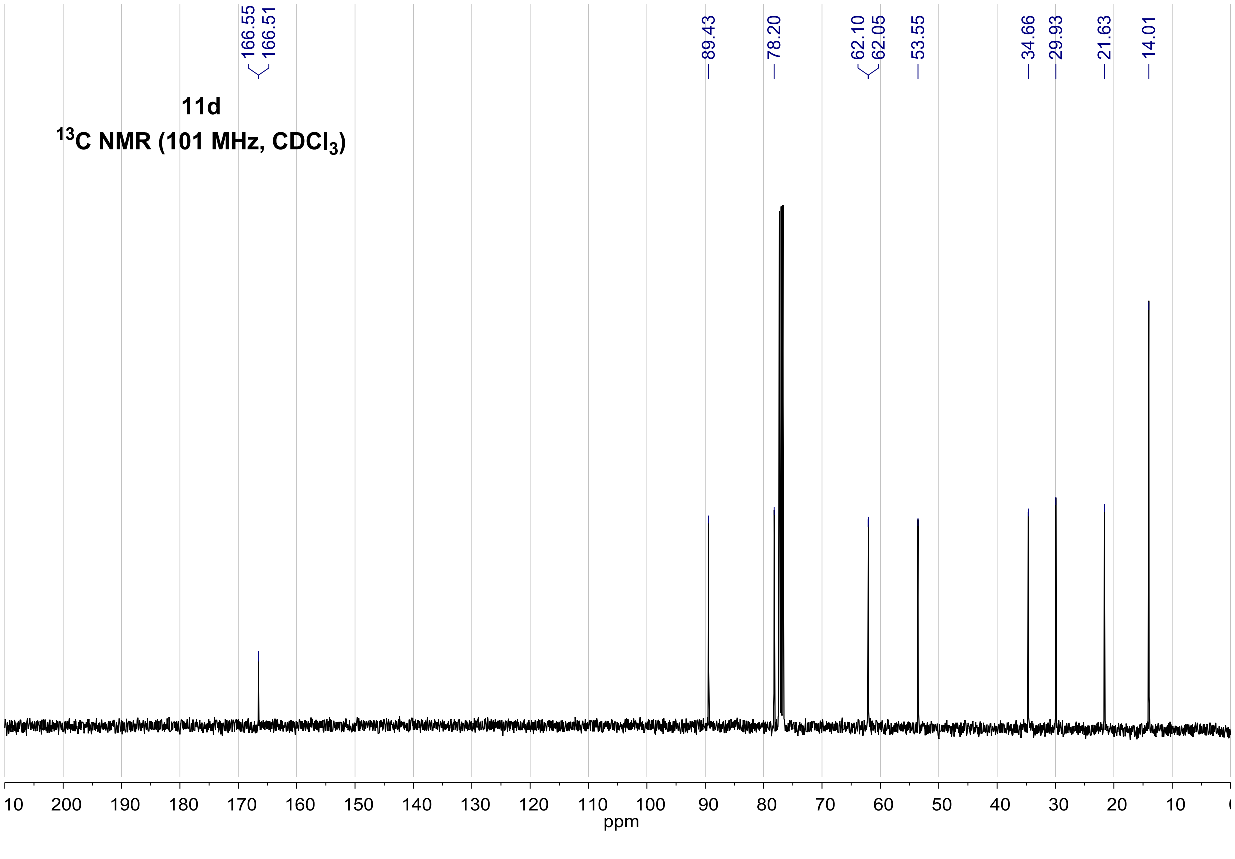

11d

^{13}C NMR (101 MHz, $CDCl_3$)

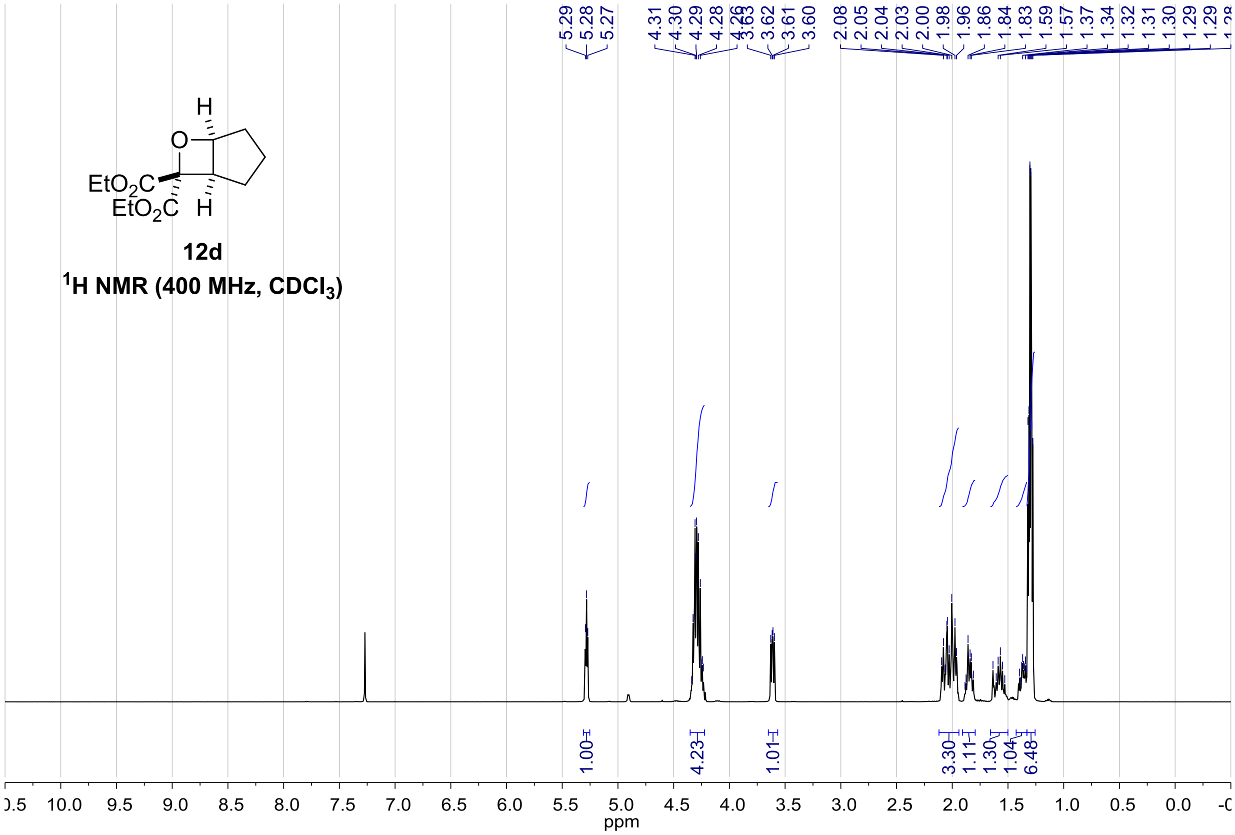
EtO2C
EtO2C
O
H
H
12d
1H NMR (400 MHz, CDCl3)
5.29 5.28 5.27
4.31 4.30 4.29 4.28
3.62 3.61 3.60
2.08 2.05 2.04 2.03 2.00 1.98 1.96 1.86 1.84 1.83 1.59 1.57 1.37 1.34 1.32 1.31 1.30 1.29 1.29
1.00 4.23 1.01 3.30 1.11 1.30 1.04 6.48
ppm

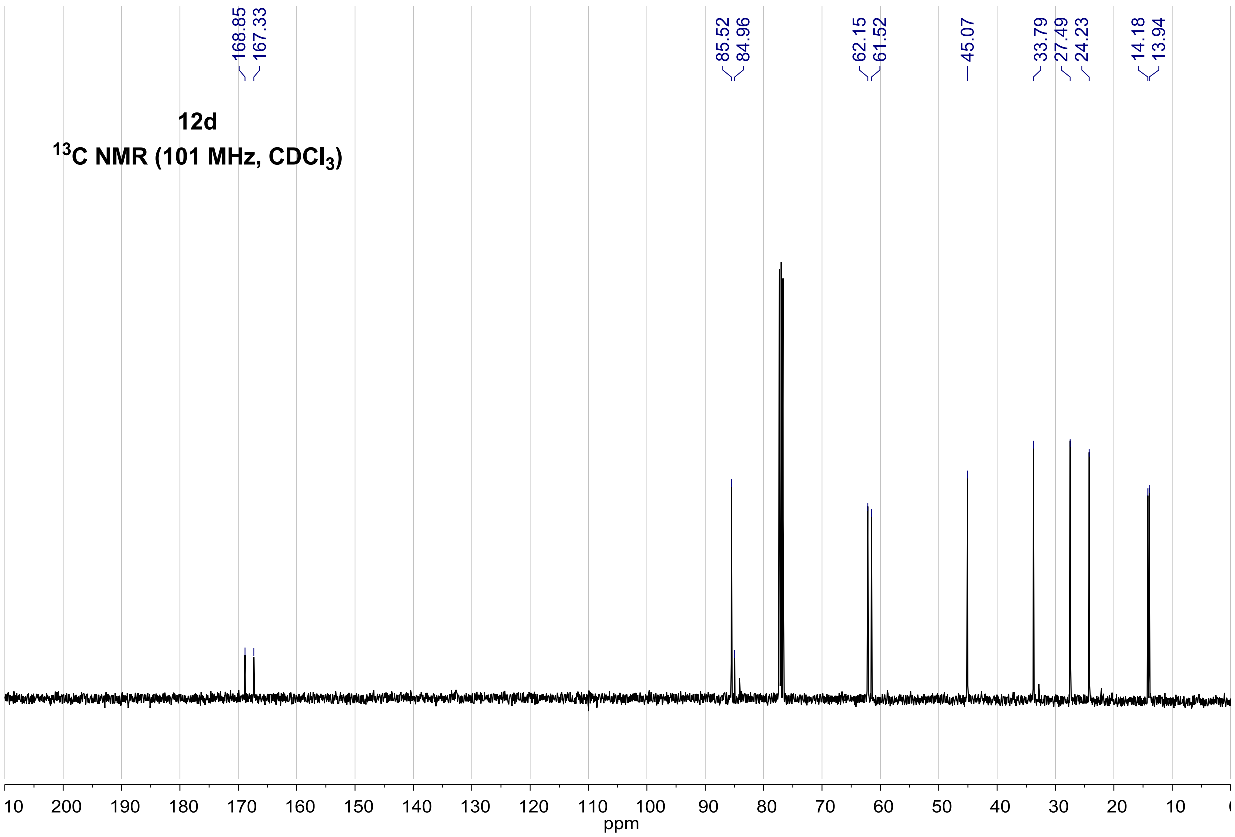
168.85
167.33
85.52
84.96
62.15
61.52
45.07
33.79
27.49
24.23
14.18
13.94
12d
13C NMR (101 MHz, CDCl3)
ppm

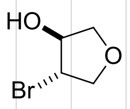

10e
^{1}H NMR (400 MHz, CDCl$_3$)

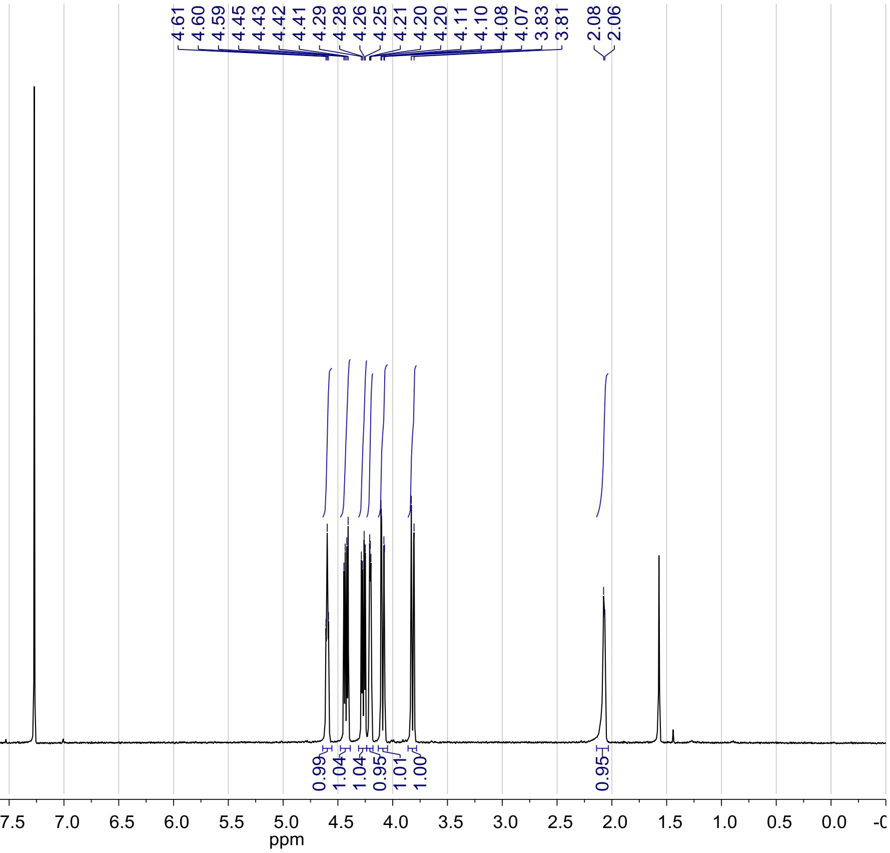

10e
^{13}C NMR (101 MHz, CDCl$_3$)

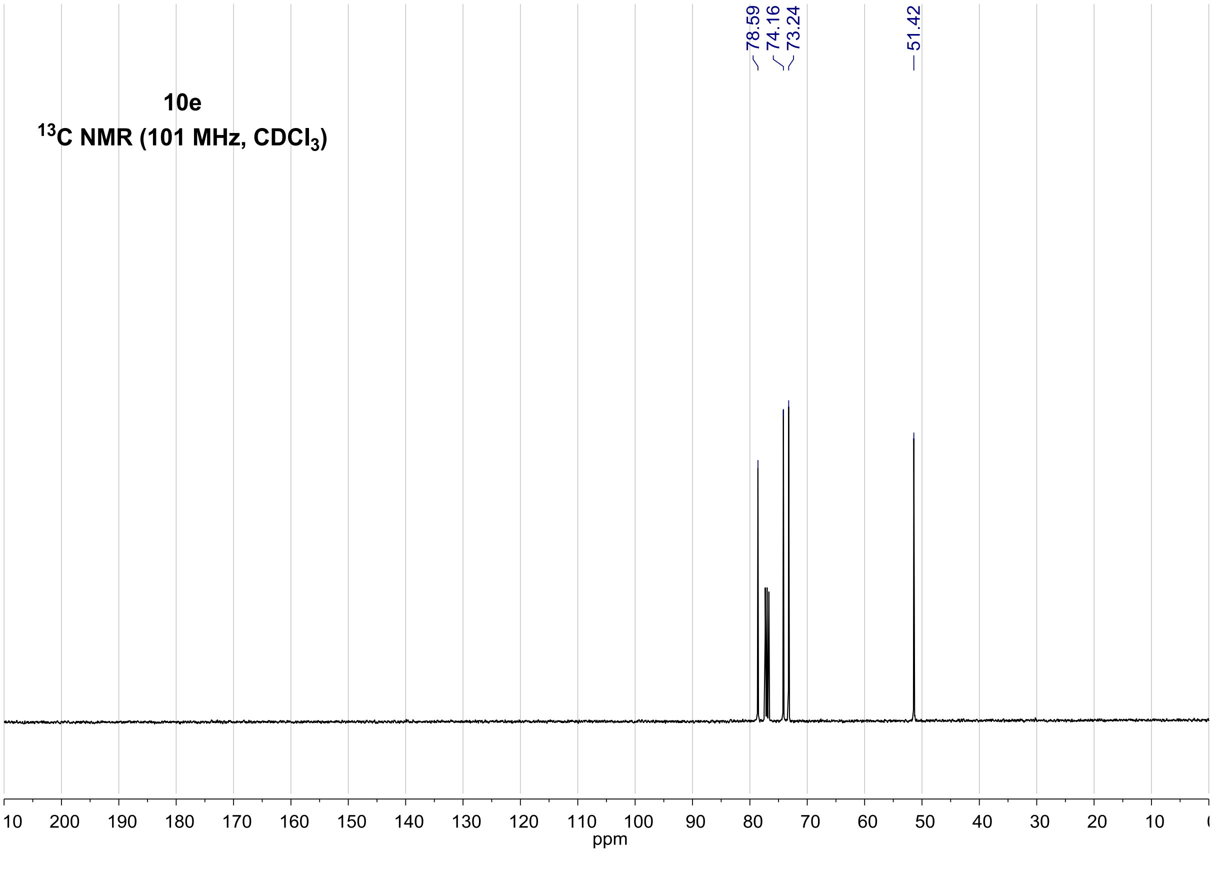

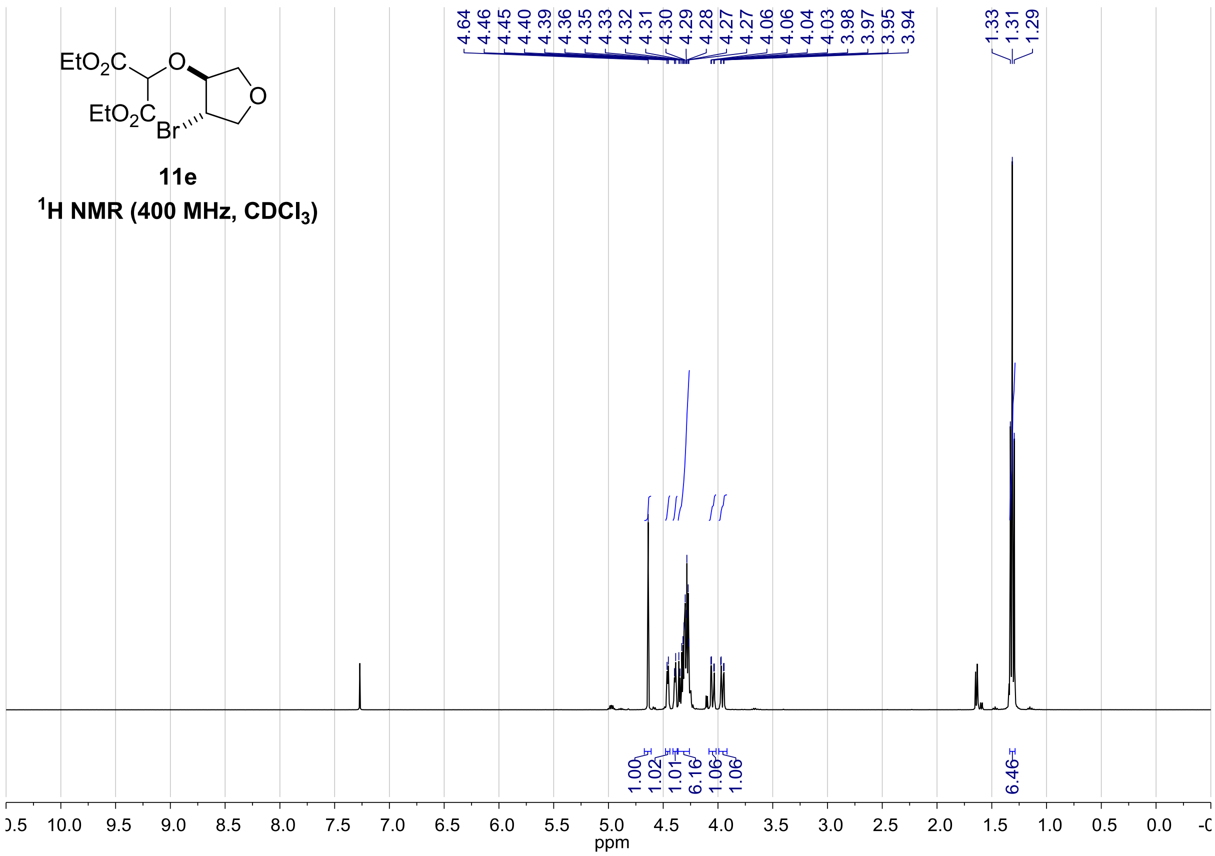
11e
^{1}H NMR (400 MHz, $CDCl_3$)
4.64 4.46 4.45 4.40 4.39 4.36 4.35 4.33 4.32 4.31 4.30 4.29 4.28 4.27 4.27 4.06 4.06 4.04 4.03 3.98 3.97 3.95 3.94
1.33 1.31 1.29
1.00 1.02 1.01 6.16 1.06 1.06 6.46
ppm

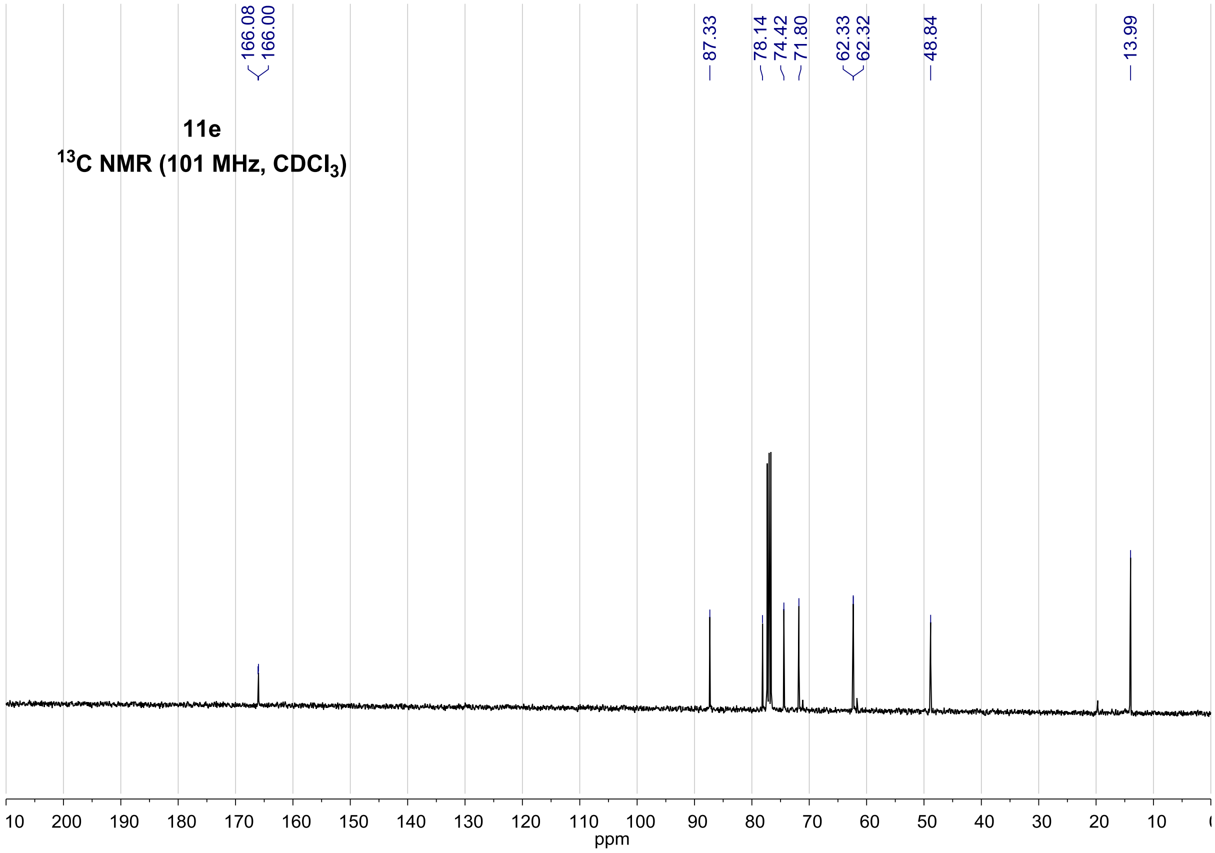
11e
^{13}C NMR (101 MHz, $CDCl_3$)
166.08 166.00 87.33 78.14 74.42 71.80 62.33 62.32 48.84 13.99
ppm

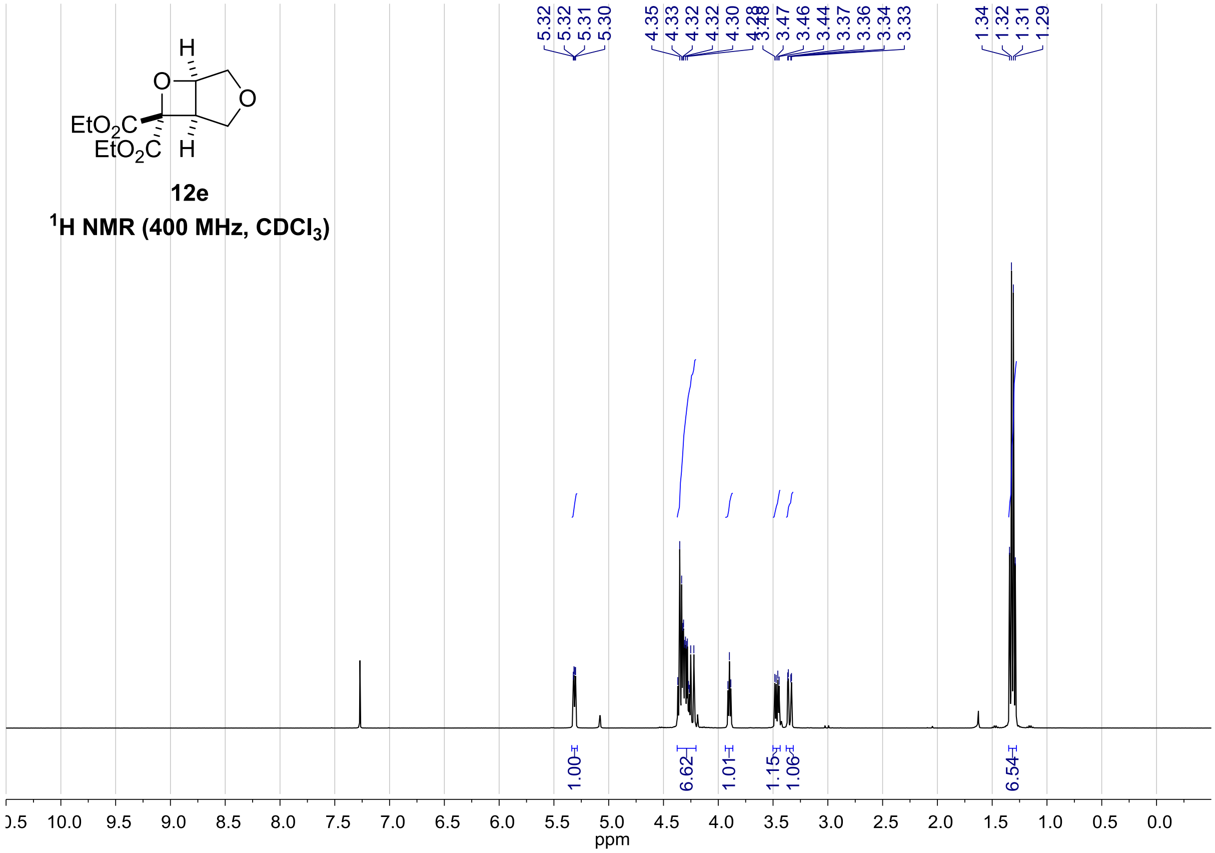

12e

^{1}H NMR (400 MHz, $CDCl_3$)

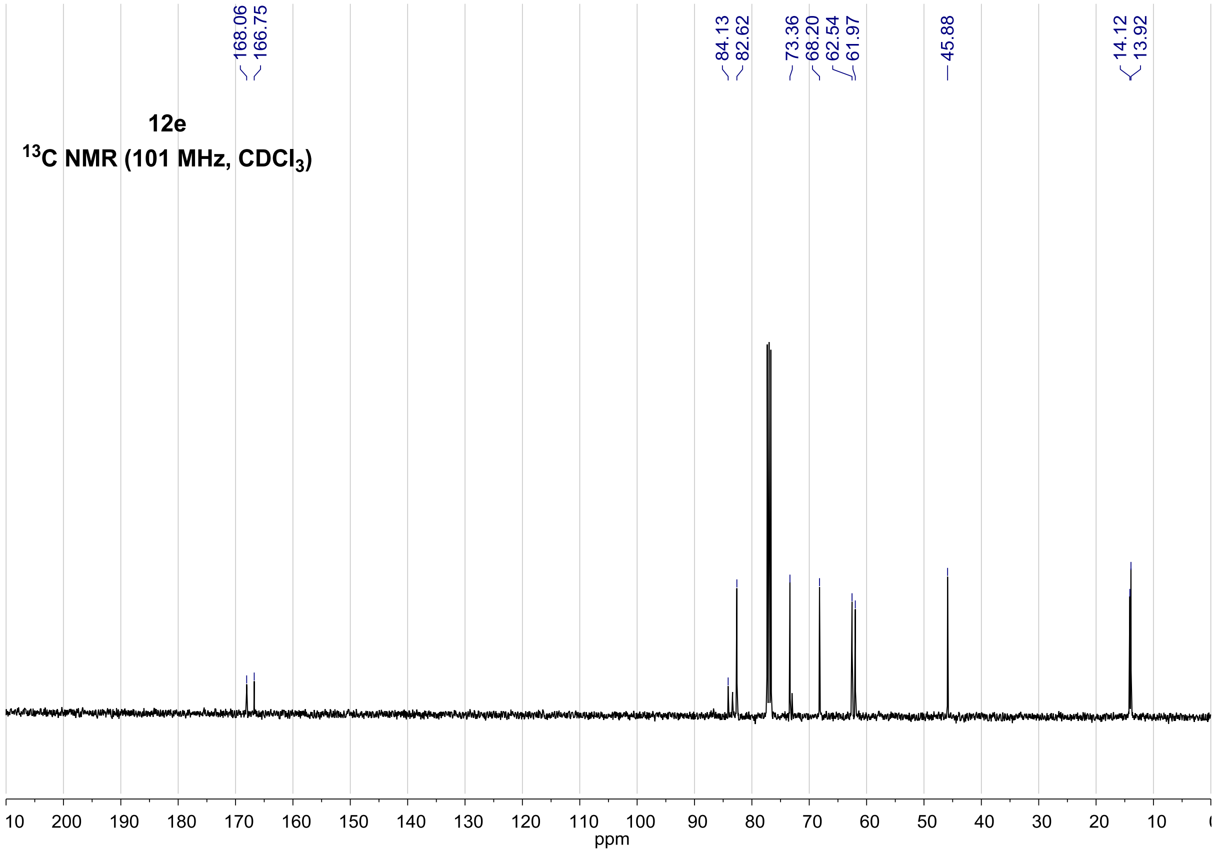

12e

^{13}C NMR (101 MHz, $CDCl_3$)

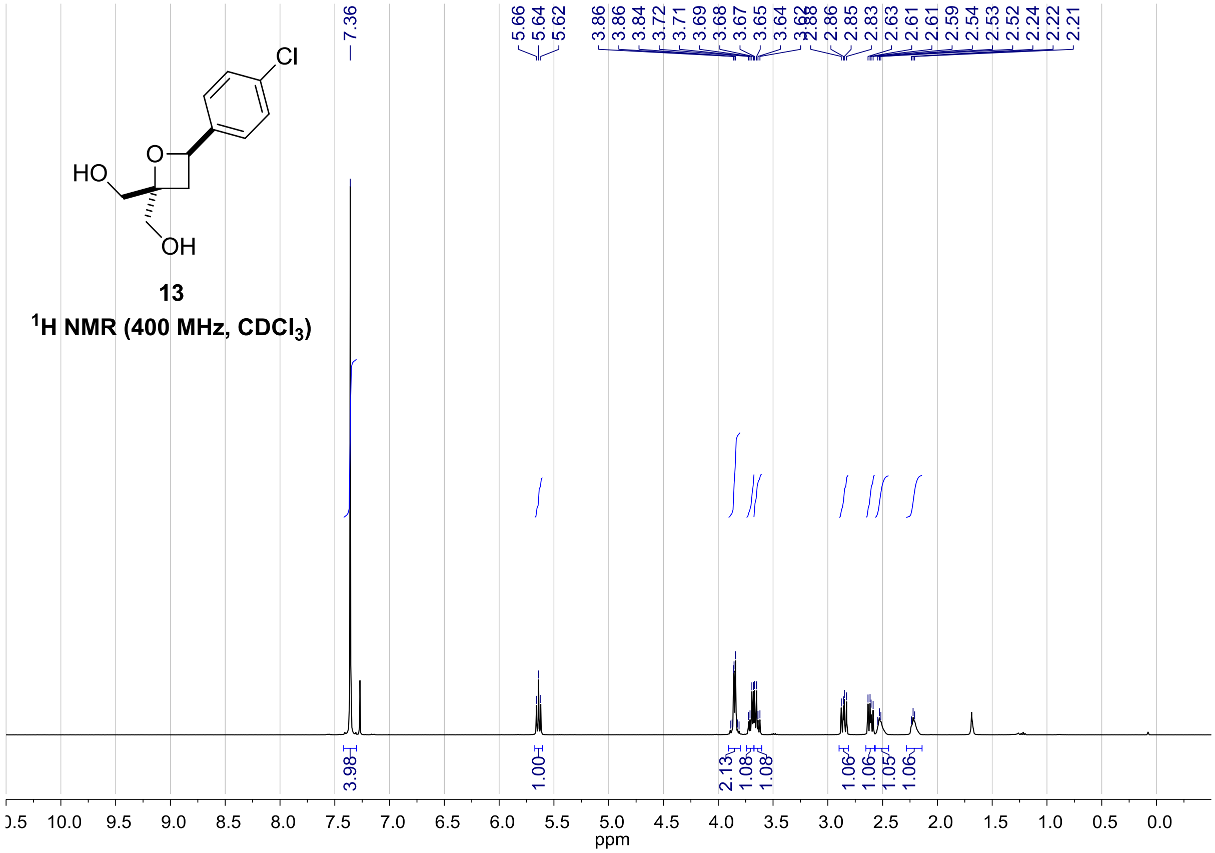
Cl
HO
O
OH
13
^{1}H NMR (400 MHz, $CDCl_3$)
7.36
5.66 5.64 5.62
3.86 3.86 3.84 3.72 3.71 3.69 3.68 3.67 3.65 3.64 3.62 2.88 2.86 2.85 2.83 2.63 2.61 2.61 2.59 2.54 2.53 2.52 2.24 2.22 2.21
3.98 1.00 2.13 1.08 1.08 1.06 1.06 1.05 1.06
ppm

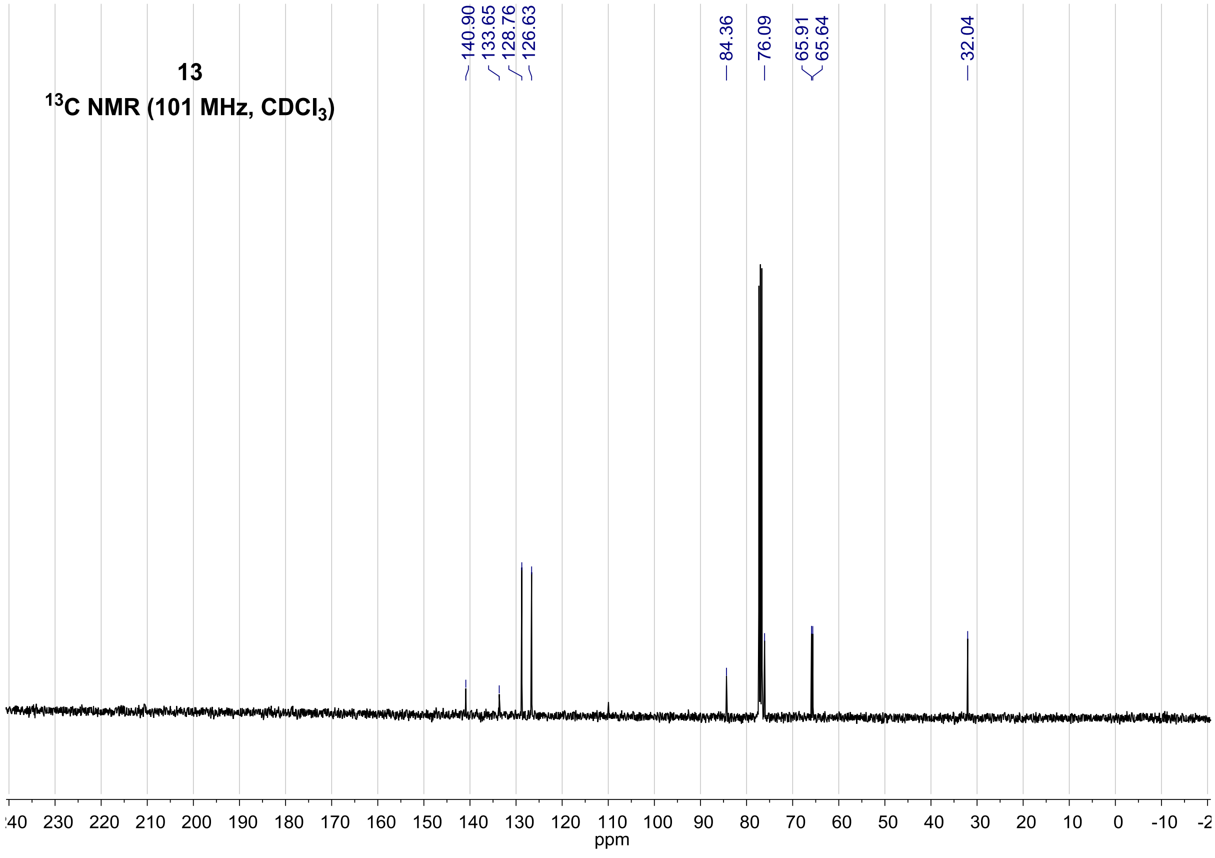
13
^{13}C NMR (101 MHz, $CDCl_3$)
140.90 133.65 128.76 126.63
84.36 76.09 65.91 65.64
32.04
ppm

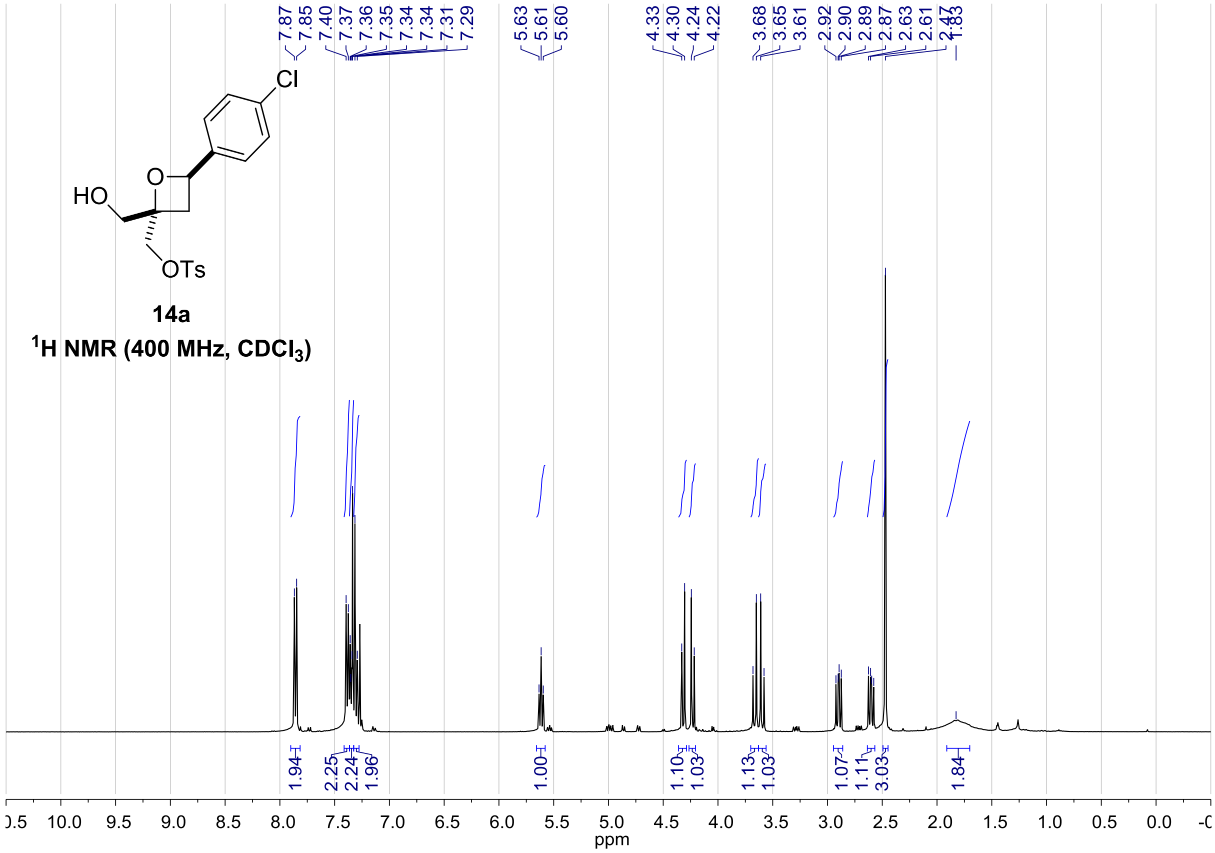
14a
^{1}H NMR (400 MHz, $CDCl_3$)
HO
O
Cl
OTs
7.87 7.85 7.40 7.37 7.36 7.35 7.34 7.34 7.31 7.29 5.63 5.61 5.60 4.33 4.30 4.24 4.22 3.68 3.65 3.61 2.92 2.90 2.89 2.87 2.63 2.61 2.47 1.83
1.94 2.25 2.24 1.96 1.00 1.10 1.03 1.13 1.03 1.07 1.11 3.03 1.84
ppm

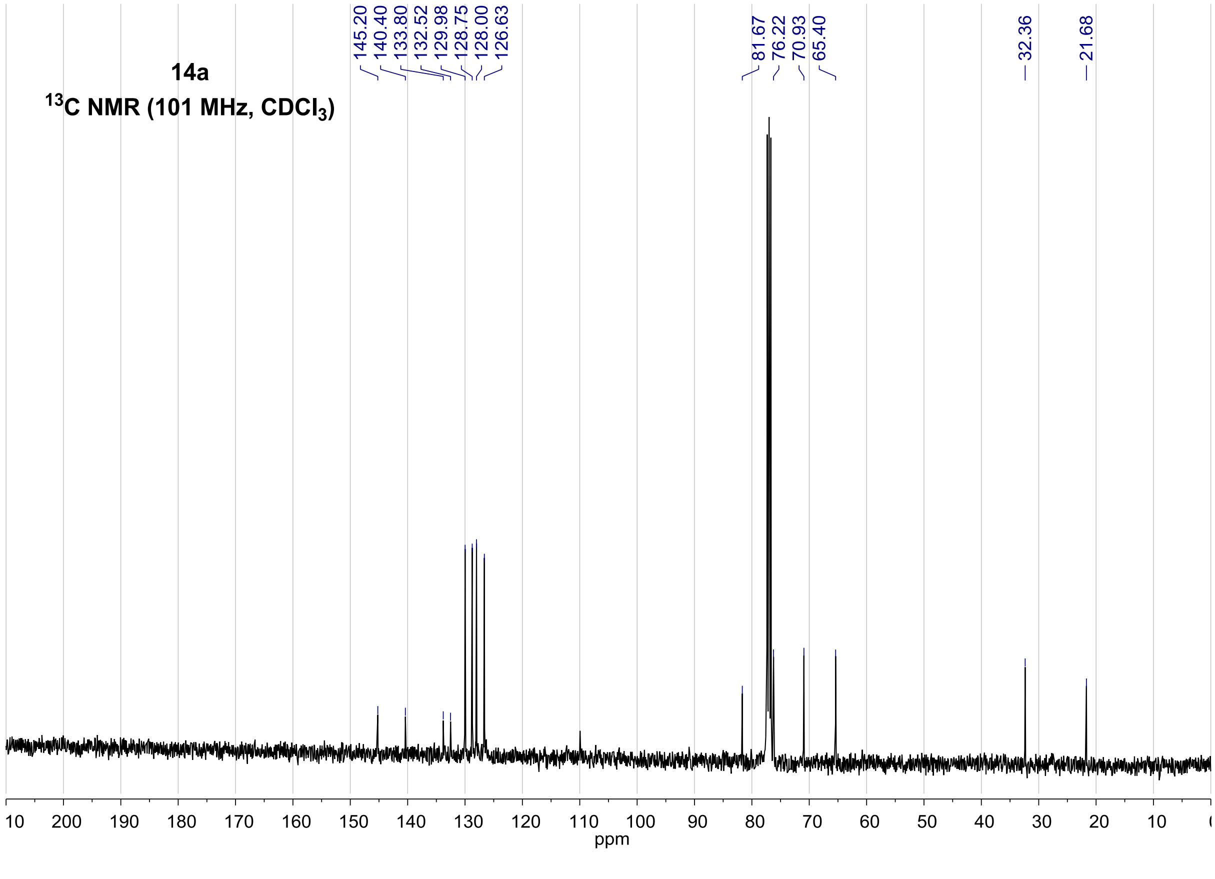
14a
^{13}C NMR (101 MHz, $CDCl_3$)
145.20 140.40 133.80 132.52 129.98 128.75 128.00 126.63 81.67 76.22 70.93 65.40 32.36 21.68
ppm

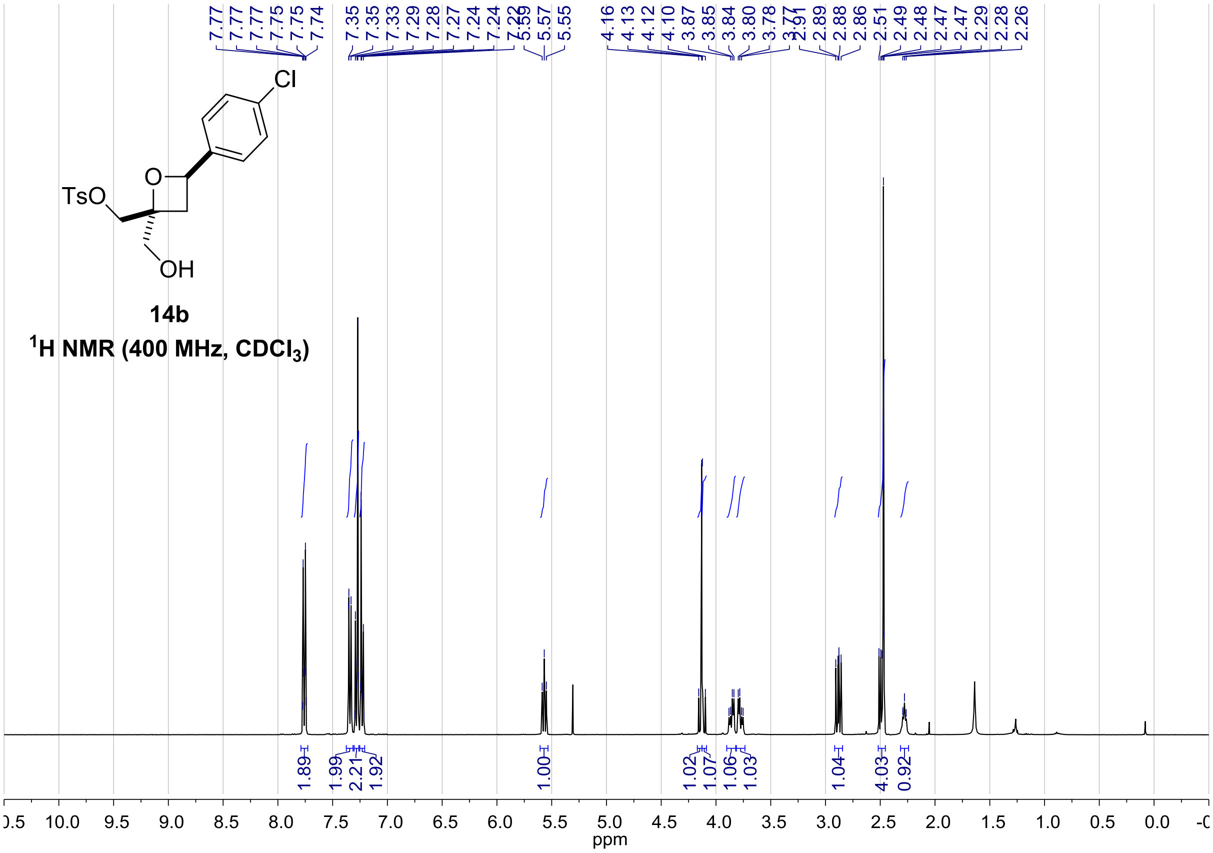
7.77 7.77 7.77 7.75 7.75 7.74 7.35 7.35 7.33 7.29 7.28 7.27 7.24 7.24 7.22 5.59 5.57 5.55 4.16 4.13 4.12 4.10 3.87 3.85 3.84 3.80 3.78 3.77 2.91 2.89 2.88 2.86 2.51 2.49 2.48 2.47 2.47 2.29 2.28 2.26
Cl
TsO
O
OH
14b
^{1}H NMR (400 MHz, CDCl$_3$)
1.89 1.99 2.21 1.92 1.00 1.02 1.07 1.06 1.03 1.04 4.03 0.92
10.0 9.5 9.0 8.5 8.0 7.5 7.0 6.5 6.0 5.5 5.0 4.5 4.0 3.5 3.0 2.5 2.0 1.5 1.0 0.5 0.0
ppm

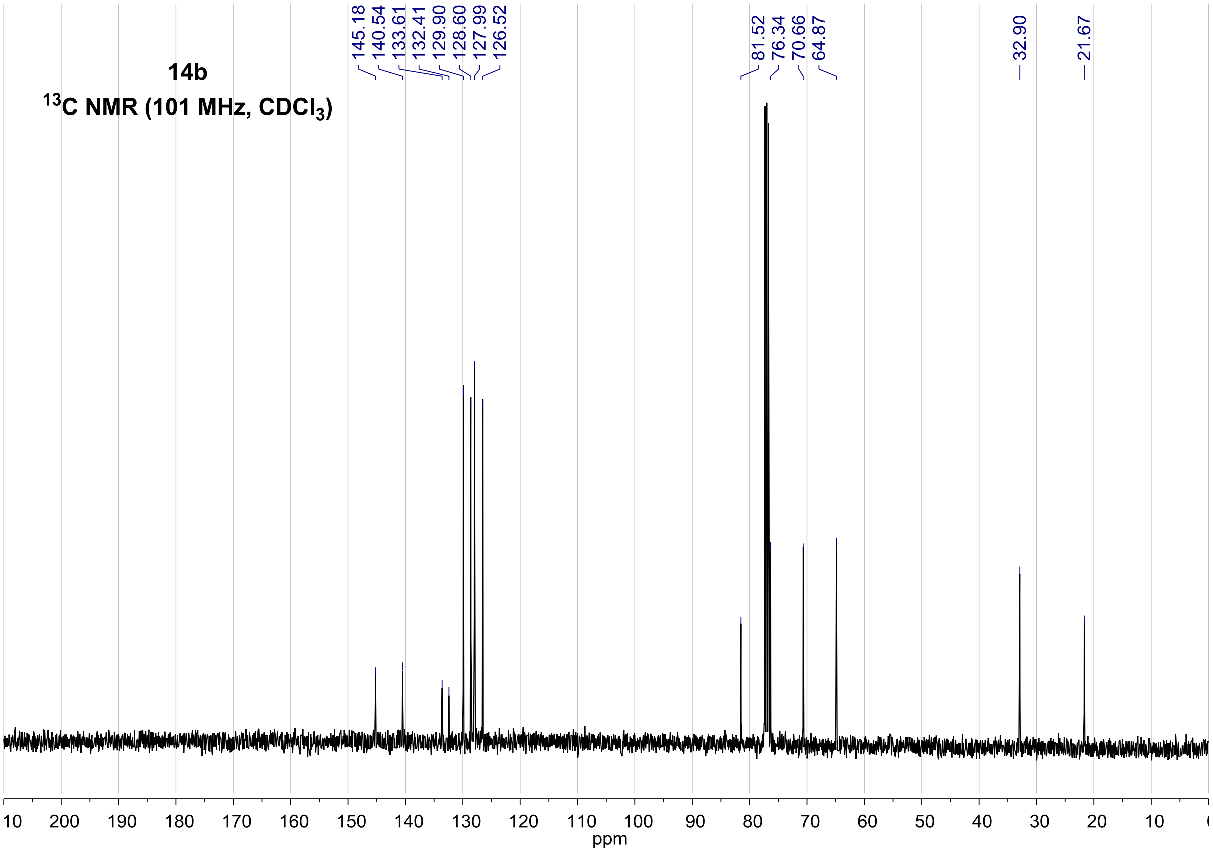
145.18 140.54 133.61 132.41 129.90 128.60 127.99 126.52
81.52 76.34 70.66 64.87
32.90
21.67
14b
^{13}C NMR (101 MHz, CDCl$_3$)
200 190 180 170 160 150 140 130 120 110 100 90 80 70 60 50 40 30 20 10
ppm

Selective NOE of minor **14a** – Irradiation at 5.61 ppm

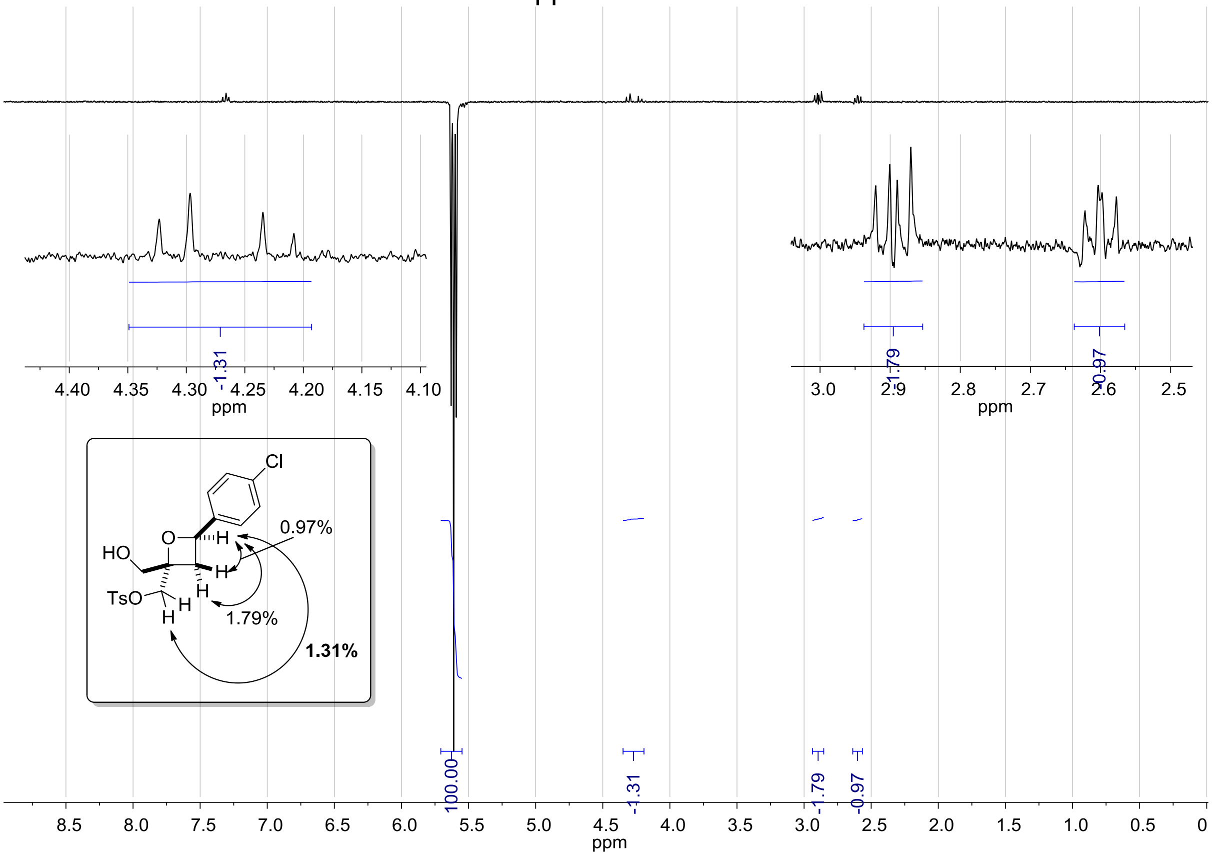

Selective NOE of major **14b** – Irradiation at 5.57 ppm

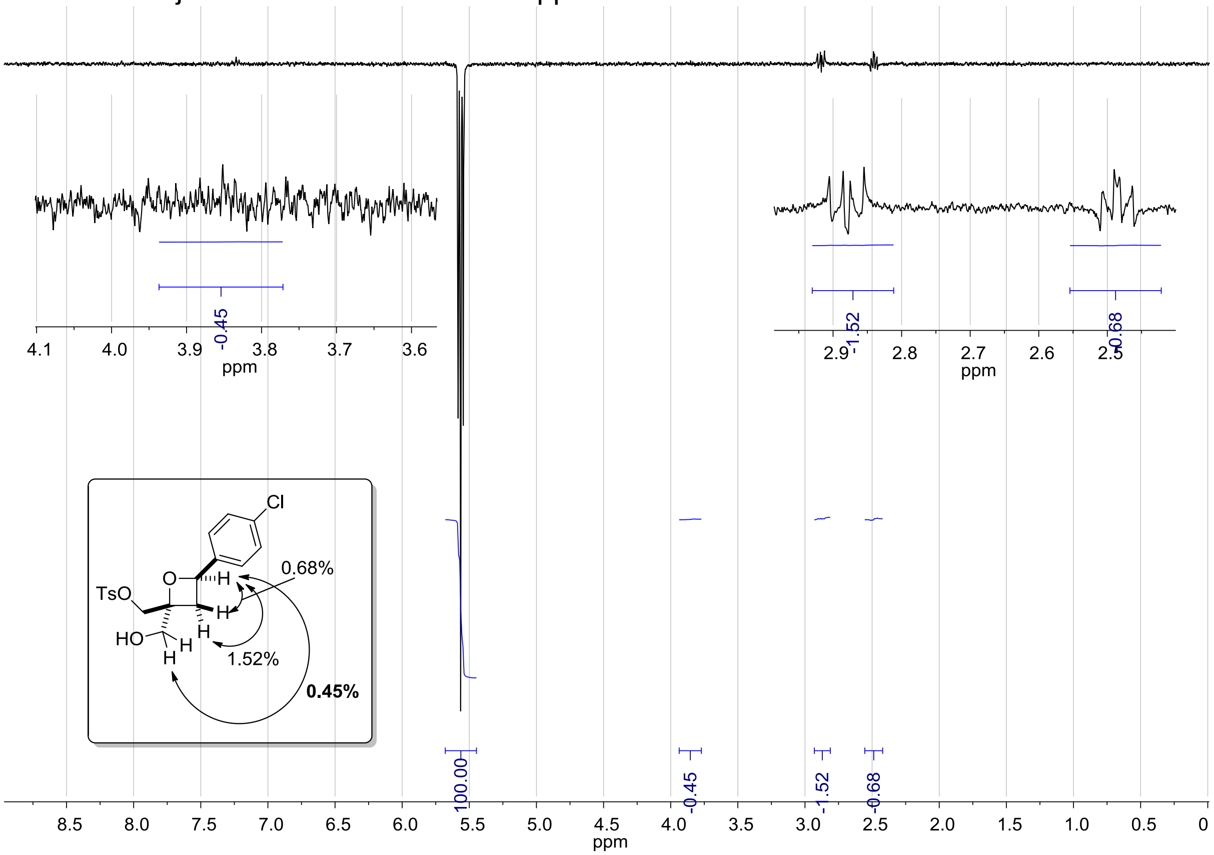

NOESY NMR of major **14b**

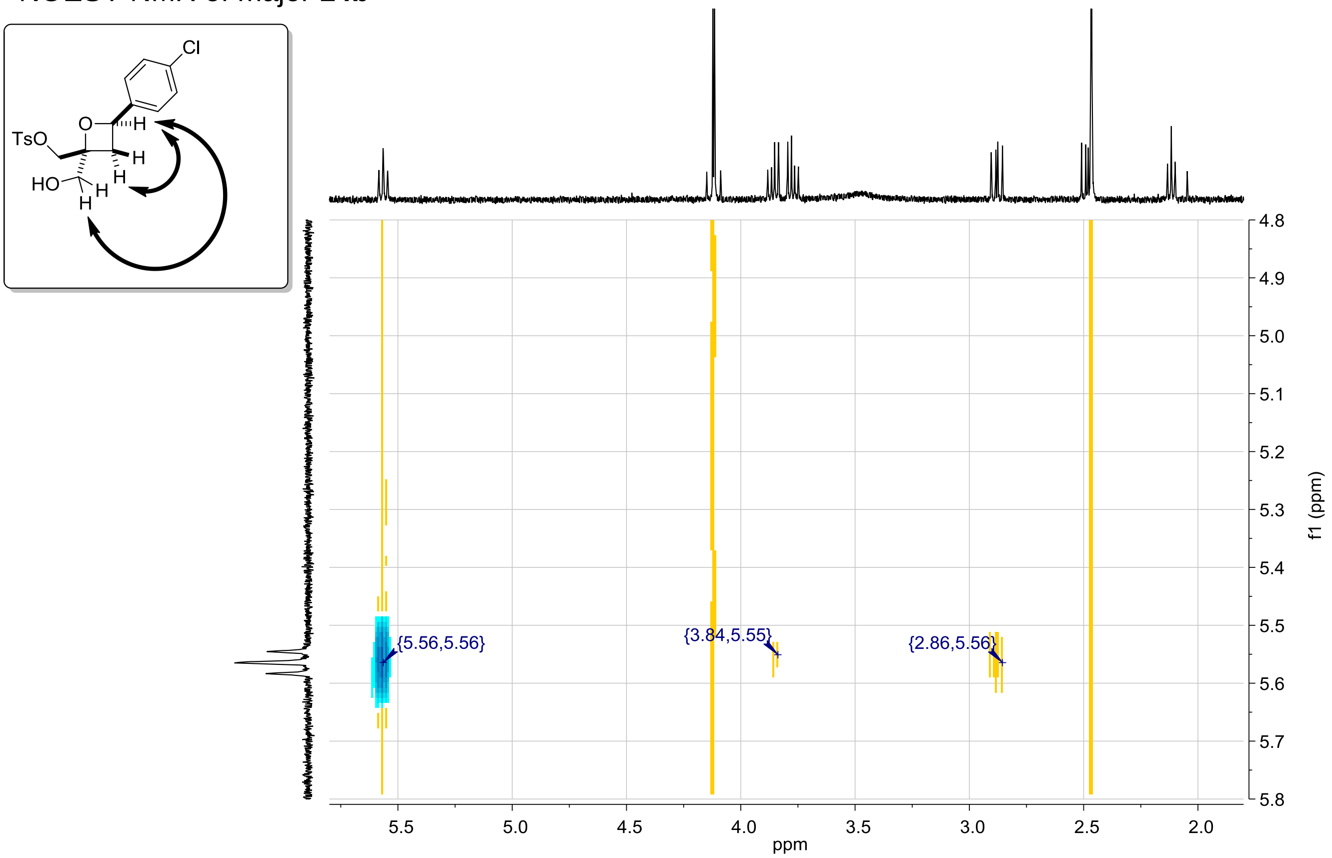

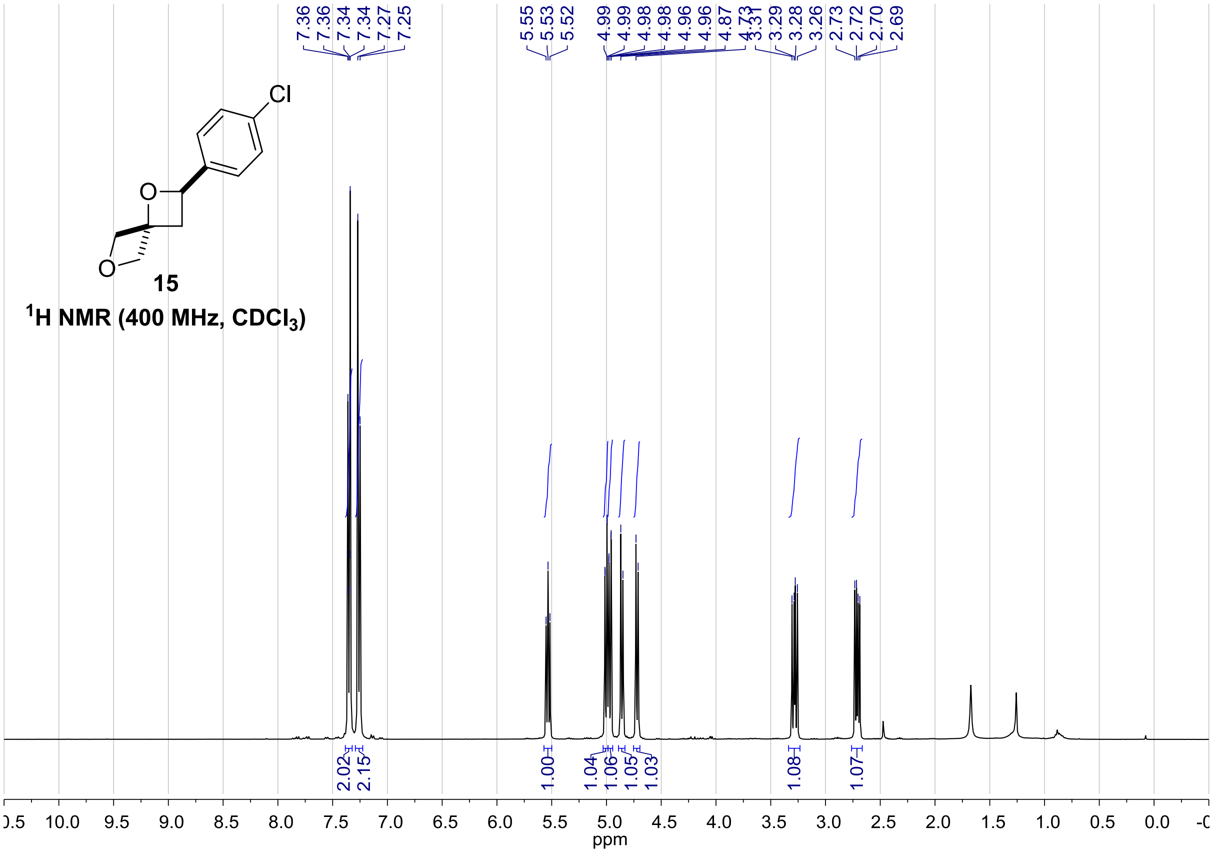

15

^{1}H NMR (400 MHz, $CDCl_3$)

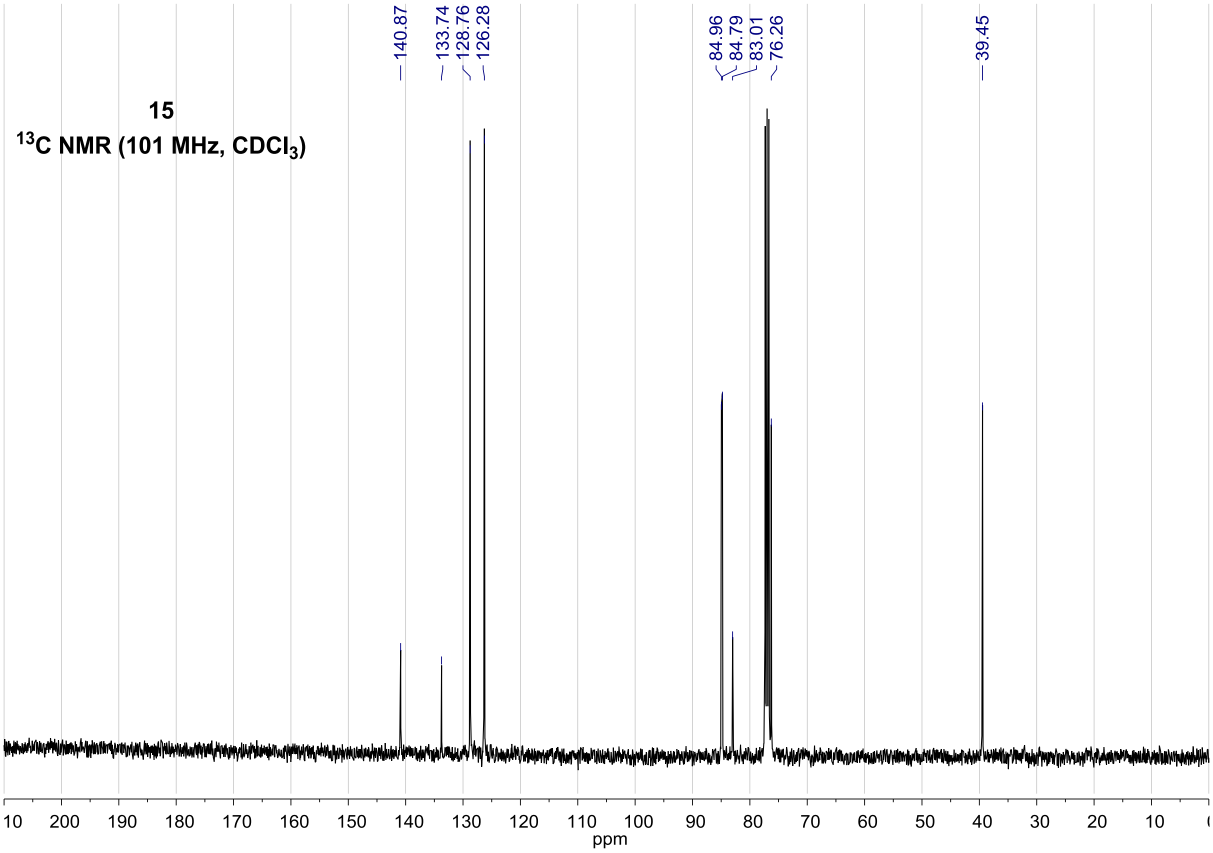

15

^{13}C NMR (101 MHz, $CDCl_3$)

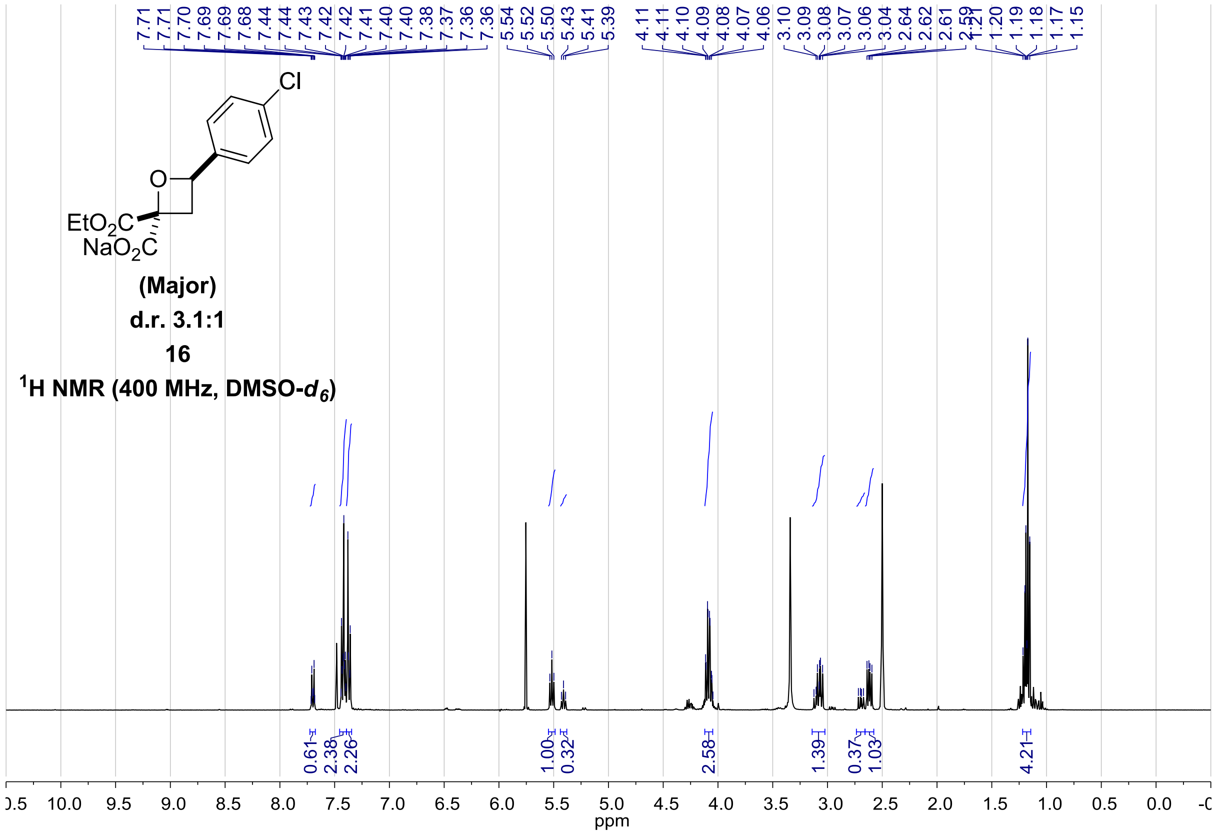
Cl
O
EtO_2C
NaO_2C
(Major)
d.r. 3.1:1
16
^{1}H NMR (400 MHz, DMSO-d_6)
7.71 7.71 7.70 7.69 7.68 7.44 7.44 7.43 7.42 7.42 7.41 7.40 7.40 7.38 7.37 7.36 5.54 5.52 5.50 5.43 5.41 5.39 4.11 4.11 4.10 4.09 4.08 4.07 4.06 3.10 3.09 3.08 3.07 3.06 3.04 2.64 2.62 2.61 2.59 1.21 1.20 1.19 1.18 1.17 1.15
0.61 2.38 2.26 1.00 0.32 2.58 1.39 0.37 1.03 4.21
ppm

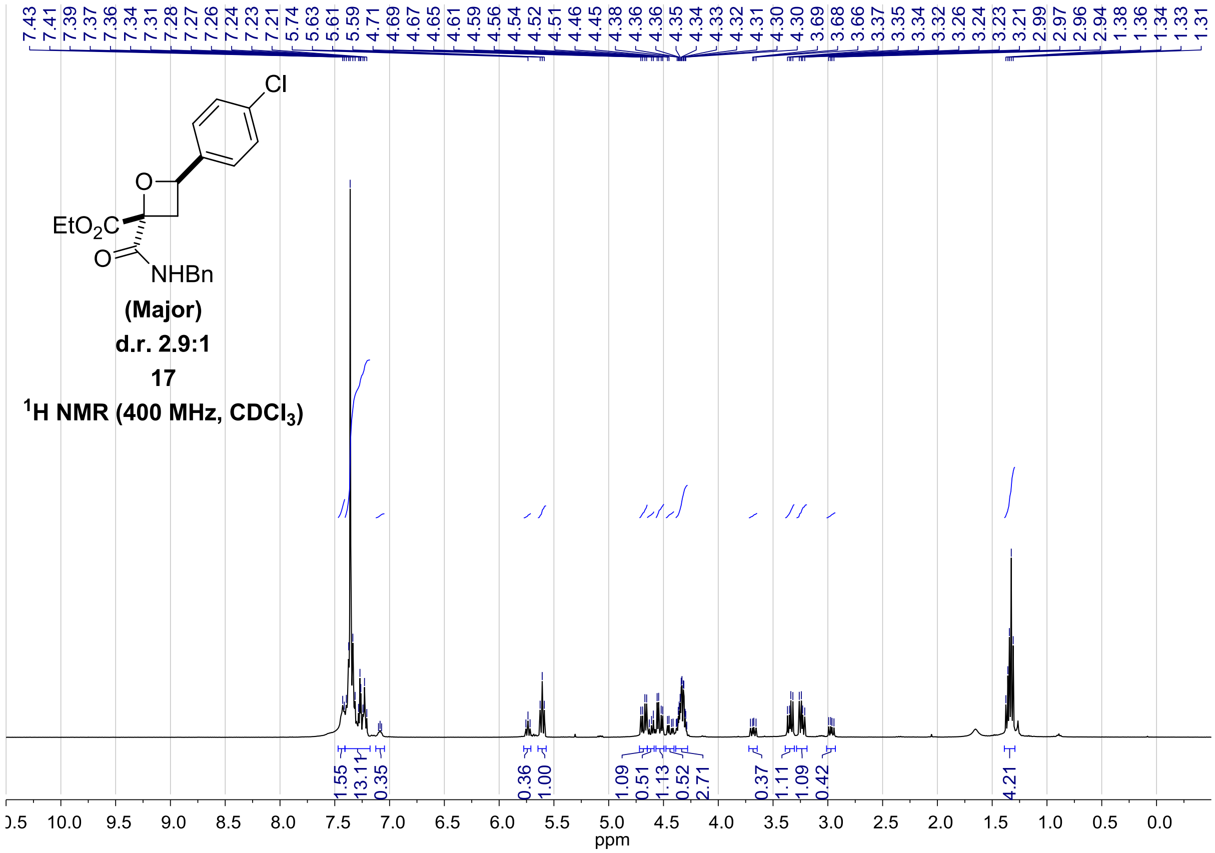
Cl
O
EtO2C
O
NHBn
(Major)
d.r. 2.9:1
17
1H NMR (400 MHz, CDCl3)
7.43 7.41 7.39 7.37 7.36 7.34 7.31 7.28 7.27 7.26 7.24 7.23 7.21 5.74 5.63 5.61 5.59 4.71 4.69 4.67 4.65 4.61 4.59 4.56 4.54 4.52 4.51 4.46 4.45 4.38 4.36 4.36 4.35 4.34 4.33 4.32 4.31 4.30 4.30 3.69 3.68 3.66 3.37 3.35 3.34 3.32 3.26 3.24 3.23 3.21 2.99 2.97 2.96 2.94 1.38 1.36 1.34 1.33 1.31
1.55 13.11 0.35 0.36 1.00 1.09 0.51 1.13 0.52 2.71 0.37 1.11 1.09 0.42 4.21
ppm

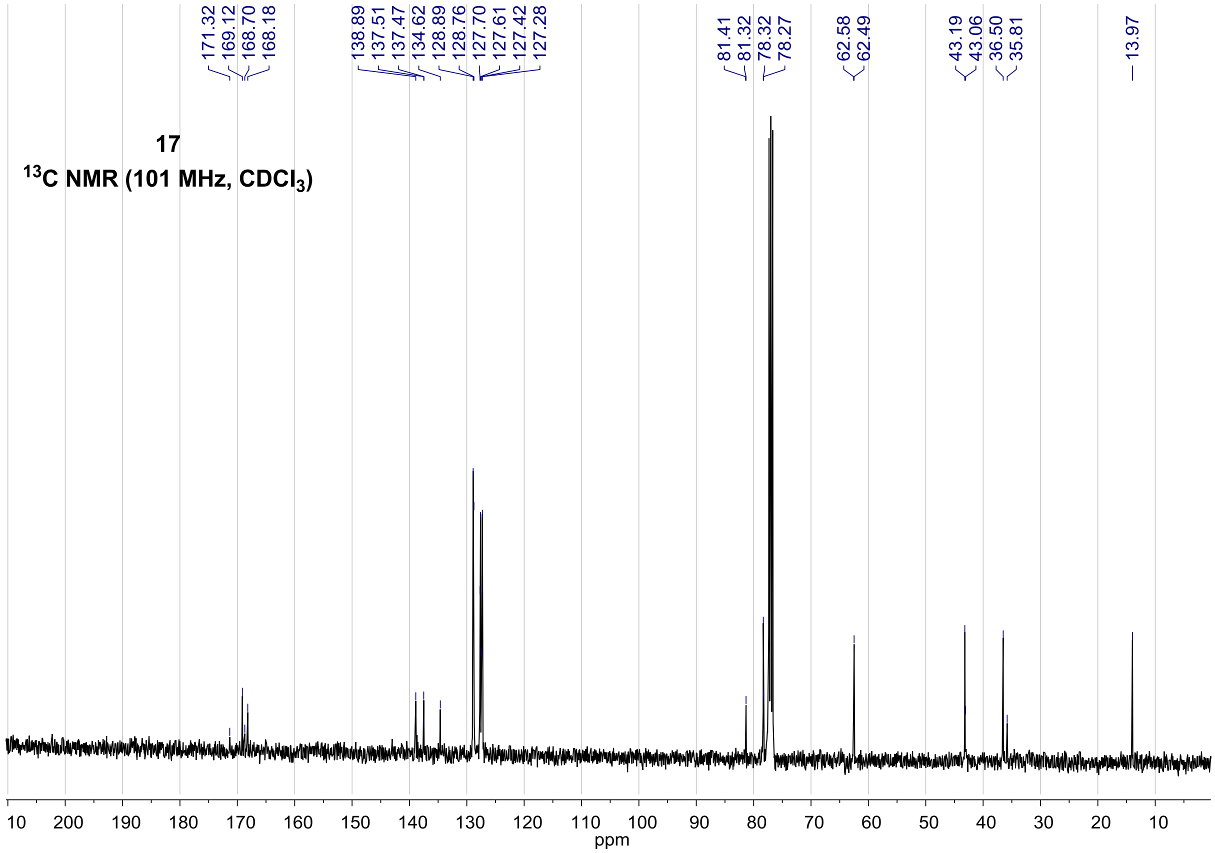
17
13C NMR (101 MHz, CDCl3)
171.32 169.12 168.70 168.18 138.89 137.51 137.47 134.62 128.89 128.76 127.70 127.61 127.42 127.28 81.41 81.32 78.32 78.27 62.58 62.49 43.19 43.06 36.50 35.81 13.97
ppm

Selective NOE of **Major Product 17** – Irradiation at 5.60 ppm

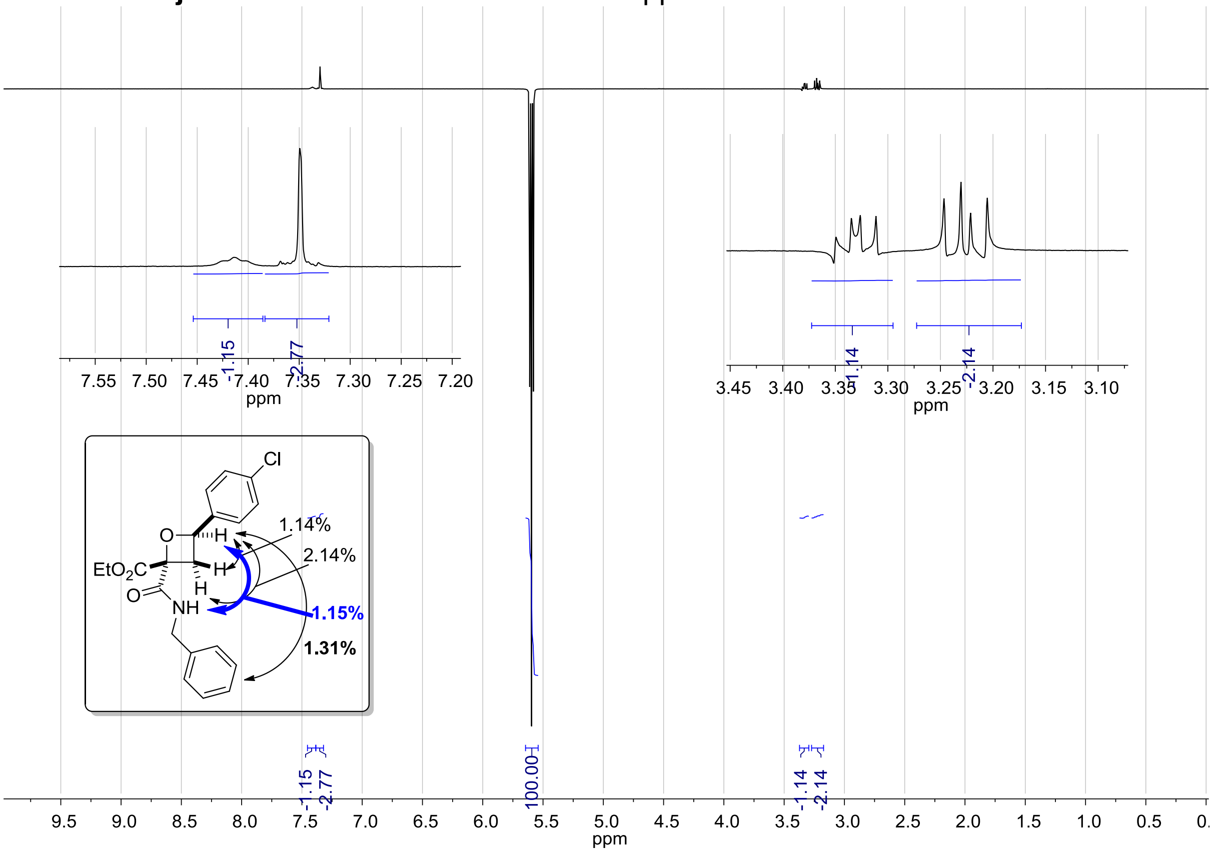

Selective NOE of **Minor Product 19** – Irradiation at 5.73 ppm

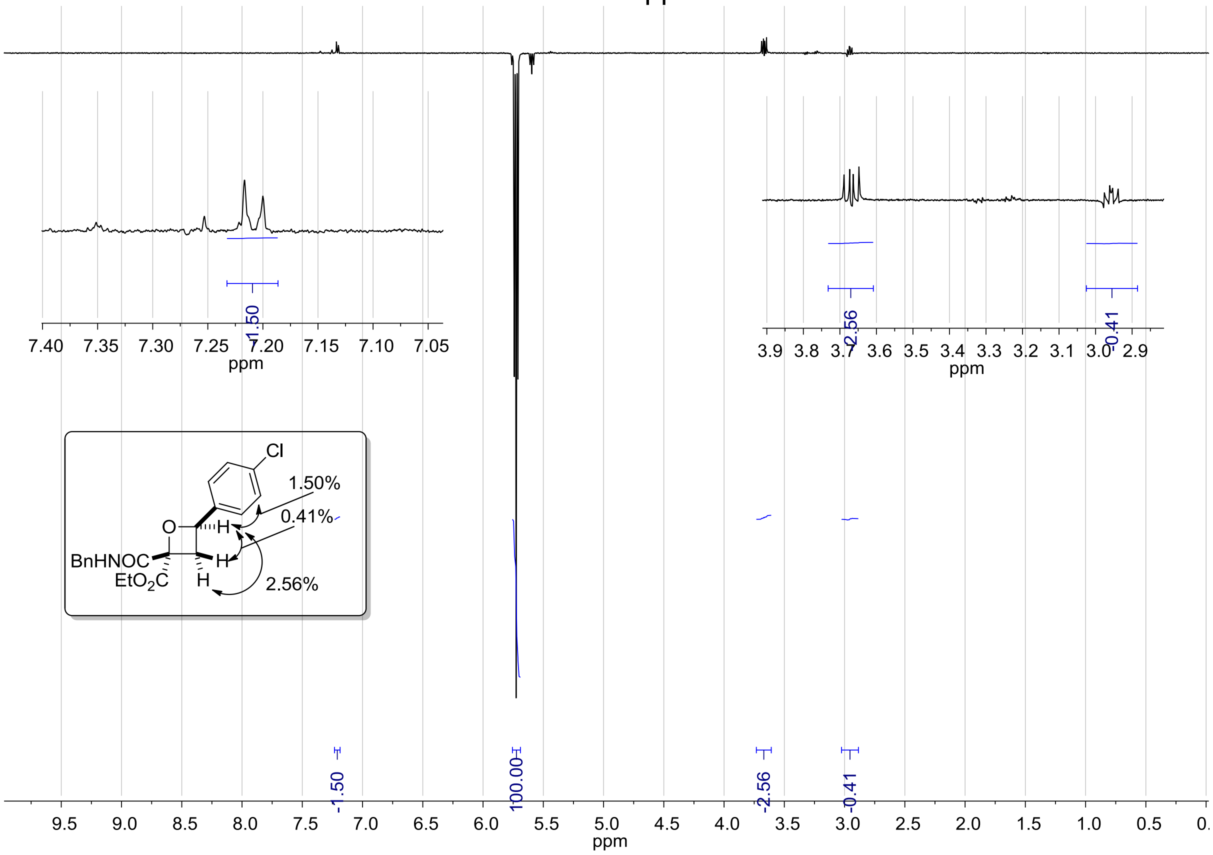

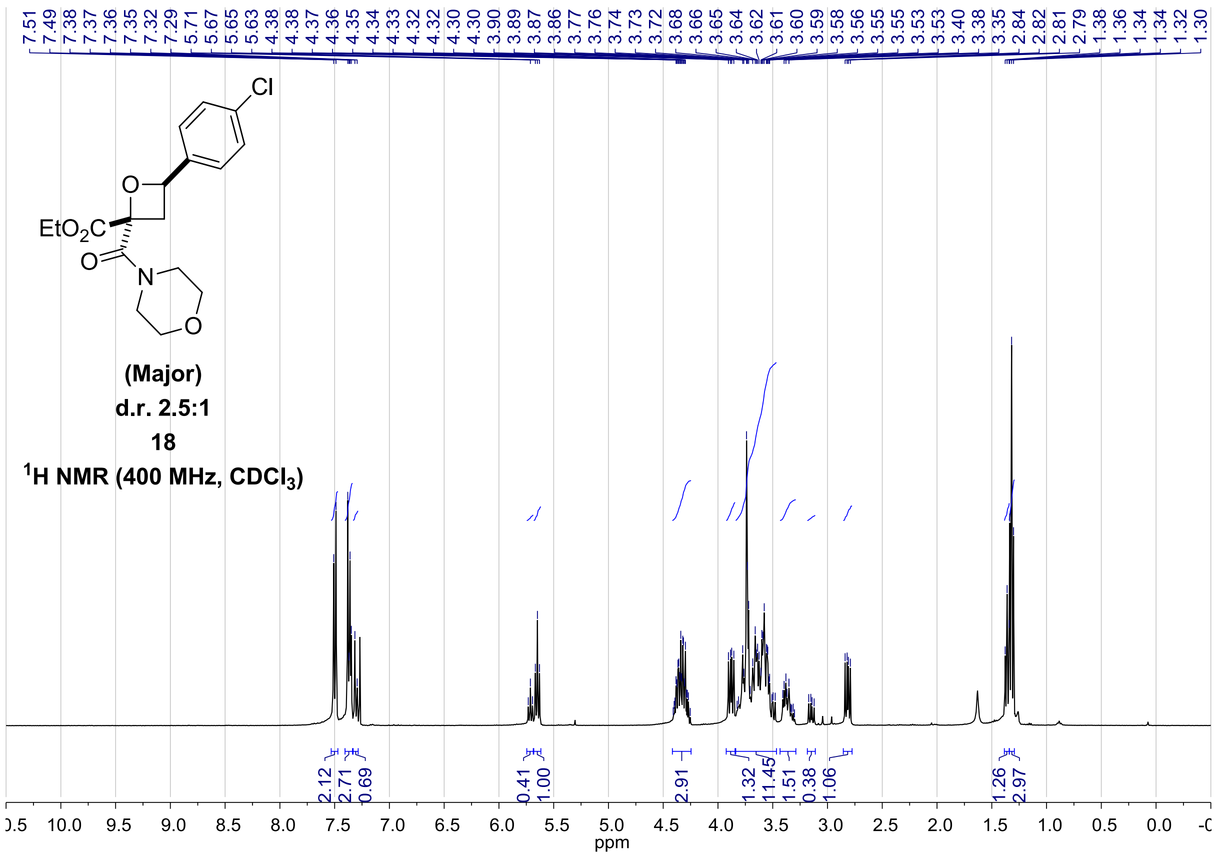

Cl
O
EtO2C
O
N
O
(Major)
d.r. 2.5:1
18
1H NMR (400 MHz, CDCl3)
7.51 7.49 7.38 7.37 7.36 7.35 7.32 7.29 5.71 5.67 5.65 5.63 4.38 4.38 4.37 4.36 4.35 4.34 4.33 4.32 4.32 4.30 4.30 3.90 3.89 3.87 3.86 3.77 3.76 3.74 3.73 3.72 3.68 3.66 3.65 3.64 3.62 3.61 3.60 3.59 3.58 3.56 3.55 3.55 3.53 3.53 3.40 3.38 3.35 2.84 2.82 2.81 2.79 1.38 1.36 1.34 1.34 1.32 1.30
2.12 2.71 0.69 0.41 1.00 2.91 1.32 11.45 1.51 0.38 1.06 1.26 2.97
ppm

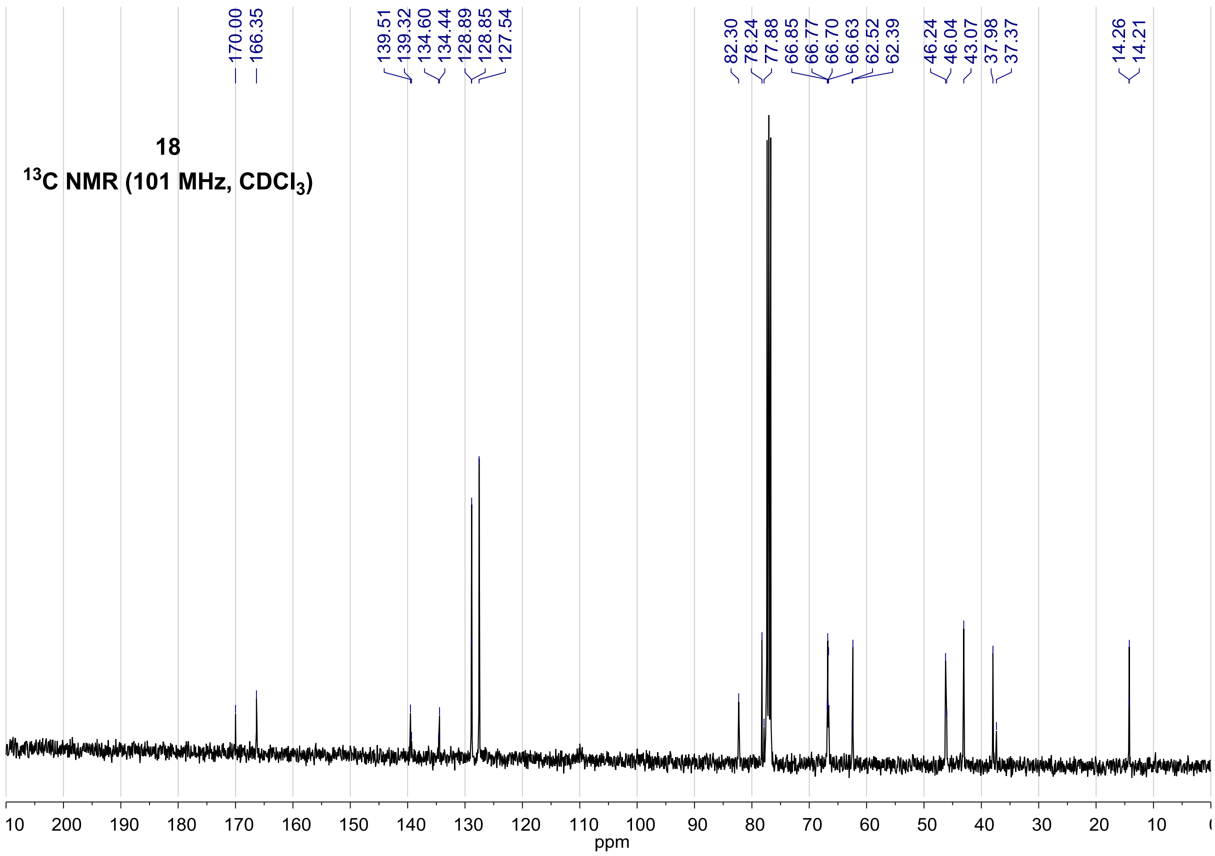

18
13C NMR (101 MHz, CDCl3)
170.00 166.35 139.51 139.32 134.60 134.44 128.89 128.85 127.54 82.30 78.24 77.88 66.85 66.77 66.70 66.63 62.52 62.39 46.24 46.04 43.07 37.98 37.37 14.26 14.21
ppm

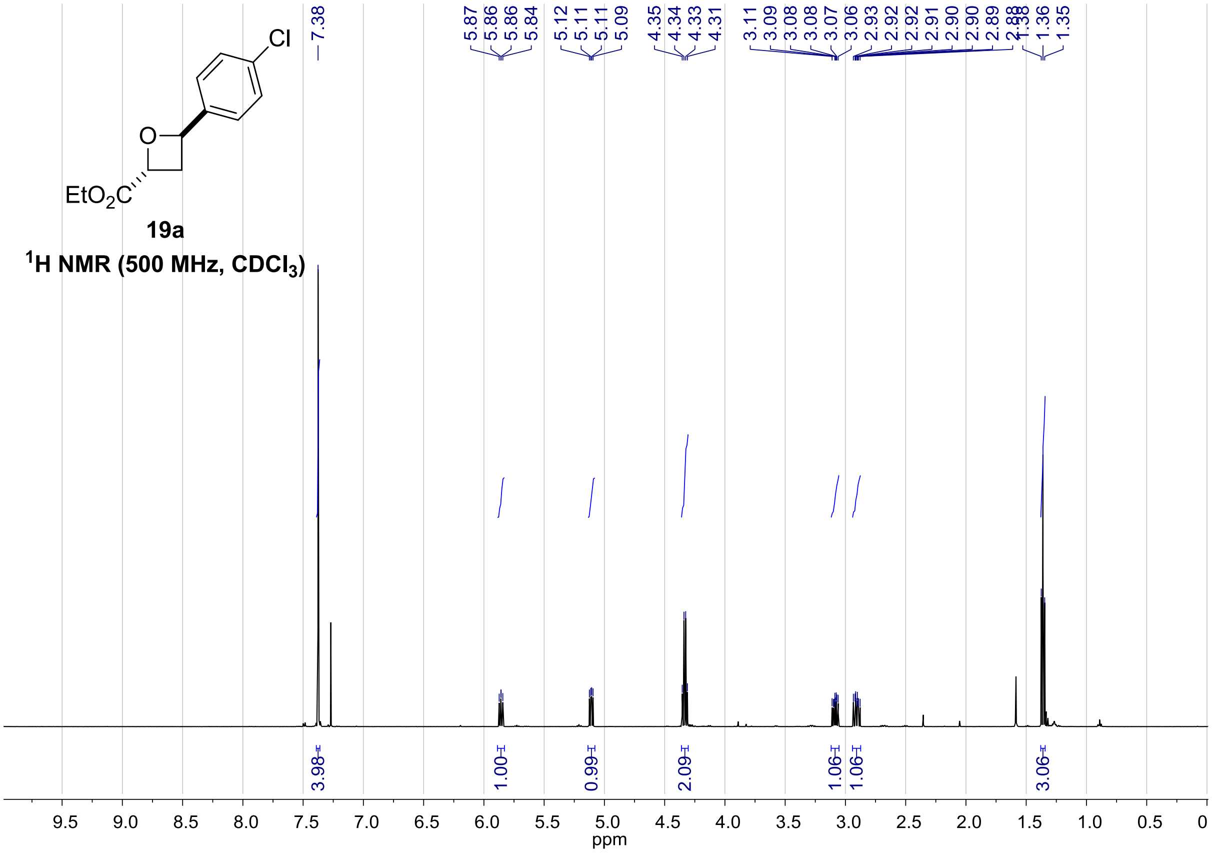
EtO2C
Cl
O
19a
1H NMR (500 MHz, CDCl3)
ppm

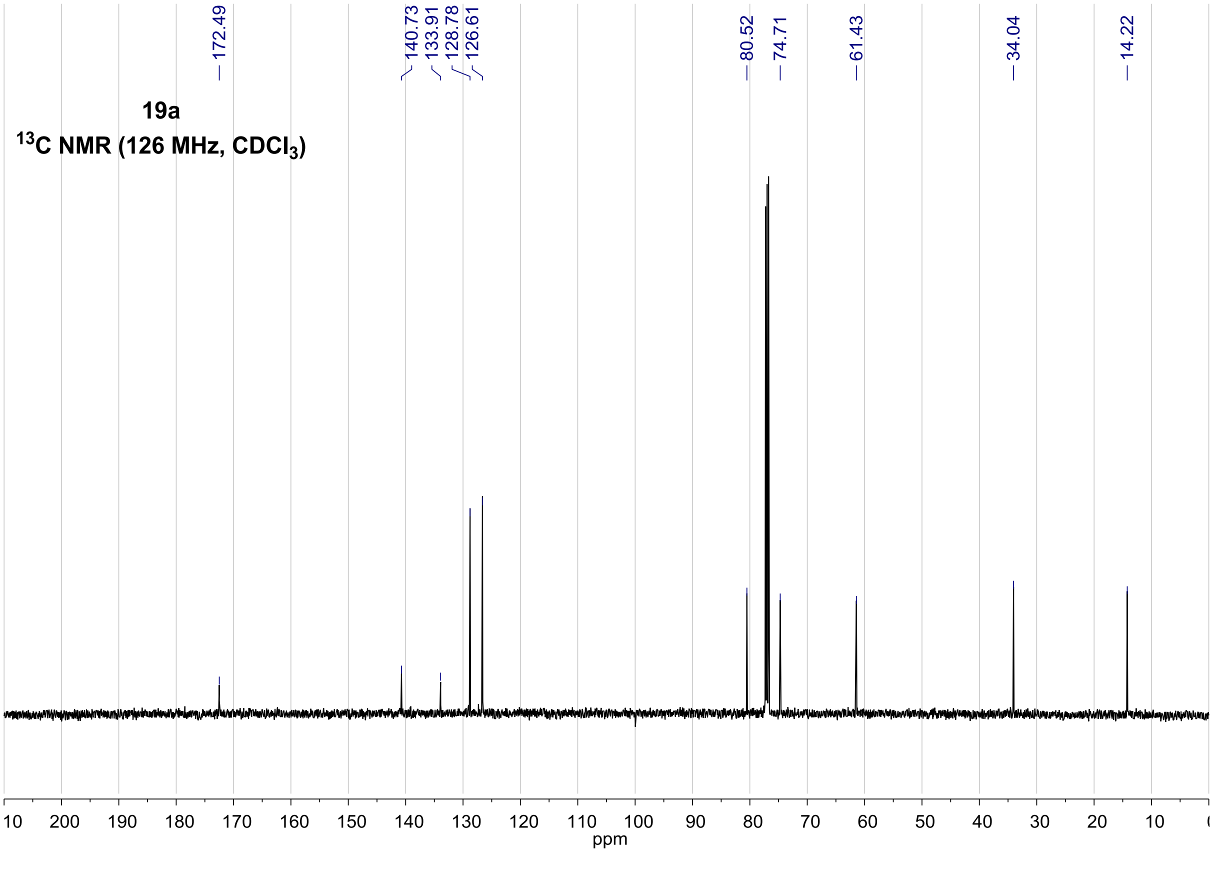
19a
13C NMR (126 MHz, CDCl3)
ppm

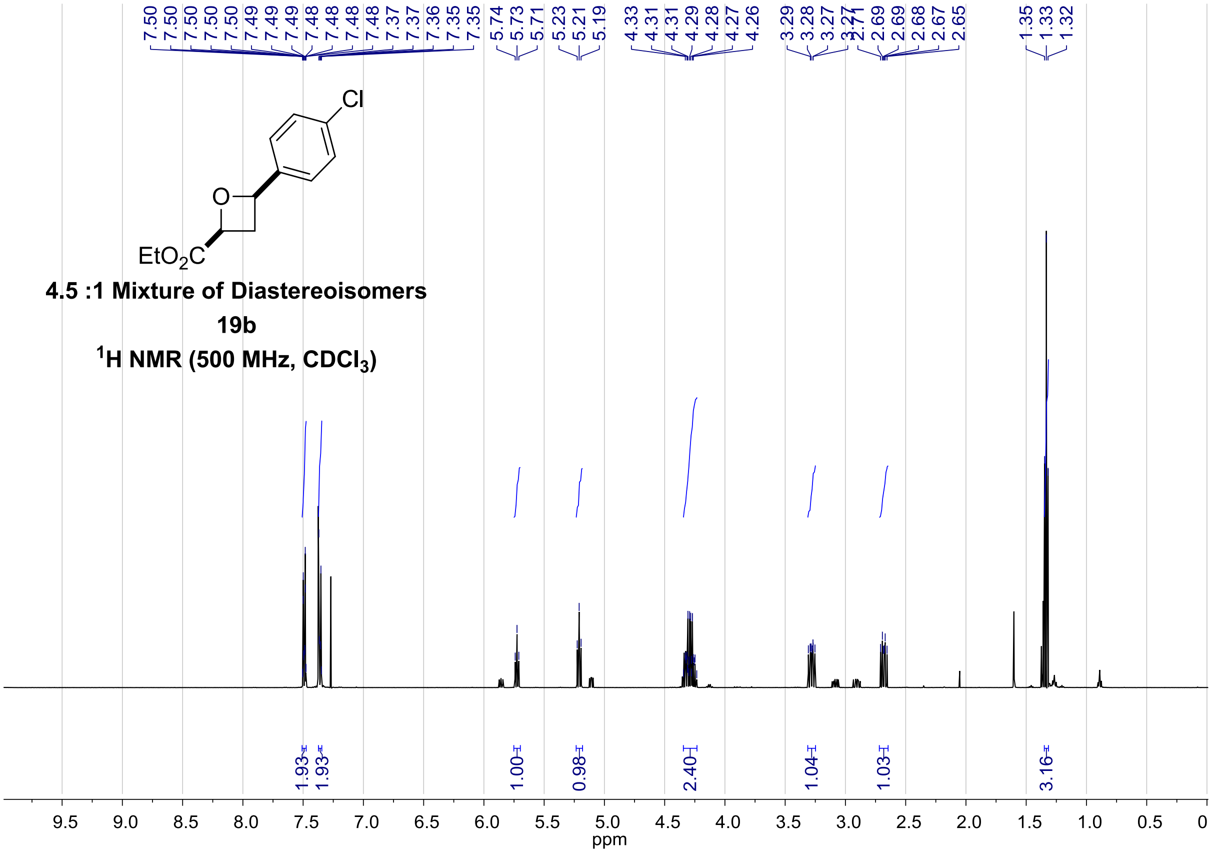
Cl
O
EtO2C
4.5 :1 Mixture of Diastereoisomers
19b
1H NMR (500 MHz, CDCl3)
7.50 7.50 7.50 7.50 7.49 7.49 7.49 7.48 7.48 7.48 7.37 7.37 7.36 7.35 7.35
5.74 5.73 5.71
5.23 5.21 5.19
4.33 4.31 4.31 4.29 4.28 4.27 4.26
3.29 3.28 3.27 3.27 2.71 2.69 2.69 2.68 2.67 2.65
1.35 1.33 1.32
1.93 1.93 1.00 0.98 2.40 1.04 1.03 3.16
ppm

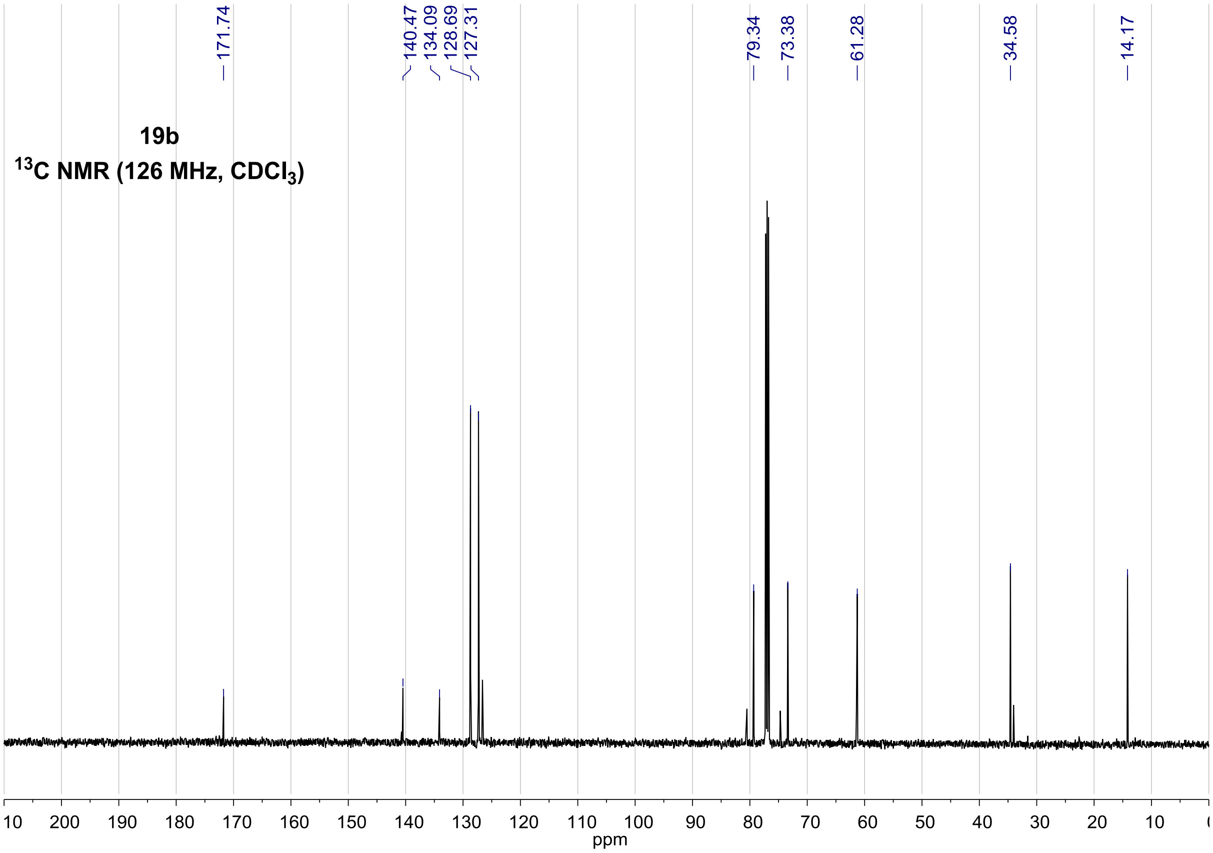
19b
13C NMR (126 MHz, CDCl3)
171.74
140.47 134.09 128.69 127.31
79.34 73.38 61.28 34.58 14.17
ppm

Selective NOE of *trans*-**19a** – Irradiation at 5.86 ppm

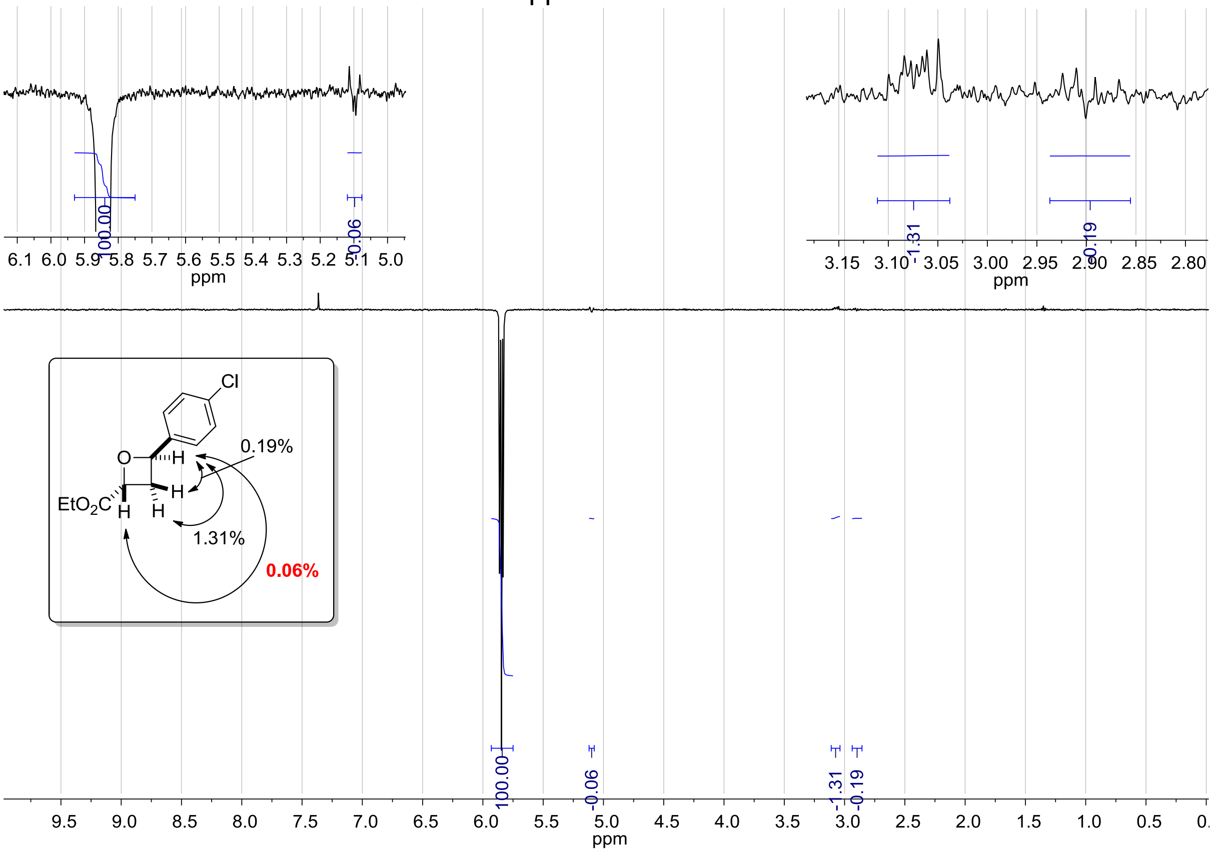

Selective NOE of *cis*-**19b** – Irradiation at 5.73 ppm

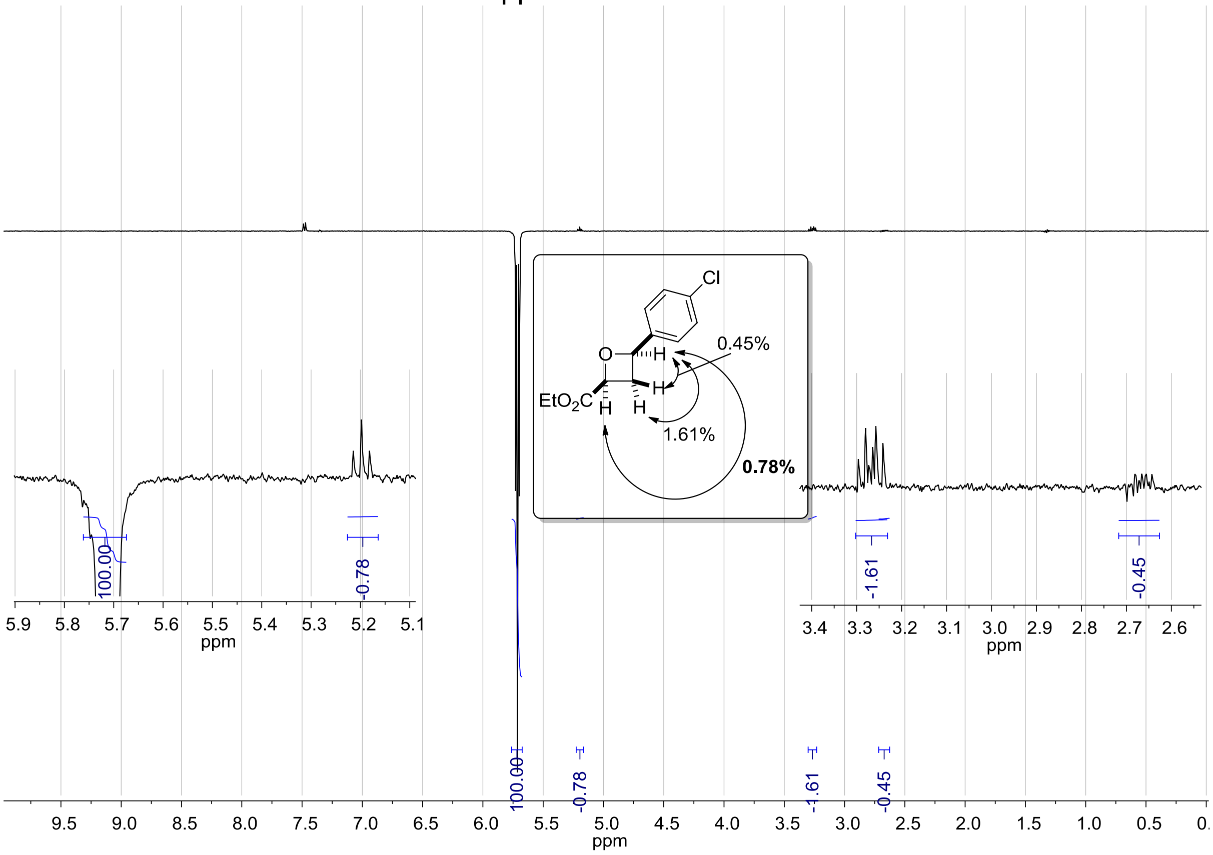

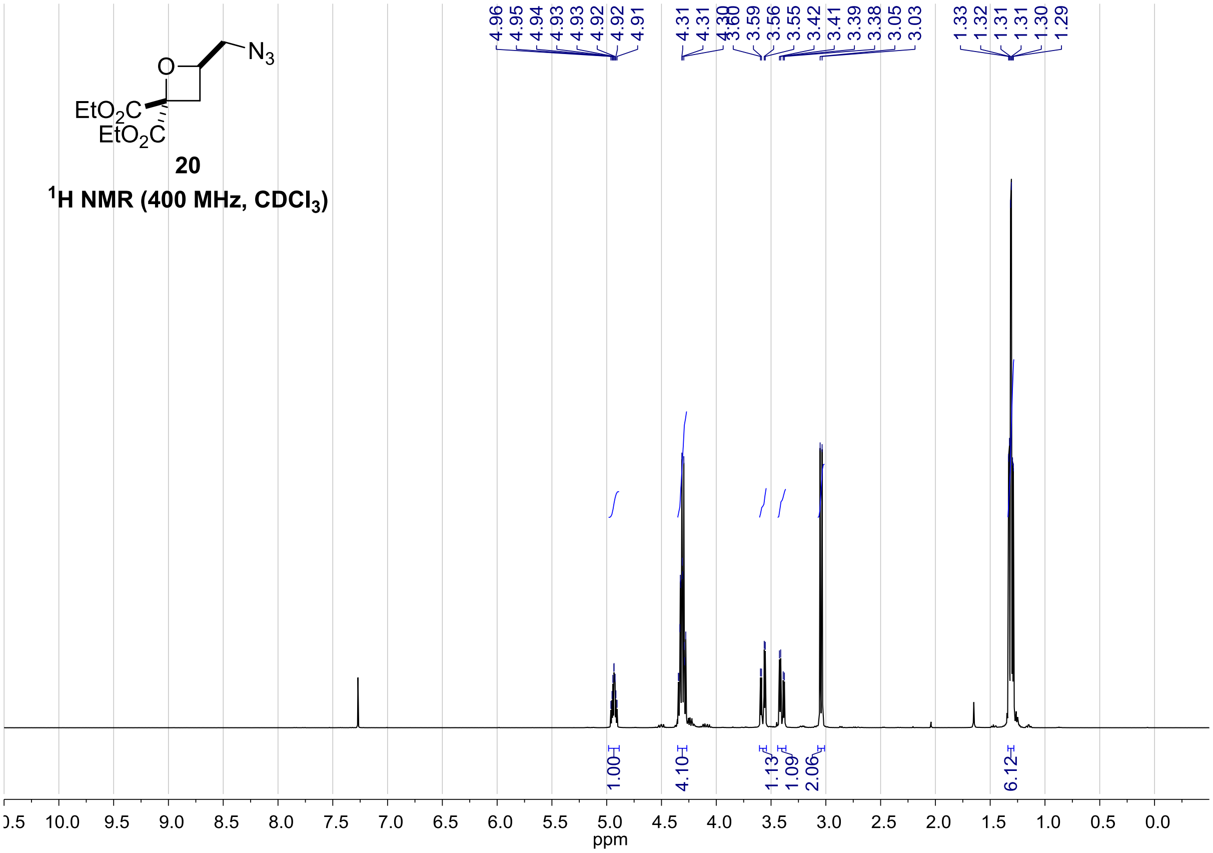

20

^{1}H NMR (400 MHz, $CDCl_3$)

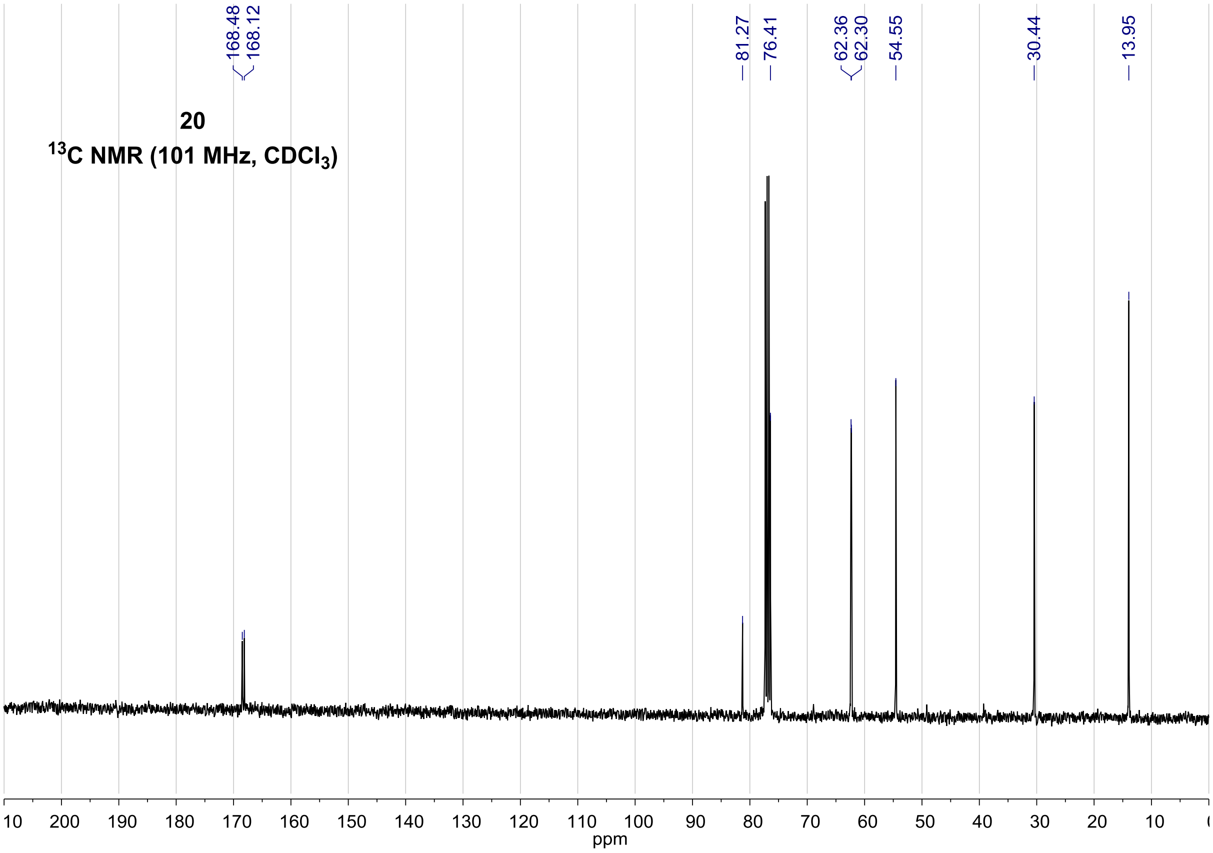

20

^{13}C NMR (101 MHz, $CDCl_3$)

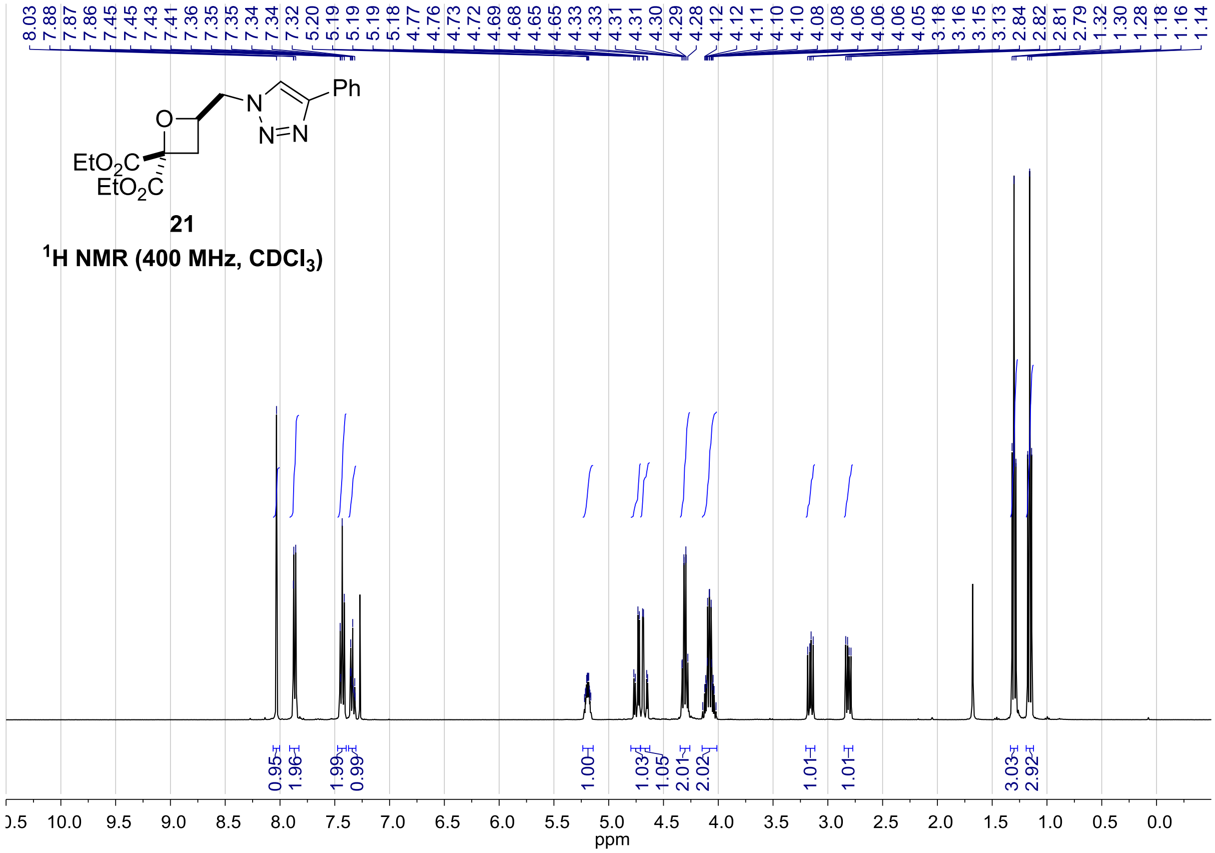
8.03 7.88 7.87 7.86 7.45 7.45 7.43 7.41 7.36 7.35 7.35 7.34 7.34 7.32 5.20 5.19 5.19 5.19 5.18 4.77 4.76 4.73 4.72 4.69 4.68 4.65 4.65 4.33 4.33 4.31 4.31 4.30 4.29 4.28 4.12 4.12 4.11 4.10 4.10 4.08 4.08 4.06 4.06 4.06 4.05 3.18 3.16 3.15 3.13 2.84 2.82 2.81 2.79 1.32 1.30 1.28 1.18 1.16 1.14
Ph
N
O
N=N
EtO2C
EtO2C
21
1H NMR (400 MHz, CDCl3)
0.95 1.96 1.99 0.99 1.00 1.03 1.05 2.01 2.02 1.01 1.01 3.03 2.92
ppm

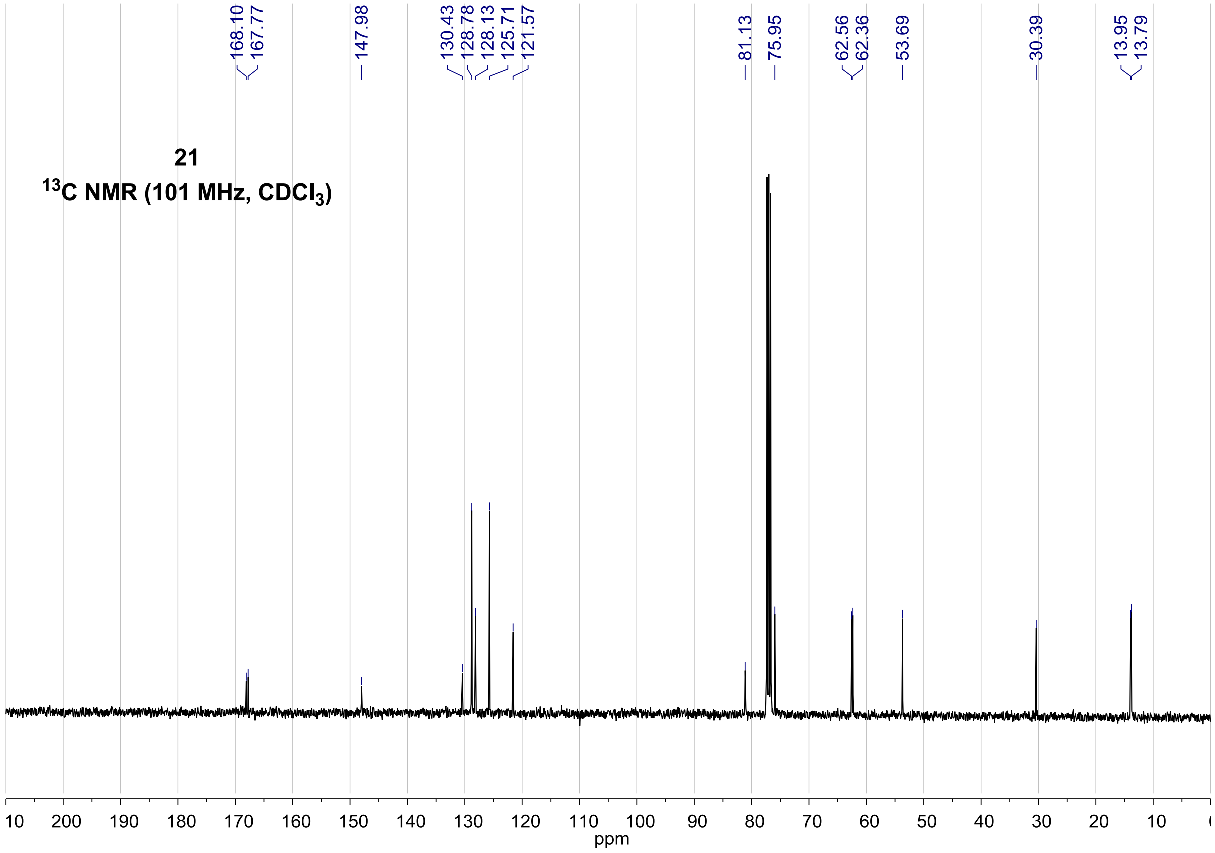
168.10 167.77 147.98 130.43 128.78 128.13 125.71 121.57 81.13 75.95 62.56 62.36 53.69 30.39 13.95 13.79
21
13C NMR (101 MHz, CDCl3)
ppm

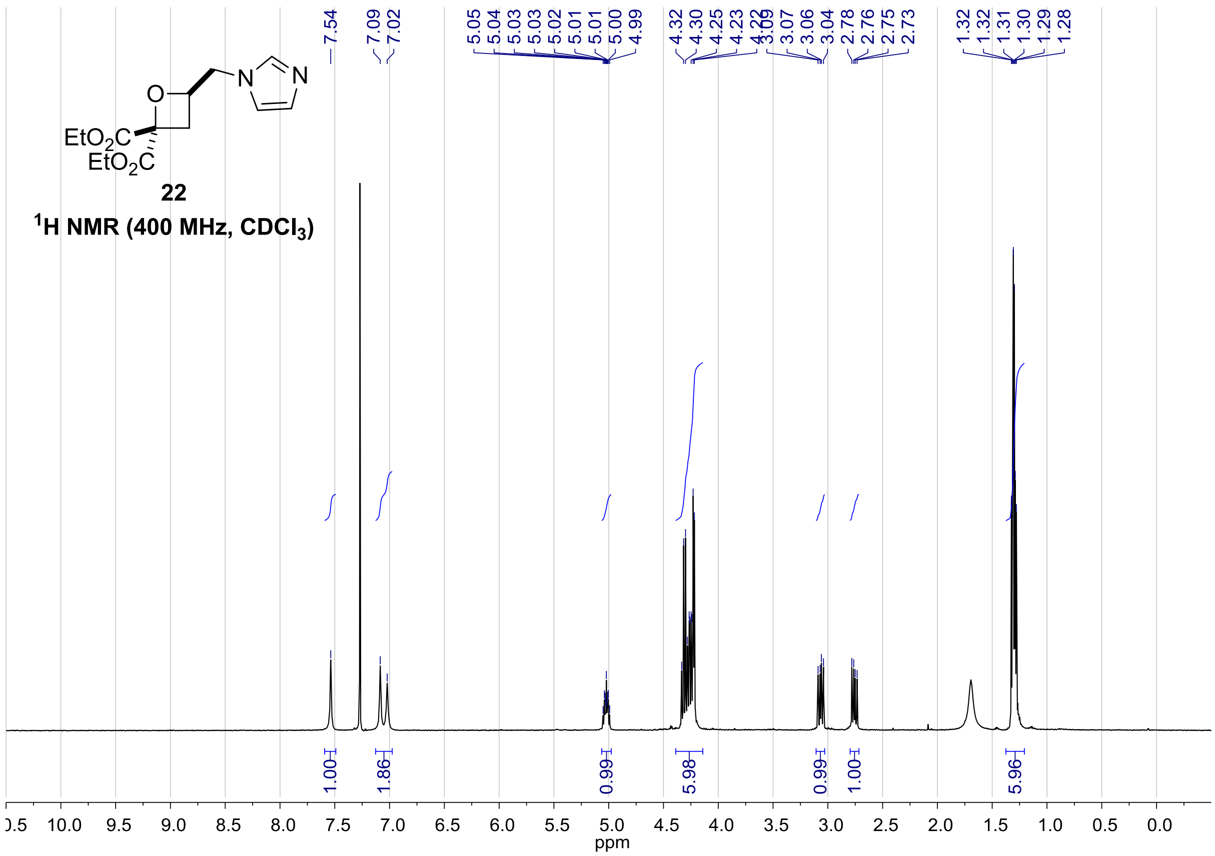
O
N
N
EtO2C
EtO2C
22
1H NMR (400 MHz, CDCl3)
7.54
7.09
7.02
5.05
5.04
5.03
5.03
5.02
5.01
5.01
5.00
4.99
4.32
4.30
4.25
4.23
3.07
3.06
3.04
2.78
2.76
2.75
2.73
1.32
1.32
1.31
1.30
1.29
1.28
1.00
1.86
0.99
5.98
0.99
1.00
5.96
ppm

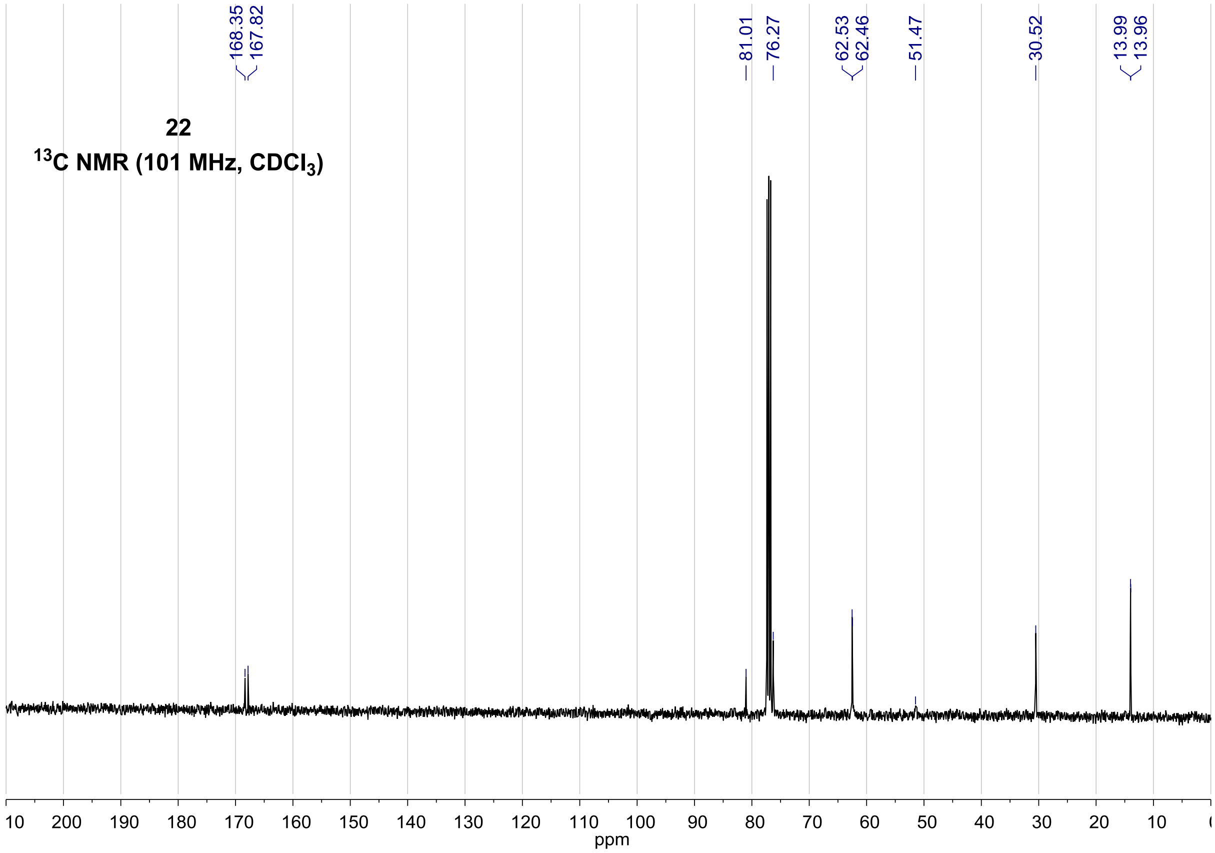
22
13C NMR (101 MHz, CDCl3)
168.35
167.82
81.01
76.27
62.53
62.46
51.47
30.52
13.99
13.96
ppm

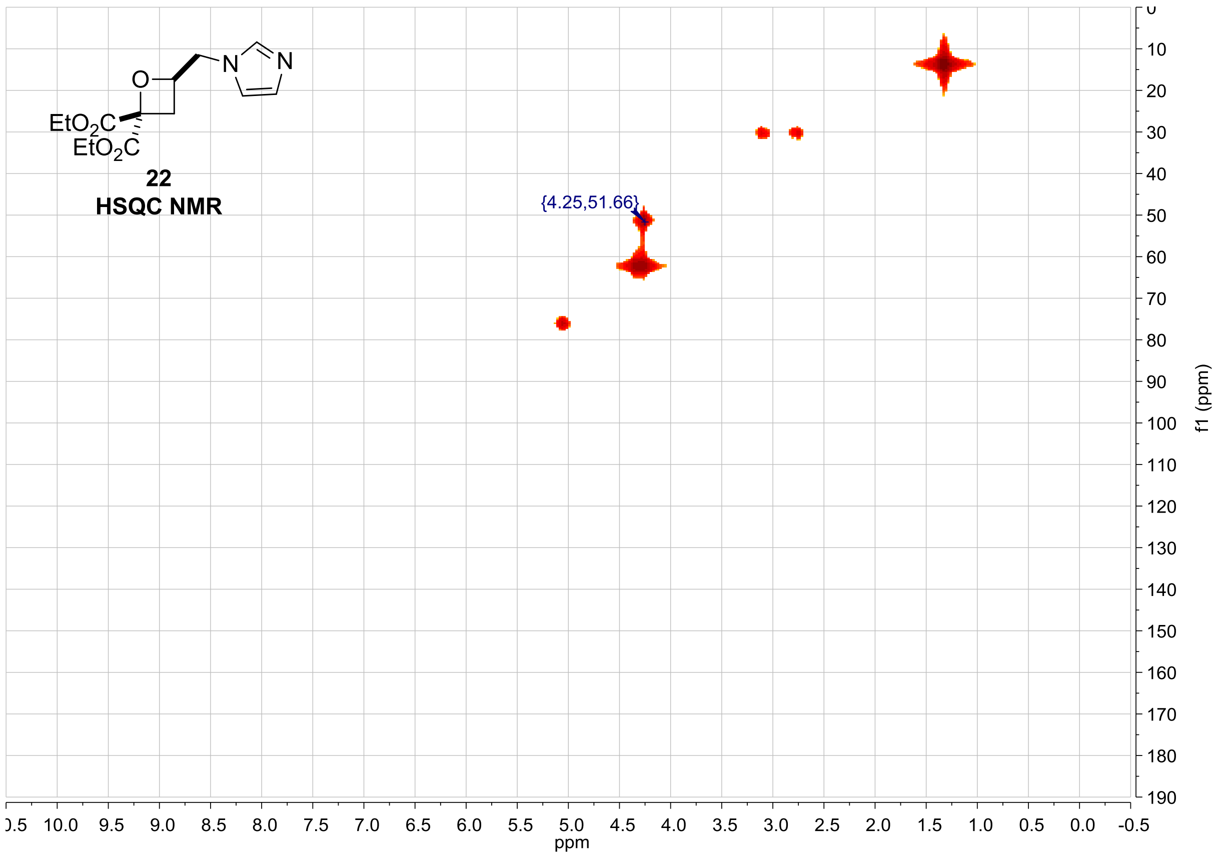

EtO2C
EtO2C
O
N
N
22
HSQC NMR
{4.25,51.66}
f1 (ppm)
ppm
0 10 20 30 40 50 60 70 80 90 100 110 120 130 140 150 160 170 180 190
10.0 9.5 9.0 8.5 8.0 7.5 7.0 6.5 6.0 5.5 5.0 4.5 4.0 3.5 3.0 2.5 2.0 1.5 1.0 0.5 0.0 -0.5

References

[1] S. Fritz, J. Moya, M. Unthank, E. McGarrigle, V. Aggarwal, *Synthesis* **2012**, *44*, 1584.
[2] P. Phukan, P. Chakraborty, D. Kataki, *J. Org. Chem.* **2006**, *71*, 7533.
[3] G. K. Dewkar, S. V. Narina, A. Sudalai, *Org. Lett.* **2003**, *5*, 4501.
[4] M. K. Agrawal, S. Adimurthy, B. Ganguly, P. K. Ghosh, *Tetrahedron* **2009**, *65*, 2791.
[5] B. F. Sels, D. E. De Vos, P. A. Jacobs, *J. Am. Chem. Soc.* **2001**, *123*, 8350.
[6] R. E. Lutz, R. K. Allison, G. Ashburn, P. S. Bailey, M. T. Clark, J. F. Codington, A. J. Deinet, J. A. Freek, K. C. Nicodemus, R. J. Rowlett, N. H. Shearer, J. D. Smith, J. W. Wilson, *J. Org. Chem.* **1947**, *12*, 617.
[7] M. A. Reddy, K. Surendra, N. Bhanumathi, K. R. Rao, *Tetrahedron* **2002**, *58*, 6003.
[8] A. K. Macharla, R. C. Nappunni, N. Nama, *Tetrahedron Lett.* **2012**, *53*, 1401.
[9] B. T. Cho, S. K. Kang, M. S. Kim, S. R. Ryu, D. K. An, *Tetrahedron* **2006**, *62*, 8164.
[10] N. B. Chapman, K. Clarke, D. J. Harvey, *J. Chem. Soc. C* **1971**, 1202.
[11] M. Bio, M. Waters, G. Javadi, Z. Song, F. Zhang, D. Thomas, *Synthesis* **2008**, 891.
[12] R. Perrone, F. Berardi, M. Leopoldo, V. Tortorella, M. D. Lograno, E. Daniele, S. Govoni, *J. Med. Chem.* **1992**, *35*, 3045.
[13] J. Ren, W. Dong, B. Yu, Q. Wu, D. Zhu, *Tetrahedron: Asymmetry* **2012**, *23*, 497.
[14] R. M. Haak, F. Berthiol, T. Jerphagnon, A. J. A. Gayet, C. Tarabiono, C. P. Postema, V. Ritleng, M. Pfeffer, D. B. Janssen, A. J. Minnaard, et al., *J. Am. Chem. Soc.* **2008**, *130*, 13508.
[15] S. Wei, R. Messerer, S. B. Tsogoeva, *Chem. Eur. J.* **2011**, *17*, 14380.
[16] J. Luis Olivares-Romero, E. Juaristi, *Tetrahedron* **2008**, *64*, 9992.
[17] S. Takano, T. Sugihara, T. Kamikubo, K. Ogasawara, *Heterocycles* **1991**, *32*, 1587.
[18] H. Sharghi, M. M. Eskandari, *Synthesis* **2002**, 1519.
[19] N. Iranpoor, H. Firouzabadi, *Can. J. Chem.* **2006**, *84*, 69.
[20] F. Camps, V. Gasol, A. Guerrero, *Synthesis* **1987**, 511.
[21] http://www.sigmaaldrich.com/catalog/product/aldrich/372404?lang=en&region=GB
[22] H. Kotsuki, T. Shimanouchi, R. Ohshima, S. Fujiwara, *Tetrahedron* **1998**, *54*, 2709.
[23] http://www.sigmaaldrich.com/catalog/product/aldrich/718076?lang=en&region=GB
[24] http://www.sigmaaldrich.com/catalog/product/aldrich/184489?lang=en&region=GB
[25] B. Tamami, I. Ghazi, H. Mahdavi, *Synth. Commun.* **2002**, *32*, 3725.
[26] M. Kandula, WO 2014/87323 A2, 2014.
[27] F. Goto, N. Takemura, T. OTani, T. Hasegawa, H. Tsubouchi, N. Utsumi, S. Fujita, H. Kuroda, T. Shitsuta, H. Sasaki, EP1553088 A1, 2005.
[28] S. C. Busman, G. D. Cuny, K. A. Zaklika, R. J. Ellis, US 1998/5756689 A1, 1998.
[29] P. Yates, B. L. Shapiro, N. Yoda, J. Fugger, *J. Am. Chem. Soc.* **1954**, *79*, 5756.
[30] G. M. Green, N. P. Peet, W. A. Metz, *J. Org. Chem.* **2001**, *66*, 2509.
[31] J. S. Bradshaw, K. E. Krakowiak, G. C. Lindh, R. M. Izatt, *Tetrahedron* **1987**, *43*, 4271.
[32] G. R. Newkome, J. D. Sauer, G. L. McClure, *Tetrahedron Lett.* **1973**, *14*, 1599.
[33] G. Heublein, H. Schütz, A. Zschunke, *Tetrahedron* **1969**, *25*, 4225.
[34] H. Masuda, K. Takase, M. Nishio, A. Hasegawa, Y. Nishiyama, Y. Ishii, *J. Org. Chem.* **1994**, *59*, 5550.
[35] Sharghi, H.; Massah, A. R.; Eshghi, H.; Niknam, K. *J. Org. Chem.* **1998**, *63*, 1455.
[36] J. Xu, S. Xu, *Synthesis* **2004**, 276.
[37] A. K. Gupta, R. J. Kazlauskas, *Tetrahedron: Asymmetry* **1993**, *4*, 879.
[38] M. Julia, H. Mestdagh, *Tetrahedron* **1984**, *40*, 327.
[39] T. J. Baker, D. F. Wiemer, *J. Org. Chem.* **1998**, *63*, 2613.
